# Photocatalytic Carboxylate to Sulfinamide Switching Delivers a Divergent Synthesis of Sulfonamides and Sulfonimidamides

**DOI:** 10.1021/jacs.3c07974

**Published:** 2023-09-22

**Authors:** Jonathan A. Andrews, Jagadeesh Kalepu, Christopher F. Palmer, Darren L. Poole, Kirsten E. Christensen, Michael C. Willis

**Affiliations:** † Department of Chemistry, University of Oxford, Mansfield Road, Oxford OX1 3TA, U.K.; ‡ Evotec (U.K.) Limited, 114 Innovation Drive, Milton Park, Abingdon OX14 4RZ, U.K.; § GlaxoSmithKline Medicines Research Centre, Gunnels Wood Road, Stevenage, SG1 2NY, U.K.

## Abstract

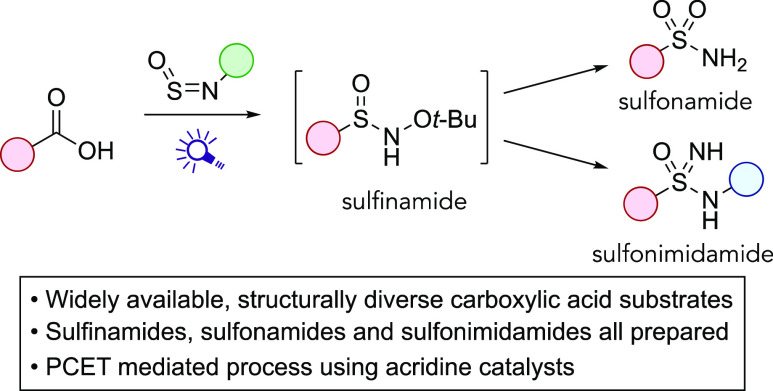

sulfinamides, sulfonamides, and sulfonimidamides are in-demand motifs in medicinal chemistry, yet methods for the synthesis of alkyl variants that start from simple, readily available feedstocks are scarce. In addition, bespoke syntheses of each class of molecules are usually needed. In this report, we detail the synthesis of these three distinct sulfur functional groups, using readily available and structurally diverse alkyl carboxylic acids as the starting materials. The method harnesses alkyl radical generation from carboxylic acids using acridine photocatalysts and 400 nm light with subsequent radical addition to sulfinylamine reagents, delivering sulfinamide products. Using the *N*-alkoxy sulfinylamine reagent *t*-BuO-NSO as the radical trap provides common *N*-alkoxy sulfinamide intermediates, which can be converted in a divergent manner to either sulfonamides or sulfonimidamides, by treatment with sodium hydroxide, or an amine, respectively. The reactions are scalable, tolerate a broad range of functional groups, and can be used for the diversification of complex biologically active compounds.

## Introduction

**Figure 1:**
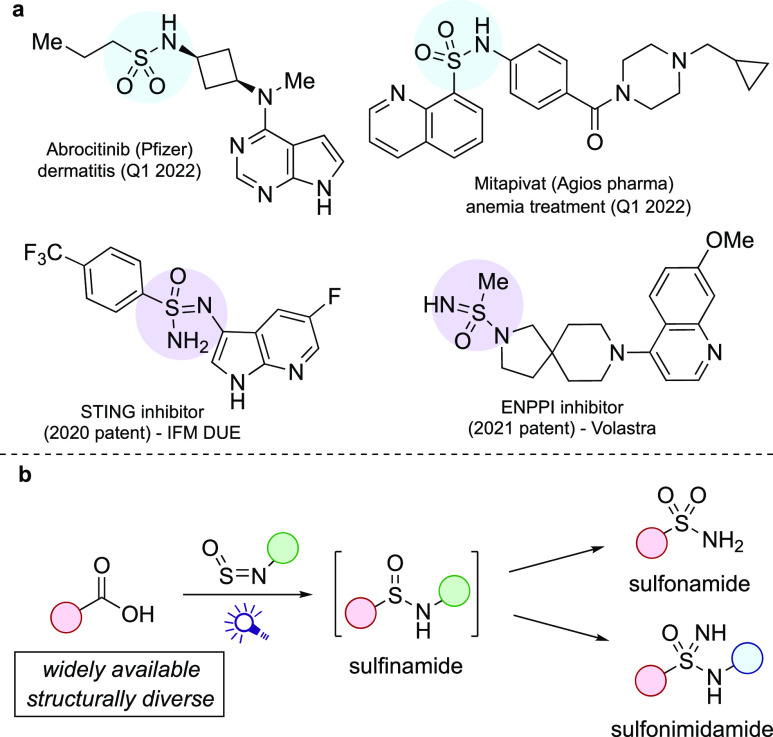
(a) Examples of bioactive sulfonamides and sulfonimidamides. (b) This approach; a sulfinamide intermediate that progresses to either sulfonamide or sulfonimidamide.

Transformations that convert readily available organic building blocks into topologically distinct, value-added molecules are particularly attractive in discovery chemistry.^1^ In this context, carboxylic acids have emerged as versatile substrates; they enjoy wide commercial availability and display broad structural diversity, and when combined with photocatalytic methods they can be converted into a plethora of functional groups.^2^ Attracted by the variance offered with carboxylic acids, we conceived of an approach in which these substrates could be converted to a family of structurally distinct, high-value products using only a single reagent class and catalyst system. The targets selected were sulfonamides, sulfonimidamides, and sulfinamides. Sulfonamides are the dominant sulfur functional group in bioactive molecules;^3^ they are present in almost 10% of FDA-approved medicines and can be found in pharmaceuticals used against a broad range of indications (Figure 1a).^4^ Sulfonimidamides, the monoaza variants of sulfonamides, are yet to appear in any marketed drugs,^5^ but the recent patent literature attests to their burgeoning profile as molecules active against varied biological targets.^6^ Sulfinamides are a lower oxidation-state functional group and are used as amide bioisosteres,^7^ with applications in areas as diverse as hepatitis C^8^ and leukemia.^9^ Sulfinamides are also high-value synthetic intermediates, providing a segue to diverse sulfur functional groups.^10^ A method that converts carboxylic acids directly into sulfonamides, sulfonimidamides, or sulfinamides would be a powerful addition to the repertoire of transformations available to discovery chemists.

Our reaction design is shown in Figure 1b, and involves the initial conversion of a carboxylic acid into sulfinamide, which can be the final product, or by choice of subsequent reaction conditions is directly converted into a sulfonamide or a sulfonimidamide.

**Scheme 1:**
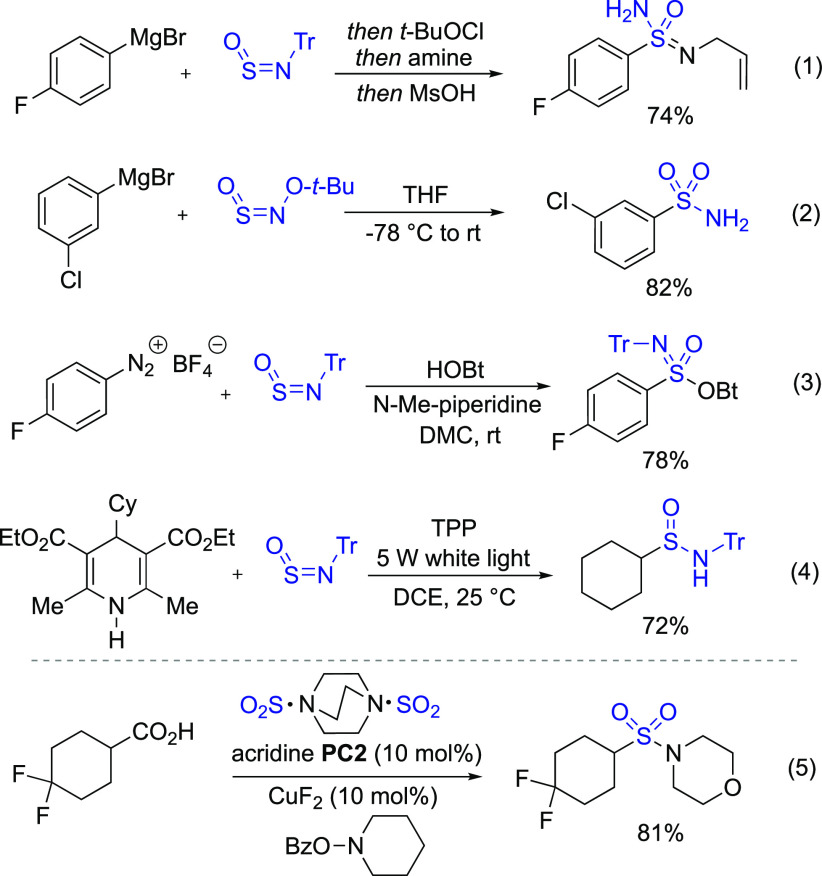
Uses of Stable Sulfinylamine Reagents, and Larionov’s Acridine-Catalyzed Decarboxylative Sulfonamide Synthesis

The direct conversion of carboxylic acids into sulfinamides has not been reported. However, we reasoned that carbon-centered radicals generated by decarboxylation should be added to an appropriate sulfinylamine reagent, which following H atom transfer would provide the desired sulfinamide. Sulfinylamines have been known for many years,^11^ although their use in synthesis has been limited by the high reactivity, and corresponding instability, associated with many reagents of this type.^12,13^ To address this, we have recently reported several sulfinylamine reagents that all display good stability, engendered by steric or electronic control,^14^ many of which are now commercially available. These new reagents undergo ready addition of preformed organometallic nucleophiles and have been exploited in the synthesis of a range of sulfur(IV) and sulfur(VI) functional groups, including sulfonimidamides^14a^ and sulfonamides^14b^ (Scheme 1, eqs 1 and 2). Sulfinylamine reagents have also been combined with carbon-centered radicals; Bolm^15^ and Liu^16^ have shown that aryl radicals generated from aryl diazonium salts can be employed, and Zhao and Li demonstrated that substituted Hantzsch ester-type reagents can be used to transfer alkyl groups (Scheme 1, eqs 3 and 4).^17^ While all of these processes work well, the variety of carbon-based reagents employed are not ideal for use in discovery chemistry, as preformed organometallics show poor functional group tolerance due to their high reactivity, diazonium salts are high-energy species that often require individual safety assessment,^18^ and Hantzsch ester-type reagents have very limited availability. The use of alkyl carboxylic acids as substrates would address all of these concerns.

## Results and Discussion

Of the many known methods to effect decarboxylation, we were drawn to the acridine catalysis originally developed by Oda,^19^ and more recently exploited by Larionov and co-workers.^20^ Using these catalysts would avoid the use of strong photo-oxidants, which we considered likely incompatible with the sulfinamide products. Of most relevance is Larionov’s report of decarboxylative amidosulfonation using acridine photocatalysts and DABSO as a SO_2_ trap (Scheme 1, eq 5),^21^ where the decarboxylation is proposed to take place via a proton-coupled electron transfer from a singlet-excited complex between the acid and catalyst.^22^ Using this approach, Larionov showed that sulfonamides were accessible using hydroxylamine derivatives in combination with copper catalysts or from anilines under oxidative conditions.^21a^

Before embarking on our proposed divergent synthesis of sulfonamides or sulfonimidamides, we first focused on sulfinamides and in establishing that they were accessible using a decarboxylative approach with sulfinylamines employed as radical traps. Accordingly, we initiated our studies using hydrocinnamic acid (0.2 mmol) and *N*-sulfinyltritylamine (Tr-NSO) (0.3 mmol) in combination with acridine photocatalyst **PC1** and 395–405 nm light-emitting diode (LEDs); using these conditions sulfinamide **1a** was isolated in 69% yield (Table 1, entry 2). We undertook a round of optimization, with the major improvements being the switch to acridine catalyst **PC2**, which improved the yield of N-Tr-sulfinamide **1a** to 98% (entry 9). We also established that reasonable variation of the reaction solvent was possible (entries 3–8), although dichloromethane remained optimal.

**Table 1:**
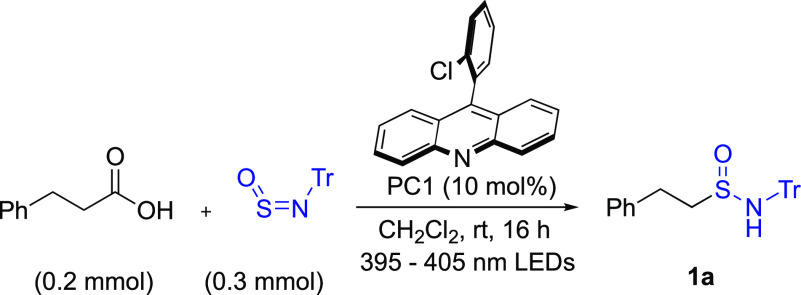

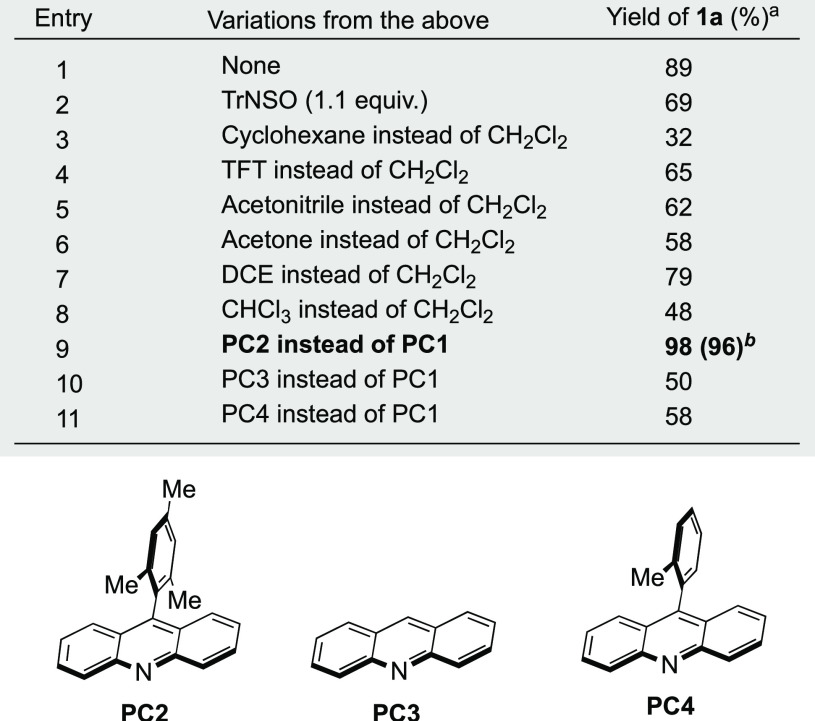
Decarboxylative Synthesis of Sulfinamide **1a**^a^

a Yields of **1a** calculated from HPLC analysis using 1,3,5-triisopropylbenzene as in internal standard.

b Isolated yield.

With an optimized system for the synthesis of N-Tr sulfinamides in hand, we explored the scope of the reaction with respect to the carboxylic acid substrate (Table 2). We found that a broad range of primary (**1a**–**1m**), secondary (**1n**–**1u**), and tertiary (**1w**–**1x**) alkyl carboxylic acids were compatible with the chemistry. Benzylic substrates could also be used (**1v**), but gave only moderate yields, likely due to rapid dimerization of the benzylic radicals. Varied functional groups, including alkenes (**1g**), alkynes (**1h**), ketones (**1i**), esters (**1j**, **1y** and **1z**), sulfones (**1t**), sulfonamides (**1u**), carbamate (**1z**), and free alcohols (**1aa** and **1ab**), were well tolerated. In addition, aromatic groups featuring bromo- (**1b**), nitro- (**1c**), and hydroxyl (**1aa**) substitutions were viable starting materials, as were substrates featuring the aromatic heterocycles pyridine (**1k**), furan (**1l**), and NH-indole (**1m**). When cyclopropaneacetic acid was used, the ring-opened product **1f** was formed in 90% yield, supporting the radical nature of the reaction. Several more complex sulfinamides, including a bicyclopropane example (**1y**), those derived from the amino acid derivative Boc-Glu-O*t*Bu (**1z**), the marketed drug mycophenolic acid (**1aa**), and the steroid natural product chenodeoxycholic acid (**1ab**), were obtained in good yields. Importantly, the transformation was equally effective on a preparative scale, with a gram-scale reaction providing sulfinamidine **1u** in 88% yield.

**Table 2:**
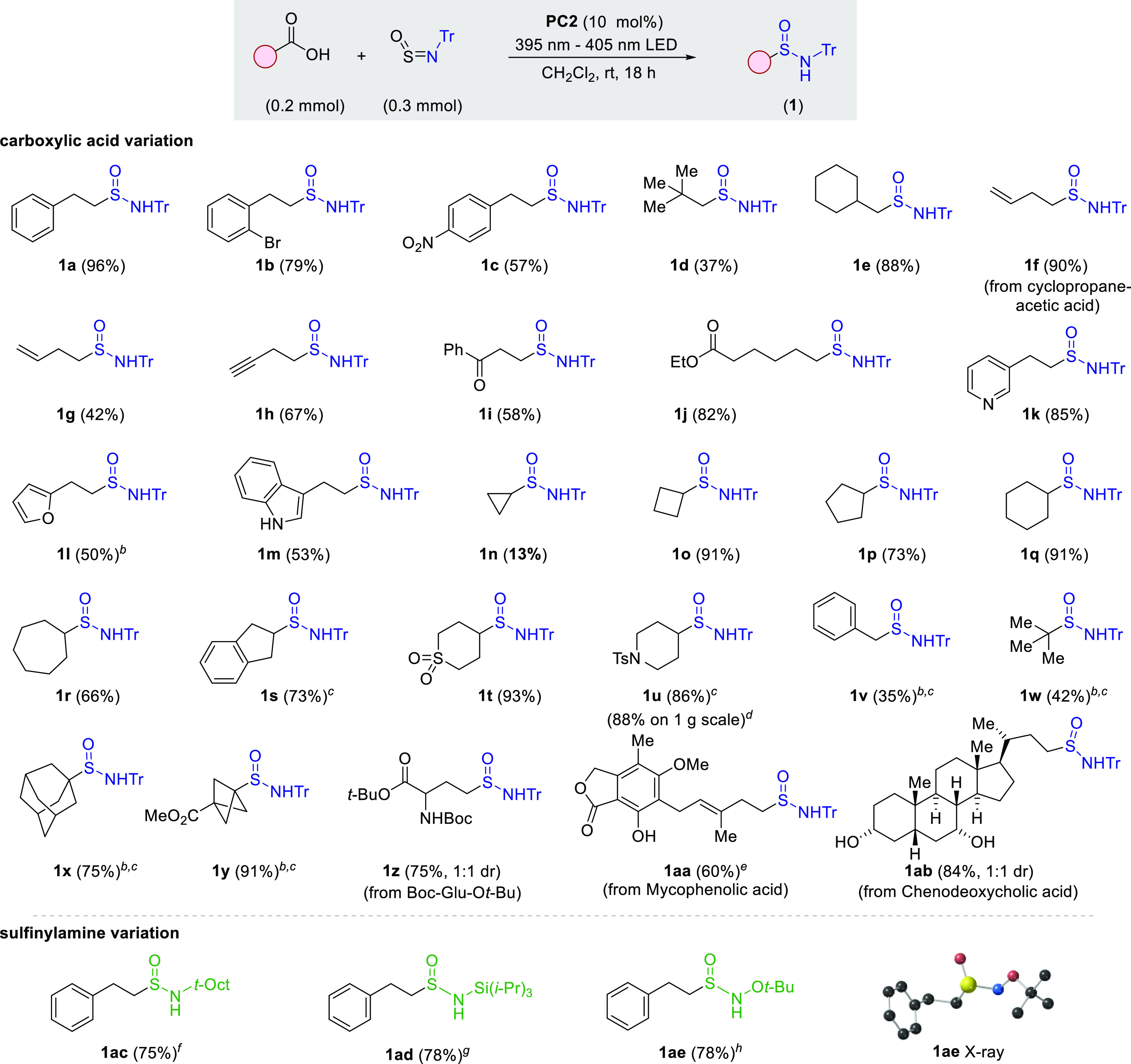
Decarboxylative Synthesis of Sulfinamides 1^a^

a Reaction conditions: (i) carboxylic acid (0.2 mmol), R-NSO (0.3 mmol), **PC2** (10 mol %), 395–405 nm LEDs, CH_2_Cl_2_ (4 mL), rt, and 18 h. Isolated yields.

b Carboxylic acid (0.3 mmol), Tr-NSO (0.2 mmol).

c CH_2_Cl_2_ (1 mL).

d Using **PC1**.

e **PC2** (20 mol %).

f Using *t*-Oct-NSO in place of Tr-NSO.

g Using (*i*-Pr)_3_Si-NSO (0.2 mmol) in place of Tr-NSO, carboxylic acid (0.3 mmol).

h Using *t*-BuO-NSO (0.2 mmol) in place of Tr-NSO, carboxylic acid (0.3 mmol).

Variation of the sulfinylamine reagent was also possible, with the *N*-*t*-octyl (**1ac**)^14c^ and *N*-Si(*i*-Pr)_3_ (**1ad**) substituted reagents,^14d^ both of which benefit from steric-stabilization, providing the corresponding sulfinamides in high yields. More importantly for our proposed divergent synthesis, the N-alkoxy sulfinamide **1ae**, derived from the *N*-*O*-*t*-Bu substituted sulfinylamine reagent,^14b^ was obtained in 78% yield. The structure of sulfinamide **1ae** was confirmed by single crystal X-ray analysis (CCDC 2209822).^23^

Having established an efficient and general route to alkyl sulfinamides, we next focused on access to sulfonamides and sulfonimidamides. The successful synthesis of *N*-*t*-butoxy sulfinamides (as in **1ae**) was key to our proposed divergent route to both sulfonamides and sulfonimidamides; in our initial report of the *t*-Bu-ONSO reagent, we showed that reaction with preformed organometallic reagents led directly to primary sulfonamide products, with the reaction proceeding via elimination of isobutene from an anionic sulfonimidate ester.^14b^ We reasoned that treatment of *t*-butoxy sulfinamides with an appropriate base would facilitate rearrangement to similar anionic sulfonimidate esters, which would then collapse to primary sulfonamides. Simply adding a solution of sodium hydroxide in isopropanol was sufficient to achieve efficient formation of the desired primary sulfonamides. Using these reaction conditions, we explored the scope with respect to the carboxylic acid (Table 3) and found the process to be compatible with primary (**2a**,**b**), secondary (**2c**,**d**), and tertiary substrates (**2f**). Protected amine (**2d**) and unprotected hydroxyl groups (**2g**) were well tolerated in the reaction, giving good yields of the primary sulfonamide products. Substrates containing esters susceptible to hydrolysis required alternative reaction conditions, and in these cases, sodium hydride could be used as a base; sulfonamides **2h** and **2i** were obtained using this modified protocol.

**Table 3:**
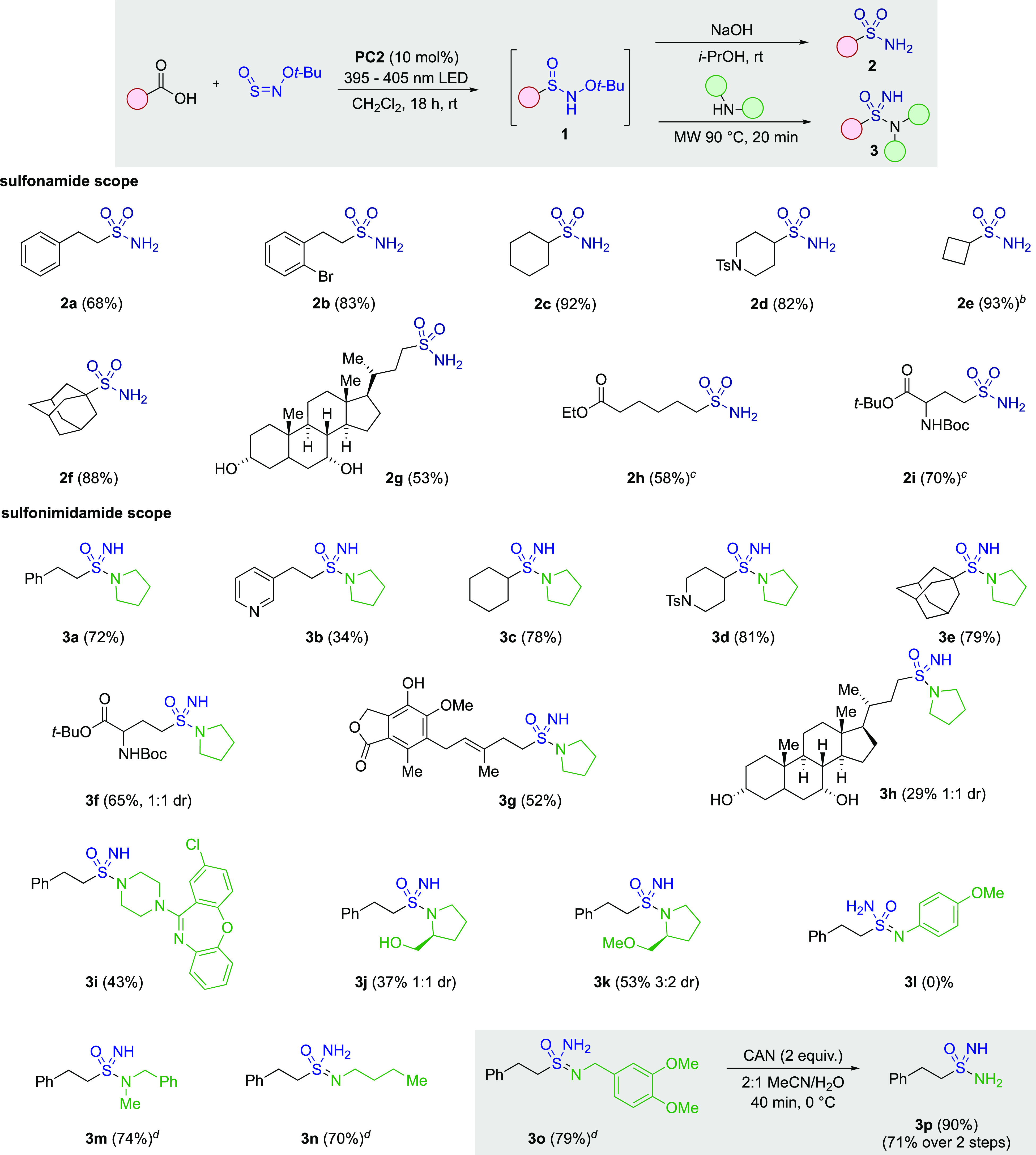
Divergent Synthesis of Sulfonamides and Sulfonimidamides from Carboxylic Acids^a^

a Reaction conditions: (i) carboxylic acid (0.3 mmol), *t*-BuO-NSO (0.2 mmol), **PC2** (10 mol %), 395–405 nm LEDs, CH_2_Cl_2_ (2 mL), rt, 16 h, then NaOH (2 equiv) in *i*-PrOH (0.4 M), rt, 16 h, or amine (2 equiv), toluene (2 mL), 20 min, 90 °C in microwave reactor. Isolated yields.

b **PC2** (20 mol %).

c NaH (1.3 equiv), THF, 0 °C to rt, 16 h, used in place of NaOH.

d 10 equiv amine used.

To access sulfonimidamide products, we took inspiration from the report of Tummanapalli and co-workers,^24^ who had demonstrated the preparation of sulfonimidamides from the addition of aryllithium reagents to the *t*-BuO-NSO reagent, followed by addition of an amine with heating. The authors of this report proposed a *t*-butyl sulfonimidate ester intermediate, and while we do not observe these esters as intermediates in our system, our success in preparing primary sulfonamides (i.e., **1ad** → **2a**) led us to be confident in targeting sulfonimidamides (**1ad** → **3a**). Our optimized protocol required a solvent switch following the initial decarboxylative alkoxy sulfinamide synthesis. Accordingly, at the conclusion of the decarboxylative step, the reaction vial was flushed with nitrogen gas to remove the dichloromethane solvent; toluene and the required amine were then added, and the vial was heated in a microwave reactor at 90 °C for 20 min. Using this approach, the reaction was again found to work well with primary (**3a**,**b**), secondary (**3c**,**d**), and tertiary (**3e**) carboxylic acid substrates. A variety of amines were also compatible with the process, although less nucleophilic acyclic secondary or primary amines required higher equivalents to achieve good yields (**3m**–**3o**). Anilines were found to be incompatible with the reaction (**3l**). Varied functional groups were tolerated, including sulfonamides (**3d**), esters (**3f**), and free alcohols (**3g, 3h,** and **3j**), although the methyl ether variant (**3k**) resulted in an improved yield relative to the free alcohol (**3j** vs **3k**). Ammonia was incompatible with the process; however, the primary sulfonimidamide **3p** could be prepared from DMB-derivative **3o** via oxidative cleavage of the DMB-protecting group using ceric ammonium nitrate.

**Scheme 2:**
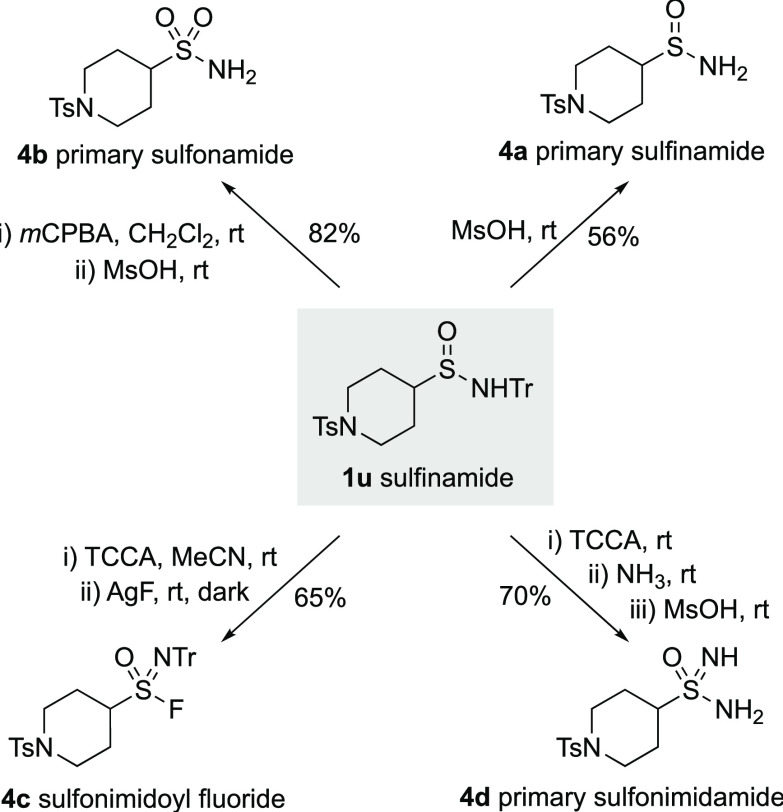
Derivatisation of Sulfinamide **1u** into Diverse Sulfur Functional Groups

To capitalize on the wide variety of sulfinamides available using the developed chemistry, we have established reaction conditions to convert the *N*-Tr sulfinamides into four related functional groups (Scheme 2). *N*-Tr-sulfinamide **1u** was used as a representative example. The trityl group could be removed by simple treatment with MsOH,^25^ providing the primary sulfinamide **4a**. Oxidation at sulfur (*m*CPBA), followed by trityl-deprotection yielded primary sulfonamide **4b**. Sulfonimdoyl fluoride **4c** was available by using a two-step sequence involving oxidative chlorination (TCCA), followed by fluoride displacement. Finally, oxidative chlorination (TCCA) followed by the addition of ammonia and then trityl-deprotection provided primary sulfonimidamide **4d**.

## Conclusions

We have shown that acridine-catalyzed visible-light-mediated, decarboxylative radical additions into sulfinylamines provide efficient access to a broad range of alkyl sulfinamides. A useful modification of this approach uses the *N*-alkoxy sulfinylamine reagent *t*-BuO-NSO and delivers *N*-alkoxy sulfinamide intermediates, which can be converted selectively into either primary sulfonamides or sulfonimidamides. The method is scalable and works on substrates featuring a wide range of functional groups as well as complex, biologically relevant examples.

## Supporting Information Available

Experimental procedures, spectral characterization, and additional data (PDF)
