# Supplementary material for: Photocatalytic Carboxylate to Sulfinamide Switching Delivers a Divergent Synthesis of Sulfonamides and Sulfonimidamides
Source: J Am Chem Soc. 2023 Sep 22;145(39):21623–9. doi: 10.1021/jacs.3c07974 (PMC10557147; doi:10.1021/jacs.3c07974)

Supporting Information

Photo-catalytic Carboxylate to Sulfinamide Switching Delivers a Divergent Synthesis of Sulfonamides and Sulfonimidamides

Jonathan A. Andrews,[a] Jagadeesh Kalepu,[a] Christopher F. Palmer,[b] Darren L. Poole,[c] Kirsten E. Christensen and Michael C. Willis[a,*]

[*] michael.willis@chem.ox.ac.uk

[a]Department of Chemistry, University of Oxford, Chemistry Research Laboratory, Mansfield Road, Oxford, OX1 3TA, UK

[b]Evotec (UK) Limited, 114 Innovation Drive, Milton Park, Abingdon OX14 4RZ, UK

[c]GlaxoSmithKline Medicines Research Centre, Gunnells Wood Road, Stevenage, SG1 2NY, UK

Table of Contents

1. **General information** S-3

  1.1. General Considerations S-3

  1.2. HPLC yields S-4

2. Optimization of conditions S-5

3. Experimental Procedures S-9

  3.1. Photochemical Reaction Setup: S-9

  3.2. General Procedures: S-10

  3.3. Sulfinamide Scope S-12

  3.4. Sulfonamide Scope S-29

  3.5. Sulfonimidamide Scope S-34

  3.6. One-gram scale reaction and derivatizations S-43

  3.7. Synthesis of photocatalysts: S-46

4. References S-51

5. NMR Spectra S-52

1. General information

1.1. General Considerations

Reactions were performed under inert nitrogen atmosphere with anhydrous solvent unless otherwise stated. Reagents were purchased from Sigma-Aldrich, Fisher Scientific, Fluorochem, Tokyo Chemical Industry UK or Strem Chemicals and were used as supplied unless otherwise stated. Other solvents, if stated as anhydrous, were collected fresh from an in-house solvent purification system, which involves passing the solvent through anhydrous alumina columns. Glassware was oven-dried, and allowed to cool to room temperature under nitrogen. Cooling to 0 °C was achieved using ice-water bath. Reactions were monitored by HPLC analysis and/or thin layer chromatography (TLC) using pre-coated aluminium-backed silica plates (Merck Kieselgel 60 F254). Plates were visualised under ultraviolet light (254 nm) followed by staining with $KMnO_4$ or ninhydrin stain. Flash column chromatography was carried out using Geduran® Si 60, 40-63 micron silica gel; the compound to be purified was either loaded as oil or pre-absorbed onto silica. Petrol refers to the fraction of light Petroleum ether boiling in the range 40-60 °C.

Reactions using a microwave reactor were conducted using a Biotage® Initiator+ microwave synthesizer.

^{1}H NMR spectra were obtained using a Bruker AVIII HD 400 (400 MHz), AVII 500 (500 MHz), AVIII HD 500 (500 MHz) NEO 600 (600 MHz) spectrometer using the residual solvent as an internal standard. ^{13}C NMR spectra were obtained on a Bruker AVIII HD 400 (101 MHz), AVII 500 (126 MHz), AVIII HD 500 (126 MHz) or NEO 600 (151 MHz) spectrometer using the ^{1}H decoupling method and the residual solvent as an internal standard. Acquisitions were carried out at room temperature unless otherwise stated. Chemical shifts (δ) are reported in parts per million (ppm) from the residual solvent peak and coupling constants (*J*) were given in Hertz (Hz). Proton multiplicity is assigned using the following abbreviations: singlet (s), doublet (d), triplet (t), quartet (q), quintet (p), multiplet (m), broad (br), apparent (app.).

Low resolution ESI mass spectra were recorded on a Waters LCT premier spectrometer. High resolution mass spectra were recorded either on a Bruker MicroTOF spectrometer under electrospray ionization conditions (ESI), a Waters LCT Premier spectrometer under the conditions of chemical ionization (CI) by the internal service at Chemistry Research Laboratory, University of Oxford, or a Waters BioAccord system. Samples for mass spectra were prepared as 1 mg/mL solution in MeOH (LRMS, HRMS-ESI) or submitted neat (HRMS-CI). Values quoted are a ratio of mass to charge in Daltons. High resolution values are

calculated to four decimal places from the molecular formula, all found within a tolerance of five ppm. Melting point values were found using a Reich Melting Point Apparatus and are reported uncorrected. Infrared spectra were determined using neat samples with a Brüker Tensor 27 FT-IR spectrometer with an internal range of 600 – 4000 cm^{-1} and all absorptions are given in wavenumbers (cm^{-1}).

1.2. HPLC yields

The HPLC data was obtained from an Agilent Technologies 1200 series HPLC Hypersol ODS, 5 μm column (100 x 4.0 mm) using an extended 12 min method H_2O (0.1 % v/v H_3PO_4): MeCN (10 – 95% (9 min) to 100% (10.5 min) to 10% (12 min)). HPLC yields for the ligand screening were obtained via a standard calibration curve using authentic desired product and 1,3,5-triisopropylbenzene as an internal standard. Yields were calculated from absorbances at 210 nm. The method was verified by product isolation which gave a result ± 2%.

2. Optimization of conditions:

Table S1: Solvent screening

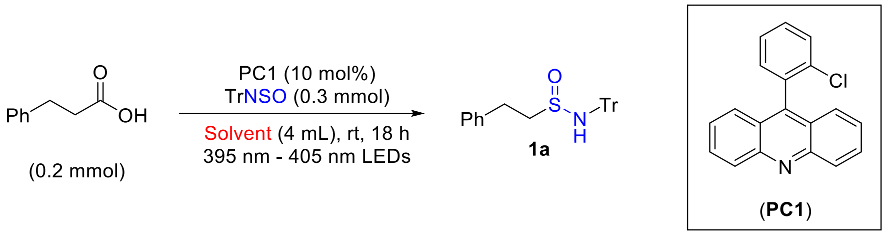

Entry	Solvent	Yield of 1a (%)
**1**	**CH_2Cl_2**	**86**
2	1,2-dichloroethane	79
3	$CHCl_3$	48
4	Trifluorotoluene	65
5	Acetone	58
6	Acetonitrile	62
7	Cyclohexane	32
8	DMF	0
9	DMSO	0
10	EtOH	0
11	THF	0

Reaction conditions: Hydrocinnamic acid (0.2 mmol), TrNSO (0.3 mmol), PC1 (10 mol%), Solvent (4 mL), rt, 18 h. Yields calculated by HPLC analysis using 1,3,5-triisopropylbenzene as an internal standard.

Table S2: Concentration screening

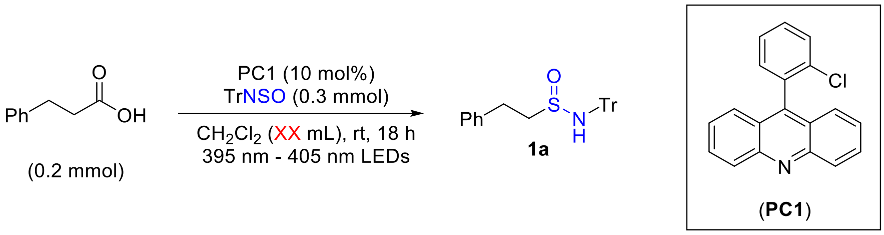

Entry	Solvent Volume (mL)	Reaction Concentration (moldm^{-3})	Yield of 1a (%)
1	1	0.2	63
2	2	0.1	67
**3**	**4**	**0.05**	**86**
4	6	0.033	85
5	8	0.025	83

Reaction conditions: Hydrocinnamic acid (0.2 mmol), TrNSO (0.3 mmol), PC1 (10 mol%), rt, 18 h. Yields calculated by HPLC analysis using 1,3,5-triisopropylbenzene as an internal standard.

Table S3: Acridine catalyst and reaction parameters screening

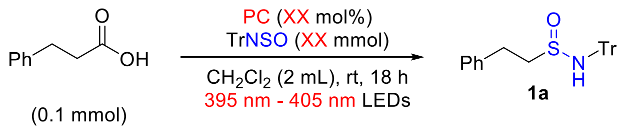

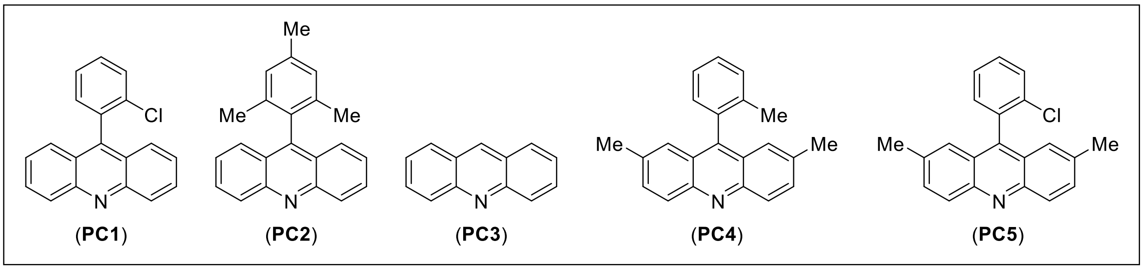

Entry	TrNSO (equiv.)	Catalyst (mol%)	Conversion (%)	Yield of 1a (%)
1	1.5	PC1 (5 mol%)	52	51
2	1.5	PC1 (10 mol%)	100	86 (88)[a]
3	1.5	PC1 (20 mol%)	100	93
4	2.0	PC1 (10 mol%)	100	94
**5**	**1.5**	**PC2 (10 mol%)**	**100**	**98 (96)[a]**
6	1.5	PC3 (10 mol%)	70	50
7	1.5	PC4 (10 mol%)	80	58
8	1.5	PC5 (10 mol%)	100	70
9	1.5	PC1 (10 mol%)	8	7

Reaction conditions: Hydrocinnamic acid (0.2 mmol), TrNSO (0.3 mmol), PC1 (10 mol%), rt, 18 h. Yields calculated by HPLC analysis using 1,3,5-triisopropylbenzene as an internal standard. [a]Isolated yield.

Table 4: Alternative catalyst screening

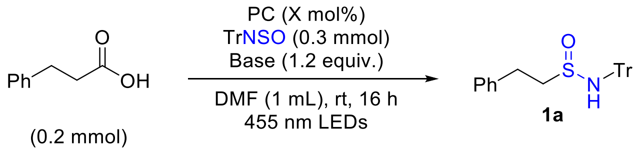

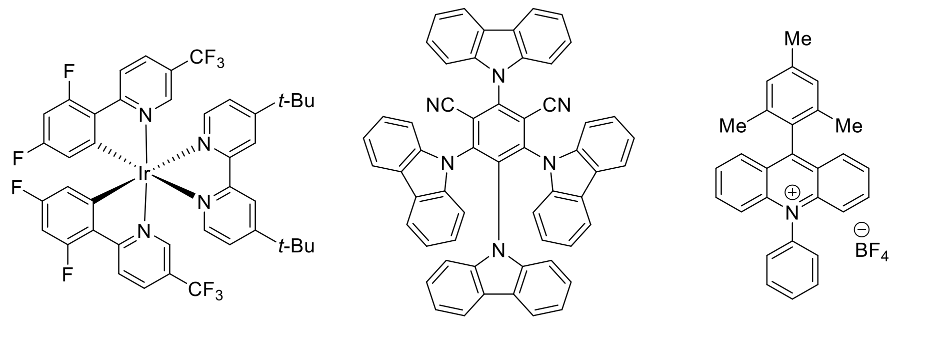

(Ir[dF(CF3)ppy]$_2$(dtbpy))PF$_6$ (**PC6**)   4CzIPN (**PC7**)   9-Mesityl-10-phenylacridinium tetrafluoroborate (**PC8**)

Entry	Catalyst	Base (1.2 equiv.)	Yield of 1a (%)
1	PC6	K_2HPO_4	0
2	PC7	Cs_2CO_3	0
3	PC8	Cs_2CO_3	0

Reaction conditions: Hydrocinnamic acid (0.2 mmol), TrNSO (0.3 mmol), PC1 (10 mol%), DMF (1 mL), rt, 18 h. Yields calculated by HPLC analysis using 1,3,5-triisopropylbenzene as an internal standard.

Table 5: Control Reactions

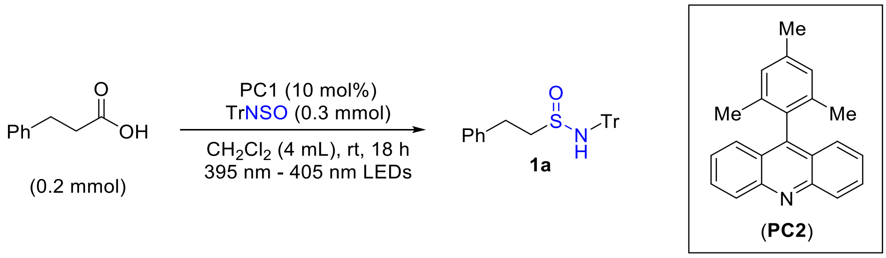

Entry	Modifications	Conversion (%)	Yield of 1a (%)
**1**	**None**	**100**	**98 (96)**
2	No photocatalyst	0	0
3	No light	0	0
4	No light, heat to 40 °C	0	0
5	Dry CH_2Cl_2, solvent sparged with air	41	16
6	"Wet" solvent, sparged with N_2	100	83
7	"Wet" solvent, under air	57	29

Reaction conditions: Hydrocinnamic acid (0.2 mmol), TrNSO (0.3 mmol), PC2 (10 mol%), CH_2Cl_2 (4 mL), rt, 18 h. Yields calculated by HPLC analysis using 1,3,5-triisopropylbenzene as an internal standard. [a]Isolated yield.

3. Experimental Procedures

3.1. Photochemical Reaction Setup:

395 – 405 nm LEDs, where specified, comprised of 10 W LED strips wrapped inside a crystallising dish. 0.20 mmol scale reactions were carried out in 8 mL sample vials, sealed with a screw cap and Parafilm. 3.5 mmol scale reactions were carried out in a round bottom flask. Fan cooling was used during reactions to give an internal temperature of ~22 °C.

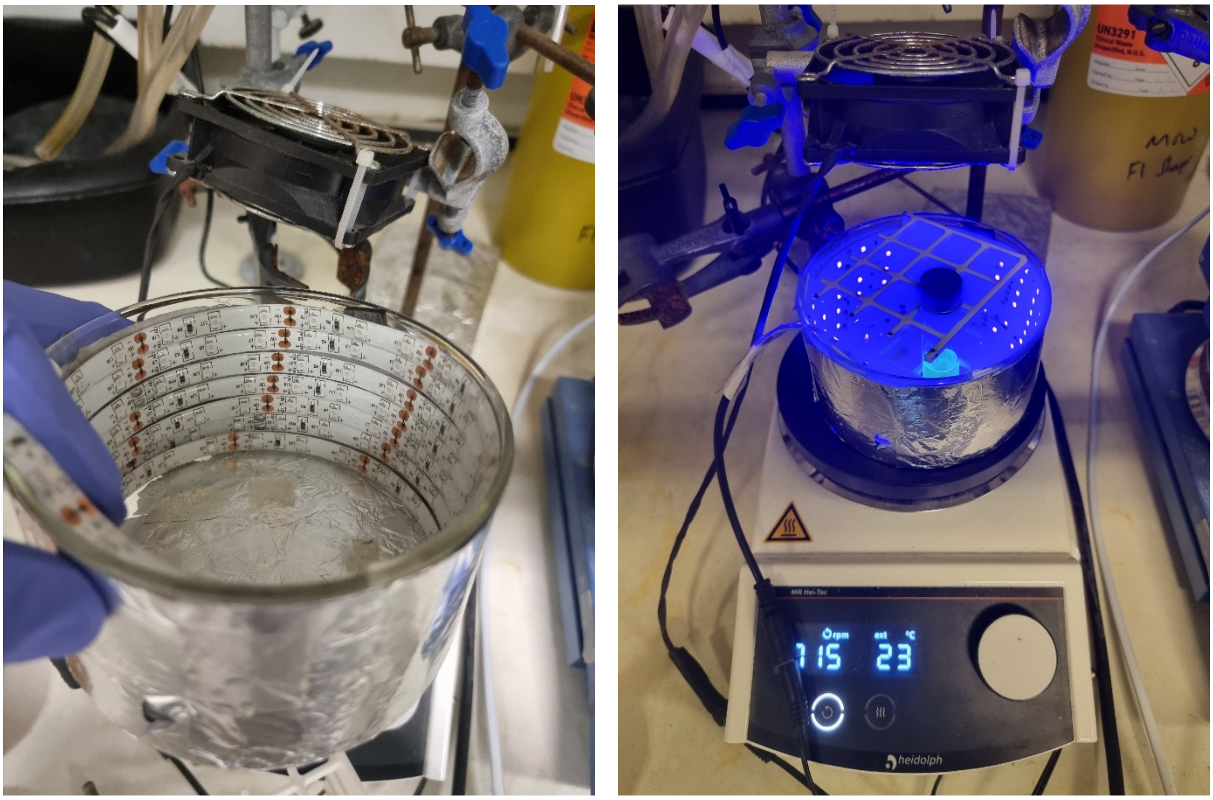

Figure S1. Left: LED strips wrapped inside crystallisation dish. Right: Reaction setup for 0.20 mmol reactions.

3.2. General Procedures

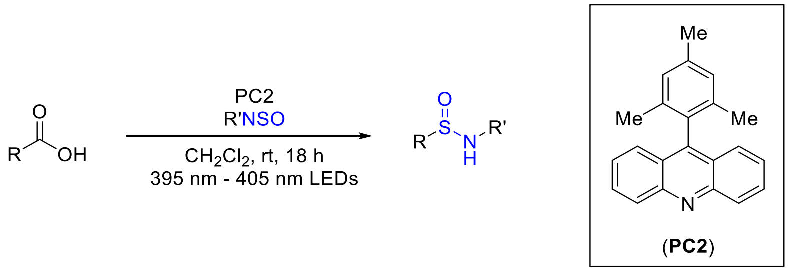

General Procedure A: Carboxylic acid (0.2/0.3 mmol), sulfinylamine/*N*-Sulfinyl-*O*-(*tert*-butyl)hydroxylamine (0.2/0.3 mmol), and photocatalyst PC2 (10/20 mol%) were weighed into a 10 mL vial, followed by dichloromethane (4/2/1 mL). The headspace was flushed with argon and the vial sealed. The reaction was then stirred under 395 – 405 nm LED irradiation (See **Figure S1** for experimental setup) with fan cooling for 18 h. Upon completion (determined by TLC), the reaction mixture was concentrated and purified using silica gel flash column chromatography.

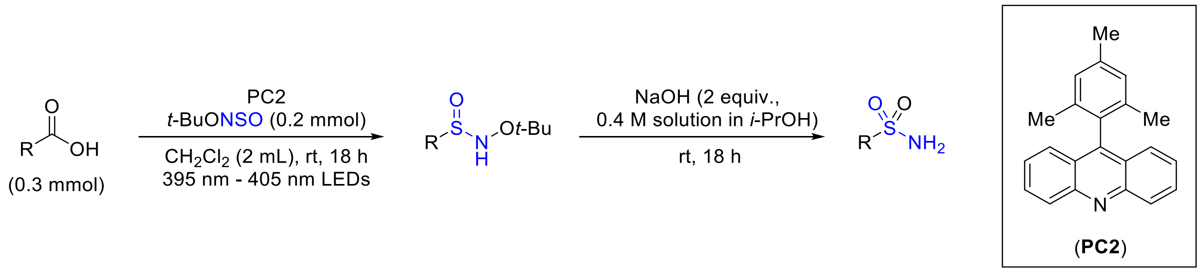

General Procedure B: Carboxylic acid (0.3 mmol), *N*-Sulfinyl-*O*-(*tert*-butyl)hydroxylamine (0.2 mmol), and photocatalyst PC2 (10/20 mol%) were weighed into a 10 mL vial, followed by dichloromethane (2.0 mL). The headspace was flushed with argon and the vial sealed. The reaction was then stirred under 395 – 405 nm LED irradiation (See **Figure S1** for experimental setup) with fan cooling for 18 h. Upon completion (determined by TLC), NaOH (0.4 mmol, 0.4 M solution in *i*-PrOH) was added and the reaction stirred at room temperature for a further 18 h. After completion (determined by TLC) the reaction mixture was washed with water (10 mL), and extracted with dichloromethane (2 × 30 mL). The combined organic layers were washed with brine, dried over Na_2SO_4 and concentrated in vacuo. The crude reaction mixture was purified by silica gel flash column chromatography.

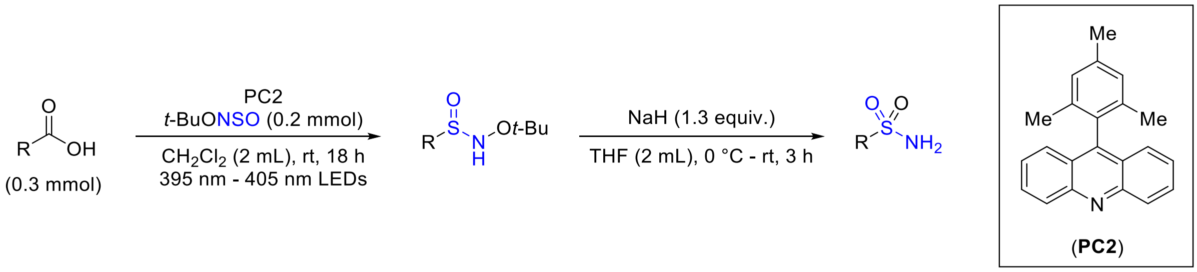

General Procedure C: Carboxylic acid (0.3 mmol), *N*-Sulfinyl-*O*-(*tert*-butyl)hydroxylamine (0.2 mmol), and 9-mesitylacridine (**PC2**) (10 mol%) were weighed into a 10 mL vial followed by dichloromethane (2.0 mL). The headspace was flushed with argon and the vial sealed. The reaction was then stirred under 395 – 405 nm LED irradiation (See **Figure S1** for experimental setup) with fan cooling for 18 h. Upon completion (determined by TLC), the reaction mixture was concentrated in vacuo. The crude material was then dissolved in THF (2.0 mL) was added to it under inert atmosphere and cooled it to 0 °C. NaH (0.26 mmol, 60% dispersion in mineral oil) was added and the reaction was allowed to warm to room temperature and stirred for a further 3 h. After completion (determined by TLC) the reaction mixture was washed with water (10 mL), and extracted with dichloromethane (2 × 30 mL). The combined organic layers were washed with brine, dried over Na_2SO_4 and concentrated in vacuo. The crude reaction mixture was purified by silica gel flash column chromatography.

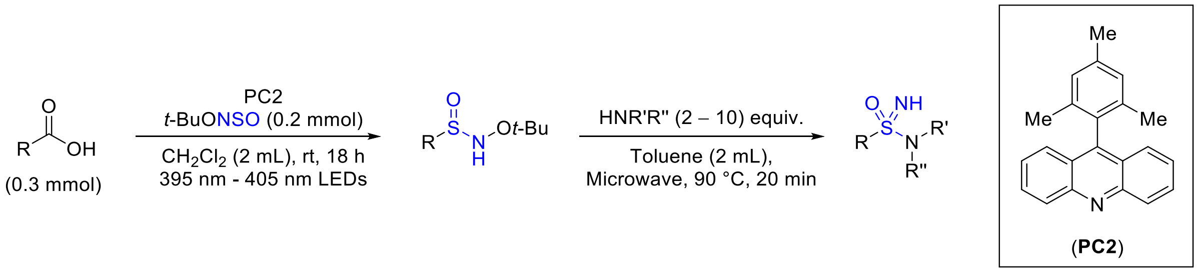

General Procedure D: Carboxylic acid (0.3 mmol), *N*-Sulfinyl-*O*-(*tert*-butyl)hydroxylamine (0.2), and photocatalyst PC2 (10 mol%) were weighed into a 10 mL vial followed by dichloromethane (2.0 mL). The headspace was flushed with argon and the vial sealed. The reaction was then stirred under 395 – 405 nm LED irradiation (See **Figure S1** for experimental setup) with fan cooling for 18 h. Upon completion (As determined by TLC), the reaction solution was transferred to a microwave vial and solvent removed under a stream of nitrogen. The crude material was then dissolved in toluene (2.0 mL), and amine (0.4-2 mmol). The microwave vial was sealed and heated to 90 °C by microwave for 20 mins. Upon completion

(determined by TLC), the reaction mixture was concentrated and purified using silica gel flash column chromatography.

3.3. Sulfinamide Scope

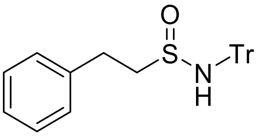

(**1a**)

2-Phenyl-*N*-tritylethane-1-sulfinamide (1a): The title compound was prepared according to **General Procedure A** using hydrocinnamic acid (0.2 mmol, 30.0 mg), *N*-sulfinyltritylamine (0.3 mmol, 91.6 mg), and 9-mesitylacridine (**PC2**) (5.9 mg, 10 mol%) in dichloromethane (4.0 mL). Purification by silica gel flash column chromatography (15 – 30% EtOAc in Petrol) afforded **1a** as a white solid (78.8 mg, 96%).

R_f = 0.4 (30% EtOAc in Petrol); **Melting point** (CH_2Cl_2) = 150 – 152 °C; **IR** (thin film, ν_{max}/cm^{-1}) 3193, 3061, 2963, 1600, 1494, 1448; **^{1}H NMR** (400 MHz, $CDCl_3$) δ 7.30 – 7.18 (m, 17H), 7.19 – 7.14 (m, 1H), 7.14 – 7.09 (m, 2H), 4.80 (s, 1H), 3.82 – 2.46 (m, 4H); **^{13}C NMR** (101 MHz, $CDCl_3$) δ 144.9, 139.2, 129.3, 128.9, 128.7, 128.2, 127.5, 126.8, 73.0, 58.5, 29.3; **LRMS** (ESI$^+$, *m/z*) 434.1 [M+Na]$^+$; **HRMS** (ESI$^+$, *m/z*): Calc. for $C_{27}H_{25}NOSNa$ [M+Na]$^+$ 434.1549, Found: 434.1548.

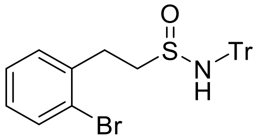

(**1b**)

2-(2-Bromophenyl)-*N*-tritylethane-1-sulfinamide (1b): The title compound was prepared according to **General Procedure A** using 3-(2-bromophenyl)propionic acid (0.2 mmol, 45.8 mg), *N*-sulfinyltritylamine (0.3 mmol, 91.6 mg), and 9-mesitylacridine (**PC2**) (5.9 mg, 10 mol%) in dichloromethane (4.0 mL). Purification by silica gel flash column chromatography (15 – 30% EtOAc in Petrol) afforded **1a** as a white solid (77.2 mg, 79%).

R_f = 0.4 (30% EtOAc in Petrol); **Melting point** (CH_2Cl_2) = 182 – 184 °C; **IR** (thin film, ν_{max}/cm^{-1}) 3192, 3059, 2919, 1597, 1493, 1444; **^{1}H NMR** (400 MHz, $CDCl_3$) δ 7.48 (dt, *J* = 8.0, 0.9 Hz, 1H), 7.32 – 7.19 (m, 15H), 7.19 – 7.14 (m, 2H), 7.08 – 7.01 (m, 1H), 4.88 (s, 1H), 3.11 –

2.87 (m, 4H); **^{13}C NMR** (101 MHz, $CDCl_3$) δ 144.9, 138.5, 133.2, 130.9, 129.3, 128.6, 128.2, 127.9, 127.5, 124.5, 73.1, 56.8, 30.2; **LRMS** (ESI$^+$, *m/z*) 512.0 [M+Na]$^+$ **HRMS** (ESI$^+$, *m/z*): Calc. for $C_{27}H_{24}{}^{79}BrNOSNa$ [M+Na]$^+$ 512.0654 Found: 512.0653.

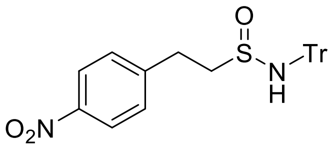

(**1c**)

2-(4-Nitrophenyl)-*N*-tritylethane-1-sulfinamide (1c): The title compound was prepared according to **General Procedure A** using 3-(4-nitrophenyl)propanoic acid (0.2 mmol, 39.0 mg), *N*-sulfinyltritylamine (0.3 mmol, 91.6 mg), and 9-mesitylacridine (**PC2**) (5.9 mg, 10 mol%) in dichloromethane (4.0 mL). Purification by silica gel flash column chromatography (15 – 50% EtOAc in Petrol) afforded **1c** as a white solid (52.1 mg, 57%).

R$_f$ = 0.3 (60% EtOAc in Petrol); **Melting point** (CH_2Cl_2) = 170 – 172 °C; **IR** (thin film, ν_{max}/cm^{-1}) 3180, 3058, 2979, 1600, 1493, 1446; **^{1}H NMR** (400 MHz, $CDCl_3$) δ 8.17 – 7.98 (m, 2H), 7.40 – 7.13 (m, 18H), 4.82 (s, 1H), 3.12 – 2.97 (m, 2H), 3.00 – 2.84 (m, 2H); **^{13}C NMR** (101 MHz, $CDCl_3$) δ 147.0, 146.8, 144.7, 129.6, 129.2, 128.3, 127.7, 124.1, 73.1, 57.8, 29.5; **LRMS** (ESI$^+$, *m/z*) 479.1 [M+Na]$^+$; **HRMS** (ESI$^+$, *m/z*): Calc. for $C_{27}H_{24}N_2O_3SNa$ [M+Na]$^+$ 479.1400, Found: 479.1399.

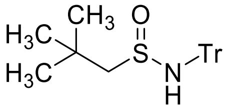

(**1d**)

2,2-Dimethyl-*N*-tritylpropane-1-sulfinamide (1d): The title compound was prepared according to **General Procedure A** using 3,3-dimethylbutanoic acid (0.2 mmol, 23.2 mg), *N*-sulfinyltritylamine (0.3 mmol, 91.6 mg), and 9-mesitylacridine (**PC2**) (5.9 mg, 10 mol%) in dichloromethane (4.0 mL). Purification by silica gel flash column chromatography (15 – 30% EtOAc in Petrol) afforded **1d** as a white solid (28.0 mg, 37%).

R$_f$ = 0.5 (30% EtOAc in Petrol); **Melting point** (CH_2Cl_2) = 140 – 142 °C; **IR** (thin film, ν_{max}/cm^{-1}) 3196, 3060, 2903, 1597, 1493, 1446; **^{1}H NMR** (400 MHz, $CDCl_3$) δ 7.40 – 7.21 (m, 15H), 4.81 (s, 1H), 2.70 (d, *J* = 12.8 Hz, 1H), 2.55 (d, *J* = 12.8 Hz, 1H), 0.98 (s, 9H); **^{13}C NMR** (101

MHz, $CDCl_3$) δ 145.1, 129.2, 128.2, 127.4, 73.1, 72.5, 31.1, 29.8; **LRMS** (ESI^+, *m/z*) 400.2 $[M+Na]^+$; **HRMS** (ESI_+, *m/z*): Calc. for $C_{24}H_{27}NOSNa$ $[M+Na]^+$ 400.1706, Found: 400.1702.

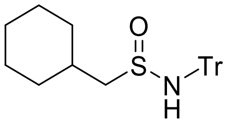

(**1e**)

1-Cyclohexyl-*N*-tritylmethanesulfinamide (1e): The title compound was prepared according to **General Procedure A** using cyclohexaneacetic acid (0.2 mmol, 28.4 mg), *N*-sulfinyltritylamine (0.3 mmol, 91.6 mg), and 9-mesitylacridine (**PC2**) (5.9 mg, 10 mol%) in dichloromethane (4.0 mL). Purification by silica gel flash column chromatography (15 – 30% EtOAc in Petrol) afforded **1e** as a white solid (71.4 mg, 88%).

$\mathbf{R_f}$ = 0.5 (40% EtOAc in Petrol); **Melting point** (CH_2Cl_2) = 172 – 174 °C; **IR** (thin film, ν_{max}/cm^{-1}) 3176, 3059, 2923, 1596, 1490, 1445; **^{1}H NMR** (400 MHz, $CDCl_3$) δ 7.38 – 7.19 (m, 15H), 4.72 (s, 1H), 2.64 (dd, *J* = 12.8, 5.1 Hz, 1H), 2.45 (dd, *J* = 12.7, 8.6 Hz, 1H), 1.82 – 1.55 (m, 6H), 1.29 – 1.03 (m, 3H), 1.03 – 0.81 (m, 2H); **^{13}C NMR** (101 MHz, $CDCl_3$) δ 145.0, 129.2, 128.0, 127.3, 72.8, 65.7, 33.6, 33.1, 32.3, 26.0, 25.9, 25.6; **LRMS** (ESI^+, *m/z*) 426.2 $[M+Na]^+$; **HRMS** (ESI^+, *m/z*): Calc. for $C_{26}H_{29}NOSNa$ $[M+Na]^+$ 426.1862, Found: 426.1862.

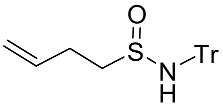

From cyclopropaneacetic acid

(**1f**)

***N*-Tritylbut-3-ene-1-sulfinamide (1f):** The title compound was prepared according to **General Procedure A** using cyclopropaneacetic acid (0.2 mmol, 20.0 mg), *N*-sulfinyltritylamine (0.3 mmol, 91.6 mg), and 9-mesitylacridine (**PC2**) (5.9 mg, 10 mol%) in dichloromethane (4.0 mL). Purification by silica gel flash column chromatography (15 – 30% EtOAc in Petrol) afforded **1f** as a white solid (65.2 mg, 90%).

$\mathbf{R_f}$ = 0.5 (30% EtOAc in Petrol); **Melting point** (CH_2Cl_2) = 110 – 112 °C; **IR** (thin film, ν_{max}/cm^{-1}) 3188, 3061, 2921, 1596, 1493, 1445; **^{1}H NMR** (400 MHz, $CDCl_3$) δ 7.38 – 7.21 (m, 15H), 5.76 (ddt, *J* = 16.8, 10.2, 6.5 Hz, 1H), 5.12 – 4.98 (m, 2H), 4.85 (s, 1H), 2.77 (t, *J* = 7.6 Hz, 2H), 2.50 – 2.32 (m, 2H); **^{13}C NMR** (101 MHz, $CDCl_3$) δ 145.0, 135.4, 129.3, 129.3, 128.2,

127.5, 116.9, 73.1, 56.6, 27.5; **LRMS** (ESI^+, *m/z*) 384.2 $[M+Na]^+$; **HRMS** (ESI^+, *m/z*): Calc. for $C_{23}H_{24}NOS$ $[M+H]^+$ 362.1573, Found: 362.1571.

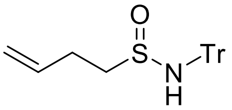

(**1g**)

***N*-Tritylbut-3-ene-1-sulfinamide (1g):** The title compound was prepared according to **General Procedure A** using pent-4-enoic acid (0.2 mmol, 20.0 mg), *N*-sulfinyltritylamine (0.3 mmol, 91.6 mg), and 9-mesitylacridine (**PC2**) (5.9 mg, 10 mol%) in dichloromethane (4.0 mL). Purification by silica gel flash column chromatography (15 – 30% EtOAc in Petrol) afforded **1g** as a white solid (30.1 mg, 42%).

$\mathbf{R_f}$ = 0.5 (30% EtOAc in Petrol); **Melting point** (CH_2Cl_2) = 110 – 112 °C; **IR** (thin film, ν_{max}/cm^{-1}) 3188, 3061, 2921, 1596, 1493, 1445; ^{1}**H NMR** (400 MHz, $CDCl_3$) δ 7.35 – 7.18 (m, 15H), 5.74 (ddt, *J* = 16.8, 10.2, 6.5 Hz, 1H), 5.10 – 4.97 (m, 2H), 4.89 (s, 1H), 2.76 (t, *J* = 7.6 Hz, 2H), 2.50 – 2.31 (m, 2H); ^{13}C NMR (101 MHz, $CDCl_3$) δ 145.0, 135.4, 129.3, 128.2, 127.5, 116.9, 73.1, 56.5, 27.5; **LRMS** (ESI^+, *m/z*) 384.2 $[M+Na]^+$; **HRMS** (ESI^+, *m/z*): Calc. for $C_{23}H_{24}NOS$ $[M+H]^+$ 362.1573, Found: 362.1572.

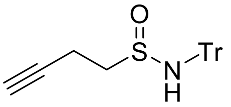

(**1h**)

***N*-Tritylbut-3-yne-1-sulfinamide (1h):** The title compound was prepared according to **General Procedure A** using 4-pentynoic acid (0.2 mmol, 19.6 mg), *N*-sulfinyltritylamine (0.3 mmol, 91.6 mg), and 9-mesitylacridine (**PC2**) (5.9 mg, 10 mol%) in dichloromethane (4.0 mL). Purification by silica gel flash column chromatography (15 – 30% EtOAc in Petrol) afforded **1h** as a white solid (48.1 mg, 67%).

$\mathbf{R_f}$ = 0.5 (30% EtOAc in Petrol); **Melting point** (CH_2Cl_2) = 155 – 157 °C; **IR** (thin film, ν_{max}/cm^{-1}) 3333, 2970, 2931, 2360, 1466, 1410; ^{1}**H NMR** (400 MHz, $CDCl_3$) δ 7.38 – 7.20 (m, 15H), 5.30 (s, 1H), 2.94 – 2.79 (m, 2H), 2.71 (dtd, *J* = 17.0, 7.1, 2.7 Hz, 1H), 2.50 (dtd, *J* = 17.1, 6.9, 2.7 Hz, 1H), 2.00 (t, *J* = 2.7 Hz, 1H); ^{13}**C NMR** (101 MHz, $CDCl_3$) δ 144.9, 129.3, 128.2, 127.6, 81.8, 73.2, 70.6, 54.6, 12.5; **LRMS** (ESI^+, *m/z*) 382.8 $[M+Na]^+$; **HRMS** (ESI^+, *m/z*): Calc. for $C_{23}H_{22}NOS$ $[M+H]^+$ 360.1417, Found: 360.1416.

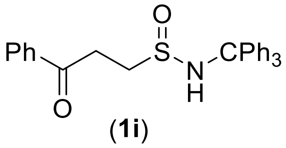

(**1i**)

3-Oxo-3-phenyl-*N*-tritylpropane-1-sulfinamide (1i): The title compound was prepared according to **General Procedure A** using 4-oxo-4-phenylbutanoic acid (0.2 mmol, 35.6 mg), *N*-sulfinyltritylamine (0.3 mmol, 91.6 mg), and 9-mesitylacridine (**PC2**) (5.9 mg, 10 mol%) in dichloromethane (4.0 ml). Purification by silica gel flash column chromatography (15 – 40% EtOAc in Petrol) afforded **1i** as a white solid (50.6 mg, 58%).

R$_f$ = 0.3 (50% EtOAc in Petrol); **Melting point** (CH_2Cl_2) = 120 – 122 °C; **IR** (thin film, ν_{max}/cm^{-1}) 3060, 3026, 2957, 16831597, 1447; **^{1}H NMR** (400 MHz, $CDCl_3$) δ 7.94 – 7.86 (m, 2H), 7.58 – 7.49 (m, 1H), 7.46 – 7.37 (m, 2H), 7.36 – 7.13 (m, 15H), 5.67 (s, 1H), 3.53 – 3.40 (m, 1H), 3.32 – 3.17 (m, 2H), 3.13 – 3.02 (m, 1H); **^{13}C NMR** (101 MHz, $CDCl_3$) δ 198.2, 145.1, 136.3, 133.7, 129.4, 128.8, 128.3, 128.2, 127.5, 73.1, 50.1, 31.6; **HRMS** (ESI$^+$, *m/z*): Calc. for $C_{28}H_{25}NO_2SNa$ [M+Na]$^+$ 462.1498, Found: 462.1497.

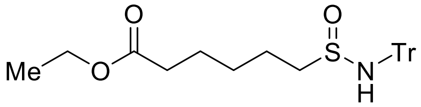

(**1j**)

Ethyl 6-((tritylamino)sulfinyl)hexanoate (1j): The title compound was prepared according to **General Procedure A** using 7-ethoxy-7-oxoheptanoic acid (0.2 mmol, 37.6 mg), *N*-sulfinyltritylamine (0.3 mmol, 91.6 mg), and 9-mesitylacridine (**PC2**) (5.9 mg, 10 mol%) in dichloromethane (4.0 mL). Purification by silica gel flash column chromatography (15 – 50% EtOAc in Petrol) afforded **1j** as a viscous brown oil (73.7 mg, 82%).

R$_f$ = 0.3 (60% EtOAc in Petrol); **IR** (thin film, ν_{max}/cm^{-1}) 3181, 3059, 2979, 1732, 1493, 1446; **^{1}H NMR** (400 MHz, $CDCl_3$) δ 7.35 – 7.17 (m, 15H), 4.83 (s, 1H), 4.07 (q, *J* = 7.1 Hz, 2H), 2.72 – 2.60 (m, 2H), 2.22 (t, *J* = 7.4 Hz, 2H), 1.70 – 1.50 (m, 4H), 1.41 – 1.26 (m, 2H), 1.20 (t, *J* = 7.2 Hz, 3H); **^{13}C NMR** (101 MHz, $CDCl_3$) δ 173.5, 145.0, 129.3, 128.1, 127.5, 73.0, 60.4, 57.5, 34.1, 28.2, 24.6, 23.1, 14.3; **LRMS** (ESI$^+$, *m/z*) 472.2 [M+Na]$^+$; **HRMS** (ESI$^+$, *m/z*): Calc. for $C_{27}H_{31}NO_3SNa$ [M+Na]$^+$ 472.1917, Found: 472.1917.

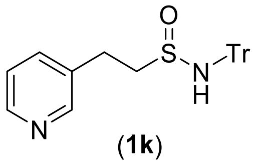

(**1k**)

2-(Pyridin-3-yl)-*N*-tritylethane-1-sulfinamide (1k): The title compound was prepared according to **General Procedure A** by using 3-(pyridin-3-yl)propanoic acid (0.2 mmol, 30.2 mg), *N*-sulfinyltritylamine (0.3 mmol, 91.6 mg), and 9-mesitylacridine (**PC2**) (5.9 mg, 10 mol%) in dichloromethane (4.0 mL). Purification by silica gel flash column chromatography (15 – 50% EtOAc in Petrol) afforded **1k** as a white solid (69.8 mg, 85%).

R_f = 0.3 (60% EtOAc in Petrol); **Melting point** (CH_2Cl_2) = 128 – 130 °C; **IR** (thin film, ν_{max}/cm^{-1}) 3178, 3059, 2927, 1595, 1492, 1445; **^{1}H NMR** (400 MHz, $CDCl_3$) δ 8.49 – 8.24 (m, 2H), 7.37 (dt, *J* = 7.8, 2.0 Hz, 1H), 7.27 – 7.16 (m, 15H), 7.12 (dd, *J* = 7.8, 4.7 Hz, 1H), 4.89 (s, 1H), 2.96 – 2.76 (m, 4H); **^{13}C NMR** (101 MHz, $CDCl_3$) δ 150.0, 148.3, 144.8, 136.1, 134.6, 129.2, 128.2, 127.6, 123.6, 73.1, 58.1, 27.0; **LRMS** (ESI$^+$, *m/z*) 413.2 $[M+H]^+$; **HRMS** (ESI$^+$, *m/z*): Calc. for $C_{26}H_{25}N_2OS$ $[M+H]^+$ 413.1682, Found: 413.1688.

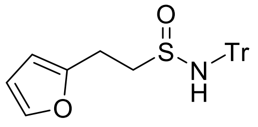

(**1l**)

2-(Furan-2-yl)-*N*-tritylethane-1-sulfinamide (1l): The title compound was prepared according to **General Procedure A** using 3-(furan-2-yl)propanoic acid (0.3 mmol, 42.0 mg), *N*-sulfinyltritylamine (0.2 mmol, 61.1 mg), and 9-mesitylacridine (**PC2**) (5.9 mg, 10 mol%) in dichloromethane (1.0 mL). Purification by silica gel flash column chromatography (15 – 35% EtOAc in Petrol) afforded **1l** as a white solid (40.2 mg, 50%).

R_f = 0.5 (40% EtOAc in Petrol); **Melting point** (CH_2Cl_2) = 148 – 150 °C; **IR** (thin film, ν_{max}/cm^{-1}) 3191, 3058, 2920, 1596, 1493, 1446; **^{1}H NMR** (400 MHz, $CDCl_3$) δ 7.44 – 6.97 (m, 16H), 6.22 (dd, *J* = 3.2, 1.9 Hz, 1H), 6.01 – 5.96 (m, 1H), 4.95 (s, 1H), 3.13 – 2.86 (m, 4H); **^{13}C NMR** (101 MHz, $CDCl_3$) δ 152.6, 144.8, 141.6, 129.2, 128.0, 127.4, 110.4, 106.3, 73.0, 54.9, 21.8; **LRMS** (ESI$^+$, *m/z*) 424.1 $[M+Na]^+$; **HRMS** (ESI$^+$, *m/z*): Calc. for $C_{25}H_{23}NO_2SNa$ $[M+Na]^+$ 424.1342, Found: 424.1343.

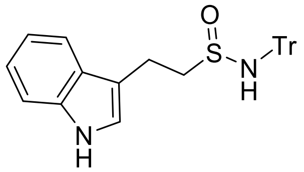

(**1m**)

2-(1*H*-indol-3-yl)-*N*-tritylethane-1-sulfinamide (1m): The title compound was prepared according to **General Procedure A** using 3-(1*H*-indol-3-yl)propanoic acid (0.2 mmol, 40.6 mg), *N*-sulfinyltritylamine (0.3 mmol, 91.6 mg), and 9-mesitylacridine (**PC2**) (5.9 mg, 10 mol%) in dichloromethane (4.0 ml). Purification by silica gel flash column chromatography (15 – 60% EtOAc in Petrol) afforded **1m** as a white solid (47.8 mg, 53%).

R_f = 0.2 (60% EtOAc in Petrol); **Melting point** (CH_2Cl_2) = 165 – 167 °C; **IR** (thin film, ν_{max}/cm^{-1}) 3415, 3217, 3057, 2923, 1493, 1445; **^{1}H NMR** (400 MHz, DMSO-d_6) δ 10.81 (bs, 1H), 7.52 (s, 1H), 7.48 (d, *J* = 7.8 Hz, 1H), 7.39 – 7.21 (m, 16H), 7.12 – 7.02 (m, 2H), 6.96 (ddd, *J* = 8.0, 7.0, 1.1 Hz, 1H), 3.29 – 3.17 (m, 2H), 3.00 – 2.83 (m, 2H); **^{13}C NMR** (101 MHz, DMSO) δ 145.2, 136.2, 129.3, 127.5, 126.9, 126.8, 122.6, 121.0, 118.3, 118.3, 111.9, 111.4, 72.4, 55.4, 19.8; **LRMS** (ESI$^+$, *m/z*) 473.2 $[M+Na]^+$; **HRMS** (ESI$^+$, *m/z*): Calc. for $C_{29}H_{26}N_2OSNa$ $[M+Na]^+$ 473.1658, Found: 473.1657.

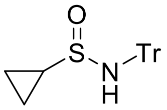

(**1n**)

***N*-Tritylcyclopropanesulfinamide (1n):** The title compound was prepared according to **General Procedure A** using cyclopropanecarboxylic acid (0.2 mmol, 17.2 mg), *N*-sulfinyltritylamine (0.3 mmol, 91.6 mg), and 9-mesitylacridine (**PC2**) (5.9 mg, 10 mol%) in dichloromethane (4.0 mL). Purification by silica gel flash column chromatography (15 – 30% EtOAc in Petrol) afforded **1n** as a white solid (9.0 mg, 13%).

R_f = 0.5 (30% EtOAc in Petrol); **Melting point** (CH_2Cl_2) = 134 – 136 °C; **IR** (thin film, ν_{max}/cm^{-1}) 3212, 3059, 2955, 1597, 1492, 1446; **^{1}H NMR** (400 MHz, $CDCl_3$) δ 7.39 – 7.12 (m, 15H), 4.83 (s, 1H), 2.03 – 1.92 (m, 1H), 1.29 – 1.17 (m, 1H), 0.72 – 0.57 (m, 1H); **^{13}C NMR** (101 MHz, $CDCl_3$) δ 145.2, 129.3, 128.2, 127.5, 72.6, 31.8, 3.9, -1.7; **LRMS** (ESI$^+$, *m/z*) 370.0 $[M+Na]^+$; **HRMS** (ESI$^+$, *m/z*): Calc. for $C_{22}H_{21}NOSNa$ $[M+Na]^+$ 370.1236, Found: 370.1235.

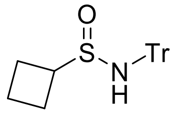

(**1o**)

***N*-Tritylcyclobutanesulfinamide (1o):** The title compound was prepared according to **General Procedure A** using cyclobutanecarboxylic acid (0.2 mmol, 20.0 mg), *N*-sulfinyltritylamine (0.3 mmol, 91.6 mg), and 9-mesitylacridine (**PC2**) (5.9 mg, 10 mol%) in dichloromethane (4.0 mL). Purification by silica gel flash column chromatography (15 – 30% EtOAc in Petrol) afforded **1o** as a white solid (66.0 mg, 91%).

R_f = 0.5 (30% EtOAc in Petrol); **Melting point** (CH_2Cl_2) = 155 – 157 °C; **IR** (thin film, ν_{max}/cm^{-1}) 3185, 3058, 2944, 1597, 1492, 1445; **^{1}H NMR** (400 MHz, $CDCl_3$) δ 7.35 – 7.17 (m, 15H), 4.53 (s, 1H), 3.50 – 3.34 (m, 1H), 2.65 – 2.50 (m, 1H), 2.28 – 2.06 (m, 2H), 2.10 – 1.80 (m, 3H); **^{13}C NMR** (101 MHz, $CDCl_3$) δ 145.1, 129.4, 128.1, 128.1, 127.5, 72.5, 57.1, 23.1, 19.7, 17.5; **LRMS** (ESI$^+$, *m/z*) 384.2 $[M+Na]^+$; **HRMS** (ESI$^+$, *m/z*): Calc. for $C_{23}H_{23}NOSNa$ $[M+Na]^+$ 384.1393, Found: 384.1392.

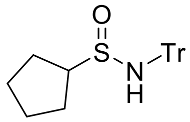

(**1p**)

***N*-Tritylcyclopentanesulfinamide (1p):** The title compound was prepared according to **General Procedure A** using cyclopentanecarboxylic acid (0.2 mmol, 22.8 mg), *N*-sulfinyltritylamine (0.3 mmol, 91.6 mg), and 9-mesitylacridine (**PC2**) (5.9 mg, 10 mol%) in dichloromethane (4.0 mL). Purification by silica gel flash column chromatography (15 – 30% EtOAc in Petrol) afforded **1p** as a white solid (54.9 mg, 73%).

R_f = 0.5 (30% EtOAc in Petrol); **Melting point** (CH_2Cl_2) = 150 – 152 °C; **IR** (thin film, ν_{max}/cm^{-1}) 3185, 3059, 2957, 1596, 1493, 1445; **^{1}H NMR** (400 MHz, $CDCl_3$) δ 7.38 – 7.15 (m, 15H), 4.67 (s, 1H), 3.08 (tt, *J* = 8.7, 6.5 Hz, 1H), 2.10 – 1.96 (m, 1H), 1.95 – 1.78 (m, 2H), 1.75 – 1.42 (m, 5H); **^{13}C NMR** (101 MHz, $CDCl_3$) δ 145.0, 129.5, 128.0, 127.4, 72.9, 65.3, 28.4, 26.0, 25.7, 25.5; **LRMS** (ESI$^+$, *m/z*) 398.1 $[M+Na]^+$; **HRMS** (ESI$^+$, *m/z*): Calc. for $C_{24}H_{25}NOSNa$ $[M+Na]^+$ 398.1549, Found: 398.1548.

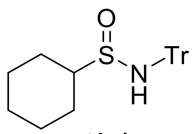

(**1q**)

***N*-Tritylcyclohexanesulfinamide (1q):** The title compound was prepared according to **General Procedure A** using cyclohexanecarboxylic acid (0.2 mmol, 25.6 mg), *N*-sulfinyltritylamine (0.3 mmol, 91.6 mg), and 9-mesitylacridine (**PC2**) (5.9 mg, 10 mol%) in dichloromethane (4.0 mL). Purification by silica gel flash column chromatography (15 – 30% EtOAc in Petrol) afforded **1q** as a white solid (70.7 mg, 91%).

$\mathbf{R_f}$ = 0.5 (30% EtOAc in Petrol); **Melting point** (CH_2Cl_2) = 149 – 151 °C; **IR** (thin film, ν_{max}/cm^{-1}) 3205, 3065, 2980, 1597, 1497, 1447; **^{1}H NMR** (400 MHz, $CDCl_3$) δ 7.42 – 7.08 (m, 15H), 4.66 (s, 1H), 2.44 (tt, *J* = 11.3, 3.7 Hz, 1H), 2.10 – 1.85 (m, 2H), 1.85 – 1.69 (m, 2H), 1.67 – 1.52 (m, 1H), 1.47 – 1.05 (m, 5H); **^{13}C NMR** (101 MHz, $CDCl_3$) δ 145.1, 129.5, 128.1, 127.5, 73.0, 64.4, 26.9, 25.8, 25.7, 25.6, 25.4; **LRMS** (ESI$^+$, *m/z*) 412.2 [M+Na]$^+$; **HRMS** (ESI$^+$, *m/z*): Calc. for $C_7H_{12}O_2Na$ [M+Na]$^+$ 412.1707, Found: 412.1704.

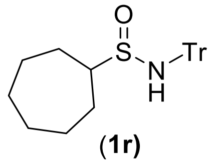

(**1r**)

***N*-Tritylcycloheptanesulfinamide (1r):** The title compound was prepared according to **General Procedure A** using cycloheptanecarboxylic acid (0.2 mmol, 28.4 mg), *N*-sulfinyltritylamine (0.3 mmol, 91.6 mg), and 9-mesitylacridine (**PC2**) (5.9 mg, 10 mol%) in dichloromethane (4.0 mL). Purification by silica gel flash column chromatography (15 – 30% EtOAc in Petrol) afforded **1r** as a white solid (53.4 mg, 66%).

$\mathbf{R_f}$ = 0.5 (30% EtOAc in Petrol); **Melting point** (CH_2Cl_2) = 151 – 153 °C; **IR** (thin film, ν_{max}/cm^{-1}) 3205, 3059, 2924, 1596, 1492, 1445; **^{1}H NMR** (400 MHz, $CDCl_3$) δ 7.52 – 7.00 (m, 15H), 4.63 (s, 1H), 2.53 (tt, *J* = 9.7, 4.3 Hz, 1H), 2.18 – 2.06 (m, 1H), 2.02 – 1.89 (m, 1H), 1.78 – 1.31 (m, 10H); **^{13}C NMR** (101 MHz, $CDCl_3$) δ 145.1, 129.6, 128.1, 127.5, 73.0, 65.5, 28.7, 28.3, 27.5, 26.9, 26.6, 26.3; **LRMS** (ESI$^+$, *m/z*) 426.2 [M+Na]$^+$; **HRMS** (ESI$^+$, *m/z*): Calc. for $C_{26}H_{29}NOSNa$ [M+Na]$^+$ 426.1862, Found: 426.1860.

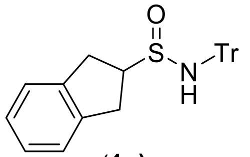

(**1s**)

***N*-Trityl-2,3-dihydro-1*H*-indene-2-sulfinamide (1s):** The title compound was prepared according to **General Procedure A** using 2,3-dihydro-1*H*-indene-2-carboxylic acid (0.2 mmol, 32.4 mg), *N*-sulfinyltritylamine (0.3 mmol, 91.6 mg), and 9-mesitylacridine (**PC2**) (5.9 mg, 10 mol%) in dichloromethane (1.0 mL). Purification by silica gel flash column chromatography (15 – 30% EtOAc in Petrol) afforded **1s** as a white solid (61.6 mg, 73%).

$\mathbf{R_f}$ = 0.5 (30% EtOAc in Petrol); **Melting point** (CH_2Cl_2) = 155 – 157 °C; **IR** (thin film, ν_{max}/cm^{-1}) 3200, 3061, 2968, 1597, 1490, 1445; **^{1}H NMR** (400 MHz, $CDCl_3$) δ 7.42 – 7.31 (m, 9H), 7.30 – 7.21 (m, 10H), 4.87 (s, 1H), 3.94 – 3.61 (m, 1H), 3.47 – 3.17 (m, 4H); **^{13}C NMR** (101 MHz, $CDCl_3$) δ 144.7, 140.9, 140.9, 129.5, 127.9, 127.4, 127.1, 126.9, 124.9, 124.3, 73.0, 63.8, 33.4, 33.1; **LRMS** (ESI^+, *m/z*) 446.0 $[M+Na]^+$; **HRMS** (ESI^+, *m/z*): Calc. for $C_{28}H_{25}NOSNa$ $[M+Na]^+$ 446.1549, Found: 446.1544.

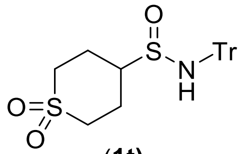

(**1t**)

***N*-Trityltetrahydro-2*H*-thiopyran-4-sulfinamide 1,1-dioxide (1t):** The title compound was prepared according to **General Procedure A** using tetrahydro-2*H*-thiopyran-4-carboxylic acid 1,1-dioxide (0.2 mmol, 35.6 mg), *N*-sulfinyltritylamine (0.3 mmol, 91.6 mg), and 9-mesitylacridine (**PC2**) (5.9 mg, 10 mol%) in dichloromethane (4.0 mL). Purification by silica gel flash column chromatography (15 – 60% EtOAc in Petrol) afforded **1t** as a white solid (82.2 mg, 93%).

$\mathbf{R_f}$ = 0.2 (60% EtOAc in Petrol); **Melting point** (CH_2Cl_2) = 198 – 200 °C; **IR** (thin film, ν_{max}/cm^{-1}) 3185, 3059, 2979, 1596, 1493, 1446; **^{1}H NMR** (400 MHz, $CDCl_3$) δ 7.45 – 7.12 (m, 15H), 4.93 (s, 1H), 3.13 – 3.01 (m, 1H), 3.03 – 2.91 (m, 1H), 2.93 – 2.80 (m, 2H), 2.69 (tt, J = 9.3, 3.7 Hz, 1H), 2.52 – 2.39 (m, 1H), 2.42 – 2.12 (m, 3H); **^{13}C NMR** (101 MHz, $CDCl_3$) δ 144.4, 129.5, 128.3, 127.9, 73.7, 60.7, 49.7, 49.6, 24.7, 24.5; **LRMS** (ESI^+, *m/z*) 462.0 $[M+Na]^+$; **HRMS** (ESI^+, *m/z*): Calc. for $C_{24}H_{25}NO_3S_2Na$ $[M+Na]^+$ 462.1168, Found: 462.1165.

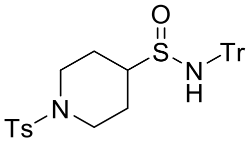

(**1u**)

1-Tosyl-*N*-tritylpiperidine-4-sulfinamide (1u): The title compound was prepared according to **General Procedure A** using 1-tosylpiperidine-4-carboxylic acid (0.2 mmol, 56.7 mg), *N*-sulfinyltritylamine (0.3 mmol, 91.6 mg), and 9-mesitylacridine (**PC2**) (5.9 mg, 10 mol%) in dichloromethane (1.0 mL). Purification by silica gel flash column chromatography (15 – 50% EtOAc in Petrol) afforded **1u** as a white solid (93.8 mg, 86%).

Also prepared on one gram scale according to **General Procedure A** using 1-tosylpiperidine-4-carboxylic acid (3.5 mmol, 1.00 g), *N*-sulfinyltritylamine (5.3 mmol, 1.62 g), and 9-(2-chlorophenyl)acridine (**PC1**) (105 mg, 10 mol%) in dichloromethane (17.5 mL). Purification by silica gel flash column chromatography (15 – 50% EtOAc in Petrol) afforded **1u** as a white solid (1.70 g, 88%).

R_f = 0.31 (50% EtOAc in Petrol); **Melting point** (CH_2Cl_2) = 182 – 184 °C; **IR** (thin film, ν_{max}/cm^{-1}) 3215, 3059, 2923, 1597, 1493, 1446; **^{1}H NMR** (400 MHz, $CDCl_3$) δ 7.79 – 7.43 (m, 2H), 7.29 – 7.13 (m, 17H), 4.69 (s, 1H), 3.68 – 3.52 (m, 2H), 2.49 – 2.26 (m, 6H), 2.06 – 1.91 (m, 2H), 1.77 – 1.55 (m, 2H); **^{13}C NMR** (101 MHz, $CDCl_3$) δ 144.7, 143.9, 133.1, 129.9, 129.5, 128.1, 127.8, 127.7, 73.4, 61.8, 45.4, 45.4, 25.7, 25.5, 21.7; **LRMS** (ESI$^+$, *m/z*) 567.2 $[M+Na]^+$; **HRMS** (ESI$^+$, *m/z*): Calc. for $C_{31}H_{32}N_2O_3S_2Na$ $[M+Na]^+$ 567.1747, Found: 567.1744.

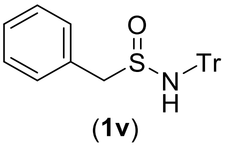

(**1v**)

1-Phenyl-*N*-tritylmethanesulfinamide (1v): The title compound was prepared according to **General Procedure A** using phenylacetic acid (0.3 mmol, 40.8 mg), *N*-sulfinyltritylamine (0.2 mmol, 61.1 mg), and 9-mesitylacridine (**PC2**) (5.9 mg, 10 mol%) in dichloromethane (1.0 mL). Purification by silica gel flash column chromatography (15 – 30% EtOAc in Petrol) afforded **1v** as a white solid (27.8 mg, 35%).

R_f = 0.5 (30% EtOAc in Petrol); **Melting point** (CH_2Cl_2) = 170 – 172 °C; **IR** (thin film, ν_{max}/cm^{-1}) 3183, 3059, 2922, 1600, 1493, 1445; **^{1}H NMR** (400 MHz, $CDCl_3$) δ 7.38 – 7.29 (m, 3H),

7.29 – 7.11 (m, 17H), 4.64 (s, 1H), 3.90 (d, *J* = 12.6 Hz, 1H), 3.76 (d, *J* = 12.7 Hz, 1H); **^{13}C NMR** (101 MHz, $CDCl_3$) δ 145.0, 131.0, 129.3, 129.2, 129.2, 128.9, 128.5, 128.1, 127.4, 72.8, 62.4; **LRMS** (ESI$^+$, *m/z*) 420.0 [M+Na]$^+$; **HRMS** (ESI$^+$, *m/z*): Calc. for $C_{26}H_{23}NOSNa$ [M+Na]$^+$ 420.1393, Found: 420.1392.

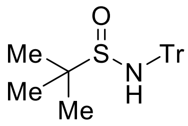

(**1w**)

2-Methyl-*N*-tritylpropane-2-sulfinamide (1w): The title compound was prepared according to **General Procedure A** using pivalic acid (0.3 mmol, 30.6 mg), *N*-sulfinyltritylamine (0.2 mmol, 60.0 mg), and 9-mesitylacridine (**PC2**) (5.9 mg, 10 mol%) in dichloromethane (1.0 mL). Purification by silica gel flash column chromatography (15 – 30% EtOAc in Petrol) afforded **1w** as a white solid (30.8 mg, 42%).

R_f = 0.5 (30% EtOAc in Petrol); **Melting point** (CH_2Cl_2) = 150 – 152 °C; **IR** (thin film, ν_{max}/cm^{-1}) 3086, 3059, 2958, 1597, 1492, 1445; **^{1}H NMR** (400 MHz, $CDCl_3$) δ 7.39 – 7.07 (m, 15H), 4.46 (s, 1H), 1.20 (s, 9H); **^{13}C NMR** (101 MHz, $CDCl_3$) δ 145.1, 129.7, 128.0, 127.5, 73.0, 57.3, 23.2; **LRMS** (ESI$^+$, *m/z*) 386.0 [M+Na]$^+$; **HRMS** (ESI$^+$, *m/z*): Calc. for $C_{23}H_{26}NOS$ [M+H]$^+$ 364.1730, Found: 364.1725.

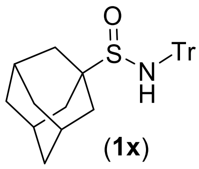

(**1x**)

***N*-Trityladamantane-1-sulfinamide (1x):** The title compound was prepared according to **General Procedure A** using adamantanecarboxylic acid (0.3 mmol, 54.1 mg), *N*-sulfinyltritylamine (0.2 mmol, 61.1 mg), and 9-mesitylacridine (**PC2**) (5.9 mg, 10 mol%) in dichloromethane (1.0 mL). Purification by silica gel flash column chromatography (15 – 30% EtOAc in Petrol) afforded **1x** as a white solid (75.0 mg, 85%).

R_f = 0.5 (40% EtOAc in Petrol); **Melting point** (CH_2Cl_2) = 208 – 210 °C; **IR** (thin film, ν_{max}/cm^{-1}) 3059, 2980, 2851 1601, 1492, 1447; **^{1}H NMR** (400 MHz, $CDCl_3$) δ 7.62 – 7.02 (m, 15H), 4.63 (s, 1H), 2.24 – 2.13 (m, 3H), 2.00 – 1.90 (m, 3H), 1.90 – 1.82 (m, 3H), 1.82 – 1.67 (m, 7H); **^{13}C NMR** (101 MHz, $CDCl_3$) δ 145.2, 129.7, 128.0, 127.4, 72.8, 59.1, 36.6, 35.4, 28.9;

LRMS (ESI$^+$, *m/z*) 442.1 [M+H]$^+$; **HRMS** (ESI$^+$, *m/z*): Calc. for $C_{29}H_{32}NOS$ [M+H]$^+$ 442.2199, Found: 442.2197.

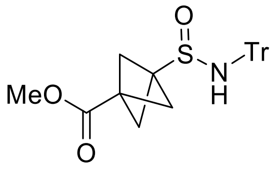

(**1y**)

Methyl 3-((tritylamino)sulfinyl)bicyclo[1.1.1]pentane-1-carboxylate (1y): The title compound was prepared according to **General Procedure A** using 3-(methoxycarbonyl)bicyclo[1.1.1]pentane-1-carboxylic acid (0.3 mmol, 51.0 mg), *N*-sulfinyltritylamine (0.2 mmol, 61.1 mg), and 9-mesitylacridine (**PC2**) (5.9 mg, 10 mol%) in dichloromethane (1.0 mL). Purification by silica gel flash column chromatography (30 – 40% EtOAc in Petrol) afforded **1y** as a colourless gum (78.5 mg, 91%).

$\mathbf{R_f}$ = 0.38 (40% EtOAc in Petrol); **IR** (thin film, ν_{max}/cm^{-1}) 3191, 3061, 1735, 1446, 1324, 1209, 702; ^{1}**H NMR** (400 MHz, $CDCl_3$) δ 7.35 – 7.26 (m, 15H), 4.66 (s, 1H), 3.71 (s, 3H), 2.31 (s, 6H); ^{13}**C NMR** (101 MHz, $CDCl_3$) δ 169.1, 144.9, 129.3, 128.2, 127.6, 72.8, 52.6, 52.3, 50.5, 37.2.; **LRMS** (ESI$^+$, *m/z*) 454.2 [M+Na]$^+$; **HRMS** (ESI$^+$, *m/z*): Calc. for $C_{26}H_{25}NO_3S$ [M+H]$^+$ 432.1628, Found: 432.1631.

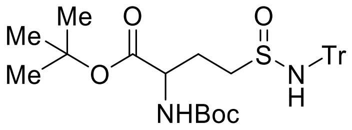

(**1z**)

***tert*-Butyl 2-((*tert*-butoxycarbonyl)amino)-4-((tritylamino)sulfinyl)butanoate (1z):** The title compound was prepared according to **General Procedure A** using Boc-DL-Glu-O*t*Bu (0.2 mmol, 60.7 mg), *N*-sulfinyltritylamine (0.3 mmol, 91.6 mg), and 9-mesitylacridine (**PC2**) (5.9 mg, 10 mol%) in dichloromethane (4.0 mL). Purification by silica gel flash column chromatography (15 – 40% EtOAc in Petrol) afforded **1z** as a pale-brown oil (85.0 mg, 75%, 1:1 inseparable mixture of diastereomers).

$\mathbf{R_f}$ = 0.4 (50% EtOAc in Petrol); **IR** (thin film, ν_{max}/cm^{-1}) 3168, 3087, 2943, 2866, 1604, 1462; ^{1}**H NMR** (400 MHz, Benzene-d_6) δ 7.43 (d, J = 7.6 Hz, 6H), 7.12 (t, J = 7.7 Hz, 6H), 7.07 – 7.00 (m, 3H), 5.51 – 5.40 (m, 0.5H), 5.36 – 5.27 (m, 0.5H), 4.48 – 4.36 (m, 1H), 2.62 (t, J =

7.4 Hz, 1H), 2.59 – 2.46 (m, 1H), 2.20 – 2.09 (m, 0.5H), 2.08 – 1.95 (m, 0.5H), 1.90 – 1.71 (m, 1H), 1.46 – 1.33 (m, 9H), 1.31 – 1.18 (m, 9H); **^{13}C NMR** (151 MHz, Acetone-d_6) δ 171.91, 171.86, 156.5, 156.4, 146.3, 130.5, 128.5, 127.94, 127.92, 81.74, 81.71, 79.3, 73.97, 73.95, 54.5, 54.4, 53.5, 53.4, 30.4, 28.6, 28.2, 28.1, 27.4, 27.2 (*3 aromatic and 2 aliphatic signals are unresolved from the corresponding signals in the other diastereomer*); **LRMS** (ESI$^+$, *m/z*) 587.1 [M+Na]$^+$; **HRMS** (ESI$^+$, *m/z*): Calc. for $C_{32}H_{40}N_2O_5SNa$ [M+Na]$^+$ 587.2550, Found: 587.2548.

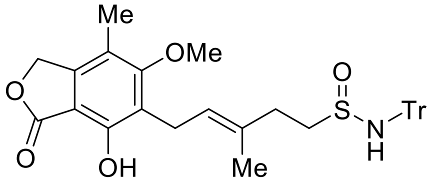

(1aa)

(*E*)-5-(4-Hydroxy-6-methoxy-7-methyl-3-oxo-1,3-dihydroisobenzofuran-5-yl)-3-methyl-*N*-tritylpent-3-ene-1-sulfinamide (1aa): The title compound was prepared according to the General Procedure A by using mycophenolic acid (0.3 mmol, 96.1 mg), *N*-sulfinyltritylamine (0.2 mmol, 92.0 mg), and 9-mesitylacridine (**PC2**) (12.0 mg, 20 mol%) in dichloromethane (4.0 mL). Purification by silica gel flash column chromatography (15 – 50% EtOAc in Petrol) afforded **1aa** as a white solid (69.8 mg, 60%).

R_f = 0.3 (60% EtOAc in Petrol); **Melting point** (CH_2Cl_2) = 114 – 116 °C; **IR** (thin film, ν_{max}/cm^{-1}) 3248, 3059, 2979, 1622, 1491, 1448; **^{1}H NMR** (400 MHz, $CDCl_3$) δ 7.61 (s, 1H), 7.32 – 7.15 (m, 16H), 5.24 (tq, *J* = 7.1, 1.4 Hz, 1H), 5.16 (s, 2H), 4.85 (s, 1H), 3.67 (s, 3H), 3.32 (d, *J* = 6.9 Hz, 2H), 2.79 – 2.72 (m, 2H), 2.30 (tdd, *J* = 14.8, 12.1, 7.5 Hz, 2H), 2.10 (s, 3H), 1.74 (d, *J* = 1.4 Hz, 3H); **^{13}C NMR** (101 MHz, $CDCl_3$) δ 172.89, 163.67, 153.61, 144.85, 144.09, 132.88, 129.19, 128.01, 127.4, 124.1, 121.8, 116.7, 106.4, 72.9, 70.0, 61.0, 55.7, 32.7, 22.6, 16.2, 11.6; **LRMS** (ESI$^+$, *m/z*) 604.2 [M+Na]$^+$; **HRMS** (ESI$^+$, *m/z*): Calc. for $C_{35}H_{35}NO_5SNa$ [M+Na]$^+$ 604.2128, Found: 604.2128.

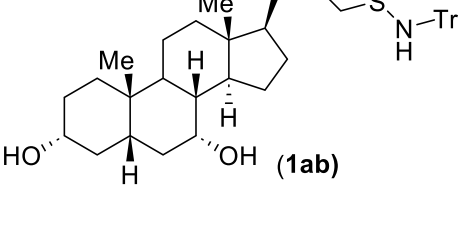

(1ab)

(3*R*)-3-((3*R*,7*R*,8*R*,9*S*,10*S*,13*R*,14*S*,17*R*)-3,7-Dihydroxy-10,13-dimethylhexadecahydro-1*H*-cyclopenta[*a*]phenanthren-17-yl)-*N*-tritylbutane-1-sulfinamide (1ab): The title compound was prepared according to **General Procedure A** using chenodeoxycholic acid (0.2 mmol, 78.5 mg), *N*-sulfinyltritylamine (0.3 mmol, 91.6 mg), and 9-mesitylacridine (**PC2**) (5.9 mg, 10 mol%) in dichloromethane (4.0 mL). Purification by silica gel flash column chromatography (15 – 80% EtOAc in Petrol) afforded **1ab** as a white solid (110 mg, 84%, 1:1 inseparable mixture of diastereomers).

R_f = 0.2 (80% EtOAc in Petrol); **Melting point** (CH_2Cl_2) = 120 – 122 °C; **IR** (thin film, ν_{max}/cm^{-1}) 3395, 3059, 2928, 1597, 1492, 1446; **^{1}H NMR** (600 MHz, $CDCl_3$) δ 7.36 – 7.24 (m, 15H), 4.95 (s, 0.5H), 4.93 (s, 0.5H), 3.81 (s, 1H), 3.49 – 3.36 (m, 1H), 2.81 – 2.69 (m, 1H), 2.68 – 2.54 (m, 1H), 2.20 (q, *J* = 12.2 Hz, 1H), 1.98 – 1.90 (m, 2H), 1.89 – 1.73 (m, 4H), 1.73 – 1.55 (m, 4H), 1.52 – 1.42 (m, 4H), 1.42 – 1.02 (m, 10H), 1.00 – 0.86 (m, 7H), 0.62 (s, 3H).; **^{13}C NMR** (151 MHz, $CDCl_3$) δ 145.0, 144.9, 129.34, 129.28, 128.09, 128.06, 127.43, 127.40, 72.94, 72.92, 72.0, 68.4, 55.9, 55.8, 55.0, 54.9, 50.5, 42.8, 41.6, 39.9, 39.7, 39.5, 35.4, 35.2, 35.12, 35.08, 34.81, 34.76, 32.9, 30.8, 29.5, 29.2, 28.3, 28.2, 23.7, 22.9, 20.7, 18.64, 18.55, 18.5, 11.89, 11.87 *(13 carbon signals are unresolved from the corresponding signals in the other diastereomer);* **HRMS** (ESI$^+$, *m/z*): Calc. for $C_{42}H_{55}NO_3SNa$ [M+Na]$^+$ 676.3795, Found: 676.3795.

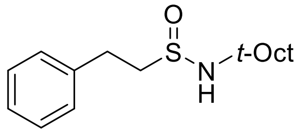

(1ac)

2-Phenyl-*N*-(2,4,4-trimethylpentan-2-yl)ethane-1-sulfinamide (1ac): The title compound was prepared according to **General Procedure A** using hydrocinnamic acid (0.2 mmol, 30.0 mg), *N*-sulfinyl-*tert*-octylamine (0.3 mmol, 53.0 mg), and 9-mesitylacridine (**PC2**) (5.9 mg, 10 mol%) in dichloromethane (4.0 mL). Purification by silica gel flash column chromatography (15 – 30% EtOAc in Petrol) afforded **1ac** as a colourless oil (42.3 mg, 75%).

R_f = 0.5 (30% EtOAc in Petrol); **IR** (thin film, ν_{max}/cm^{-1}) 3175, 2951, 2868, 1604, 1496, 1455; **^{1}H NMR** (400 MHz, $CDCl_3$) δ 7.34 – 7.28 (m, 2H), 7.26 – 7.19 (m, 3H), 3.60 (s, 1H), 3.06 – 2.88 (m, 4H), 1.67 – 1.47 (m, 2H), 1.38 (d, *J* = 2.6 Hz, 6H), 1.00 (s, 9H); **^{13}C NMR** (101 MHz, $CDCl_3$) δ 139.3, 128.8, 128.7, 126.7, 58.5, 58.1, 56.2, 32.4, 31.9, 31.9, 29.6, 29.5; **LRMS**

(ESI$^+$, *m/z*) 304.2 [M+Na]$^+$; **HRMS** (ESI$^+$, *m/z*): Calc. for $C_{16}H_{27}NOSNa$ [M+Na]$^+$ 304.1706, Found: 304.1707;

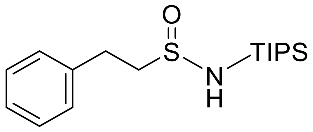

(**1ad**)

2-Phenyl-*N*-(triisopropylsilyl)ethane-1-sulfinamide (1ad): The title compound was prepared according to **General Procedure A** using hydrocinnamic acid (0.3 mmol, 38 mg), *N*-sulfinyl-triisopropylsilylamine (0.2 mmol, 44.0 mg), and 9-mesitylacridine (**PC2**) (5.9 mg, 10 mol%) in dichloromethane (4.0 mL). Purification by silica gel flash column chromatography (15 – 30% EtOAc in Petrol) afforded **1ad** as a colourless oil (51.0 mg, 78%).

R_f = 0.5 (30% EtOAc in Petrol); **IR** (thin film, ν_{max}/cm^{-1}) 3168, 3029, 2943, 1604, 1497, 1462; **^{1}H NMR** (400 MHz, $CDCl_3$) δ 7.35 – 7.27 (m, 2H), 7.27 – 7.19 (m, 3H), 3.48 (bs, 1H), 3.14 – 2.93 (m, 4H), 1.21 – 1.09 (m, 3H), 1.06 (m, 18H); **^{13}C NMR** (101 MHz, $CDCl_3$) δ 139.4, 128.9, 128.6, 126.7, 61.9, 29.4, 18.0, 17.9, 11.8; **LRMS** (ESI$^+$, *m/z*) 326.2 [M+H]$^+$; **HRMS** (ESI$^+$, *m/z*): Calc. for $C_{17}H_{32}NOSSi$ [M+H]$^+$ 326.1968, Found: 326.1967;

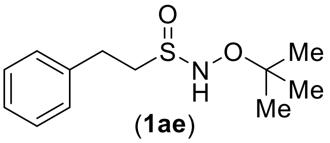

(**1ae**)

***N*-(*tert*-Butoxy)-2-phenylethane-1-sulfinamide (1ae):** The title compound was prepared according to **General Procedure A** using hydrocinnamic acid (0.3 mmol, 54.0 mg), *N*-sulfinyl-*O*-(*tert*-butyl)hydroxylamine (0.2 mmol, 27.0 mg), 9-mesitylacridine (**PC2**) (5.9 mg, 10 mol%) in dichloromethane (2.0 mL). Purification by silica gel flash column chromatography (15 – 25% EtOAc in Petrol) afforded **1ae** as a white solid (41.0 mg, 85%).

R_f = 0.5 (30% EtOAc in Petrol); **Melting point** (CH_2Cl_2) = 116 – 118 °C; **IR** (thin film, ν_{max}/cm^{-1}) 3347, 2971, 2929, 2884, 1649, 1464; **^{1}H NMR** (400 MHz, $CDCl_3$) δ 7.33 – 7.24 (m, 2H), 7.24 – 7.16 (m, 3H), 6.92 (bs, 1H), 3.09 – 2.93 (m, 4H), 1.17 (s, 9H); **^{13}C NMR** (101 MHz, $CDCl_3$) δ 138.9, 128.9, 128.6, 126.9, 80.4, 52.6, 29.0, 26.7; **LRMS** (ESI$^+$, *m/z*) 264.0 [M+Na]$^+$ **HRMS** (ESI$^+$, *m/z*): Calc. for $C_{12}H_{19}NO_2SNa$ [M+Na]$^+$ 264.1029, Found: 264.1028.

Single Crystal Data for **1ae**: C12H19NO2S, Mr =241.35. 150 K – monoclinic, $P2_1/n$, a = 13.12190(10) Å, b = 5.80900(10) Å, c = 17.4736(2) Å, β = 94.2238(10)°, V = 1328.31(3) $Å^3$, Data/restraints/parameters – 2745/0/146, Rint = 0.021, Final R1 = 0.0266, wR2 = 0.0737 (I>2σ(I)).

Crystal Structure for **1ae**:

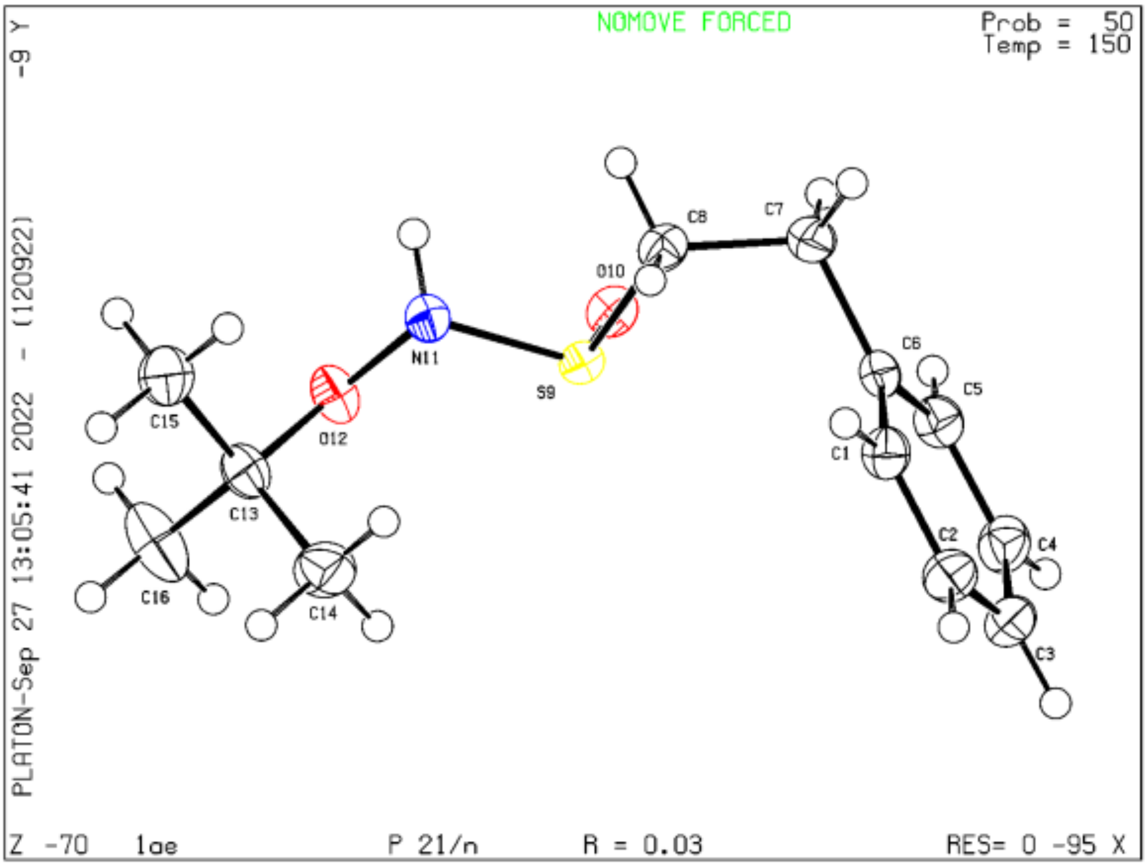

3.4. Sulfonamide Scope

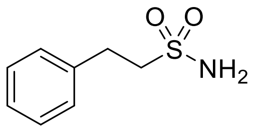

(**2a**)

2-Phenylethane-1-sulfonamide (2a): The title compound was prepared according to **General Procedure B** using hydrocinnamic acid (0.3 mmol, 45.0 mg), *N*-Sulfinyl-*O*-(*tert*-butyl)hydroxylamine (0.2 mmol, 27.0 mg), and 9-mesitylacridine (**PC2**) (5.9 mg, 10 mol%) in dichloromethane (2.0 mL). NaOH (1.0 mL of 0.4 M solution of NaOH in *i*-PrOH) was used in the second step. Purification by silica gel flash column chromatography (15 – 30% EtOAc in Petrol) afforded **2a** as a white solid (25.3 mg, 68%).

R_f = 0.4 (40% EtOAc in Petrol); **Melting point** (CH_2Cl_2) = 106 – 108 °C; **IR** (thin film, ν_{max}/cm^{-1}) 3355, 3250, 2922, 2851, 1548, 1452; **^{1}H NMR** (400 MHz, $CDCl_3$) δ 7.45 – 7.30 (m, 2H), 7.30 – 7.18 (m, 3H), 4.53 (s, 2H), 3.46 – 3.37 (m, 2H), 3.22 – 3.12 (m, 2H); **^{13}C NMR** (101 MHz, $CDCl_3$) δ 137.8, 129.1, 128.6, 127.3, 56.7, 30.4; **LRMS** (ESI$^+$, *m/z*) 186.0 $[M+H]^+$; **HRMS** (ESI$^-$, *m/z*): Calc. for $C_8H_{10}NO_2S$ $[M-H]^-$ 184.0438, Found: 184.0430.

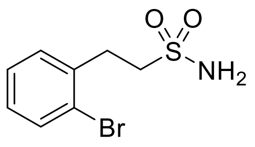

(2b)

2-(2-Bromophenyl)ethane-1-sulfonamide (2b): The title compound was prepared according to **General Procedure B** using 3-(2-bromophenyl)propionic acid (0.3 mmol, 68.7 mg), *N*-Sulfinyl-*O*-(*tert*-butyl)hydroxylamine (0.2 mmol, 27.0 mg), and 9-mesitylacridine (**PC2**) (5.9 mg, 10 mol%) in dichloromethane (2.0 mL). NaOH (1.0 mL of 0.4 M solution of NaOH in *i*-PrOH) was used in the second step. Purification by silica gel flash column chromatography (15 – 40% EtOAc in Petrol) afforded **2b** as a white solid (43.0 mg, 81%).

R_f = 0.3 (40% EtOAc in Petrol); **Melting point** (CH_2Cl_2) = 102 – 104 °C; **IR** (thin film, ν_{max}/cm^{-1}) 3355, 2971, 2931, 2883, 1646, 1465; **^{1}H NMR** (400 MHz, DMSO-*d*$_6$) δ 7.61 (dd, *J* = 8.0, 1.3 Hz, 0H), 7.42 (s, 0H), 7.35 (td, *J* = 7.4, 1.3 Hz, 0H), 7.20 (td, *J* = 7.8, 1.8 Hz, 0H), 6.96 (s, 1H), 3.27 – 3.16 (m, 1H), 3.18 – 3.07 (m, 1H); **^{13}C NMR** (101 MHz, DMSO-*d*$_6$) δ 137.8, 132.7,

130.9, 128.8, 128.1, 123.6, 53.9, 30.2; **LRMS** (ESI$^+$, *m/z*) 285.8 [M+Na]$^+$; **HRMS** (ESI$^+$, *m/z*): Calc. for $C_8H_{10}{}^{79}BrNO_2SNa$ [M+Na]$^+$ 285.9508, Found: 285.9509.

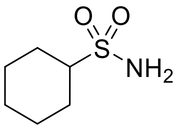

(**2c**)

Cyclohexanesulfonamide (2c): The title compound was prepared according to **General Procedure B** using cyclohexanecarboxylic acid (0.3 mmol, 38.4 mg), *N*-Sulfinyl-*O*-(*tert*-butyl)hydroxylamine (0.2 mmol, 27.0 mg), and 9-mesitylacridine (**PC2**) (5.9 mg, 10 mol%) in dichloromethane (2.0 mL). NaOH (1.0 mL of 0.4 M solution of NaOH in *i*-PrOH) was used in the second step. Purification by silica gel flash column chromatography (15 – 40% EtOAc in Petrol) afforded **2c** as a white solid (30.0 mg, 92%).

R$_f$ = 0.27 (40% EtOAc in Petrol); **Melting point** (CH_2Cl_2) = 82 – 84 °C; **IR** (thin film, ν_{max}/cm^{-1}) 3350, 2970, 2934, 2865, 1646, 1460; **^{1}H NMR** (400 MHz, $CDCl_3$) δ 4.73 (s, 2H), 2.91 (tt, *J* = 12.1, 3.4 Hz, 1H), 2.28 – 2.17 (m, 2H), 1.96 – 1.85 (m, 2H), 1.78 – 1.66 (m, 1H), 1.49 (qd, *J* = 12.4, 3.3 Hz, 2H), 1.37 – 1.10 (m, 3H); **^{13}C NMR** (101 MHz, $CDCl_3$) δ 62.9, 26.7, 25.2 (2C); **LRMS** (ESI$^+$, *m/z*) 327.1 [2M+H]$^+$; **HRMS** (ESI$^-$, *m/z*): Calc. for $C_6H_{12}NO_2S$ [M-H]$^-$ 162.0594, Found: 162.0587.

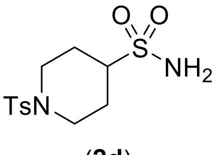

(**2d**)

1-Tosylpiperidine-4-sulfonamide (2d): The title compound was prepared according to **General Procedure B** using 1-tosylpiperidine-4-carboxylic acid (0.3 mmol, 85.0 mg), *N*-Sulfinyl-*O*-(*tert*-butyl)hydroxylamine (0.2 mmol, 27.0 mg), and 9-mesitylacridine (**PC2**) (5.9 mg, 10 mol%) in dichloromethane (2.0 mL). NaOH (1.0 mL of 0.4 M solution of NaOH in *i*-PrOH) was used in the second step. Purification by silica gel flash column chromatography (15 – 50% EtOAc in Petrol) afforded **2d** as a white solid (52.2 mg, 82%).

R$_f$ = 0.4 (60% EtOAc in Petrol); **Melting point** (CH_2Cl_2) = 178 – 180 °C; **IR** (thin film, ν_{max}/cm^{-1}) 3350, 3266, 3099, 2979, 1597, 1557, 1451; **^{1}H NMR** (400 MHz, Acetone-*d*$_6$) δ 7.71 – 7.63 (m, 2H), 7.49 – 7.41 (m, 2H), 6.10 (s, 2H), 3.86 (ddq, *J* = 11.8, 4.5, 2.0 Hz, 2H), 2.94 (tt, *J* =

11.9, 3.8 Hz, 1H), 2.44 (s, 3H), 2.40 (td, J = 12.2, 2.7 Hz, 2H), 2.25 – 2.15 (m, 2H), 1.79 (dtd, J = 13.2, 12.0, 4.3 Hz, 2H); **^{13}C NMR** (101 MHz, Acetone-d_6) δ 144.6, 134.8, 130.7, 128.7, 59.8, 46.0, 27.0, 21.5; **LRMS** (ESI^+, *m/z*) 319.0 $[M+H]^+$; **HRMS** (ESI^+, *m/z*): Calc. for $C_{12}H_{19}N_2O_4S_2$ $[M+H]^+$ 319.0781, Found: 319.0781.

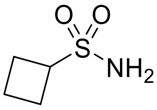

(**2e**)

Cyclobutanesulfonamide (2e): The title compound was prepared according to **General Procedure B** using cyclobutanecarboxylic acid (0.3 mmol, 30.0 mg), *N*-Sulfinyl-*O*-(*tert*-butyl)hydroxylamine (0.2 mmol, 27.0 mg), and 9-mesitylacridine (**PC2**) (12.0 mg, 20 mol%) in dichloromethane (2.0 mL). NaOH (1.0 mL of 0.4 M solution of NaOH in *i*-PrOH) was used in the second step. Purification by silica gel flash column chromatography (15 – 40% EtOAc in Petrol) afforded **2e** as a white solid (25.2 mg, 93%).

R_f = 0.16 (40% EtOAc in Petrol); **Melting point** (CH_2Cl_2) = 78 – 80 °C; **IR** (thin film, ν_{max}/cm^{-1}) 3344, 3256, 3106, 2953,1633, 1559, 1436; **^{1}H NMR** (400 MHz, $CDCl_3$) δ 4.75 (s, 2H), 3.93 – 3.80 (m, 1H), 2.56 – 2.42 (m, 2H), 2.42 – 2.29 (m, 2H), 2.09 – 1.94 (m, 2H); **^{13}C NMR** (101 MHz, $CDCl_3$) δ 56.5, 24.1, 16.6; **LRMS** (ESI^+, *m/z*) 271.1 $[2M+H]^+$; **HRMS** (ESI^+, *m/z*): Calc. for $C_4H_{10}NO_2S$ $[M+H]^+$ 136.0427, Found: 136.0427.

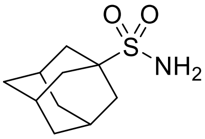

(**2f**)

Adamantane-1-sulfonamide (2f): The title compound was prepared according to **General Procedure B** using adamantane-1-carboxylic acid (0.3 mmol, 54.1 mg), *N*-Sulfinyl-*O*-(*tert*-butyl)hydroxylamine (0.2 mmol, 27.0 mg), and 9-mesitylacridine (**PC2**) (5.9 mg, 10 mol%) in dichloromethane (2.0 mL). NaOH (1.0 mL of 0.4 M solution of NaOH in *i*-PrOH) was used in the second step. Purification by silica gel flash column chromatography (15 –40% EtOAc in Petrol) afforded **2f** as a white solid (37.8 mg, 88%).

R_f = 0.3 (40% EtOAc in Petrol); **Melting point** (CH_2Cl_2) = 176 – 178 °C; **IR** (thin film, ν_{max}/cm^{-1}) 3293, 3250, 3061, 2936, 2907, 1577, 1451; **^{1}H NMR** (400 MHz, $CDCl_3$) δ 4.47 (s, 2H), 2.17

(app. p, J = 3.2 Hz, 3H), 2.04 (d, J = 3.0 Hz, 6H), 1.79 – 1.65 (m, 6H); **^{13}C NMR** (101 MHz, $CDCl_3$) δ 60.2, 35.9, 35.8, 28.2; **LRMS** (ESI$^+$, *m/z*) 238.0 [2M+Na]$^+$; **HRMS** (ESI$^+$, *m/z*): Calc. for $C_{10}H_{17}NO_2SNa$ [M+Na]$^+$ 238.0872, Found: 238.0874.

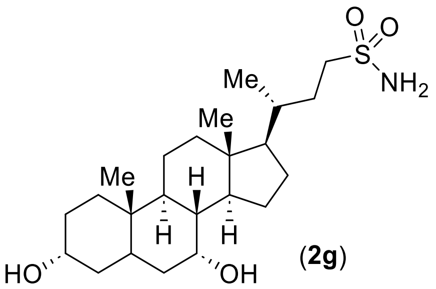

(**2g**)

(3*R*)-3-((3*R*,7*R*,8*R*,9*S*,10*S*,13*R*,14*S*,17*R*)-3,7-Dihydroxy-10,13-dimethylhexadecahydro-1*H*-cyclopenta[*a*]phenanthren-17-yl)butane-1-sulfonamide (2g): The title compound was prepared according to **General Procedure B** using chenodeoxycholic acid (0.3 mmol, 118 mg), *N*-Sulfinyl-*O*-(*tert*-butyl)hydroxylamine (0.2 mmol, 27.0 mg), and 9-mesitylacridine (**PC2**) (5.9 mg, 10 mol%) in dichloromethane (2.0 mL). NaOH (2.0 mL of 0.4 M solution of NaOH in *i*-PrOH) was used in the second step. Purification by silica gel flash column chromatography (15 – 100% EtOAc in Petrol) afforded **2g** as a white solid (45.0 mg, 53%).

R_f = 0.3 (100% EtOAc); **Melting point** (CH_2Cl_2) = 215 – 217 °C; **IR** (thin film, ν_{max}/cm^{-1}) 3352, 3097, 2929, 1567, 1465, 1448; **^{1}H NMR** (600 MHz, DMSO-d_6) δ 6.69 (s, 2H), 4.30 (d, J = 4.6 Hz, 1H), 4.10 (d, J = 3.5 Hz, 1H), 3.63 (p, J = 3.2 Hz, 1H), 3.18 (ddt, J = 11.2, 7.2, 4.1 Hz, 1H), 2.97 (ddd, J = 13.5, 11.8, 4.4 Hz, 1H), 2.85 (ddd, J = 13.6, 11.4, 4.8 Hz, 1H), 2.19 (td, J = 13.4, 11.3 Hz, 1H), 1.90 (dt, J = 12.3, 3.5 Hz, 1H), 1.84 – 1.72 (m, 4H), 1.68 (tdd, J = 12.2, 6.6, 3.1 Hz, 2H), 1.54 – 1.32 (m, 8H), 1.28 – 1.05 (m, 6H), 1.00 (qd, J = 12.0, 6.3 Hz, 1H), 0.90 (d, J = 6.4 Hz, 3H), 0.88 – 0.84 (m, 1H), 0.84 (s, 3H), 0.62 (s, 3H); **^{13}C NMR** (151 MHz, DMSO-d_6) δ 70.3, 66.1, 55.2, 51.7, 50.0, 41.9, 41.4, 40.1, 39.6, 39.3, 35.3, 34.8, 34.7, 34.2, 32.3, 30.5, 29.5, 27.7, 23.1, 22.7, 20.2, 18.3, 11.7; **LRMS** (ESI$^+$, *m/z*) 450.2 [M+Na]$^+$; **HRMS** (ESI$^+$, *m/z*): Calc. for $C_{23}H_{41}NO_4SNa$ [M+Na]$^+$ 450.2649, Found: 450.2649; **$[\alpha]_D^{25}$** (c = 1, methanol) = +5.1.

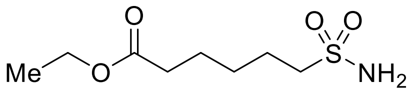

(**2h**)

Ethyl 6-sulfamoylhexanoate (2h): The title compound was prepared according to **General Procedure C** using 7-ethoxy-7-oxoheptanoic acid (0.3 mmol, 56.5 mg), *N*-Sulfinyl-*O*-(*tert*-butyl)hydroxylamine (0.2 mmol, 27.0 mg), and 9-mesitylacridine (**PC2**) (5.9 mg, 10 mol%) in dichloromethane (2.0 mL). In the second step THF (2.0 mL), NaH (60% in mineral oil) (0.26 mmol, 10.4 mg) were used. Purification by silica gel flash column chromatography (15 – 50% EtOAc in Petrol) afforded **2h** as a colourless oil (26.0 mg, 58%).

R_f = 0.4 (50% EtOAc in Petrol); **IR** (thin film, ν_{max}/cm^{-1}) 3347, 3264, 2941, 1720, 1562, 1460; **^{1}H NMR** (400 MHz, $CDCl_3$) δ 5.01 (s, 2H), 4.11 (q, *J* = 7.2 Hz, 2H), 3.17 – 3.07 (m, 2H), 2.31 (t, *J* = 7.3 Hz, 2H), 1.92 – 1.80 (m, 3H), 1.72 – 1.60 (m, 2H), 1.54 – 1.41 (m, 2H), 1.24 (t, *J* = 7.1 Hz, 3H); **^{13}C NMR** (101 MHz, $CDCl_3$) δ 173.6, 60.6, 55.1, 34.0, 27.7, 24.4, 23.8, 14.3; **LRMS** (ESI$^+$, *m/z*) 246.0 [M+Na]$^+$; **HRMS** (ESI$^+$, *m/z*): Calc. for $C_8H_{17}NO_4SNa$ [M+Na]$^+$ 246.0770, Found: 246.0770.

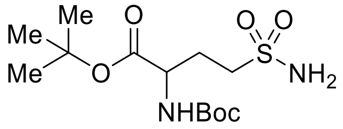

(**2i**)

***tert*-Butyl 2-((*tert*-butoxycarbonyl)amino)-4-sulfamoylbutanoate (2i):** The title compound was prepared according to General Procedure C using Boc-DL-Glu-O*t*Bu (0.3 mmol, 91.0 mg), *N*-Sulfinyl-*O*-(*tert*-butyl)hydroxylamine (0.2 mmol, 27.0 mg), and 9-mesitylacridine (**PC2**) (5.9 mg, 10 mol%) in dichloromethane (2.0 mL). In the second step THF (2.0 mL), NaH (60% in mineral oil) (0.26 mmol, 10.4 mg) were used. Purification by silica gel flash column chromatography (15 – 40% EtOAc in Petrol) afforded **2i** as a colourless oil (47.2 mg, 70%).

R_f = 0.4 (40% EtOAc in Petrol); **IR** (thin film, ν_{max}/cm^{-1}) 3344, 2976, 1699, 1519, 1455; **^{1}H NMR** (400 MHz, $CDCl_3$) δ 5.32 (d, *J* = 7.8 Hz, 1H), 5.23 (s, 2H), 4.38 – 3.99 (m, 1H), 3.20 (qdd, *J* = 14.1, 10.0, 5.6 Hz, 2H), 2.35 (ddt, *J* = 13.8, 10.6, 5.5 Hz, 1H), 2.13 (dddd, *J* = 13.7, 10.0, 7.8, 5.6 Hz, 1H), 1.46 (s, 9H), 1.42 (s, 9H); **^{13}C NMR** (101 MHz, $CDCl_3$) δ 170.7, 155.7, 83.1, 80.4, 52.6, 51.6, 28.4, 28.1, 27.7; **LRMS** (ESI$^+$, *m/z*) 361.1 [M+H]$^+$; **HRMS** (ESI$^+$, *m/z*): Calc. for $C_{13}H_{26}N_2O_6SNa$ [M+Na]$^+$ 361.1404, Found: 361.1402.

3.5. Sulfonimidamide Scope

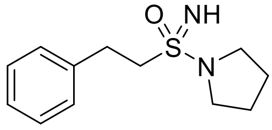

(**3a**)

1-(2-Phenylethylsulfonimidoyl)pyrrolidine (3a): The title compound was prepared according to **General Procedure D** using hydrocinnamic acid (0.3 mmol, 45.1 mg), *N*-sulfinyl-*O*-(*tert*-butyl)hydroxylamine (0.2 mmol, 27.0 mg) and 9-mesitylacridine (**PC2**) (5.9 mg, 10 mol%) in dichloromethane (2.0 mL). Pyrrolidine (0.4 mmol, 32.8 µL) in toluene (2.0 mL) was used in the second step. Purification by silica gel flash column chromatography (90 – 100% EtOAc in Petrol) afforded **3a** as a colourless oil (34.2 mg, 72%).

R_f = 0.27 (90% EtOAc in Petrol); **IR** (thin film, ν_{max}/cm^{-1}) 3273, 2973, 2875, 1497, 1455, 1240; **^{1}H NMR** (400 MHz, $CDCl_3$) δ 7.34 – 7.27 (m, 2H), 7.25 – 7.19 (m, 3H), 3.37 – 3.26 (m, 5H), 3.22 – 3.03 (m, 3H), 2.26 (br. s, 1H), 1.97 – 1.81 (m, 4H); **^{13}C NMR** (101 MHz, $CDCl_3$) δ 138.7, 128.9, 128.6, 126.9, 50.8, 48.5, 30.1, 26.1; **LRMS** (ESI$^+$, *m/z*) 239.0 [M+H]$^+$, 261.0 [M+Na]$^+$; **HRMS** (ESI$^+$, *m/z*): Calc. for $C_{12}H_{19}N_2OS$ [M+H]$^+$ 239.1213, Found: 239.1213.

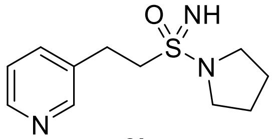

(**3b**)

3-(2-(Pyrrolidine-1-sulfonimidoyl)ethyl)pyridine (3b): The title compound was prepared according to **General Procedure D** using 3-pyridinepropanoic acid (0.3 mmol, 45.3 mg), *N*-sulfinyl-*O*-(*tert*-butyl)hydroxylamine (0.2 mmol, 27.0 mg) and 9-mesitylacridine (**PC2**) (5.9 mg, 10 mol%) in dichloromethane (2.0 mL). Pyrrolidine (0.4 mmol, 32.8 µL) in toluene (2.0 mL) was used in the second step. Purification by silica gel flash column chromatography (5-10% MeOH in EtOAc) afforded **3b** as a pale-yellow oil (16.1 mg, 34%).

R_f = 0.14 (10% MeOH in EtOAc); **IR** (thin film, ν_{max}/cm^{-1}) 3276, 2955, 2878, 1480, 1426, 1239; **^{1}H NMR** (400 MHz, $CDCl_3$) δ 8.57 – 8.44 (m, 2H), 7.63 – 7.53 (m, 1H), 7.26 – 7.21 (m, 1H), 3.37 – 3.24 (m, 5H), 3.25 – 3.16 (m, 2H), 3.15 – 3.05 (m, 1H), 2.32 (br. s, 1H), 1.96 – 1.81 (m, 4H); **^{13}C NMR** (101 MHz, $CDCl_3$) δ 149.9, 148.4, 136.2, 134.3, 123.7, 50.2, 48.4,

27.5, 26.1; **LRMS** (ESI$^+$, *m/z*) 240.1 [M+H]$^+$; **HRMS** (ESI$^+$, *m/z*): Calc. for $C_{11}H_{18}N_3OS$ [M+H]$^+$ 240.1166, Found: 240.1165.

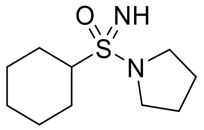

(**3c**)

1-(Cyclohexanesulfonimidoyl)pyrrolidine (3c): The title compound was prepared according to **General Procedure D** using cyclohexanecarboxylic acid (0.3 mmol, 38.5 mg), *N*-sulfinyl-*O*-(*tert*-butyl)hydroxylamine (0.2 mmol, 27.0 mg) and 9-mesitylacridine (**PC2**) (5.9 mg, 10 mol%) in dichloromethane (2.0 mL). Pyrrolidine (0.4 mmol, 32.8 μL) in toluene (2.0 mL) was used in the second step. Purification by silica gel flash column chromatography (100% EtOAc) afforded **3c** as a colourless oil (33.7 mg, 78%).

$\mathbf{R_f}$ = 0.27 (100% EtOAc); **IR** (thin film, ν_{max}/cm^{-1}) 3262, 2934, 2857, 1452, 1236, 1009; **^{1}H NMR** (400 MHz, $CDCl_3$) δ 3.55 – 3.27 (m, 5H), 2.29 (br. d, *J* = 12.2 Hz, 1H), 2.11 (br. d, *J* = 12.2 Hz, 1H), 2.03 – 1.81 (m, 6H), 1.76 – 1.53 (m, 3H), 1.43 – 1.10 (m, 4H); **^{13}C NMR** (101 MHz, $CDCl_3$) δ 61.8, 48.5, 27.4, 26.6, 26.1, 25.53, 25.51, 25.3; **LRMS** (ESI$^+$, *m/z*) 217.0 [M+H]$^+$; **HRMS** (ESI$^+$, *m/z*): Calc. for $C_{10}H_{21}N_2OS$ [M+H]$^+$ 217.1369, Found: 217.1370.

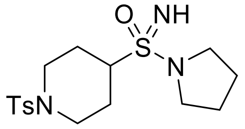

(**3d**)

4-(Pyrrolidine-1-sulfonimidoyl)-1-tosylpiperidine (3d): The title compound was prepared according to **General Procedure D** using 1-tosylpiperidine-4-carboxylic acid (0.3 mmol, 85.0 mg), *N*-sulfinyl-*O*-(*tert*-butyl)hydroxylamine (0.2 mmol, 27.0 mg) and 9-mesitylacridine (**PC2**) (5.9 mg, 10 mol%) in dichloromethane (2.0 mL). Pyrrolidine (0.4 mmol, 32.8 μL) in toluene (2.0 mL) was used in the second step. Purification by silica gel flash column chromatography (100% EtOAc) afforded **3d** as a pale yellow solid (60.0 mg, 81%).

$\mathbf{R_f}$ = 0.17 (100% EtOAc); **Melting point** (CH_2Cl_2) = 124-126 °C **IR** (thin film, ν_{max}/cm^{-1}) 3299, 2935, 2849, 1333, 1243, 1163; **^{1}H NMR** (400 MHz, $CDCl_3$) δ 7.65 – 7.58 (m, 2H), 7.31 (d, *J* = 7.9 Hz, 2H), 3.95 – 3.84 (m, 2H), 3.30 (tt, *J* = 6.7, 3.4 Hz, 4H), 2.91 (tt, *J* = 12.0, 3.7 Hz, 1H), 2.42 (s, 3H), 2.35 (br. s, 1H), 2.27 (td, *J* = 12.2, 1.9 Hz, 2H), 2.22 – 2.08 (m, 2H), 2.00 –

1.80 (m, 6H); **^{13}C NMR** (101 MHz, $CDCl_3$) δ 143.9, 133.2, 129.9, 127.7, 59.5, 48.8, 45.4, 45.4, 26.3, 26.2, 26.1, 21.6; **LRMS** (ESI^+, *m/z*) 372.1 $[M+H]^+$; **HRMS** (ESI^+, *m/z*): Calc. for $C_{16}H_{26}N_3O_3S_2$ $[M+H]^+$372.1410, Found: 372.1410.

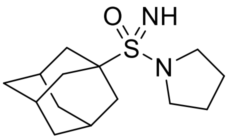

(**3e**)

1-((3*s*,5*s*,7*s*)-Adamantane-1-sulfonimidoyl)pyrrolidine (3e): The title compound was prepared according to **General Procedure D** using 1-adamantanecarboxylic acid (0.3 mmol, 54.1 mg), *N*-sulfinyl-*O*-(*tert*-butyl)hydroxylamine (0.2 mmol, 27.0 mg) and 9-mesitylacridine (**PC2**) (5.9 mg, 10 mol%) in dichloromethane (2.0 mL). Pyrrolidine (0.4 mmol, 32.8 µL) in toluene (2.0 mL) was used in the second step. Purification by silica gel flash column chromatography (70 – 80% EtOAc in Petrol) afforded **3e** as a white solid (42.1 mg, 79%).

R_f = 0.27 (70% EtOAc in Petrol); **Melting point** (CH_2Cl_2) = 82 – 84 °C; **IR** (thin film, ν_{max}/cm^{-1}) 3275, 2908. 2851, 1454, 1231, 999; **^{1}H NMR** (400 MHz, $CDCl_3$) δ 3.42 (tq, *J* = 7.4, 3.3 Hz, 4H), 2.56 (br. s, 1H), 2.13 (s, 3H), 2.08 (app. d, *J* = 2.8 Hz, 6H), 1.94 – 1.83 (m, 4H), 1.69 (s, 6H); **^{13}C NMR** (101 MHz, $CDCl_3$) δ 63.7, 49.9, 36.7, 36.1, 28.7, 26.3; **HRMS** (ESI^+, *m/z*): Calc. for $C_{14}H_{25}N_2OS$ $[M+H]^+$ 269.1682, Found: 269.1684.

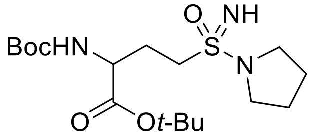

(**3f**)

***tert*-Butyl 2-((*tert*-butoxycarbonyl)amino)-4-(pyrrolidine-1-sulfonimidoyl)butanoate (3f)**: The title compound was prepared according to **General Procedure D** using Boc-DL-Glu-O*t*Bu (0.3 mmol, 54.1 mg), *N*-sulfinyl-*O*-(*tert*-butyl)hydroxylamine (0.2 mmol, 27.0 mg) and 9-mesitylacridine (**PC2**) (5.9 mg, 10 mol%) in dichloromethane (2.0 mL). Pyrrolidine (0.4 mmol, 32.8 µL) in toluene (2.0 mL) was used in the second step. Purification by silica gel flash column chromatography (70 – 80% EtOAc in Petrol) afforded **3f** as a colourless oil (50.8 mg, 65%, 1:1 inseparable mixture of diastereomers).

R_f = 0.25 (80% EtOAc in Petrol); **IR** (thin film, ν_{max}/cm^{-1}) 3310, 2976, 2933, 1710, 1518, 1367; **^{1}H NMR** (600 MHz, Acetone-d_6, 1:1 mix of diastereomers) δ 4.22 – 4.02 (m, 1H), 3.35 – 3.23

(m, 4H), 3.14 (ddd, J = 13.6, 10.4, 5.5 Hz, 0.5H), 3.12 – 2.99 (m, 1H), 2.96 (ddd, J = 14.1, 10.5, 5.0 Hz, 0.5H), 2.37 – 2.25 (m, 1H), 2.20 – 2.09 (m, 1H), 2.09 – 2.06 (m, 2H), 1.95 – 1.82 (m, 4H), 1.46 (s, 9H), 1.41 (s, 9H); **^{13}C NMR** (151 MHz, Acetone-*d*$_6$, 1:1 mix of diastereomers) δ 171.8, 156.5, 81.8, 79.3, 54.24, 54.21, 48.9, 46.3, 46.2, 28.6, 28.2, 27.12, 27.09, 26.5 (8 carbon signals are unresolved from the corresponding signals in the other diastereomer); **LRMS** (ESI$^+$, *m/z*) 392.2 [M+Na]$^+$; **HRMS** (ESI$^+$, *m/z*): Calc. for $C_{17}H_{33}N_3O_5SNa$ [M+Na]$^+$ 392.2214, Found: 392.2212.

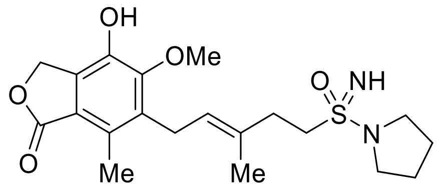

(**3g**)

(*E*)-4-Hydroxy-5-methoxy-7-methyl-6-(3-methyl-5-(pyrrolidine-1-sulfonimidoyl)pent-2-en-1-yl)isobenzofuran-1(3*H*)-one (3g): The title compound was prepared according to **General Procedure D** using mycophenolic acid (0.3 mmol, 96.1 mg), *N*-sulfinyl-*O*-(*tert*-butyl)hydroxylamine (0.2 mmol, 27.0 mg) and 9-mesitylacridine (**PC2**) (5.9 mg, 10 mol%) in dichloromethane (2.0 mL). Pyrrolidine (0.4 mmol, 32.8 μL) in toluene (2.0 mL) was used in the second step. Purification by silica gel flash column chromatography (0 – 10% MeOH in EtOAc) afforded **3g** as a colourless gum (42.6 mg, 52%).

R$_f$ = 0.18 (100% EtOAc in Petrol); **IR** (thin film, ν_{max}/cm^{-1}) 3423, 2943, 1734, 1620, 1456, 1099; **^{1}H NMR** (400 MHz, $CDCl_3$) δ 5.33 – 5.25 (m, 1H), 5.18 (s, 2H), 3.75 (s, 3H), 3.37 (d, *J* = 7.0 Hz, 2H), 3.34 – 3.23 (m, 4H), 3.09 (ddd, *J* = 13.2, 11.3, 5.7 Hz, 1H), 2.95 (ddd, *J* = 13.3, 11.0, 5.7 Hz, 1H), 2.49 (tq, *J* = 14.3, 7.1 Hz, 2H), 2.13 (s, 3H), 1.94 – 1.85 (m, 4H), 1.81 (s, 3H); **^{13}C NMR** (101 MHz, $CDCl_3$) δ 173.0, 163.8, 153.7, 144.3, 132.6, 124.0, 121.9, 116.9, 106.5, 70.2, 61.1, 48.4, 48.3, 33.4, 26.0, 22.7, 16.4, 11.7; **LRMS** (ESI$^+$, *m/z*) 409.1 [M+H]$^+$; **HRMS** (ESI$^+$, *m/z*): Calc. for $C_{20}H_{29}N_2O_5S$ [M+H]$^+$ 409.1792, Found: 409.1790.

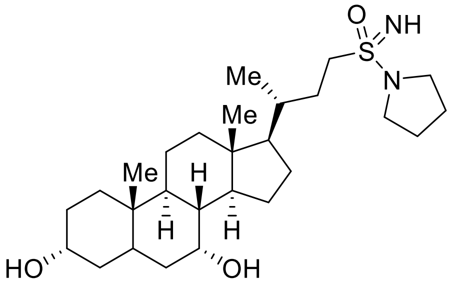

(**3h**)

(3*R*,7*R*,8*R*,9*S*,10*S*,13*R*,14*S*,17*R*)-10,13-dimethyl-17-((2*R*)-4-(pyrrolidine-1-sulfonimidoyl)butan-2-yl)hexadecahydro-1*H*-cyclopenta[*a*]phenanthrene-3,7-diol (3h): The title compound was prepared according to **General Procedure D** using chenodeoxycholic acid (0.3 mmol, 118 mg), *N*-sulfinyl-*O*-(*tert*-butyl)hydroxylamine (0.2 mmol, 27.0 mg) and 9-mesitylacridine (**PC2**) (5.9 mg, 10 mol%) in dichloromethane (2.0 mL). Pyrrolidine (0.4 mmol, 32.8 μL) in toluene (2.0 mL) was used in the second step. Purification by silica gel flash column chromatography (5 – 10% MeOH in EtOAc) afforded **3h** as a colourless gum (28.0 mg, 29%, 1:1 inseparable mixture of diastereomers).

R_f = 0.20 (5% MeOH in EtOAc); **IR** (thin film, ν_{max}/cm^{-1}) 3365, 2930, 2866, 1449, 1239, 1110; **^{1}H NMR** (600 MHz, $CDCl_3$, 1:1 mix of diastereomers) δ 3.89 – 3.80 (m, 1H), 3.48 – 3.42 (m, 1H), 3.39 – 3.28 (m, 4H), 3.13 (td, *J* = 12.8, 4.7 Hz, 0.5H), 3.05 – 2.94 (m, 1H), 2.88 – 2.80 (m, 0.5H), 2.20 (q, *J* = 13.0 Hz, 1H), 2.05 – 1.87 (m, 9H), 1.86 – 1.79 (m, 2H), 1.75 – 1.22 (m, 15H), 1.21 – 1.09 (m, 3H), 1.01 – 0.92 (m, 4H), 0.90 (s, 3H), 0.66 (s, 3H); **^{13}C NMR** (151 MHz, $CDCl_3$, 1:1 mix of diastereomers) δ 72.1, 68.6, 55.7, 55.60, 50.59, 50.57, 48.49, 48.48, 47.0, 42.9, 41.6, 40.0, 39.7, 39.6, 39.54, 35.46, 35.19, 35.18, 35.1, 34.83, 34.81, 33.0, 30.8, 29.5, 29.4, 28.31, 28.29, 26.1, 23.8, 22.9, 20.7, 18.7, 18.6, 11.9 (16 carbon signals are unresolved from the corresponding signals in the other diastereomer); **LRMS** (ESI$^+$, *m/z*) 481.3 $[M+H]^+$; **HRMS** (ESI$^+$, *m/z*): Calc. for $C_{27}H_{49}N_2O_3S$ $[M+H]^+$ 481.3458, Found: 481.3458.

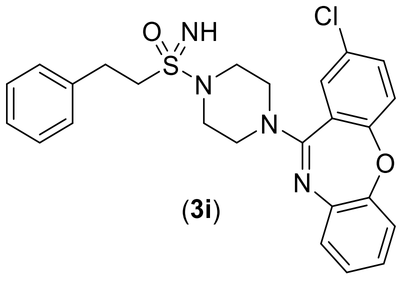

(**3i**)

2-Chloro-11-(4-(2-phenylethylsulfonimidoyl)piperazin-1-yl)dibenzo[*b*,*f*][1,4]oxazepine (3i): The title compound was prepared according to **General Procedure D** using hydrocinnamic acid (0.3 mmol, 45.1 mg), *N*-sulfinyl-*O*-(*tert*-butyl)hydroxylamine (0.2 mmol, 27.0 mg) and 9-mesitylacridine (**PC2**) (5.9 mg, 10 mol%) in dichloromethane (2.0 mL). Amoxapine (0.4 mmol, 126 mg) in toluene (2.0 mL) was used in the second step. Purification by silica gel flash column chromatography (50 – 60% EtOAc in Petrol) afforded **3i** as a colourless oil (41.0 mg, 43%).

R_f = 0.28 (50% EtOAc in Petrol); **IR** (thin film, ν_{max}/cm^{-1}) 3331, 2981, 2853, 1602, 1588, 1243; **^{1}H NMR** (600 MHz, $CDCl_3$) δ 7.41 (dd, J = 8.7, 2.5 Hz, 1H), 7.34 – 7.29 (m, 2H), 7.28 (d, *J* = 2.5 Hz, 1H), 7.26 – 7.22 (m, 3H), 7.20 (d, *J* = 8.7 Hz, 1H), 7.18 – 7.15 (m, 1H), 7.12 – 7.08 (m, 2H), 7.05 – 7.00 (m, 1H), 3.61 (app. br. s, 4H), 3.40 (app. br. s, 4H), 3.29 (td, *J* = 11.9, 5.0 Hz, 1H), 3.25 – 3.15 (m, 2H), 3.08 (td, *J* = 11.7, 4.9 Hz, 1H), 2.28 (br. s, 1H); **^{13}C NMR** (101 MHz, $CDCl_3$) δ 159.5, 158.6, 151.9, 139.8, 138.4, 133.0, 130.6, 129.0, 128.9, 128.6, 127.3, 127.1, 126.0, 125.2, 124.9, 123.0, 120.3, 50.6, 47.9, 46.4, 29.8; **LRMS** (ESI$^+$, *m/z*) 481.1, 483.1 [M+H]$^+$; **HRMS** (ESI$^+$, *m/z*): Calc. for $C_{25}H_{26}{}^{35}ClN_4O_2S$ [M+H]$^+$ 481.1460, Found: 481.1460, Calc. for $C_{25}H_{26}{}^{37}ClN_4O_2S$ [M+H]$^+$ 483.1432, Found: 483.1429.

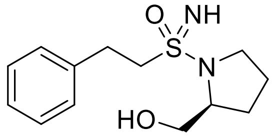

(**3j**)

((2*S*)-1-(2-Phenylethylsulfonimidoyl)pyrrolidin-2-yl)methanol (3j): The title compound was prepared according to **General Procedure D** using hydrocinnamic acid (0.3 mmol, 45.1 mg), *N*-sulfinyl-*O*-(*tert*-butyl)hydroxylamine (0.2 mmol, 27.0 mg) and 9-mesitylacridine (**PC2**) (5.9 mg, 10 mol%) in dichloromethane (2.0 mL). L-prolinol (0.4 mmol, 39.1 μL) in toluene (2.0 mL) was used in the second step. Purification by silica gel flash column chromatography (100% EtOAc) afforded **3j** as a pale-yellow gum (19.8 mg, 37%, 11:10 inseparable mixture of diastereomers).

R_f = 0.21 (100% EtOAc); **IR** (thin film, ν_{max}/cm^{-1}) 3299, 2935. 2873, 1497, 1455, 1237; **^{1}H NMR** (600 MHz, $CDCl_3$, asterisk denotes signals arising from minor diastereomer) δ 7.35 – 7.30 (m, 2H), 7.27 – 7.22 (m, 3H), 3.99 – 3.92 (m, 0.55H), 3.90 – 3.80 (m, 0.5H)*, 3.66 – 3.30 (m, 5H), 3.28 – 3.14 (m, 3H), 2.62 (app. br. s, 2H), 2.07 – 1.98 (m, 1H), 1.98 – 1.88 (m, 1H),

1.86 – 1.77 (m, 1H), 1.78 – 1.66 (m, 1H); **^{13}C NMR** (101 MHz, $CDCl_3$, 11:10 mix of diastereomers, asterisk denotes signals arising from minor diastereomer) δ 138.3, 138.2*, 129.1*, 129.0, 128.67, 128.65*, 127.14*, 127.09, 66.7, 66.3*, 62.4*, 62.0, 51.9, 51.5*, 50.2, 50.1*, 30.04, 30.01*, 29.54*, 29.50, 25.3, 24.8*; **LRMS** (ESI$^+$, *m/z*) 269.1 [M+H]$^+$; **HRMS** (ESI$^+$, *m/z*): Calc. for $C_{13}H_{21}N_2O_2S$ [M+H]$^+$ 269.1318, Found: 269.1319.

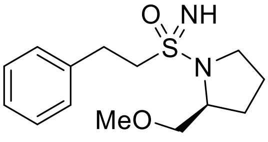

(**3k**)

(2*S*)-2-(Methoxymethyl)-1-(2-phenylethylsulfonimidoyl)pyrrolidine (3k): The title compound was prepared according to **General Procedure D** using hydrocinnamic acid (0.3 mmol, 45.1 mg), *N*-sulfinyl-*O*-(*tert*-butyl)hydroxylamine (0.2 mmol, 27.0 mg) and 9-mesitylacridine (**PC2**) (5.9 mg, 10 mol%) in dichloromethane (2.0 mL). *O*-Methyl-L-prolinol (0.4 mmol, 49.4 μL) in toluene (2.0 mL) was used in the second step. Purification by silica gel flash column chromatography (70 – 80% EtOAc in Petrol) afforded **3k** as a colourless oil (29.9 mg, 53%, 3:2 inseparable mixture of diastereomers).

R_f = 0.24 (70% EtOAc in Petrol); **IR** (thin film, ν_{max}/cm^{-1}) 3271, 2927, 2875, 1455, 1244, 1109; **^{1}H NMR** (600 MHz, $CDCl_3$, asterisk denotes signals arising from minor diastereomer) δ 7.34 – 7.29 (m, 2H), 7.26 – 7.18 (m, 3H), 3.97 – 3.92 (m, 0.6H), 3.92 – 3.87 (m, 0.4H)*, 3.54 – 3.00 (m, 11H), 2.70 – 2.43 (br. m, 1H), 1.98 – 1.89 (m, 2.4H), 1.90 – 1.81 (m, 1.6H)*; **^{13}C NMR** (151 MHz, $CDCl_3$, 3:2 mix of diastereomers, asterisk denotes signals arising from minor diastereomer) δ 138.8, 138.7*, 128.9, 128.6, 126.88*, 126.87, 75.62, 75.59*, 59.17*, 59.16, 59.1*, 58.9, 51.5, 51.3*, 49.8*, 49.5, 30.4, 30.1*, 29.2*, 29.0, 24.9, 24.8* (2 carbon signals are unresolved from the corresponding signals in the other diastereomer); **LRMS** (ESI$^+$, *m/z*) 283.1 [M+H]$^+$; **HRMS** (ESI$^+$, *m/z*): Calc. for $C_{14}H_{23}N_2O_2S$ [M+H]$^+$ 283.1475, Found: 283.1474.

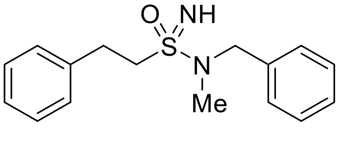

(**3m**)

***N*-Benzyl-*N*-methyl-2-phenylethane-1-sulfonimidamide (3m)**: The title compound was prepared according to **General Procedure D** using hydrocinnamic acid (0.3 mmol, 45.1 mg),

N-sulfinyl-*O*-(*tert*-butyl)hydroxylamine (0.2 mmol, 27.0 mg) and 9-mesitylacridine (**PC2**) (5.9 mg, 10 mol%) in dichloromethane (2.0 mL). *N*-Methylbenzylamine (2.0 mmol, 258 μL) in toluene (2.0 mL) was used in the second step. Purification by silica gel flash column chromatography (30 – 40% EtOAc in Petrol) afforded **3m** as a colourless oil (42.8 mg, 74%).

R$_f$ = 0.25 (40% EtOAc in Petrol); **IR** (thin film, ν_{max}/cm^{-1}) 3316, 3062, 3028, 1496, 1455, 1243; **^{1}H NMR** (400 MHz, CDCl$_3$) δ 7.34 – 7.19 (m, 7H), 7.22 – 7.13 (m, 3H), 4.33 (d, *J* = 14.4 Hz, 1H), 4.27 (d, *J* = 14.4 Hz, 1H), 3.34 – 3.23 (m, 1H), 3.19 – 3.02 (m, 3H), 2.74 (s, 3H), 2.33 (s, 1H); **^{13}C NMR** (101 MHz, CDCl$_3$) δ 138.5, 136.7, 129.0, 128.9, 128.6, 128.2, 128.0, 127.0, 54.8, 51.8, 35.4, 30.1; **LRMS** (ESI$^+$, *m/z*) 289.1 [M+H]$^+$; **HRMS** (ESI$^+$, *m/z*): Calc. for $C_{16}H_{21}N_2OS$ [M+H]$^+$ 289.1368, Found: 289.1368.

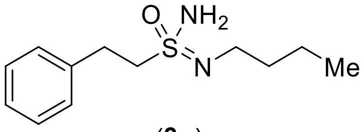

(**3n**)

***N'*-Butyl-2-phenylethane-1-sulfonimidamide (3n)**: The title compound was prepared according to **General Procedure D** using hydrocinnamic acid (0.3 mmol, 45.1 mg), *N*-sulfinyl-*O*-(*tert*-butyl)hydroxylamine (0.2 mmol, 27.0 mg) and 9-mesitylacridine (**PC2**) (5.9 mg, 10 mol%) in dichloromethane (2.0 mL). Butylamine (2.0 mmol, 198 μL) in toluene (2.0 mL) was used in the second step. Purification by silica gel flash column chromatography (50% EtOAc in Petrol) afforded **3n** as a colourless oil (33.5 mg, 70%).

R$_f$ = 0.29 (50% EtOAc in Petrol); **IR** (thin film, ν_{max}/cm^{-1}) 3264, 2958, 2933 2871, 1455, 1237; **^{1}H NMR** (400 MHz, CDCl$_3$) δ 7.35 – 7.29 (m, 2H), 7.28 – 7.22 (m, 3H), 3.45 – 3.24 (m, 2H), 3.21 – 3.10 (m, 2H), 3.02 – 2.74 (m, 4H), 1.46 – 1.23 (m, 4H), 0.90 (t, *J* = 7.2 Hz, 3H); **^{13}C NMR** (101 MHz, CDCl$_3$) δ 138.3, 129.0, 128.6, 127.1, 54.2, 43.7, 32.5, 30.6, 20.0, 13.8; **LRMS** (ESI$^+$, *m/z*) 241.2 [M+H]$^+$; **HRMS** (ESI$^+$, *m/z*): Calc. for $C_{12}H_{21}N_2OS$ [M+H]$^+$ 241.1369, Found: 241.1370.

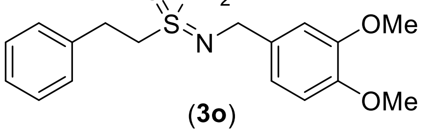

(**3o**)

***N'*-(3,4-Dimethoxybenzyl)-2-phenylethane-1-sulfonimidamide (3o)**: The title compound was prepared according to **General Procedure D** using hydrocinnamic acid (0.3 mmol, 45.1

mg), *N*-sulfinyl-*O*-(*tert*-butyl)hydroxylamine (0.2 mmol, 27.0 mg) and 9-mesitylacridine (**PC2**) (5.9 mg, 10 mol%) in dichloromethane (2.0 mL). 3,4-Dimethoxybenzylamine (2.0 mmol, 302 µL) in toluene (2.0 mL) was used in the second step. Purification by silica gel flash column chromatography (70 – 80% EtOAc in Petrol) afforded **3o** as a colourless oil (52.9 mg, 79%).

R_f = 0.28 (70% EtOAc in Petrol); **IR** (thin film, ν_{max}/cm^{-1}) 3277, 2936, 2836, 1515, 1262, 1239; **^{1}H NMR** (400 MHz, $CDCl_3$) δ 7.31 – 7.25 (m, 2H), 7.25 – 7.19 (m, 1H), 7.14 – 7.09 (m, 2H), 6.82 – 6.78 (m, 3H), 4.13 (d, *J* = 14.1 Hz, 1H), 4.09 (d, *J* = 14.1 Hz, 1H), 3.85 (s, 6H), 3.46 (s, 2H), 3.30 – 3.23 (m, 2H), 3.11 – 3.04 (m, 2H); **^{13}C NMR** (101 MHz, $CDCl_3$) δ 149.3, 148.9, 138.1, 130.1, 128.9, 128.5, 127.0, 120.3, 111.4, 111.3, 56.1, 54.9, 47.8, 30.5; **LRMS** (ESI^+, *m/z*): 335.1 $[M+H]^+$; **HRMS** (ESI^+, *m/z*): Calc. for $C_{17}H_{23}N_2O_3S$ $[M+H]^+$ 335.1424, Found: 335.1425.

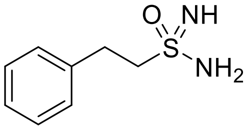

(**3p**)

2-Phenylethane-1-sulfonimidamide (3p): To a solution of *N'*-(3,4-Dimethoxybenzyl)-2-phenylethane-1-sulfonimidamide (**3o**) (0.158 mmol, 52.9 mg) in acetonitrile (1.6 mL) and water (800 µL) cooled to 0 °C was added ceric ammonium nitrate (0.4 mmol, 173 mg). The reaction was stirred at the same temperature for 1h. Solvent was removed *in vacuo*, and the residue purified by silica gel flash column chromatography (80 – 100% EtOAc in Petrol, then 0-5% MeOH in EtOAc) afforded **3p** as an off-white solid (26.2 mg, 90%).

R_f = 0.17 (100% EtOAc); **Melting point** (MeOH) = 54 – 56 °C (decomp.); **IR** (thin film, ν_{max}/cm^{-1}) 3510, 3279. 2923, 1637, 1254, 1034; **^{1}H NMR** (400 MHz, Methanol-d_4) δ 7.34 – 7.25 (m, 4H), 7.25 – 7.20 (m, 1H), 3.41 – 3.35 (m, 2H), 3.17 – 3.11 (m, 2H); **^{13}C NMR** (101 MHz, Methanol-d_4) δ 139.9, 129.8, 129.5, 127.7, 59.0, 31.6; **LRMS** (ESI^+, *m/z*) 185.0 $[M+H]^+$; **HRMS** (ESI^+, *m/z*): Calc. for $C_8H_{13}N_2OS$ $[M+H]^+$ 185.0743, Found: 185.0742.

Note: The product was found to be soluble in water, such that aqueous workup was not effective at separating the product from inorganic impurities. For less water-soluble unprotected sulfonimidamides, aqueous workup may improve the subsequent column chromatography purification.

3.6. Sulfinamide derivatizations

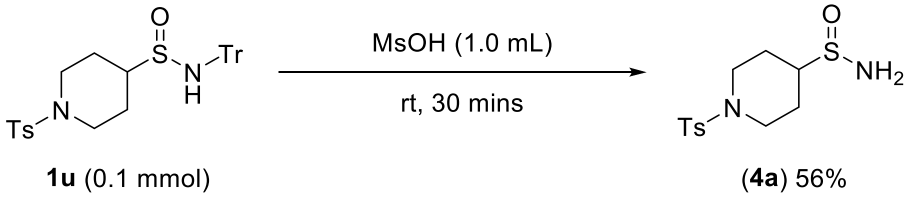

Synthesis of 1-tosylpiperidine-4-sulfinamide (4a): Methanesulfonic acid (1.0 mL) was added to 1-tosyl-*N*-tritylpiperidine-4-sulfinamide (**1u**) (0.1 mmol, 54.5 mg) at room temperature, and the reaction was stirred at the same temperature for 30 min. After completion of the reaction (determined by TLC), it was neutralized with saturated aq. $NaHCO_3$ and extracted with CH_2Cl_2 (2×20 mL). The combined organic layers were washed with brine, dried over anhydrous Na_2SO_4, filtered and concentrated in vacuo. The crude reaction mixture was purified by silica gel flash column chromatography (50 – 100% EtOAc in Petrol) to afford compound **4a** as a white solid (16.0 mg, 56%).

R_f = 0.1 (100% EtOAc); **Melting point** (CH_2Cl_2) = 258 – 260 °C; **IR** (thin film, ν_{max}/cm^{-1}) 3355, 3255, 2922, 2852, 1596, 1450; **^{1}H NMR** (400 MHz, $CDCl_3$) δ 7.68 – 7.59 (m, 2H), 7.33 (app. d, *J* = 8.3 Hz, 2H), 3.98 (s, 2H), 3.80 (m, 2H), 2.48 – 2.33 (m, 3H), 2.43 (s, 3H), 2.14 – 2.01 (m, 1H), 1.89 – 1.67 (m, 2H); **^{13}C NMR** (101 MHz, $CDCl_3$) δ 144.0, 133.3, 129.9, 127.8, 61.3, 45.4, 45.4, 25.2, 24.8, 21.4; **LRMS** (ESI^+, *m/z*) 325.0 $[M+Na]^+$; **HRMS** (ESI^+, *m/z*): Calc. for $C_{12}H_{18}N_2O_3S_2Na$ $[M+Na]^+$ 325.0651, Found: 325.0653.

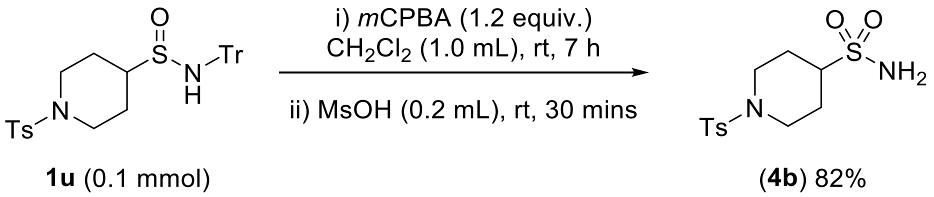

Synthesis of 1-tosylpiperidine-4-sulfonamide (4b) 1-Tosyl-*N*-tritylpiperidine-4-sulfinamide (**1u**) (0.1 mmol, 54.5 mg) was dissolved in dichloromethane (1.0 mL). *m*-CPBA (60% by weight) (0.12 mmol, 34.0 mg) was added at room temperature, and the reaction was stirred at the same temperature for 7 h. After completion of the reaction (determined by TLC), methanesulfonic acid (0.2 mL) was added at room temperature and the reaction was stirred for a further 30 mins. The reaction mixture was then neutralized with saturated aq. $NaHCO_3$ and extracted with dichloromethane (2×20 mL). The combined organic layers were washed with brine, dried over anhydrous Na_2SO_4, filtered and concentrated in vacuo. The crude reaction

mixture was purified by silica gel flash column chromatography (15 – 50% EtOAc in Petrol) to afford compound **4b** as a white solid (26 mg, 82%).

Characterisation as before (**2d**).

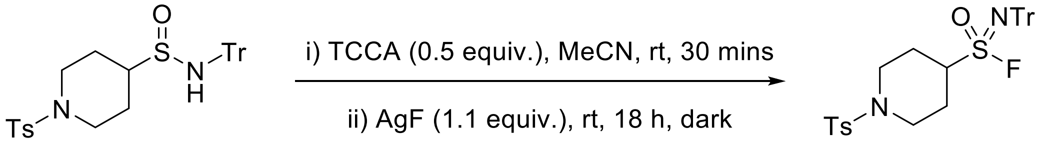

1u (0.1 mmol) (**4c**)

Synthesis of 1-tosyl-*N*-tritylpiperidine-4-sulfonimidoyl fluoride (4c): 1-Tosyl-*N*-tritylpiperidine-4-sulfinamide (**1u**) (0.1 mmol, 54.5 mg) was weighed into an oven-dried 10 mL reaction vial, sealed, evacuated and back-filled with N_2 (×3). Dry acetonitrile (1.0 mL) was then added. Trichloroisocyanuric acid (0.05 mmol, 11.6 mg) was added under a cone of nitrogen, the vial re-sealed and the reaction was stirred at room temperature for 30 mins. AgF (0.11 mmol, 14.0 mg) was then added under a cone of nitrogen, the vial re-sealed and the reaction was stirred at room temperature 18 h (protected from light using aluminium foil). After completion of the reaction (determined by TLC), the reaction mixture was concentrated in vacuo, and the crude reaction mixture was purified by silica gel flash column chromatography (60 – 70% CH_2Cl_2 in Petrol) to afford compound **4c** as a white solid (36.5 mg, 65%).

R$_f$ = 0.26 (20% EtOAc in Petrol); **Melting point** (CH_2Cl_2) = 216 – 218 °C; **IR** (thin film, ν_{max}/cm^{-1}) 2958, 2928, 2856, 1449, 1344 1169; **^{1}H NMR** (400 MHz, $CDCl_3$) δ 7.61 (d, *J* = 8.3 Hz, 2H), 7.25 (dddd, *J* = 14.2, 8.6, 7.0, 5.0 Hz, 17H), 3.94 (d, *J* = 12.3 Hz, 2H), 3.17 (tt, *J* = 11.9, 3.4 Hz, 1H), 2.38 (s, 3H), 2.37 – 2.27 (m, 4H), 2.21 – 2.01 (m, 2H); **^{13}C NMR** (101 MHz, $CDCl_3$) δ 146.2 (d, *J* = 4.3 Hz), 144.2, 133.0, 130.0, 128.8, 127.9, 127.8, 127.2, 73.6 (d, *J* = 4.4 Hz), 61.8 (d, *J* = 21.1 Hz), 45.1, 26.8, 26.4, 21.7; **^{19}F NMR** (377 MHz, $CDCl_3$) δ 68.24; **HRMS** (ESI$^+$, *m/z*): Calc. for $C_{31}H_{31}FN_2O_3S_2Na$ $[M+Na]^+$ 585.1655, Found: 585.1644.

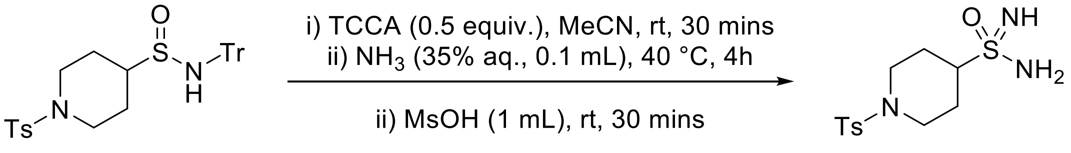

1u (0.1 mmol) (**4d**) 70%

Synthesis of 1-tosylpiperidine-4-sulfonimidamide (4d): 1-Tosyl-*N*-tritylpiperidine-4-sulfinamide (**1u**) (0.1 mmol, 54.5 mg) was weighed into an oven-dried 10 mL reaction vial,

sealed, evacuated and back-filled with N_2 (×3). Dry acetonitrile (1.0 mL) was then added. Trichloroisocyanuric acid (0.05 mmol, 11.6 mg) was added under a cone of nitrogen, the vial re-sealed and the reaction was stirred at room temperature for 30 mins. 35% aq NH_3 (0.1 mL) was then added and the reaction was stirred at 40 °C for 4 h. The reaction was cooled to room temperature and methanesulfonic acid (1.0 mL) was added dropwise and the mixture was stirred at rt for 30 min. After completion of the reaction (determined by TLC), the reaction mixture was then neutralized with saturated aq. $NaHCO_3$ and extracted with EtOAc (4×20 mL). The combined organic layers were washed with brine, dried over anhydrous Na_2SO_4, filtered and concentrated in vacuo. The crude reaction mixture was purified by silica gel flash column chromatography (0 – 2% MeOH in EtOAc) to afford compound **4d** as a white solid (22.2 mg, 70%).

R_f = 0.16 (2% MeOH in EtOAc); **Melting point** (MeOH) = 157 – 159 °C; **IR** (thin film, ν_{max}/cm^{-1}) 3296, 3042, 2942, 1333, 1249, 1159; **^{1}H NMR** (400 MHz, DMSO-*d*$_6$) δ 7.62 (d, *J* = 8.3 Hz, 2H), 7.44 (d, *J* = 8.0 Hz, 2H), 5.51 (br. s, 2H), 3.73 (d, *J* = 11.7 Hz, 2H), 2.77 (tt, *J* = 11.8, 3.7 Hz, 1H), 2.40 (s, 3H), 2.25 (td, *J* = 12.3, 2.3 Hz, 2H), 2.12 – 2.05 (m, 2H), 1.64 (qd, *J* = 12.7, 4.2 Hz, 2H); **^{13}C NMR** (101 MHz, DMSO-*d*$_6$) δ 143.6, 132.8, 129.9, 127.4, 59.0, 44.9, 25.9, 21.0; **LRMS** (ESI$^+$, *m/z*) 318.0 [M+H]$^+$; **HRMS** (ESI$^+$, *m/z*): Calc. for $C_{12}H_{20}N_3O_3S_2$ [M+H]$^+$ 318.0941, Found: 318.0937.

3.7. Synthesis of photocatalysts:

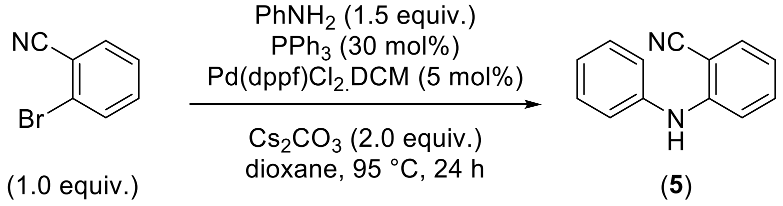

Synthesis of 9-(2-chlorophenyl)acridine (PC1): The title catalyst was prepared according to a literature procedure.[1]

Step 1: 2-Bromobenzonitrile (455 mg, 2.5 mmol, 1.0 equiv.), Pd(dppf)Cl2.DCM (102 mg, 0.125 mmol, 5 mol%), triphenylphosphine (197 mg, 3.75 mmol, 0.3 equiv.), and caesium carbonate (1.63 g, 5 mmol, 2.0 equiv.) were weighed into a dry two-neck round-bottomed flask fitted with a condenser. This was sealed, evacuated, and back-filled with nitrogen (×3). Aniline (342 µL, 3.75 mmol, 1.5 equiv.) and 10 mL of anhydrous dioxane were added and the mixture was then heated to 95 °C for 24 hours. Upon completion, the reaction was quenched with water and extracted with ethyl acetate (3×20 mL). The combined organic layers were washed with brine, dried over anhydrous Na_2SO_4, filtered and concentrated in vacuo. The crude product was purified silica gel flash column chromatography (1 – 5% EA/PE) to give 2-(phenylamino)benzonitrile (**5**) as a pale-yellow solid (410 mg, 84%).

R_f = 0.43 (2% EtOAc in Petrol); **^{1}H NMR** (400 MHz, $CDCl_3$) δ 7.54 – 7.47 (m, 1H), 7.41 – 7.32 (m, 3H), 7.23 – 7.17 (m, 3H), 7.17 – 7.09 (m, 1H), 6.84 (td, J = 7.8, 1.0 Hz, 1H), 6.33 (s, 1H); **^{13}C NMR** (101 MHz, $CDCl_3$) δ ^{13}C NMR (101 MHz, $CDCl_3$) δ 147.4, 140.0, 134.0, 133.2, 129.8, 124.4, 121.9, 119.4, 117.7, 114.3, 98.6; **LRMS** (ESI$^+$, *m/z*) 195.2 $[M+H]^+$; **HRMS** (ESI$^+$, *m/z*) Calc. for $C_{13}H_{11}N_2$ $[M+H]^+$ 195.0917, Found: 195.0917.

The spectral data is consistent with the literature.[1]

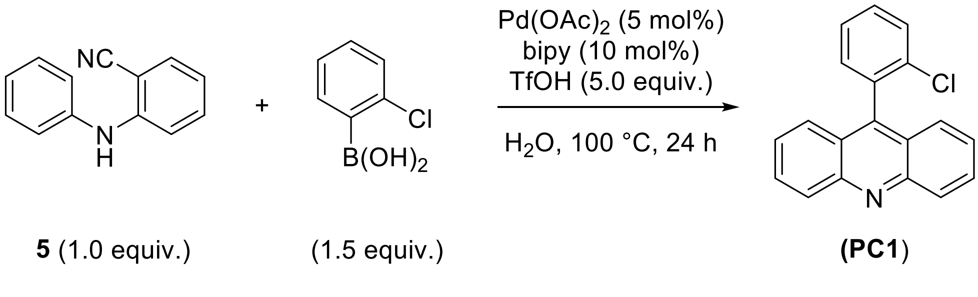

Step 2: 2-(Phenylamino)benzonitrile (**5**) (388 mg, 2.0 mmol), *o*-chlorobenzene (468 mg, 3.0 mmol), $Pd(OAc)_2$ (22.0 mg, 0.1 mmol) and 2,2′-bipyridyl (31.0 mg, 0.2 mmol) were weighed into a round-bottom flask, and water (8.0 mL) was then added. The mixture was stirred before dropwise addition of trifluoromethanesulfonic acid (3.00 g, 20 mmol). The heterogeneous reaction mixture was then heated to 100 °C and stirred for 24 hours. The reaction mixture was basified with saturated $NaHCO_3$, and extracted with ethyl acetate (3×50 mL). The combined organic layers were washed with brine, dried over anhydrous Na_2SO_4, filtered and concentrated in vacuo. The crude product was purified silica gel flash column chromatography (5 – 10% EtOAc in Petrol) to give 9-(2-chlorophenyl)acridine **(PC1)** as a yellow solid (202 mg, 35%).

$\mathbf{R_f}$ = 0.4 (20% EtOAc in Petrol); ^{1}**H NMR** (400 MHz, $CDCl_3$) δ 8.31 (dt, *J* = 8.8, 1.0 Hz, 2H), 7.78 (ddd, *J* = 8.8, 6.4, 1.5 Hz, 2H), 7.69 – 7.63 (m, 1H), 7.58 – 7.41 (m, 6H), 7.38 – 7.34 (m, 1H); ^{13}**C NMR** (101 MHz, $CDCl_3$) δ 149.0, 144.2, 135.1, 134.4, 132.2, 130.2, 130.1, 130.0, 129.9, 127.0, 126.4, 126.2, 125.1 (*1 aromatic carbon signal is not observed due to signal overlap*); **LRMS** (ESI^+, *m/z*) 290.0 $[M+H]^+$; **HRMS** (ESI^+, *m/z*): Calc. for $C_{19}H_{13}{}^{35}ClN$ $[M+H]^+$ 290.0731, Found: 290.0731.

The spectral data is consistent with the literature.[1]

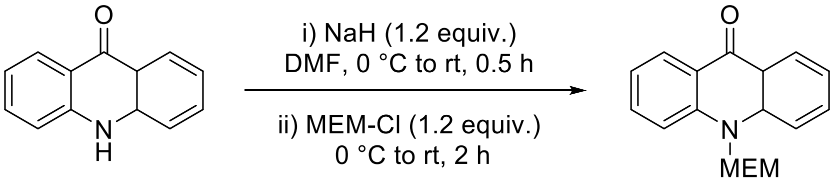

(1.0 equiv.) (**6**)

Synthesis of 9-mesitylacridine (PC2): The title catalyst was prepared according to a literature procedure.[2]

Step 1: 9-acridone (2.00 g, 10.2 mmol) was dissolved in dry DMF in an oven-dried round bottom flask, and the resulting solution cooled to 0 °C under nitrogen. NaH (60% in mineral oil) (491 mg, 12.3 mmol) was then added in portions. The reaction was allowed to warm to room temperature and stirred for 30 mins. The reaction mixture was cooled to 0 °C, and MEM-Cl (1.46 mL, 12.8 mmol) was then added dropwise. The reaction was allowed to warm to room temperature and stirred for 30 mins. The reaction mixture was quenched with water and stirred for a further 30 mins, before extraction with ethyl acetate (3×50 mL). The combined organic layers were washed with water, brine, dried over Na_2SO_4, filtered and concentrated in vacuo.

The crude material was purification by silica gel flash column chromatography (2% MeOH in CH_2Cl_2) afforded *N*-MEM-9-acridone **(6)** as an off-white solid (1.90 g, 66%).

R_f = 0.45 (2% MeOH in DCM); **^{1}H NMR** (400 MHz, $CDCl_3$) 8.57 – 8.49 (m, 2H), 7.78 – 7.67 (m, 4H), 7.33 (ddd, *J* = 8.0, 5.9, 2.0 Hz, 2H), 5.82 (s, 2H), 3.92 – 3.79 (m, 2H), 3.67 – 3.62 (m, 2H), 3.44 (s, 3H); **^{13}C NMR** (101 MHz, $CDCl_3$) δ 178.6, 142.6, 134.1, 127.8, 122.6, 122.3, 115.4, 77.3, 72.4, 67.5, 59.4; **LRMS** (ESI$^+$, *m/z*) 284.1 $[M+H]^+$; **HRMS** (ESI$^+$, *m/z*): Calc. for $C_{17}H_{20}NO_3$ $[M+H]^+$ 284.1281, Found: 284.1282.

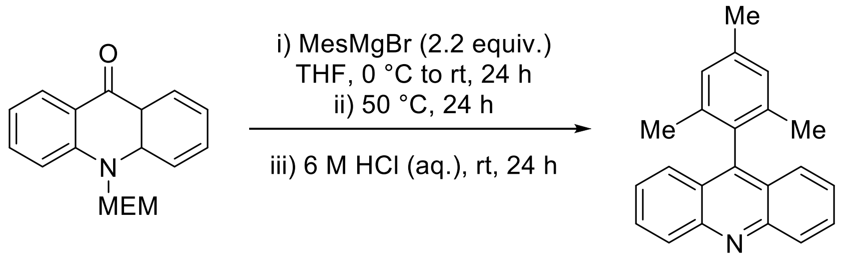

6 (1.0 equiv.) (**PC2**)

Step 2: *N*-(2-methoxy-ethoxymethyl)-9-acridone (**6**) (500 mg, 1.76 mmol) was dissolved in dry tetrahydrofuran (20 mL) in an oven-dried round bottom flask and the resulting solution cooled to 0 °C under nitrogen. 2-Mesitylmagnesium bromide (1 M in THF) (3.87 mL, 2.2 equiv.) was added dropwise at the same temperature. The reaction mixture was allowed to warm to room temperature and stirred for 24 h, followed by a further 24 h at 50 °C. At this point 50% aqueous HCl solution (50 mL) was added to the reaction mixture, and the reaction stirred for a further 24 h at room temperature. After completion (determined by TLC), it was basified with saturated aq. $NaHCO_3$ and extracted with $CHCl_3$ (3×50 mL). The combined organic layers were washed with brine, dried over Na_2SO_4, filtered and concentrated in vacuo. The crude material was purification by silica gel flash column chromatography (5 – 20% EtOAc in Petrol) afforded 9-mesitylacridine **(PC2)** as an off-white solid (350 mg, 67%).

R_f = 0.25 (10% EtOAc in Petrol); **^{1}H NMR** (400 MHz, $CDCl_3$) δ 8.29 (d, *J* = 8.8 Hz, 2H), 7.77 (ddd, *J* = 8.7, 6.5, 1.5 Hz, 2H), 7.55 – 7.48 (m, 2H), 7.40 (ddd, *J* = 8.7, 6.5, 1.1 Hz, 2H), 7.09 (s, 2H), 2.45 (s, 3H), 1.71 (s, 6H); **^{13}C NMR** (101 MHz, $CDCl_3$) δ 149.2, 138.2, 136.8, 130.3, 130.0, 128.5, 126.2, 126.0, 125.2, 21.4, 20.1; **LRMS** (ESI$^+$, *m/z*) 298.2 $[M+H]^+$; **HRMS** (ESI$^+$, *m/z*): Calc. for $C_{22}H_{20}N$ $[M+H]^+$ 298.1590, Found: 298.1586.

The compound spectral data is consistent with the literature.[2]

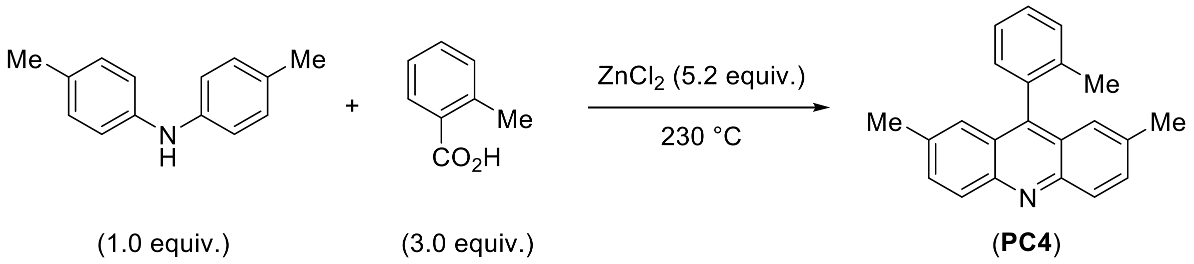

Synthesis of 2,7-dimethyl-9-(*o*-tolyl)acridine (PC4): The title catalyst was prepared according to a literature procedure.[3] Di-*p*-tolylamine (1.42 g, 7.2 mmol), 2-methylbenzoic acid (2.94 g, 21.6 mmol) and zinc chloride (5.10 g, 37.4 mmol) were weighed into an oven-dried 10 mL round bottom flask. The reaction flask was heated at 230 °C for 18 h. After completion, the reaction mixture was quenched with concentrated aqueous ammonia to dissolve the zinc chloride. The insoluble residue was extracted with dichloromethane (3×70 mL). The combined organic layers were washed with water, brine, dried over Na_2SO_4, filtered and solvent removed in vacuo. The crude material was purified by silica gel flash column chromatography (10 – 20% EtOAc in Petrol) afford 2,7-dimethyl-9-(*o*-tolyl)acridine (**PC4**) as yellow solid (1.19 g, 80%).

R_f = 0.4 (20% EtOAc in Petrol); **^{1}H NMR** (400 MHz, $CDCl_3$) δ 8.16 (d, *J* = 8.9 Hz, 2H), 7.57 (dd, *J* = 8.9, 2.0 Hz, 2H), 7.53 – 7.43 (m, 2H), 7.41 (td, *J* = 7.3, 1.8 Hz, 1H), 7.25 – 7.18 (m, 3H), 2.43 (d, *J* = 1.1 Hz, 6H), 1.88 (s, 3H); **^{13}C NMR** (101 MHz, $CDCl_3$) δ 147.4, 144.6, 137.1, 136.1, 135.6, 132.6, 130.4, 130.3, 129.6, 128.5, 126.0, 125.4, 124.5, 22.1, 19.9; **LRMS** (ESI$^+$, *m/z*) 298.1 [M+H]$^+$; **HRMS** (ESI$^+$, *m/z*): Calc. for $C_{22}H_{20}N$ [M+H]$^+$ 298.1590, Found: 298.1586.

The spectral data is consistent with the literature.[3]

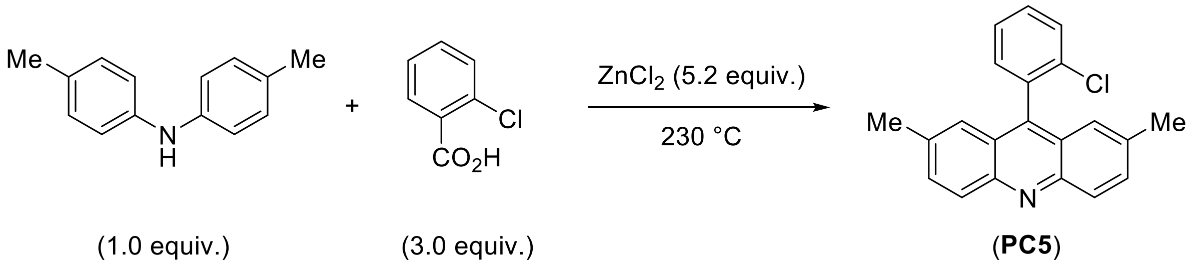

Synthesis of 9-(2-chlorophenyl)-2,7-dimethylacridine (PC5): The title catalyst was prepared according to a literature procedure.[4] Di-*p*-tolylamine (710 mg, 3.60 mmol), 2-chlorobenzoic acid (1.69 g, 10.8 mmol) and zinc chloride (2.55 g, 18.7 mmol) were weighed into an oven-

dried 10 mL pressure tube. The reaction flask was heated at 230 °C for 18 h. After completion, the reaction mixture was quenched with concentrated aqueous ammonia to dissolve the zinc chloride. The insoluble residue was extracted with ethyl acetate (3×75 mL). The combined organic layers were washed with water, brine, dried over Na_2SO_4, filtered and solvent removed in vacuo. The crude material was purified by silica gel flash column chromatography (10 – 20% EtOAc in Petrol) to afford 9-(2-chlorophenyl)-2,7-dimethyl-9-(*o*-tolyl)acridinedimethylacridine (**PC5**) as an off-white solid (362 mg, 57%).

R_f = 0.35 (10% EtOAc in Petrol); **^{1}H NMR** (400 MHz, $CDCl_3$) δ 8.17 (d, *J* = 8.9 Hz, 2H), 7.68 (dd, *J* = 7.9, 1.3 Hz, 1H), 7.62 – 7.55 (m, 3H), 7.51 (td, *J* = 7.4, 1.4 Hz, 1H), 7.34 (dd, *J* = 7.4, 1.7 Hz, 1H), 7.19 (s, 2H), 2.45 (d, *J* = 0.8, 6H); **^{13}C NMR** (101 MHz, $CDCl_3$) δ 147.4, 141.8, 136.0, 135.5, 134.4, 132.7, 132.2, 130.1, 130.0, 129.6, 127.1, 125.2, 124.2, 22.2; **LRMS** (ESI^+, *m/z*) 318.0 $[M+H]^+$; **HRMS** (ESI^+, *m/z*): Calc. for $C_{21}H_{17}ClN$ $[M+H]^+$ 318.1044, Found: 318.1029.

The spectral data is consistent with the literature.[4]

4. References

1. Ye X, Xu B, Sun J, Dai L, Shao Y, Zhang Y*, et al.* Pd-Catalyzed Approach for Assembling 9-Arylacridines via a Cascade Tandem Reaction of 2-(Arylamino)benzonitrile with Arylboronic Acids in Water. *J Org Chem* 2020, **85**(20)**:** 13004-13014.

2. Benniston AC, Harriman A, Li P, Rostron JP, Van Ramesdonk HJ, Groeneveld MM*, et al.* Charge Shift and Triplet State Formation in the 9-Mesityl-10-methylacridinium Cation. *J Am Chem Soc* 2005, **127**(46)**:** 16054-16064.

3. Zadykowicz B, Storoniak P. Lattice energetics and thermochemistry of acridine derivatives and substituted acridinium trifluoromethanesulphonates. *J Therm Anal Calorim* 2017, **129**(3)**:** 1613-1624.

4. Nguyen VD, Trevino R, Greco SG, Arman HD, Larionov OV. Tricomponent Decarboxysulfonylative Cross-coupling Facilitates Direct Construction of Aryl Sulfones and Reveals a Mechanistic Dualism in the Acridine/Copper Photocatalytic System. *ACS Catal* 2022, **12**(14)**:** 8729-8739.

5. NMR Spectra

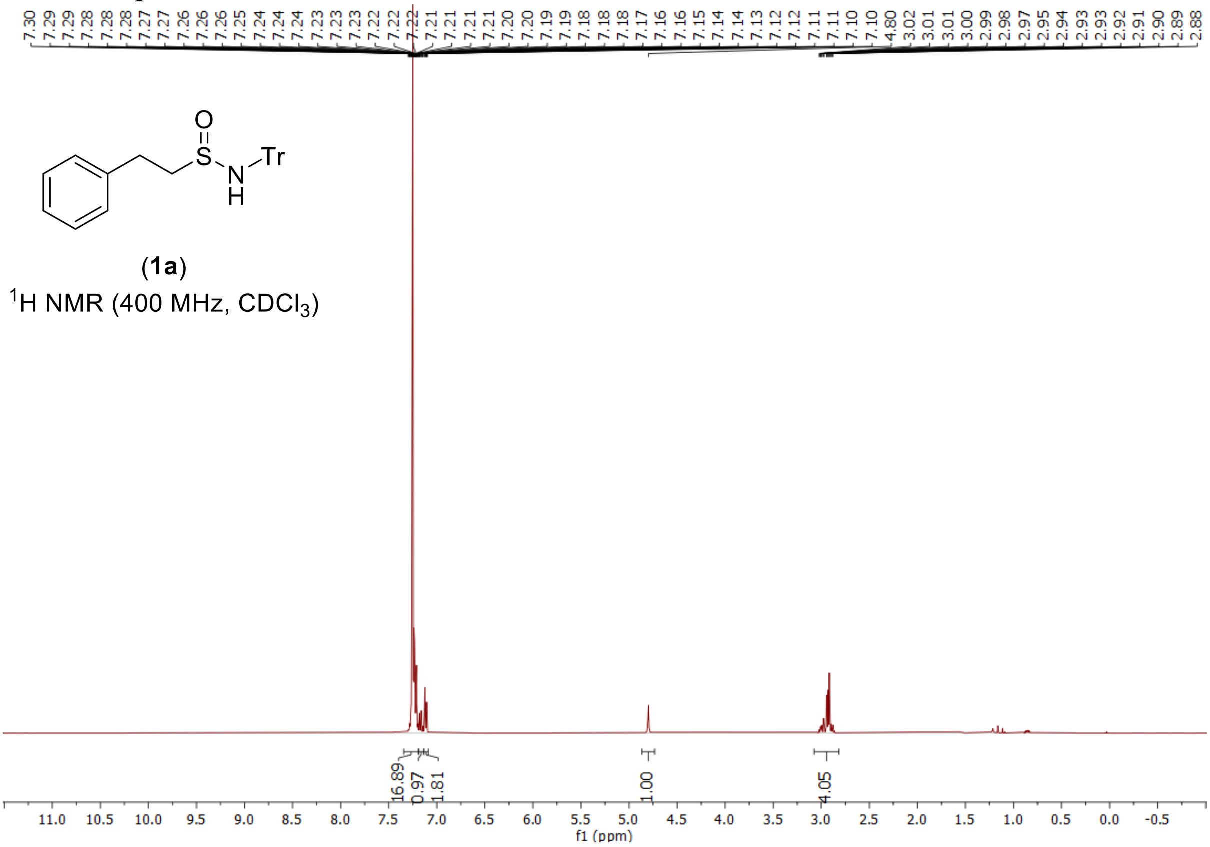

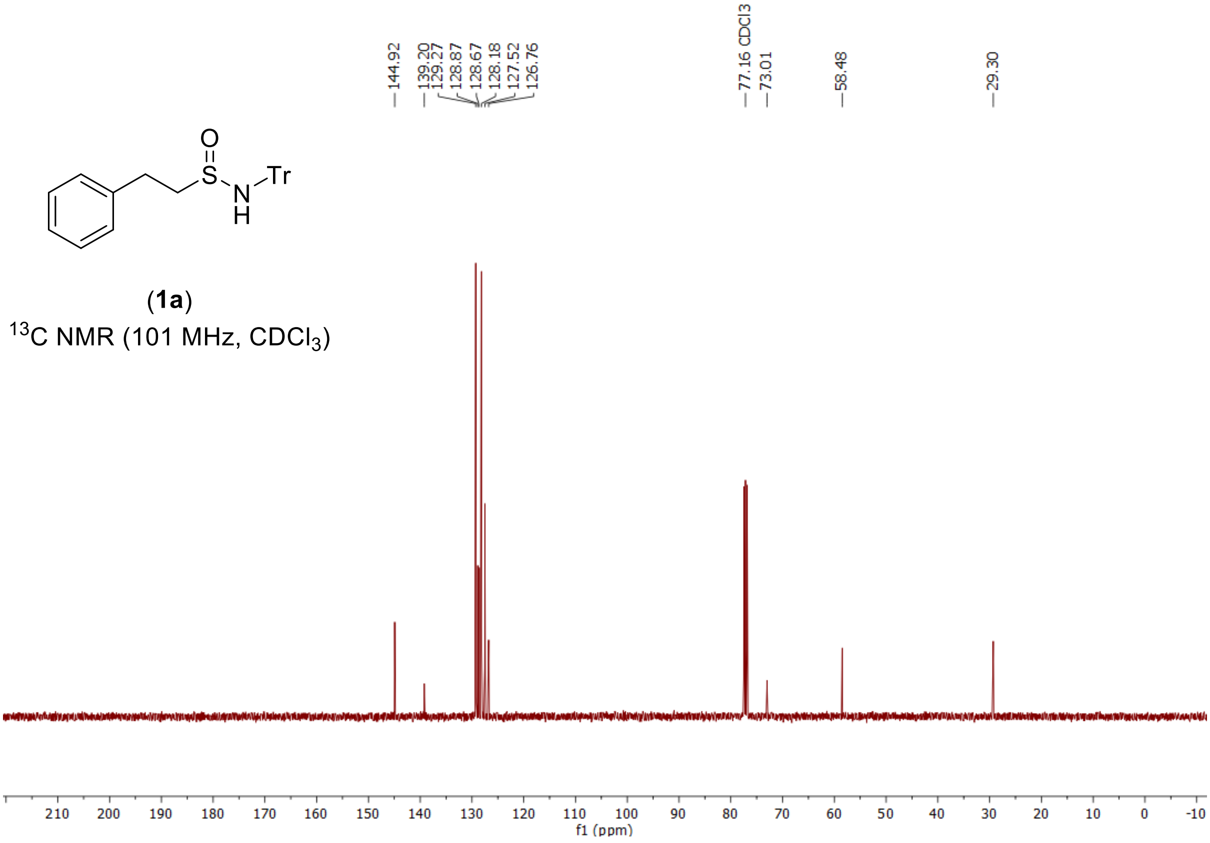

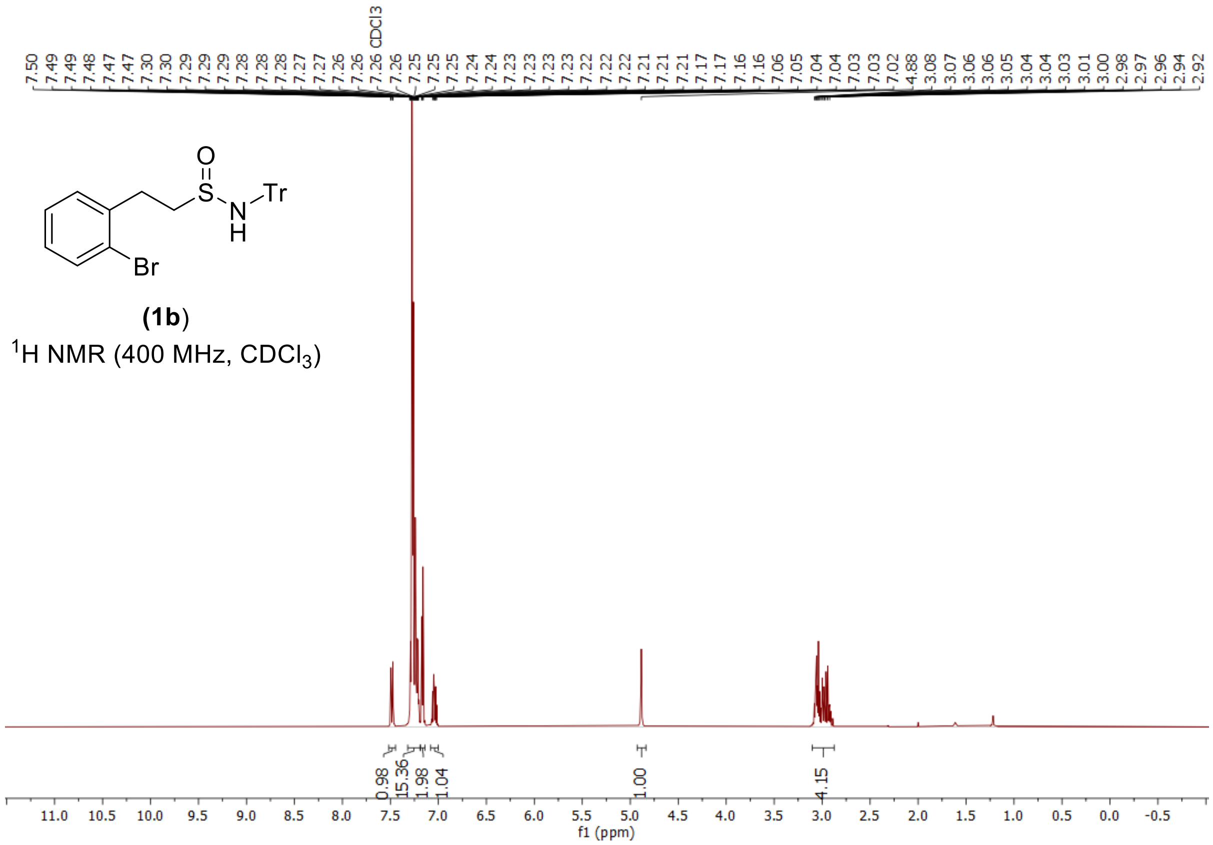

(**1b**)

^{1}H NMR (400 MHz, $CDCl_3$)

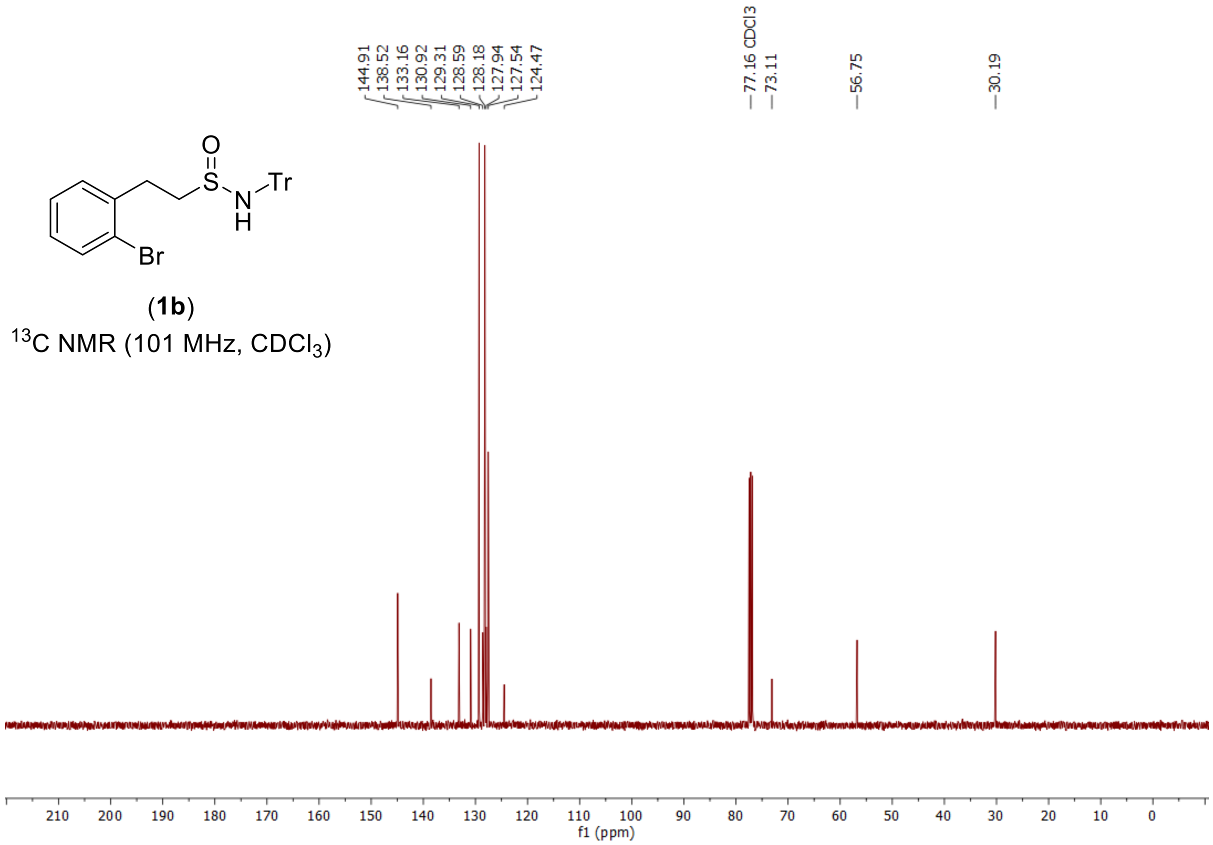

(**1b**)

^{13}C NMR (101 MHz, $CDCl_3$)

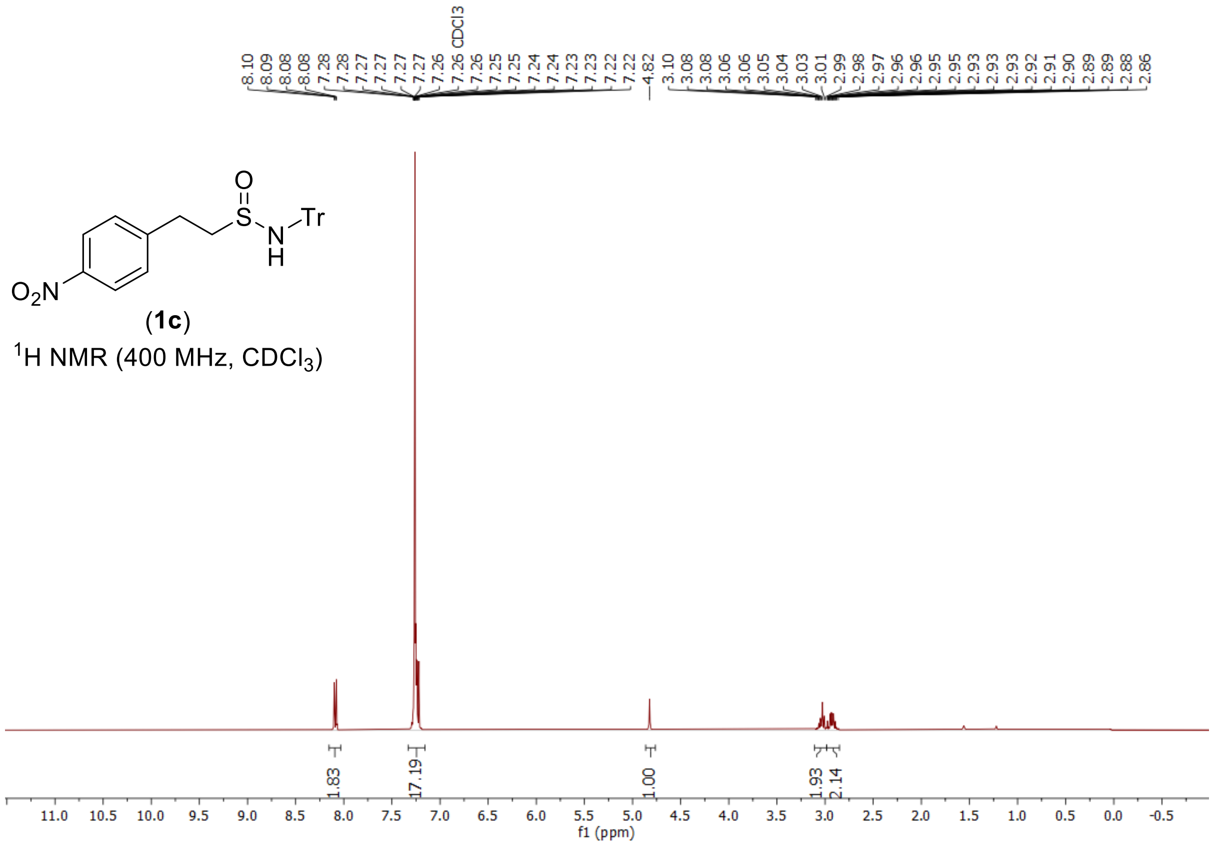

(**1c**)

^{1}H NMR (400 MHz, $CDCl_3$)

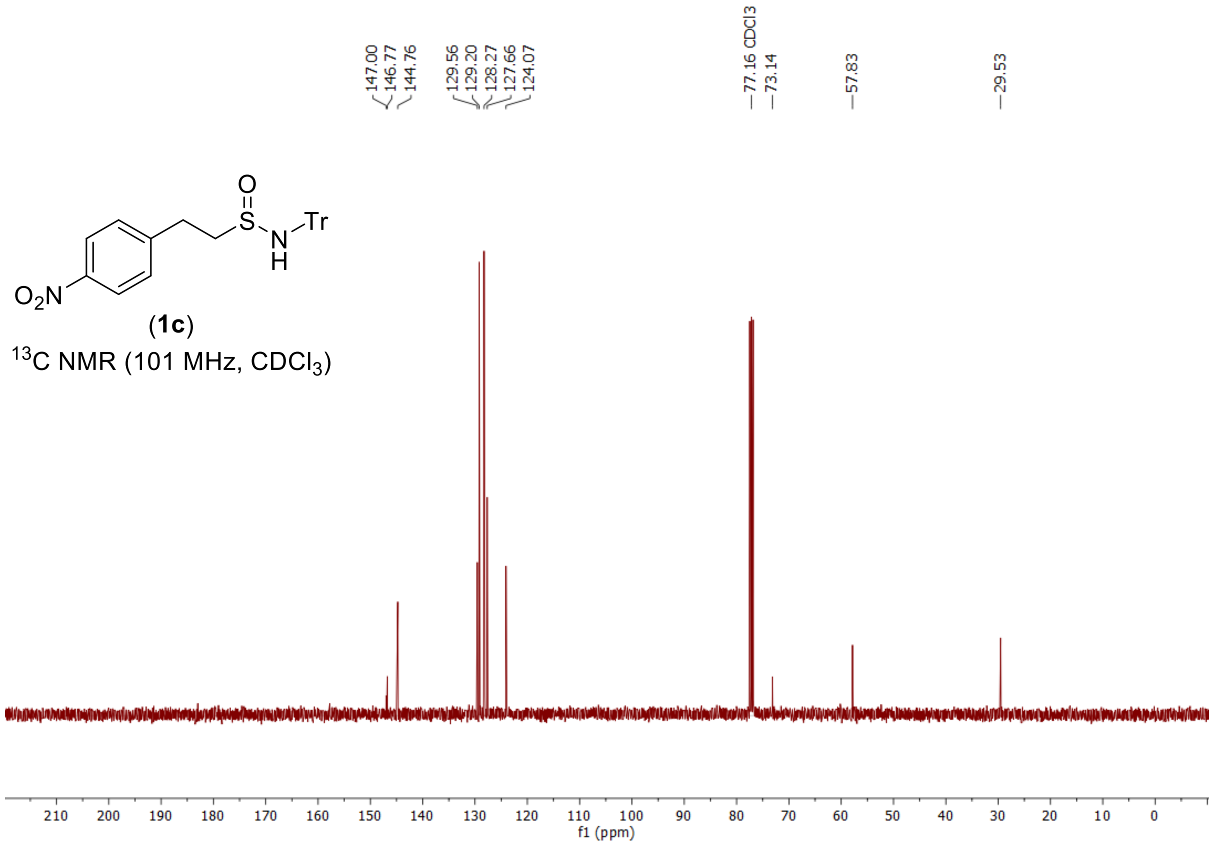

(**1c**)

^{13}C NMR (101 MHz, $CDCl_3$)

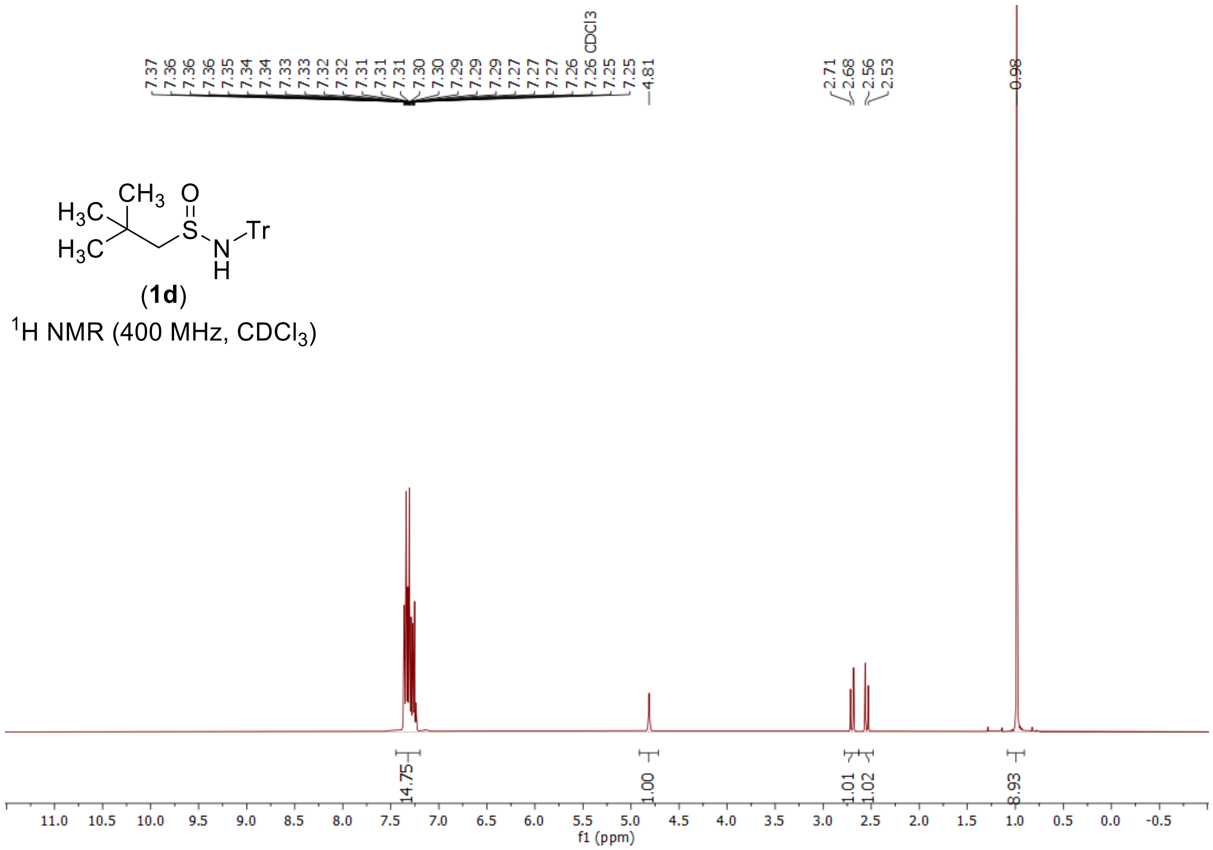
H3C CH3 O S N Tr H H3C
(1d)
1H NMR (400 MHz, CDCl3)
7.37 7.36 7.36 7.36 7.35 7.34 7.34 7.33 7.33 7.33 7.32 7.32 7.31 7.31 7.31 7.30 7.30 7.29 7.29 7.29 7.29 7.27 7.27 7.27 7.26 7.26 CDCl3 7.25 7.25
4.81
2.71 2.68 2.56 2.53
0.98
14.75
1.00
1.01
1.02
8.93
f1 (ppm)

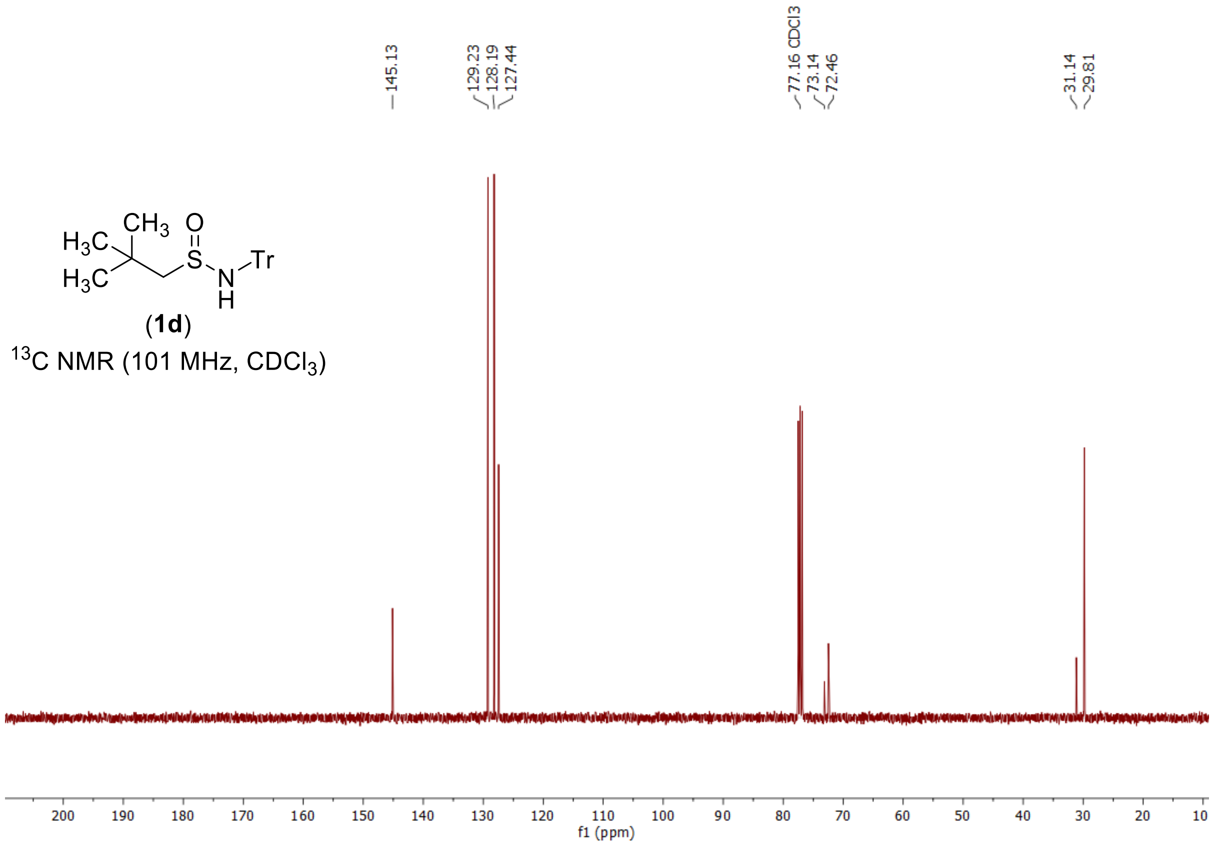
H3C CH3 O S N Tr H H3C
(1d)
13C NMR (101 MHz, CDCl3)
145.13
129.23 128.19 127.44
77.16 CDCl3
73.14 72.46
31.14 29.81
f1 (ppm)

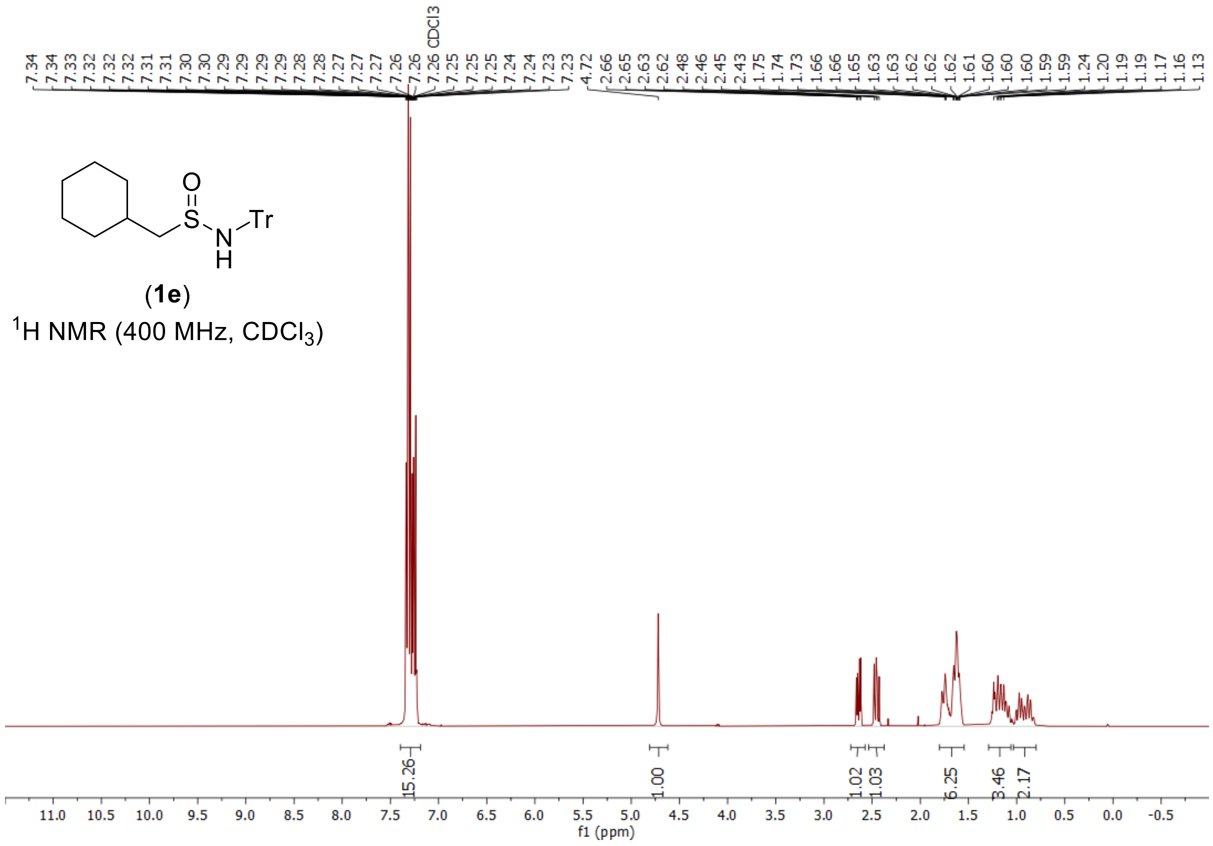
(1e)
^{1}H NMR (400 MHz, $CDCl_3$)
7.34 7.34 7.33 7.32 7.32 7.32 7.31 7.30 7.30 7.29 7.29 7.29 7.29 7.28 7.28 7.27 7.27 7.27 7.26 7.26 7.26 CDCl3 7.25 7.25 7.25 7.24 7.24 7.23 7.23
4.72 2.66 2.65 2.63 2.62 2.48 2.46 2.45 2.43 1.75 1.74 1.73 1.66 1.66 1.65 1.63 1.63 1.62 1.62 1.62 1.61 1.60 1.60 1.60 1.59 1.59 1.24 1.20 1.19 1.19 1.17 1.16 1.13
15.26 1.00 1.02 1.03 6.25 3.46 2.17
f1 (ppm)

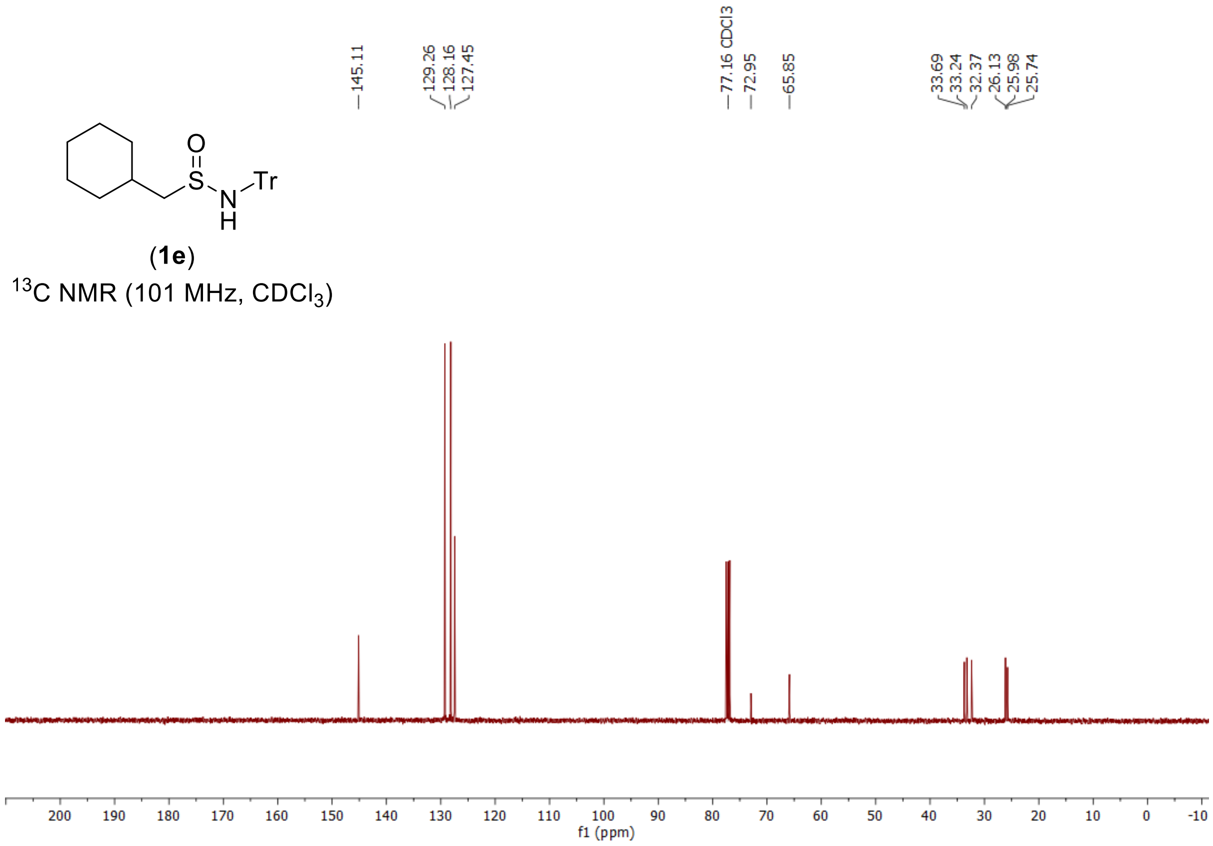
(1e)
^{13}C NMR (101 MHz, $CDCl_3$)
145.11
129.26 128.16 127.45
77.16 CDCl3 72.95 65.85
33.69 33.24 32.37 26.13 25.98 25.74
f1 (ppm)

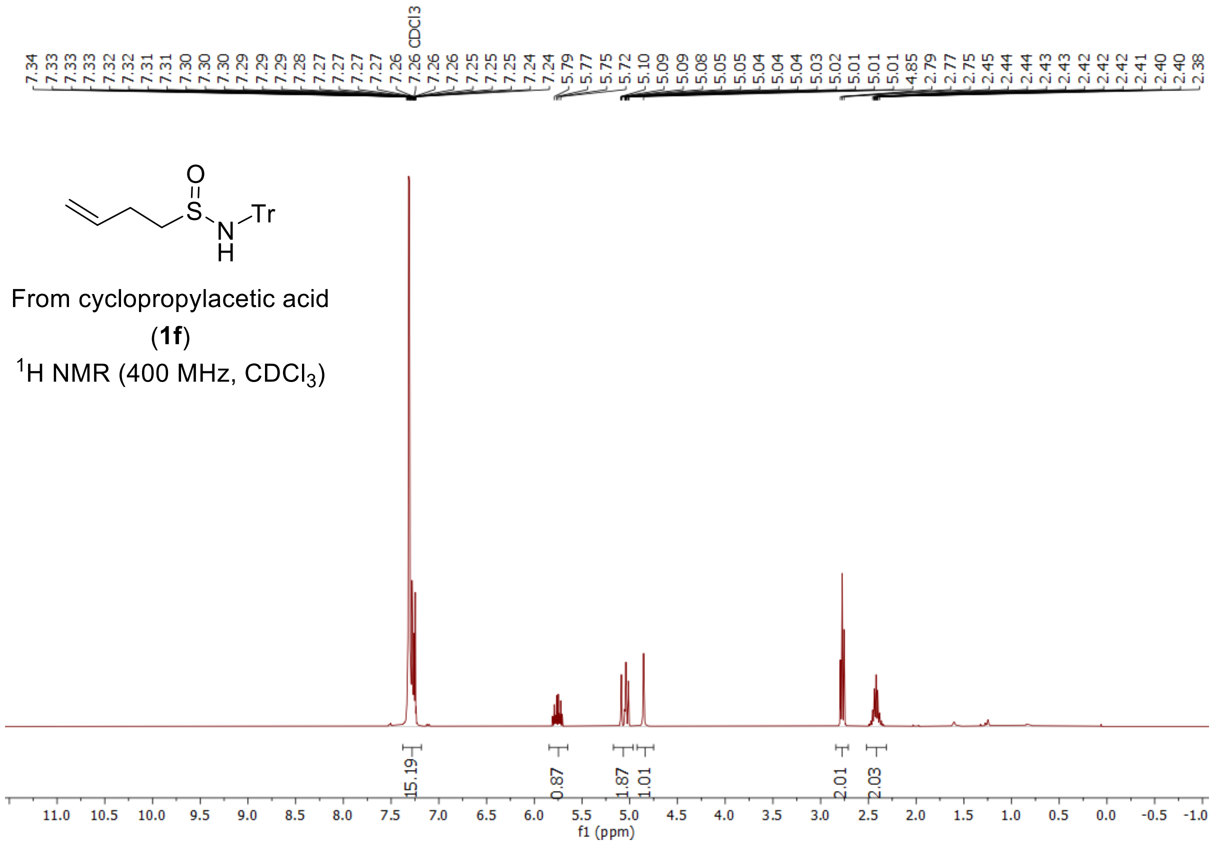

Tr
N
H
S
O
From cyclopropylacetic acid
(1f)
1H NMR (400 MHz, CDCl3)
f1 (ppm)

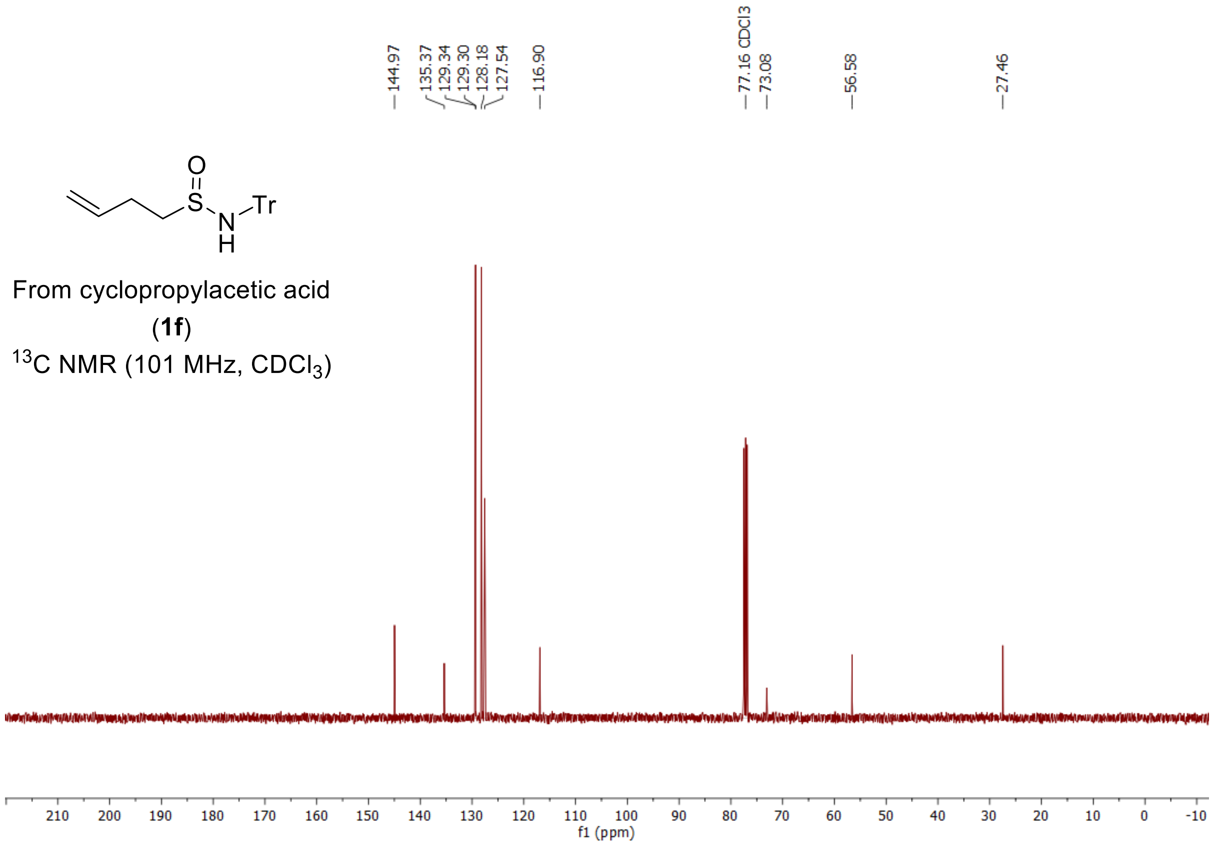

Tr
N
H
S
O
From cyclopropylacetic acid
(1f)
13C NMR (101 MHz, CDCl3)
144.97
135.37
129.34
129.30
128.18
127.54
116.90
77.16 CDCl3
73.08
56.58
27.46
f1 (ppm)

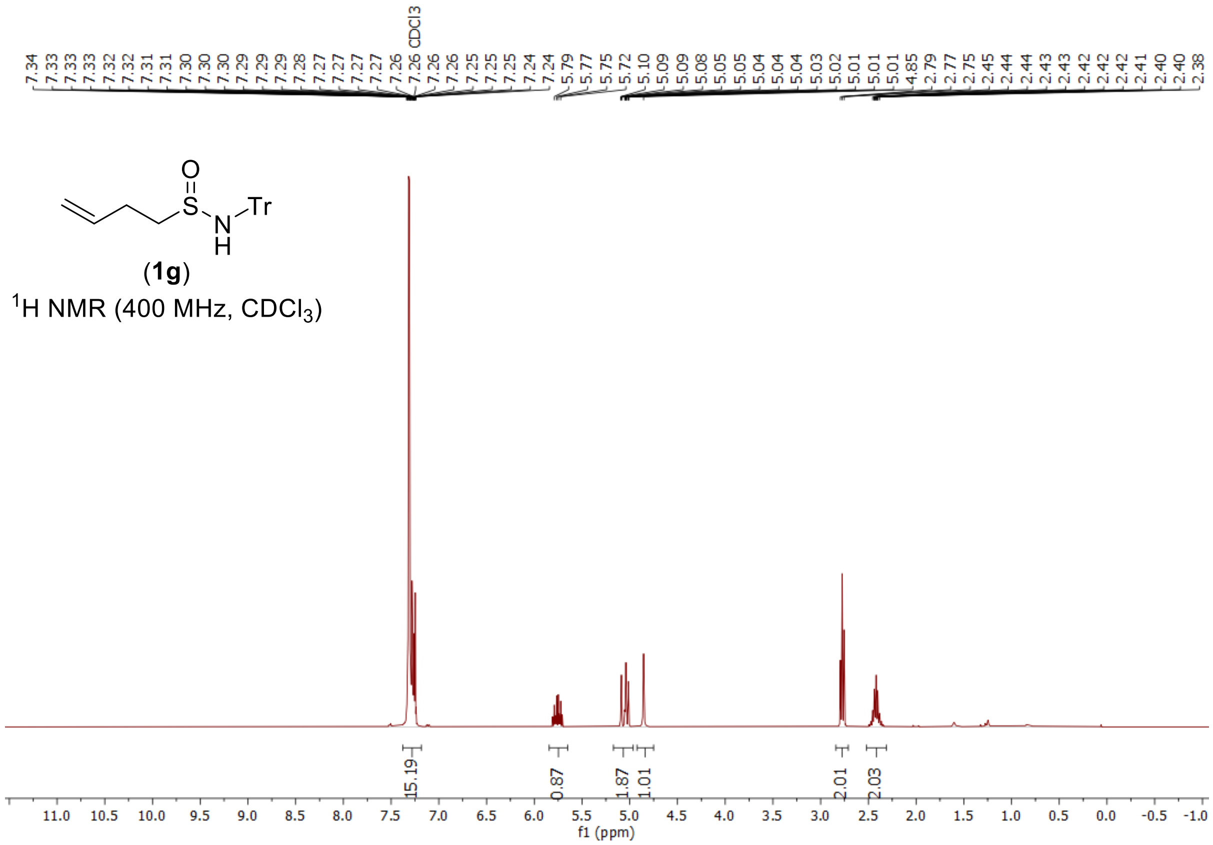
O
S
N
Tr
H
(1g)
1H NMR (400 MHz, CDCl3)
7.34 7.33 7.33 7.33 7.32 7.32 7.31 7.31 7.30 7.30 7.30 7.29 7.29 7.29 7.28 7.27 7.27 7.27 7.27 7.26 7.26 CDCl3 7.26 7.26 7.25 7.25 7.25 7.24 7.24 5.79 5.77 5.75 5.72 5.10 5.09 5.09 5.08 5.05 5.05 5.04 5.04 5.04 5.03 5.02 5.01 5.01 5.01 4.85 2.79 2.77 2.75 2.45 2.44 2.44 2.43 2.42 2.42 2.42 2.41 2.40 2.40 2.38
15.19 0.87 1.87 1.01 2.01 2.03
f1 (ppm)

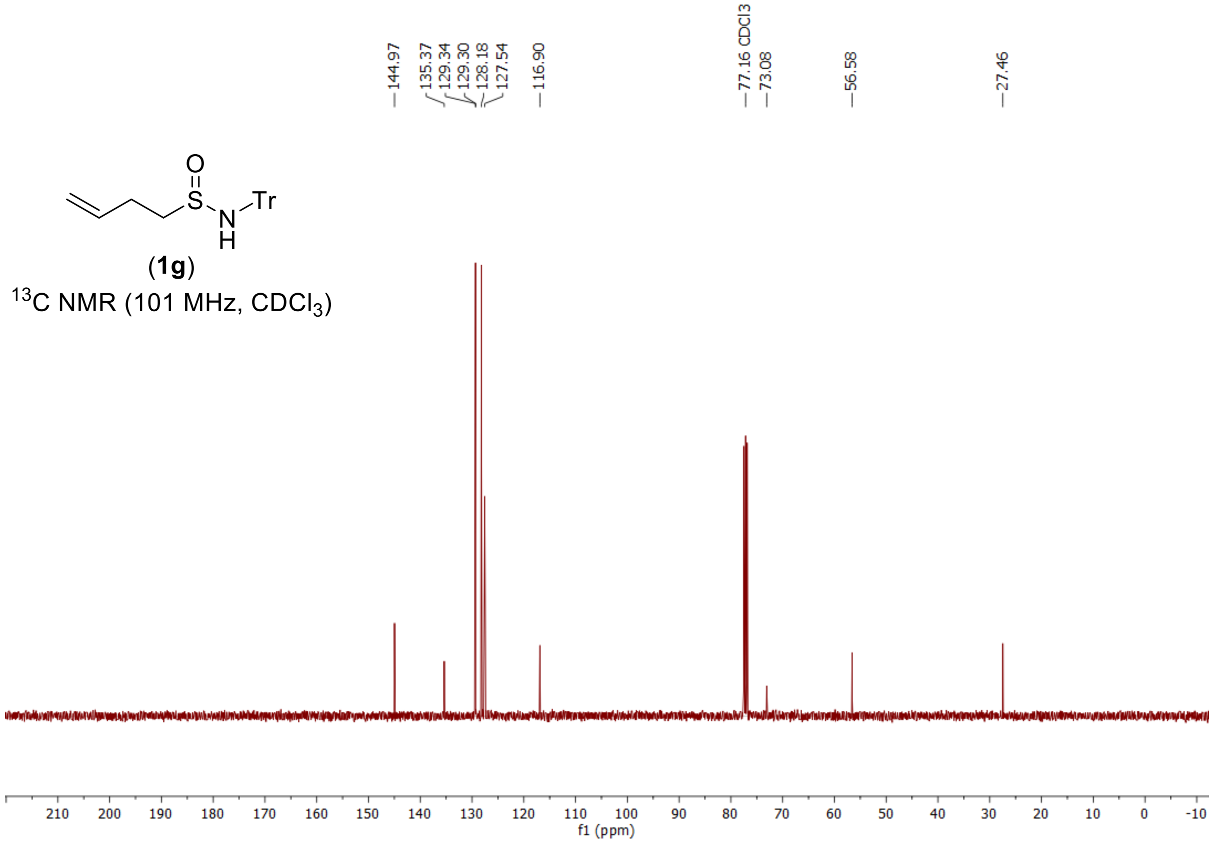
144.97 135.37 129.34 129.30 128.18 127.54 116.90 77.16 CDCl3 73.08 56.58 27.46
O
S
N
Tr
H
(1g)
13C NMR (101 MHz, CDCl3)
f1 (ppm)

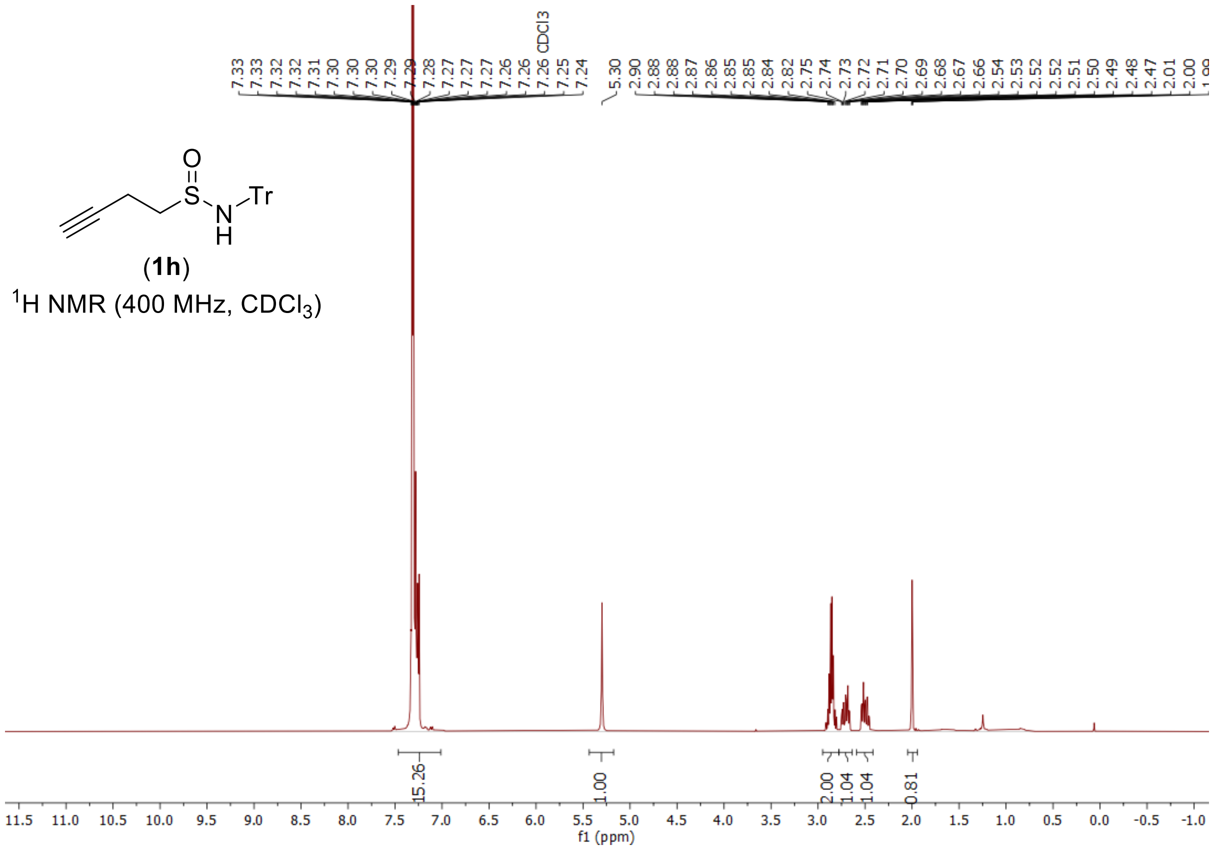

(**1h**)

^{1}H NMR (400 MHz, $CDCl_3$)

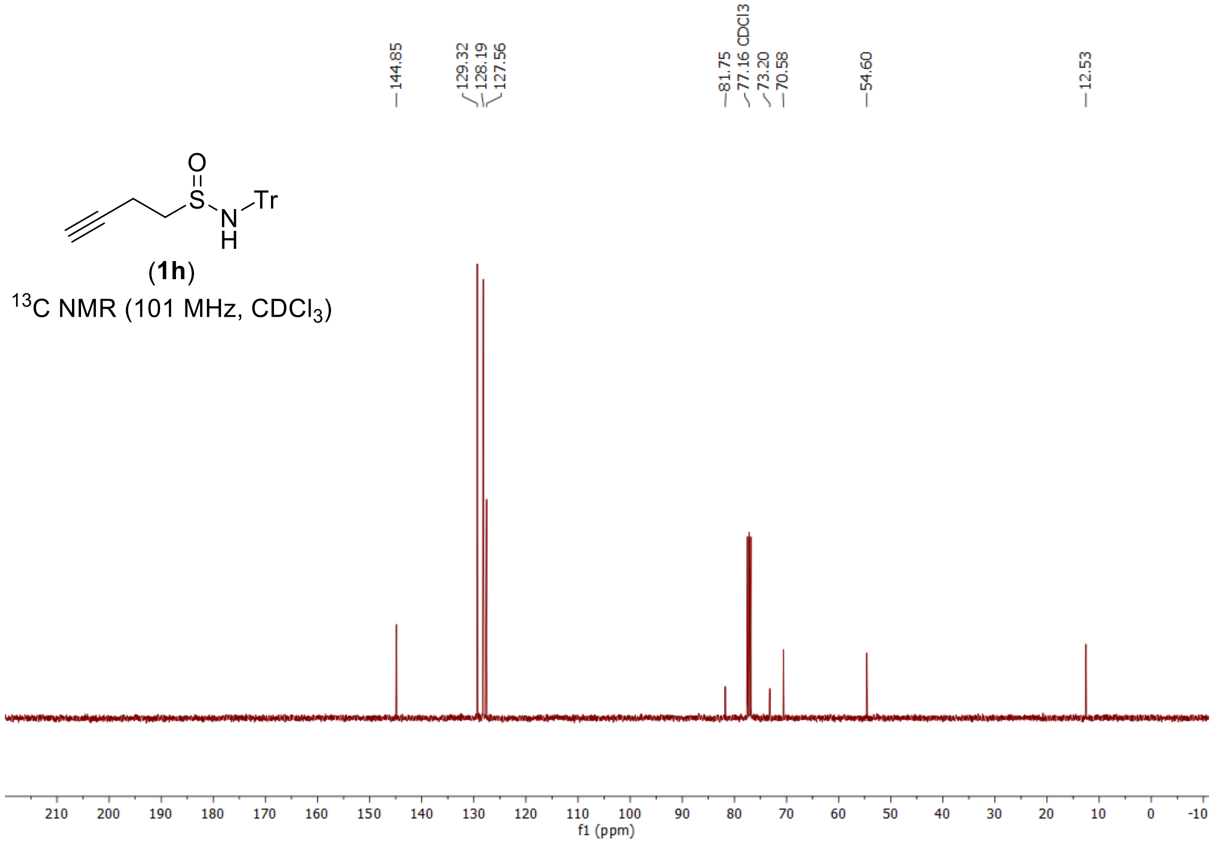

(**1h**)

^{13}C NMR (101 MHz, $CDCl_3$)

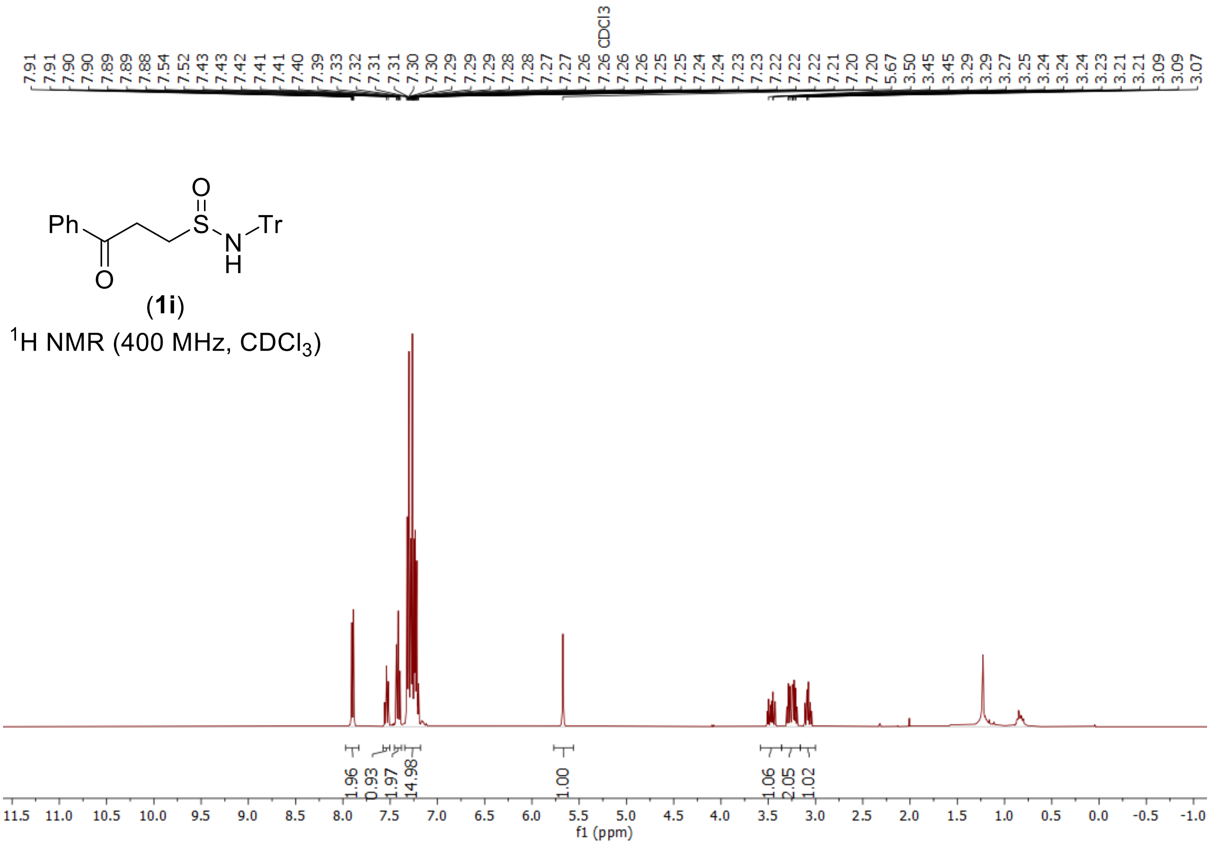
7.91 7.91 7.90 7.90 7.89 7.89 7.88 7.54 7.52 7.43 7.43 7.42 7.41 7.41 7.40 7.39 7.33 7.32 7.31 7.31 7.30 7.30 7.29 7.29 7.29 7.28 7.28 7.27 7.27 7.26 7.26 CDCl3 7.26 7.26 7.25 7.25 7.24 7.24 7.23 7.23 7.22 7.22 7.22 7.21 7.20 7.20 5.67 3.50 3.45 3.45 3.29 3.29 3.27 3.25 3.24 3.24 3.24 3.23 3.21 3.21 3.09 3.09 3.07
Ph
O
S
N
Tr
H
O
(1i)
1H NMR (400 MHz, CDCl3)
1.96
0.93
1.97
14.98
1.00
1.06
2.05
1.02
11.5 11.0 10.5 10.0 9.5 9.0 8.5 8.0 7.5 7.0 6.5 6.0 5.5 5.0 4.5 4.0 3.5 3.0 2.5 2.0 1.5 1.0 0.5 0.0 -0.5 -1.0
f1 (ppm)

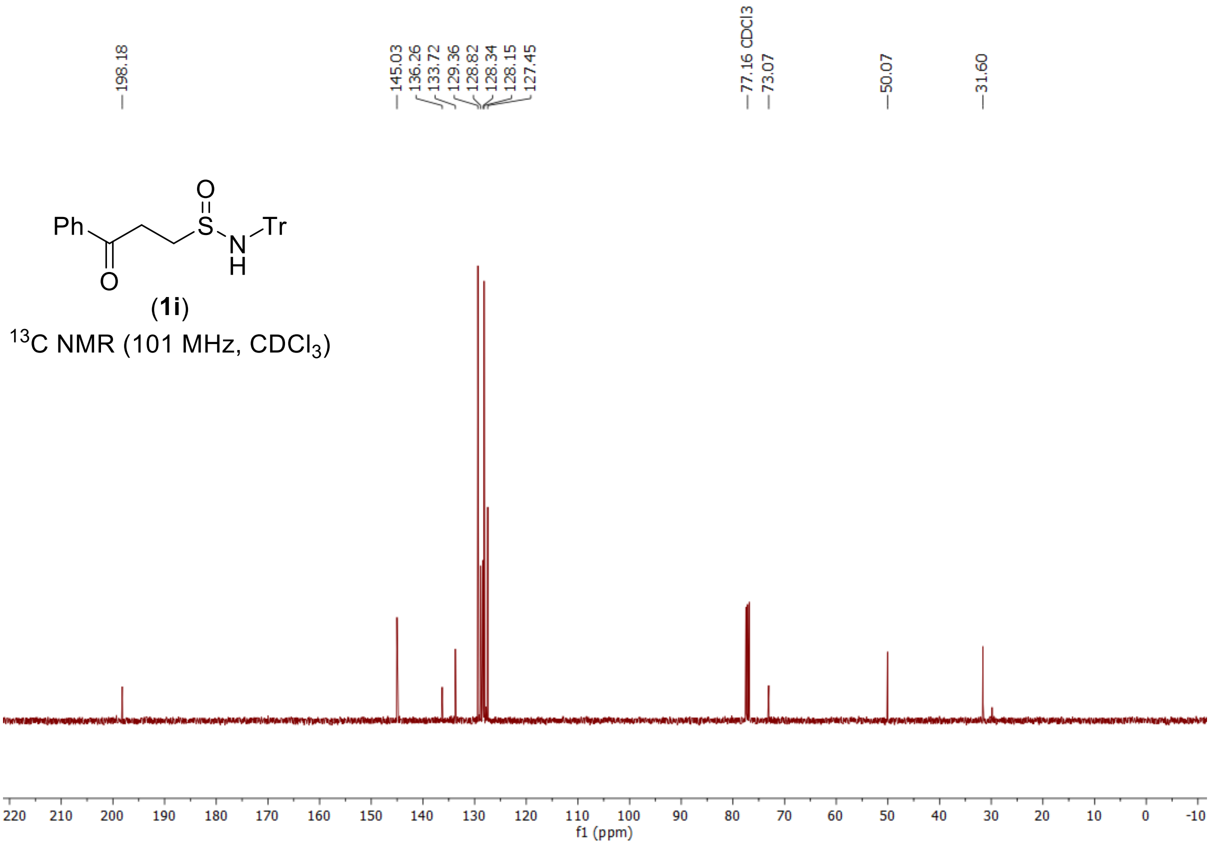
198.18
145.03 136.26 133.72 129.36 128.82 128.34 128.15 127.45
77.16 CDCl3
73.07
50.07
31.60
Ph
O
S
N
Tr
H
O
(1i)
13C NMR (101 MHz, CDCl3)
220 210 200 190 180 170 160 150 140 130 120 110 100 90 80 70 60 50 40 30 20 10 0 -10
f1 (ppm)

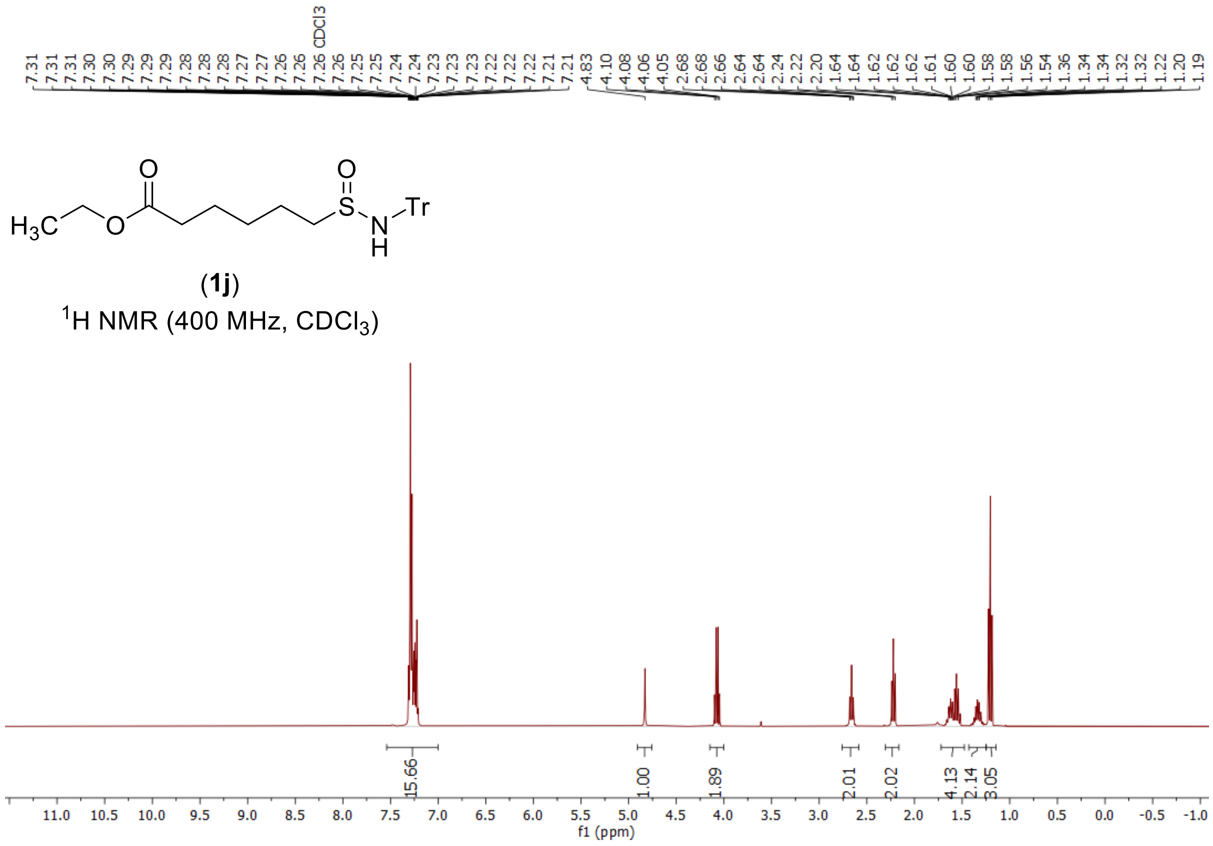

(**1j**)

^{1}H NMR (400 MHz, $CDCl_3$)

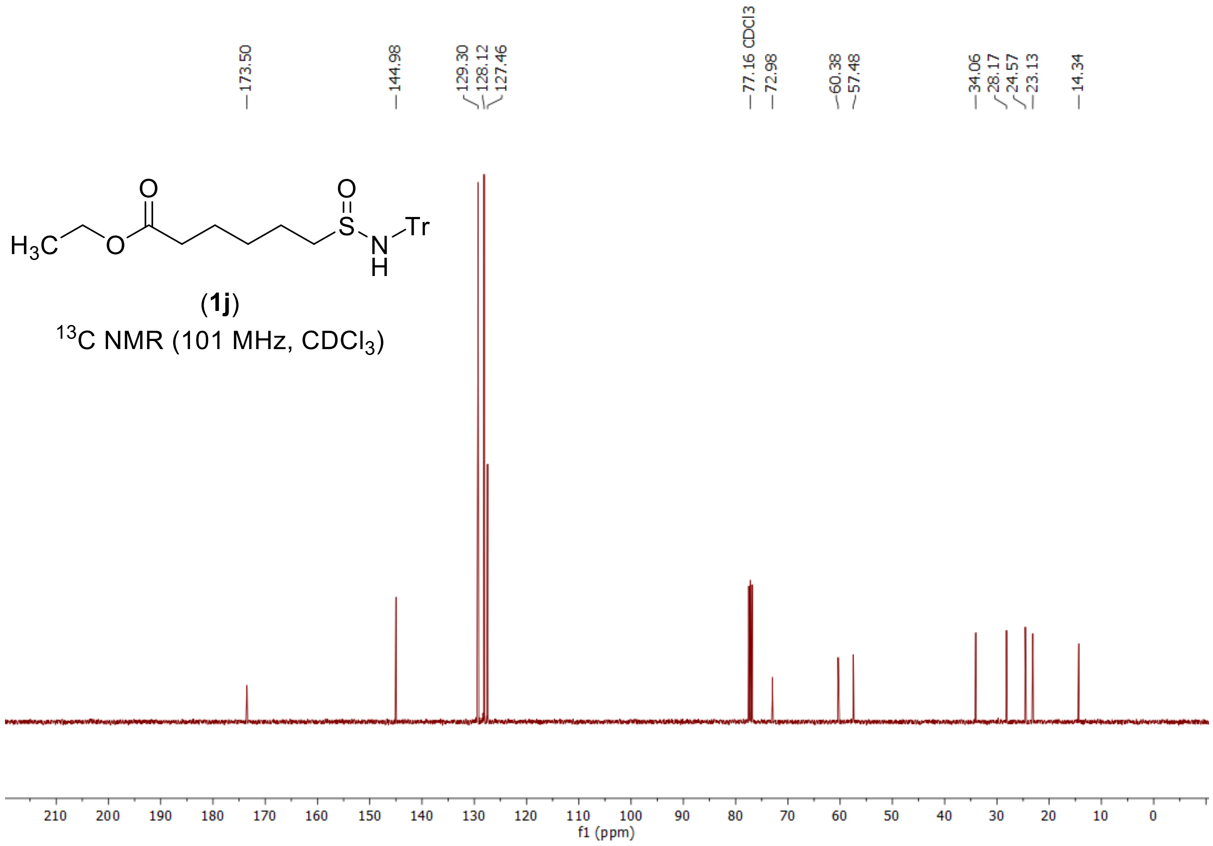

(**1j**)

^{13}C NMR (101 MHz, $CDCl_3$)

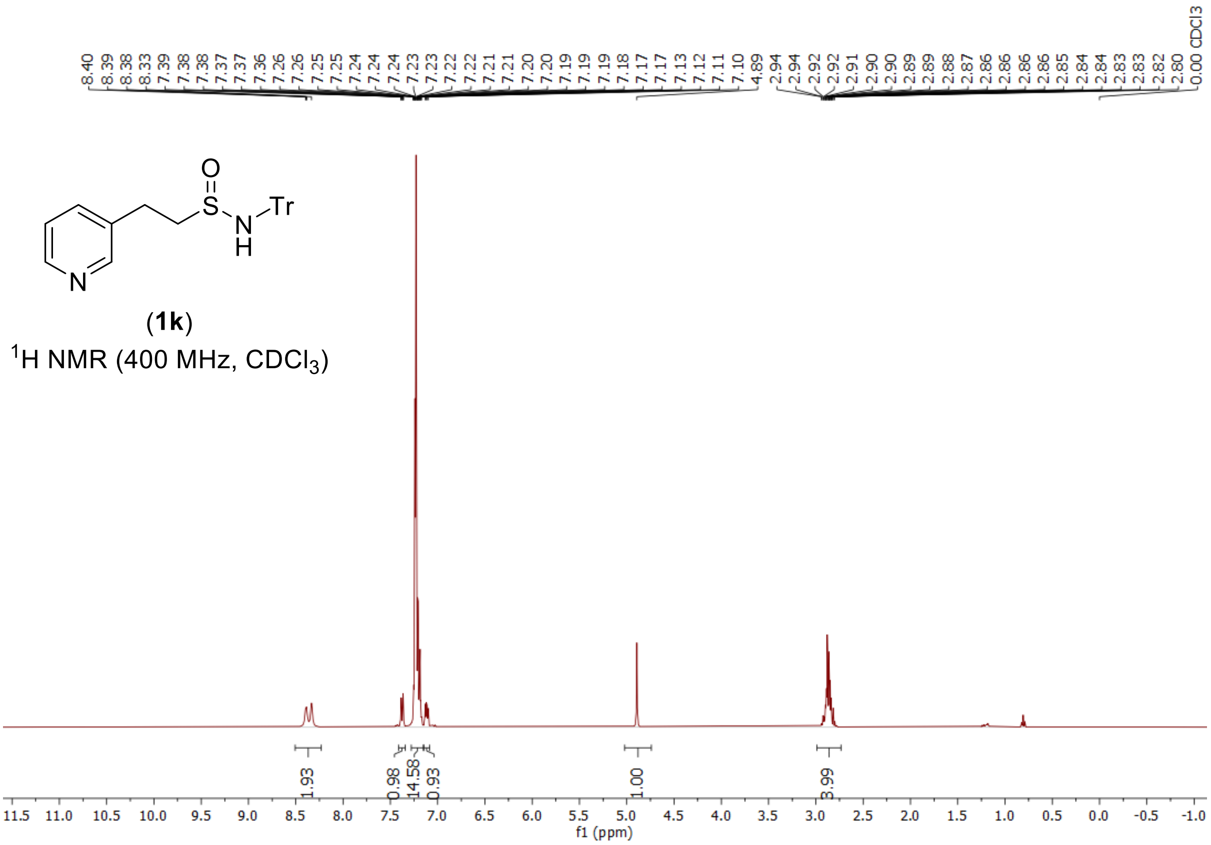

(1k)
^{1}H NMR (400 MHz, CDCl$_3$)
f1 (ppm)

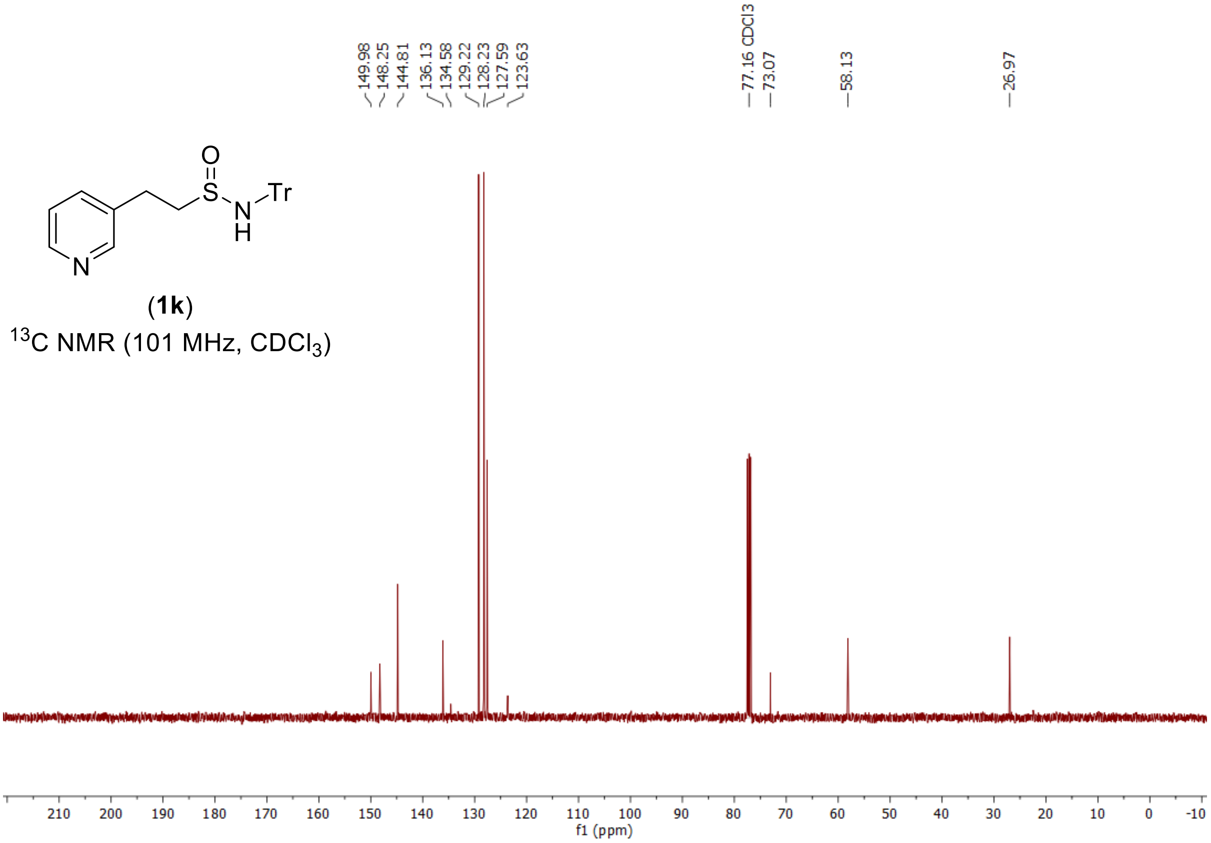

(1k)
^{13}C NMR (101 MHz, CDCl$_3$)
149.98
148.25
144.81
136.13
134.58
129.22
128.23
127.59
123.63
77.16 CDCl3
73.07
58.13
26.97
f1 (ppm)

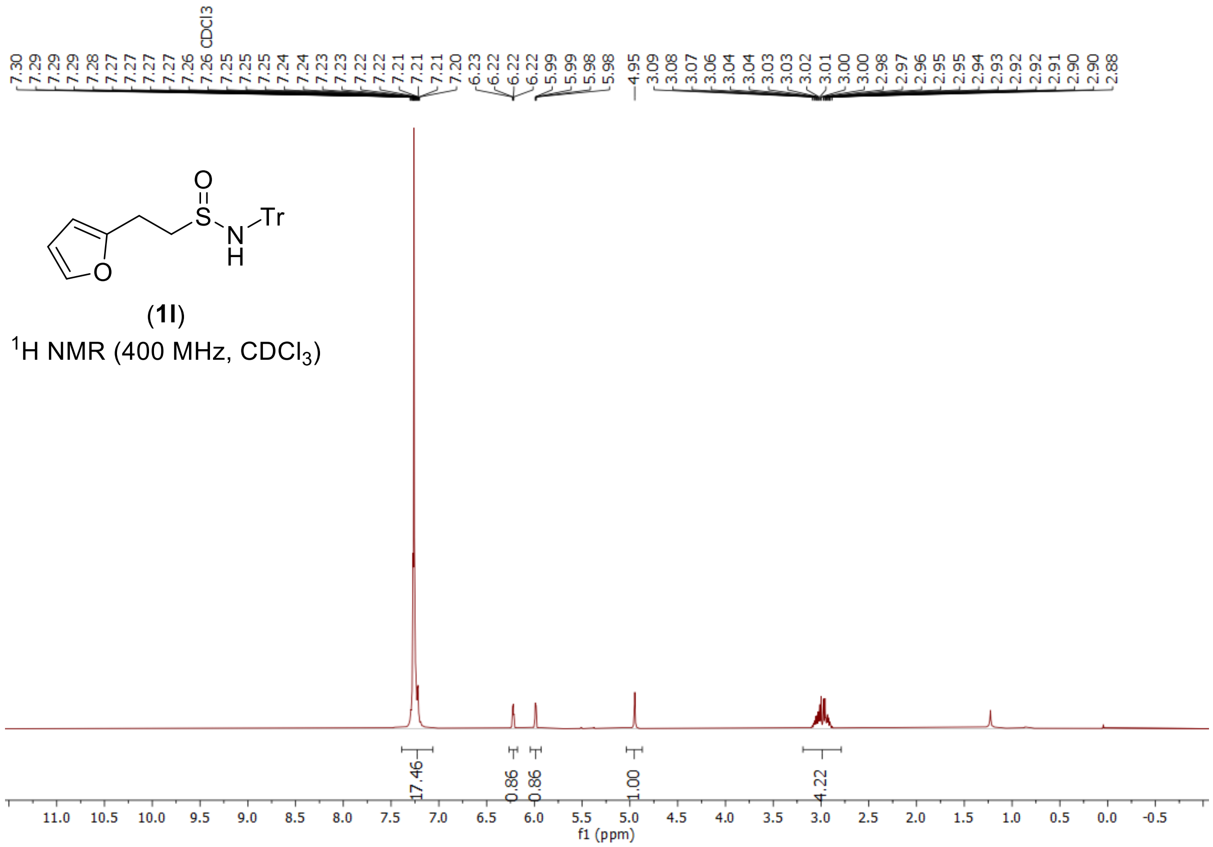

(**1l**)

^{1}H NMR (400 MHz, $CDCl_3$)

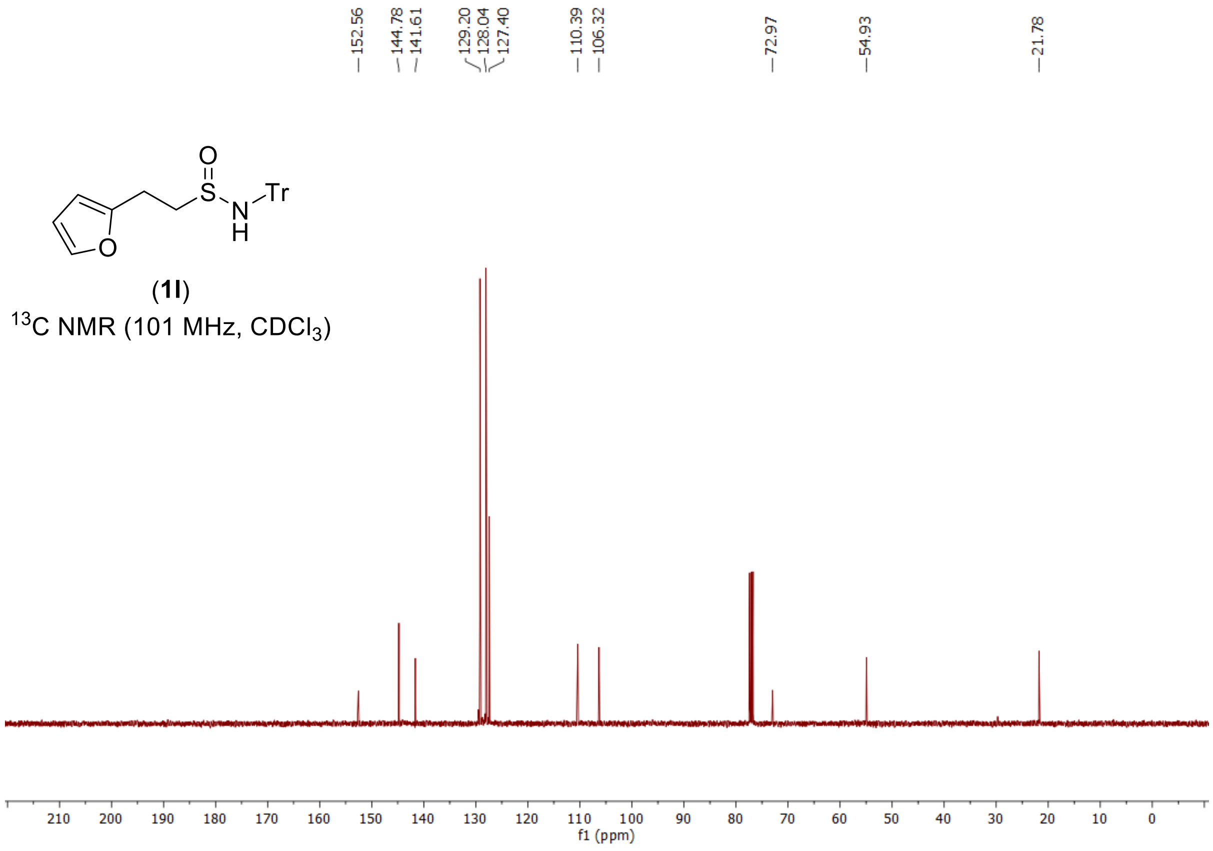

(**1l**)

^{13}C NMR (101 MHz, $CDCl_3$)

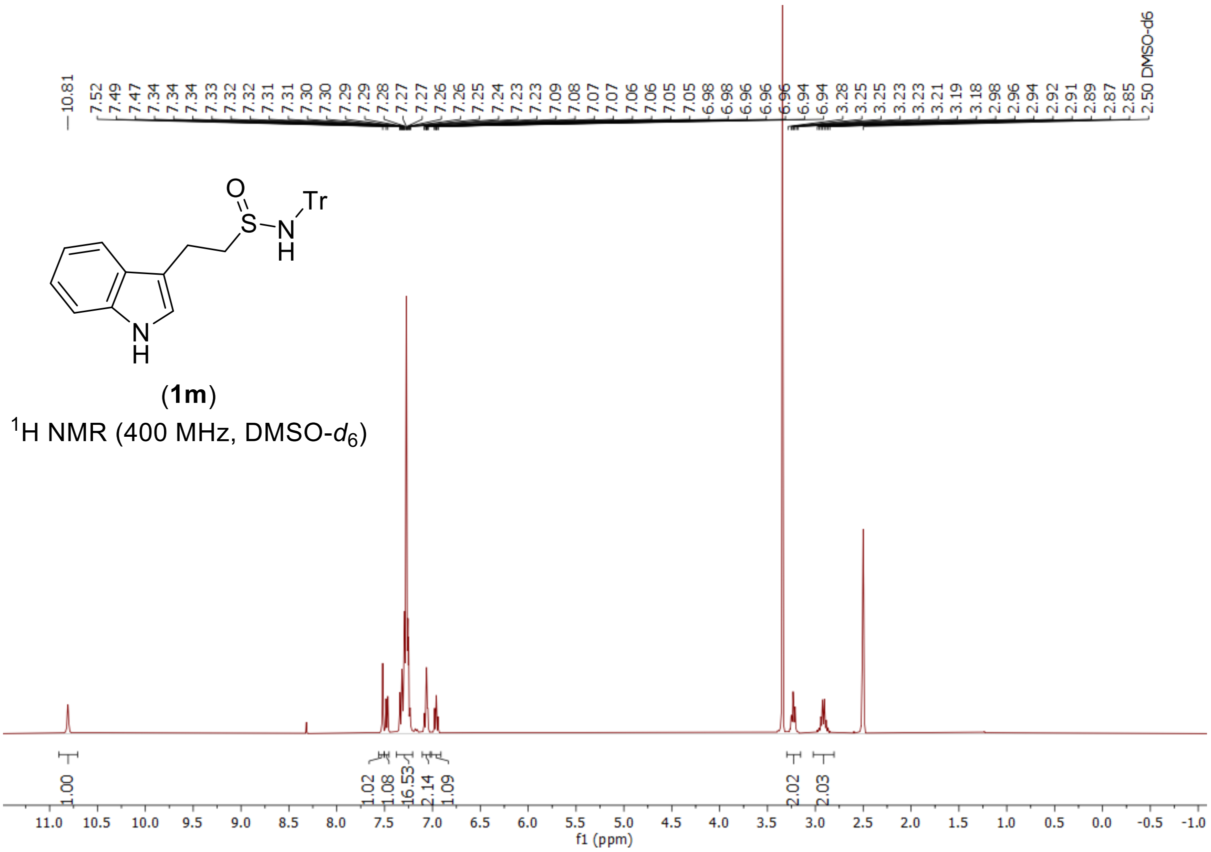

(1m)
1H NMR (400 MHz, DMSO-d6)
f1 (ppm)

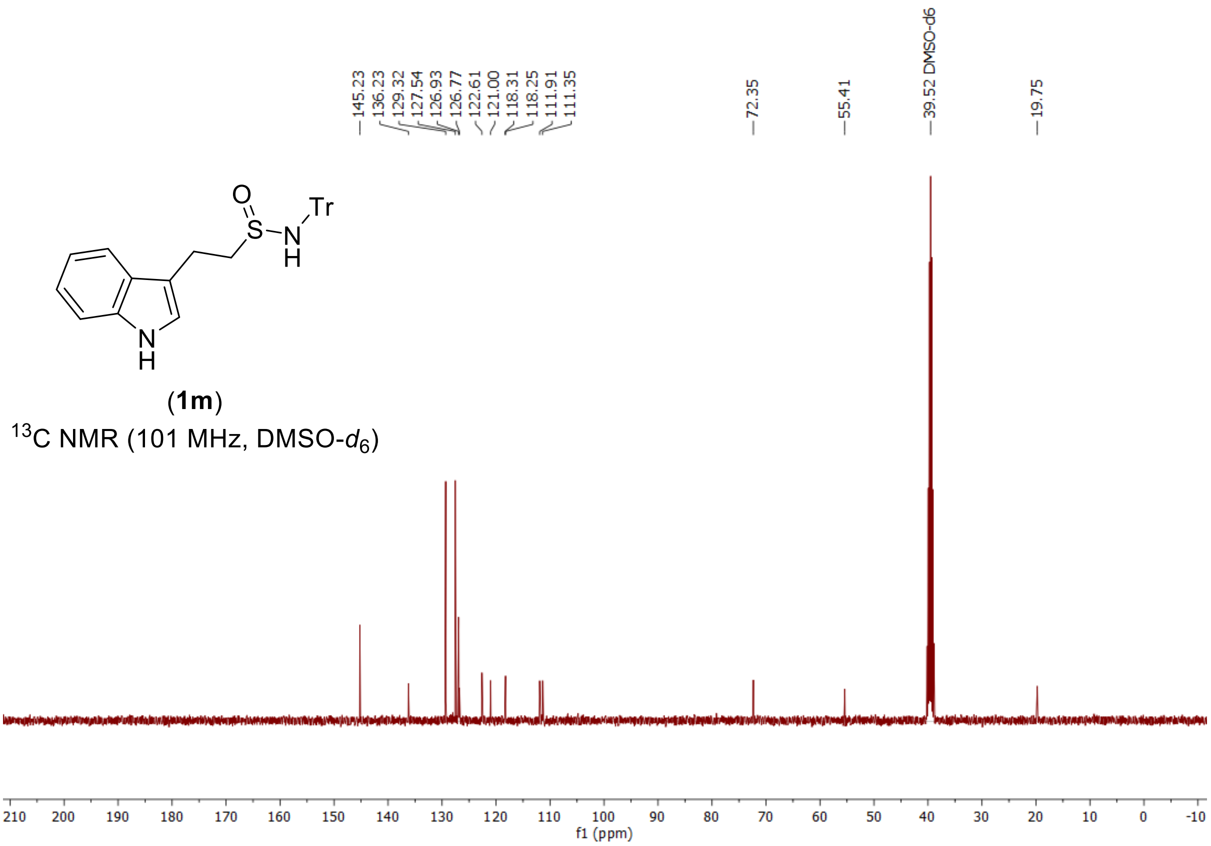

(1m)
13C NMR (101 MHz, DMSO-d6)
f1 (ppm)

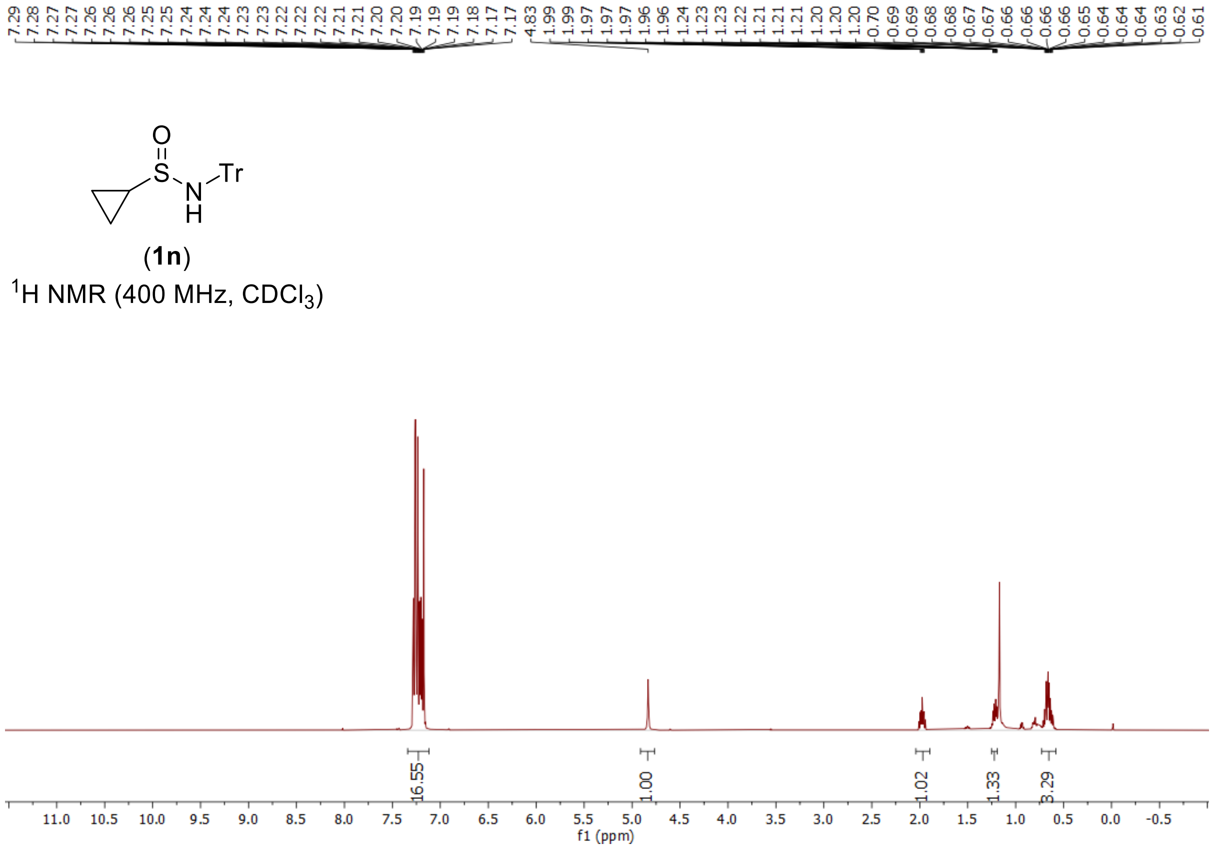
Tr
(1n)
1H NMR (400 MHz, CDCl3)
16.55
1.00
1.02
1.33
3.29
f1 (ppm)

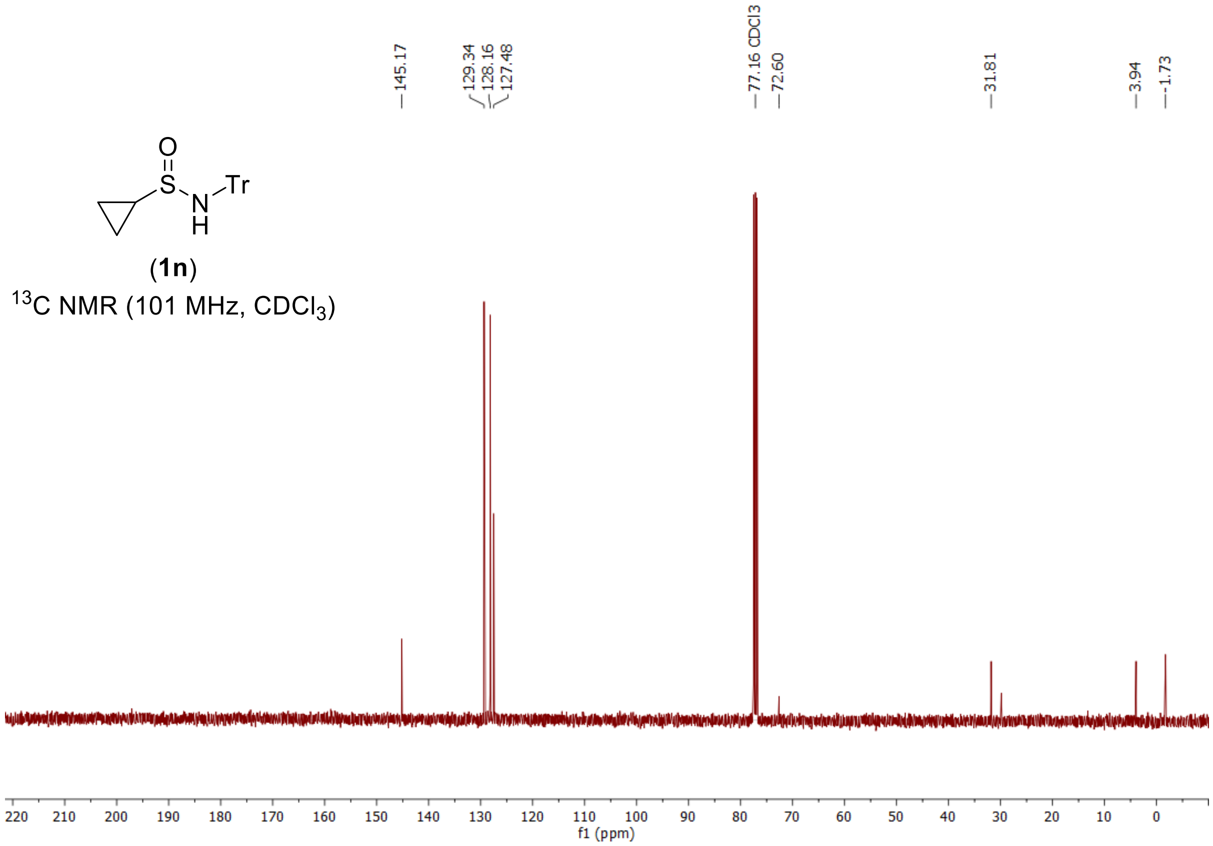
145.17
129.34
128.16
127.48
77.16 CDCl3
72.60
31.81
3.94
1.73
Tr
(1n)
13C NMR (101 MHz, CDCl3)
f1 (ppm)

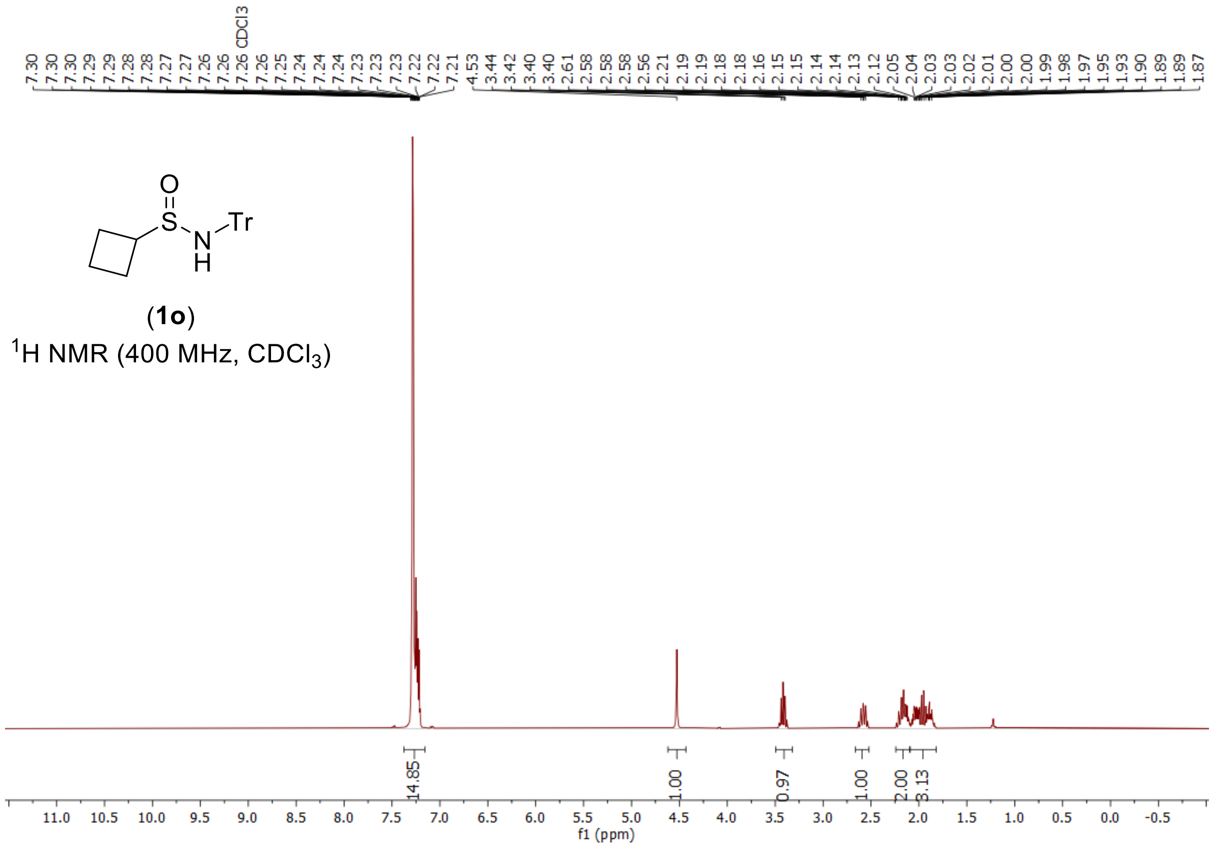

7.30 7.30 7.30 7.29 7.29 7.28 7.28 7.27 7.27 7.26 7.26 CDCl3 7.26 7.25 7.24 7.24 7.24 7.23 7.23 7.23 7.22 7.22 7.21
4.53 3.44 3.42 3.40 3.40 2.61 2.58 2.58 2.58 2.56 2.21 2.19 2.19 2.18 2.18 2.16 2.15 2.15 2.14 2.14 2.13 2.12 2.05 2.04 2.03 2.03 2.02 2.01 2.00 2.00 1.99 1.98 1.97 1.95 1.93 1.90 1.89 1.89 1.87
Tr
(1o)
1H NMR (400 MHz, CDCl3)
14.85
1.00
0.97
1.00
2.00
3.13
f1 (ppm)

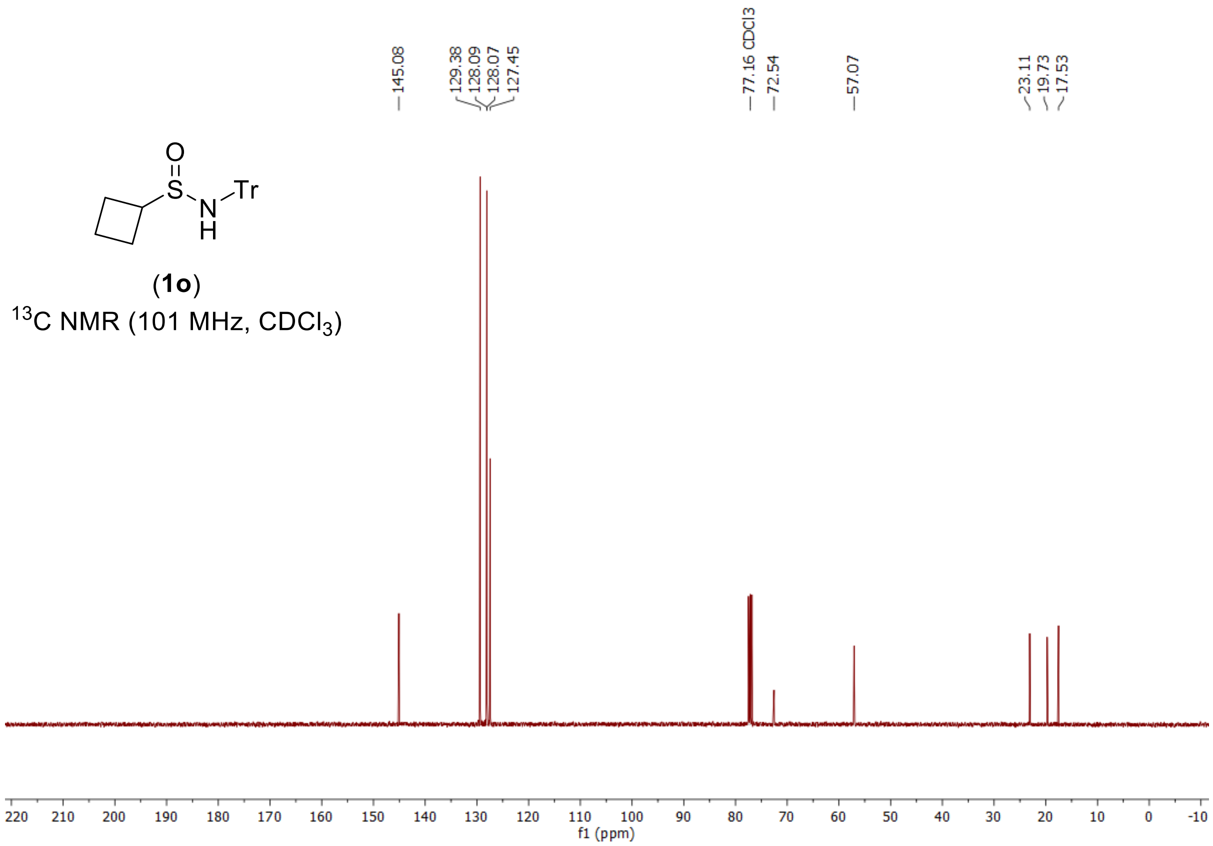

145.08
129.38 128.09 128.07 127.45
77.16 CDCl3
72.54
57.07
23.11 19.73 17.53
Tr
(1o)
13C NMR (101 MHz, CDCl3)
f1 (ppm)

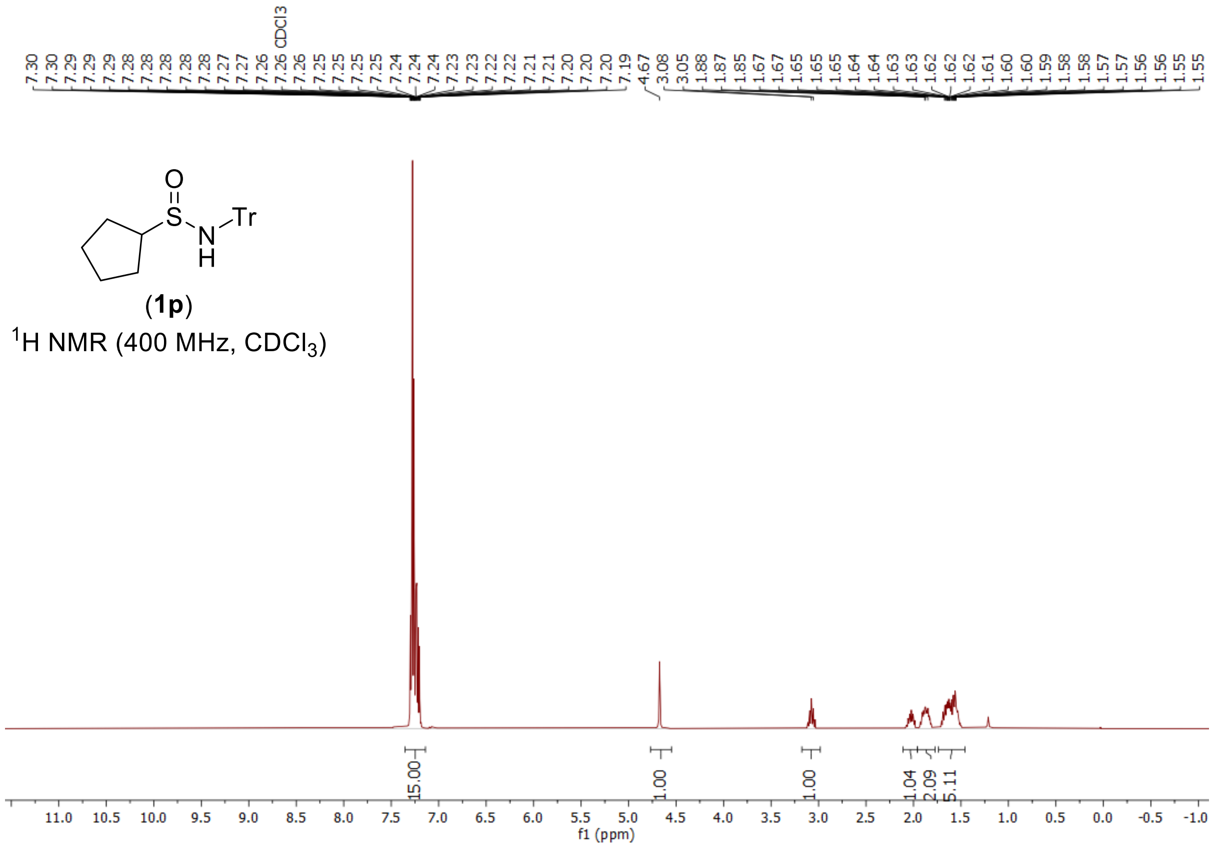

7.30
7.30
7.29
7.29
7.28
7.28
7.28
7.28
7.28
7.27
7.26
7.26 CDCl3
7.26
7.25
7.25
7.25
7.25
7.24
7.24
7.24
7.23
7.23
7.22
7.22
7.21
7.21
7.20
7.20
7.19
4.67
3.08
3.05
1.88
1.87
1.85
1.67
1.67
1.65
1.65
1.65
1.64
1.63
1.63
1.62
1.62
1.62
1.61
1.60
1.60
1.59
1.58
1.58
1.57
1.57
1.56
1.56
1.55
1.55
O
S
N
Tr
H
(1p)
1H NMR (400 MHz, CDCl3)
15.00
1.00
1.00
1.04
2.09
5.11
11.0 10.5 10.0 9.5 9.0 8.5 8.0 7.5 7.0 6.5 6.0 5.5 5.0 4.5 4.0 3.5 3.0 2.5 2.0 1.5 1.0 0.5 0.0 -0.5 -1.0
f1 (ppm)

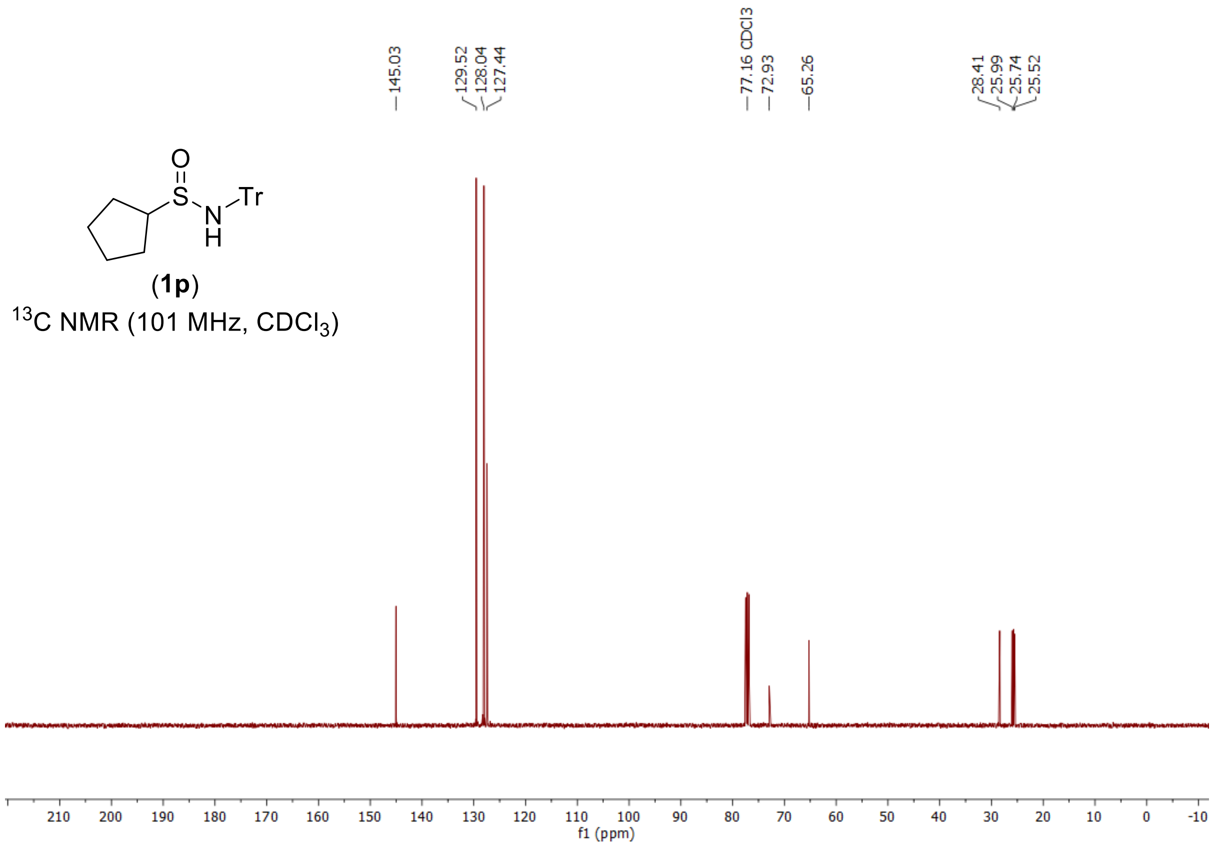

145.03
129.52
128.04
127.44
77.16 CDCl3
72.93
65.26
28.41
25.99
25.74
25.52
O
S
N
Tr
H
(1p)
13C NMR (101 MHz, CDCl3)
210 200 190 180 170 160 150 140 130 120 110 100 90 80 70 60 50 40 30 20 10 0 -10
f1 (ppm)

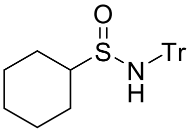

(**1q**)

^{1}H NMR (400 MHz, $CDCl_3$)

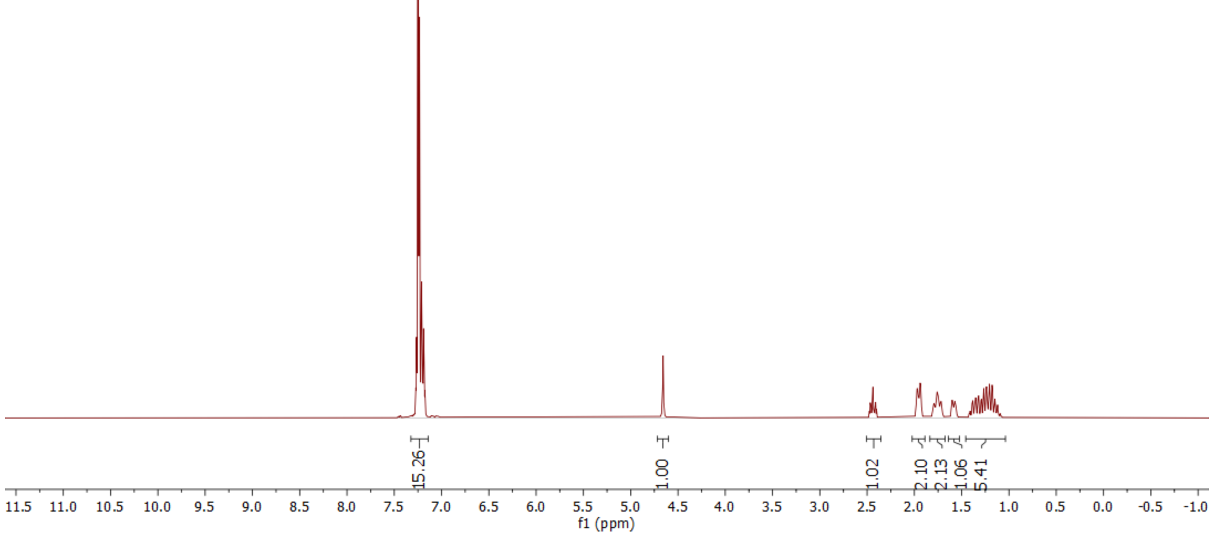

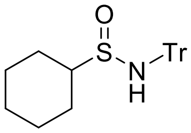

(**1q**)

^{13}C NMR (101 MHz, $CDCl_3$)

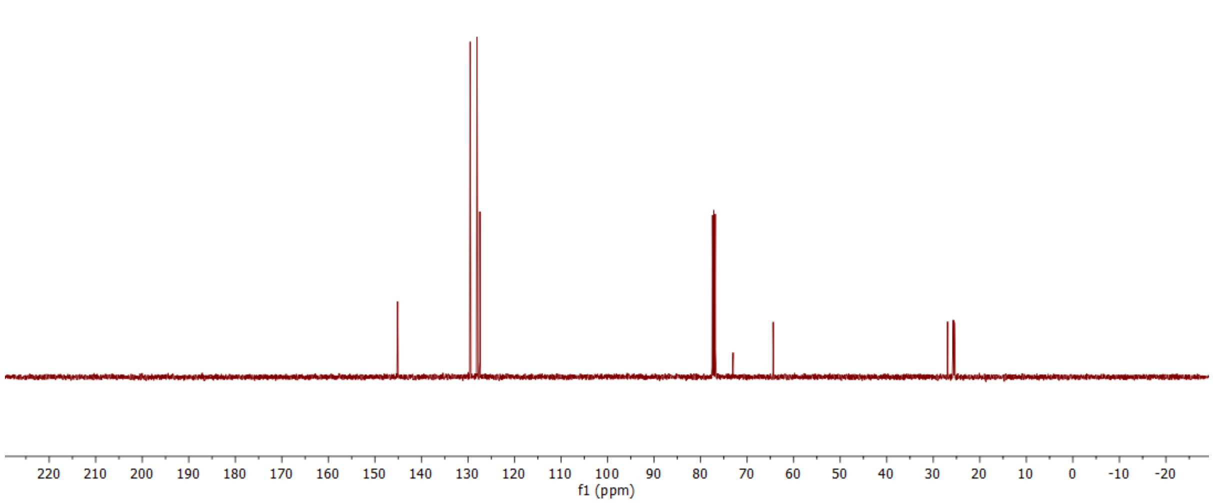

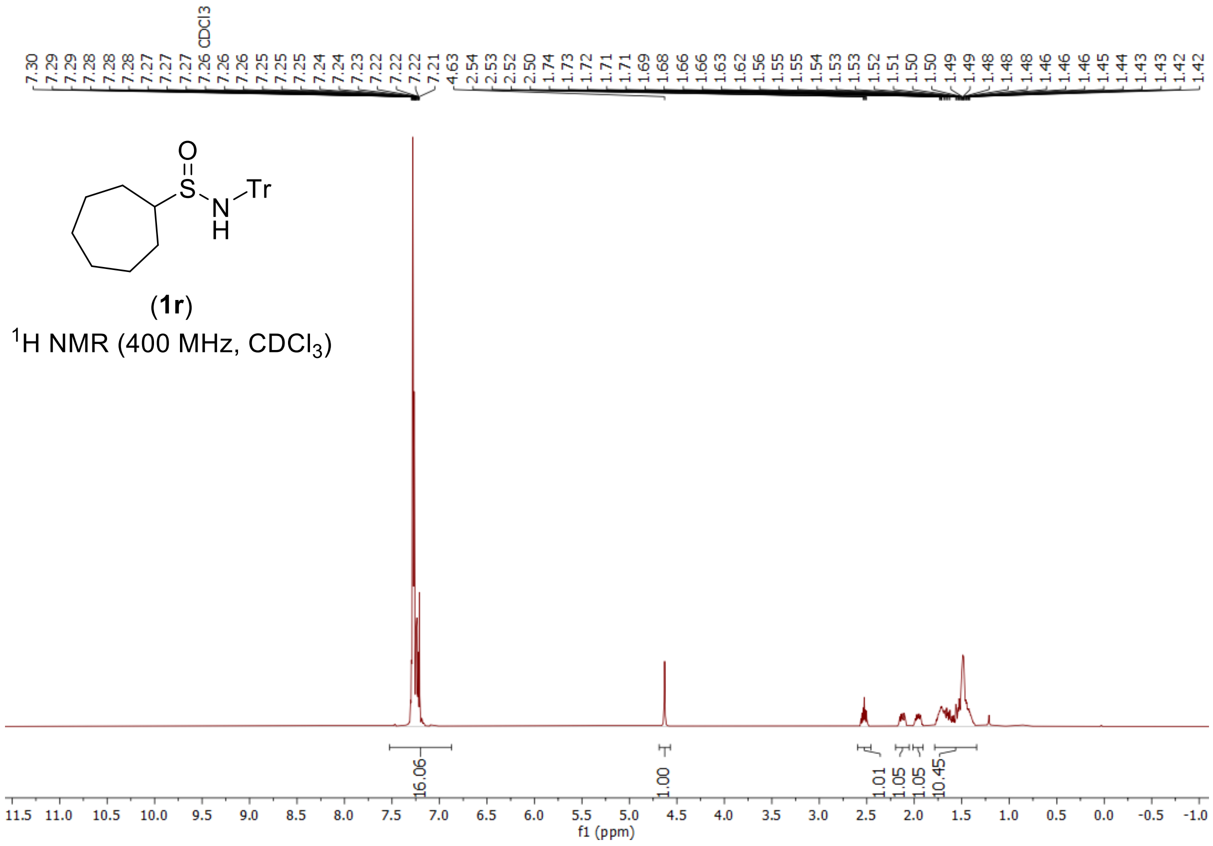

(**1r**)

^{1}H NMR (400 MHz, $CDCl_3$)

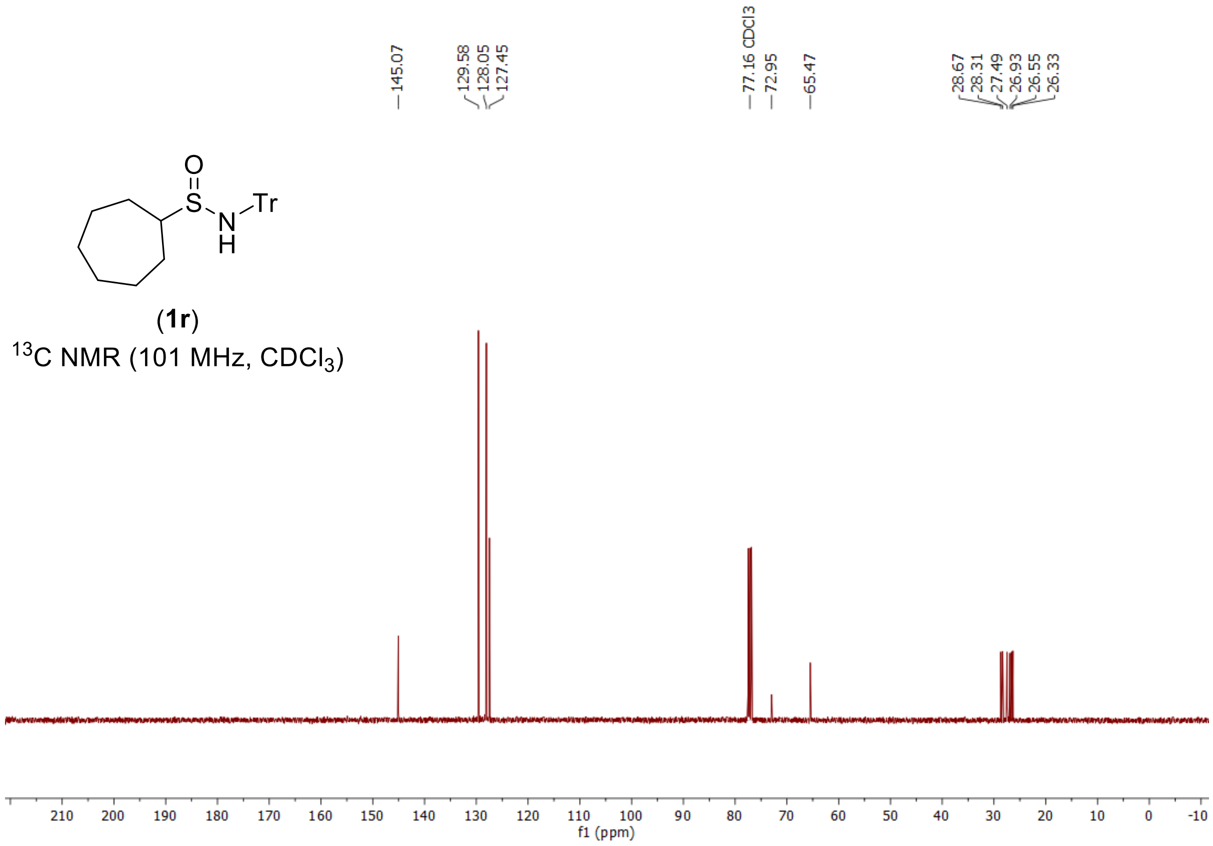

(**1r**)

^{13}C NMR (101 MHz, $CDCl_3$)

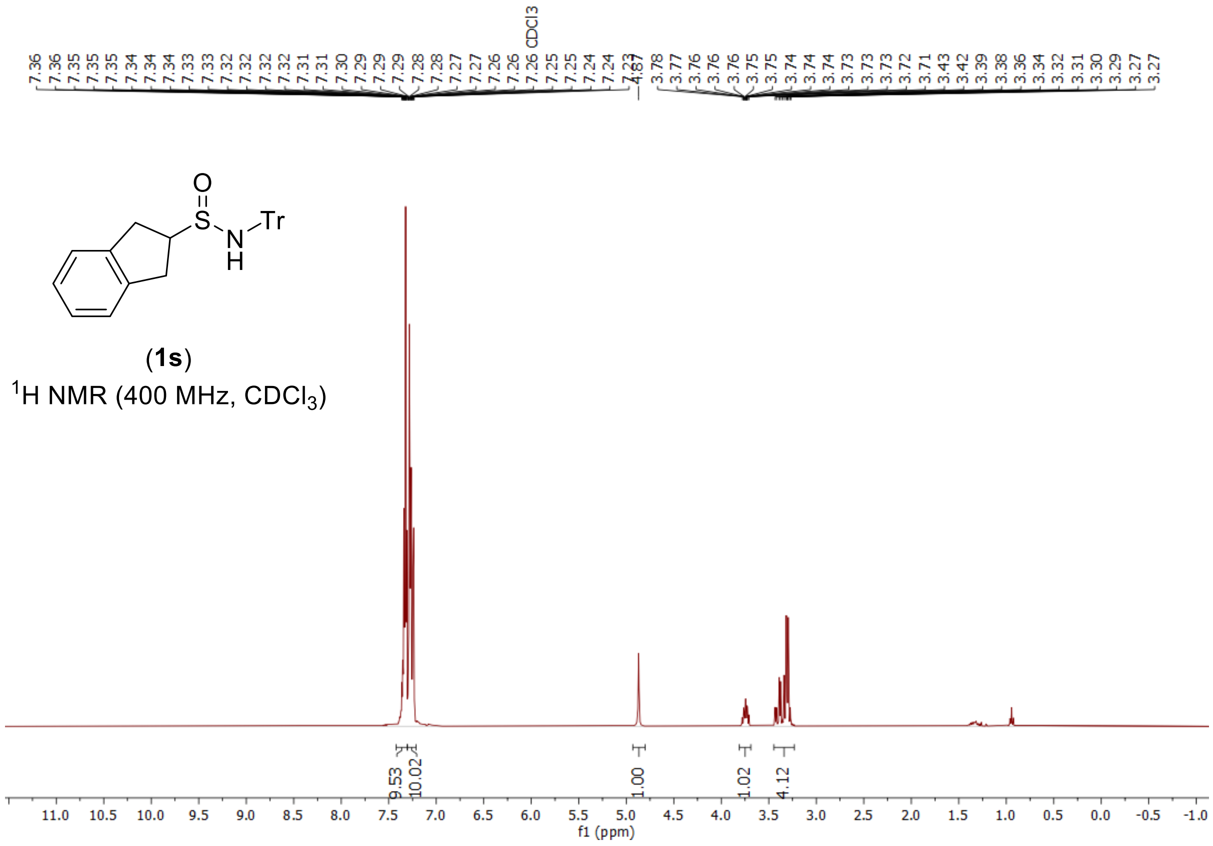
CDCl3
(1s)
1H NMR (400 MHz, CDCl3)
9.53
10.02
1.00
1.02
4.12
f1 (ppm)

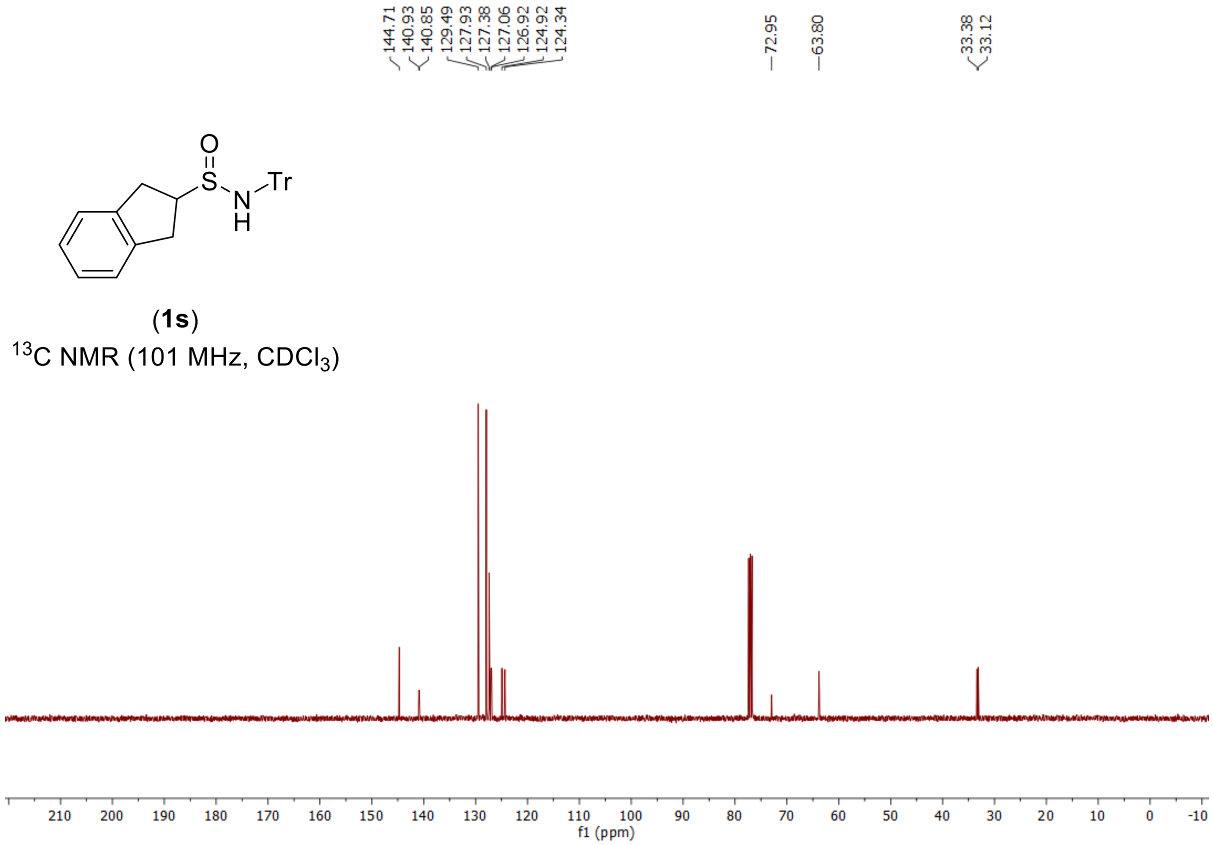
144.71
140.93
140.85
129.49
127.93
127.38
127.06
126.92
124.92
124.34
72.95
63.80
33.38
33.12
(1s)
13C NMR (101 MHz, CDCl3)
f1 (ppm)

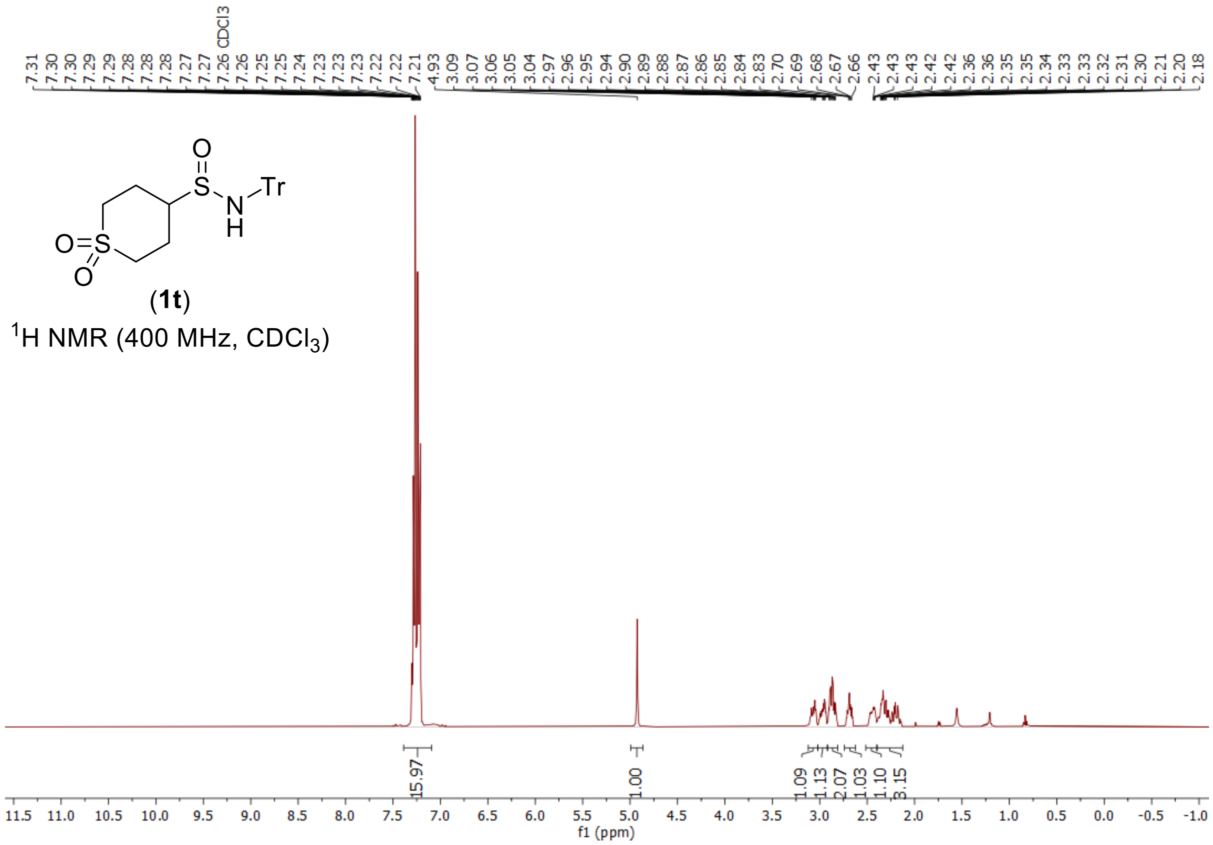

(**1t**)

^{1}H NMR (400 MHz, $CDCl_3$)

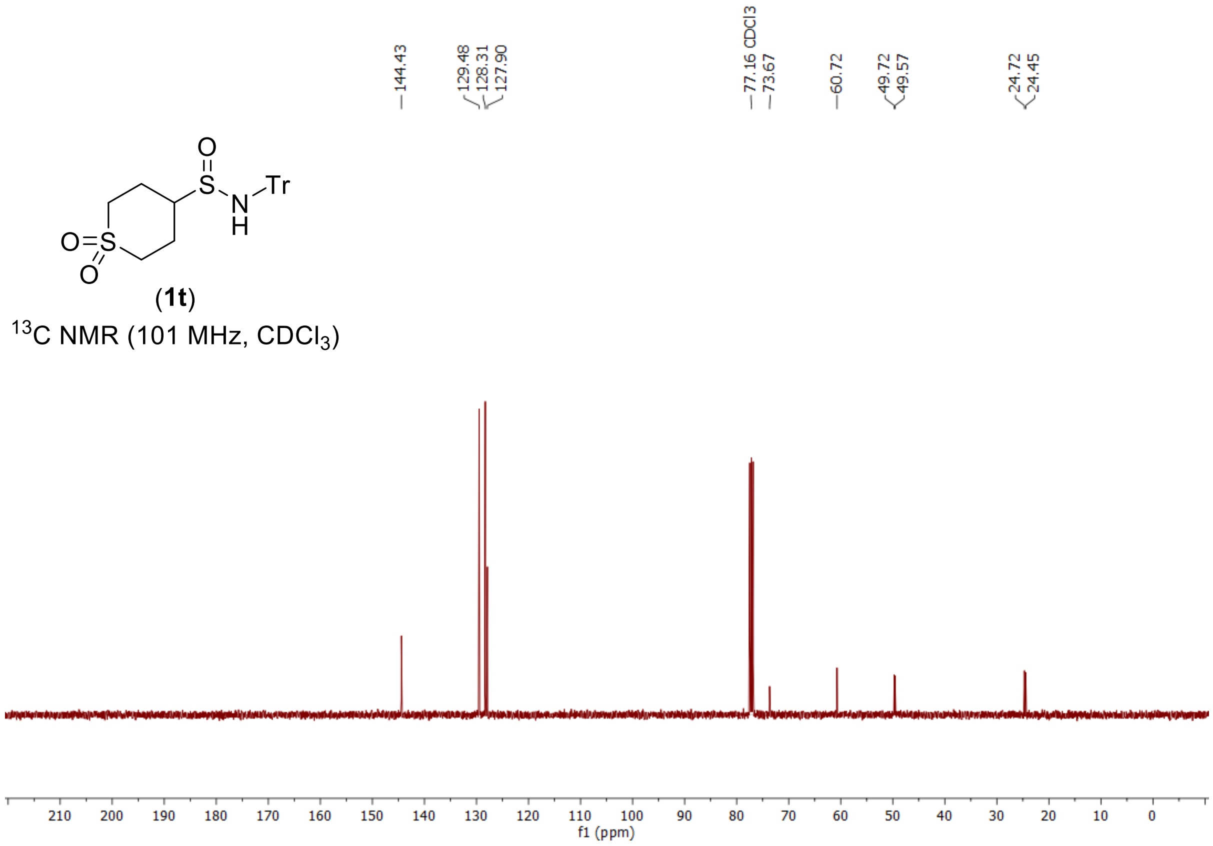

(**1t**)

^{13}C NMR (101 MHz, $CDCl_3$)

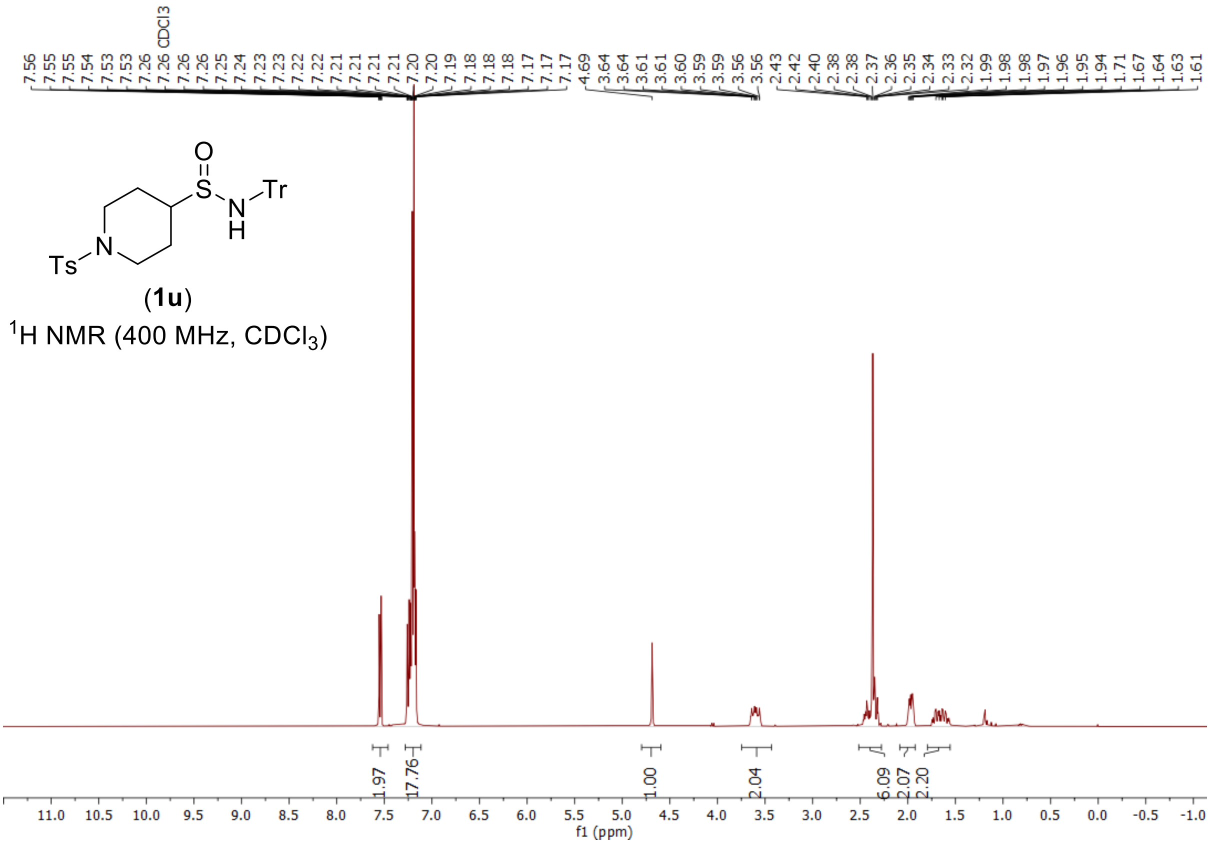

(1u)
^{1}H NMR (400 MHz, CDCl$_3$)
f1 (ppm)

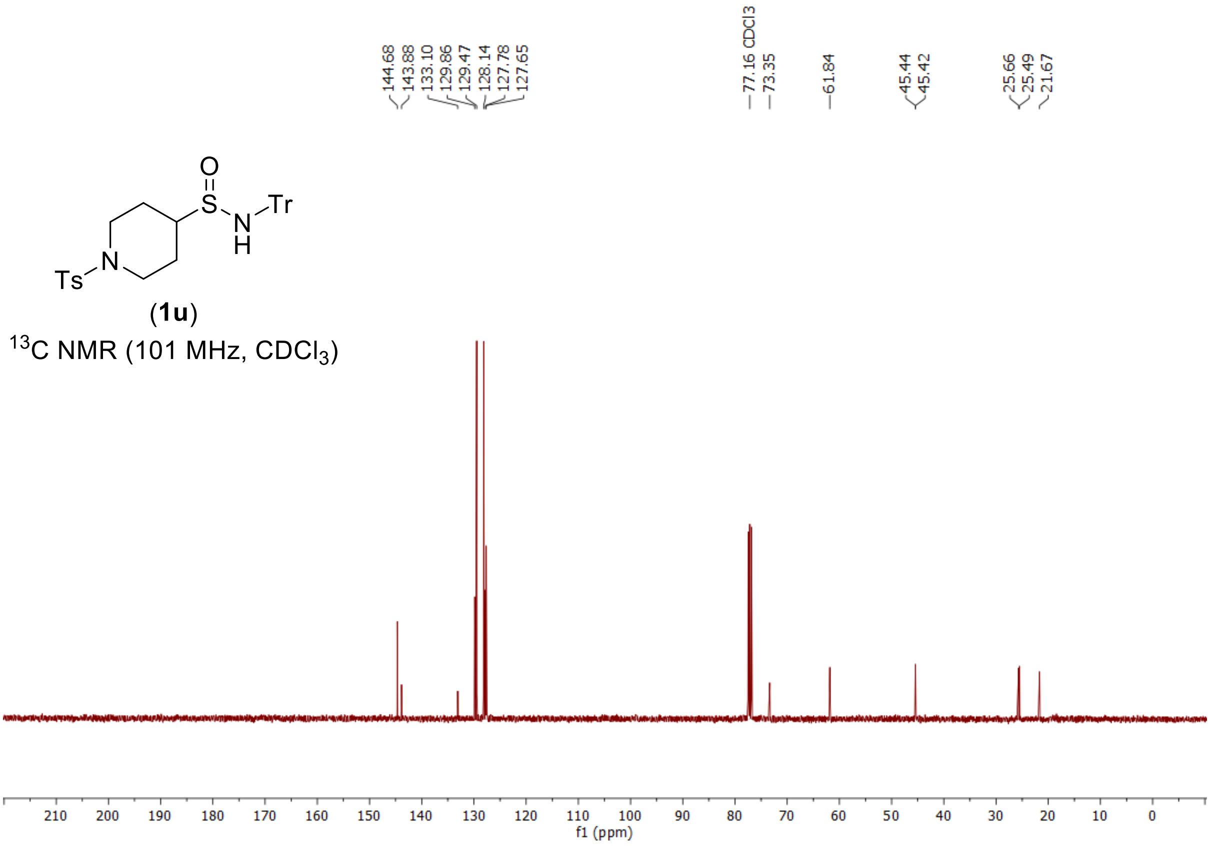

(1u)
^{13}C NMR (101 MHz, CDCl$_3$)
f1 (ppm)

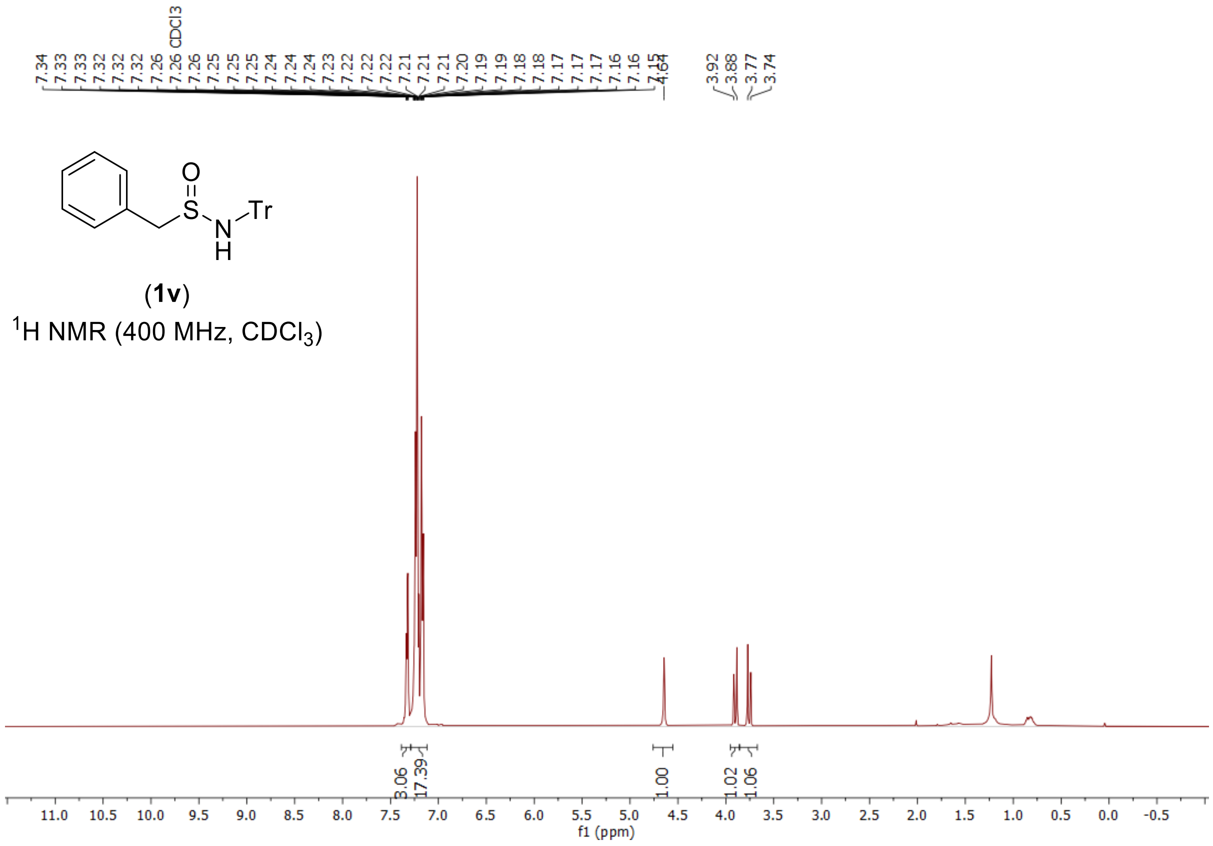
7.34 7.33 7.33 7.32 7.32 7.32 7.26 7.26 CDCl3 7.26 7.25 7.25 7.25 7.24 7.24 7.24 7.23 7.22 7.22 7.22 7.21 7.21 7.21 7.20 7.19 7.19 7.18 7.18 7.17 7.17 7.17 7.16 7.16 7.15 4.64
3.92 3.88 3.77 3.74
O
S
N
Tr
H
(1v)
1H NMR (400 MHz, CDCl3)
3.06
17.39
1.00
1.02
1.06
11.0 10.5 10.0 9.5 9.0 8.5 8.0 7.5 7.0 6.5 6.0 5.5 5.0 4.5 4.0 3.5 3.0 2.5 2.0 1.5 1.0 0.5 0.0 -0.5
f1 (ppm)

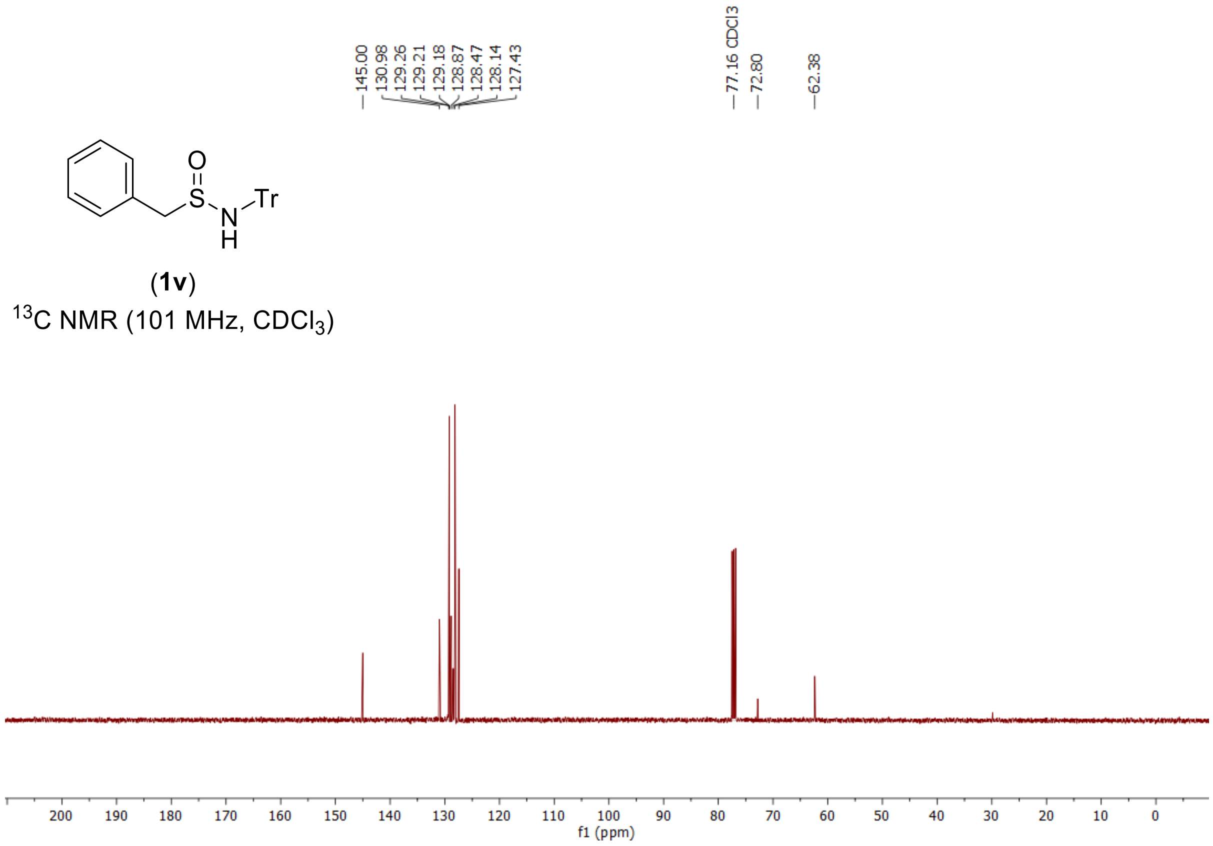
145.00
130.98 129.26 129.21 129.18 128.87 128.47 128.14 127.43
77.16 CDCl3
72.80
62.38
O
S
N
Tr
H
(1v)
13C NMR (101 MHz, CDCl3)
200 190 180 170 160 150 140 130 120 110 100 90 80 70 60 50 40 30 20 10 0
f1 (ppm)

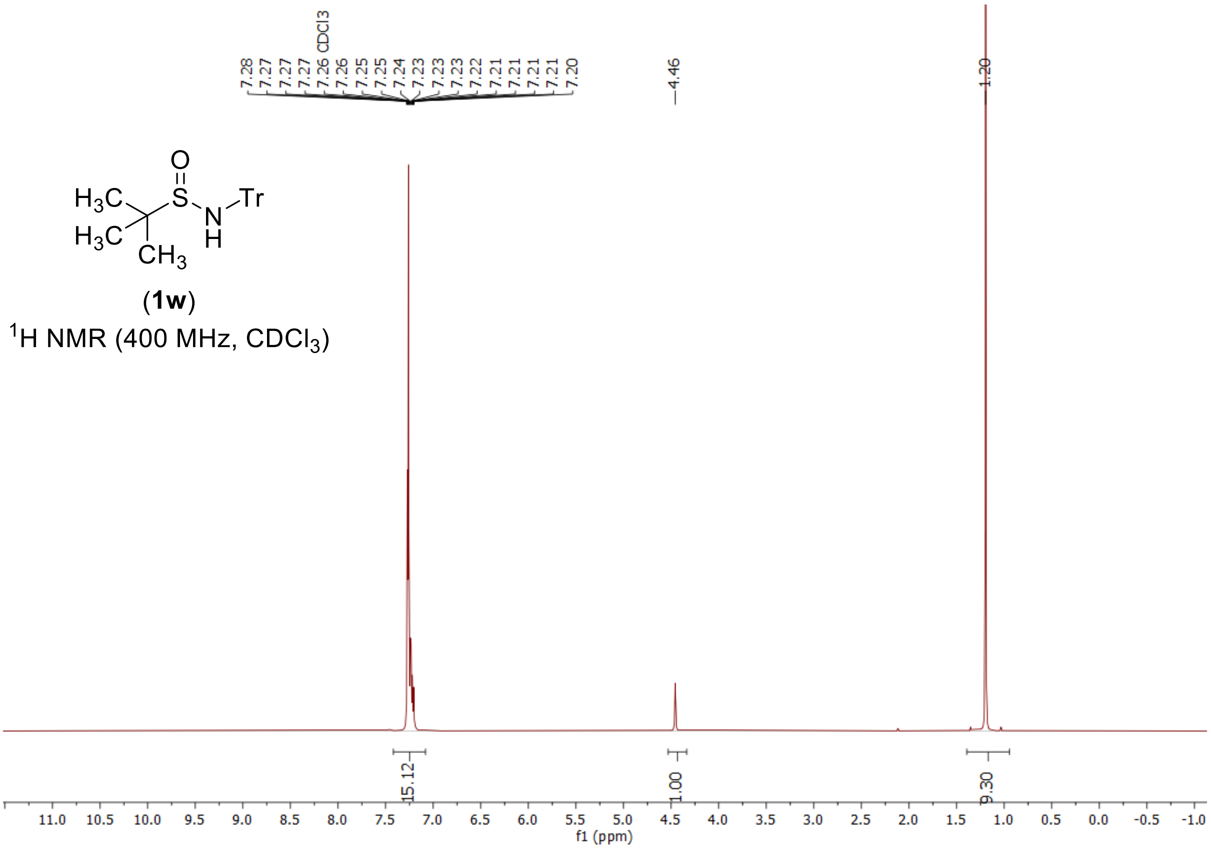

(**1w**)

^{1}H NMR (400 MHz, $CDCl_3$)

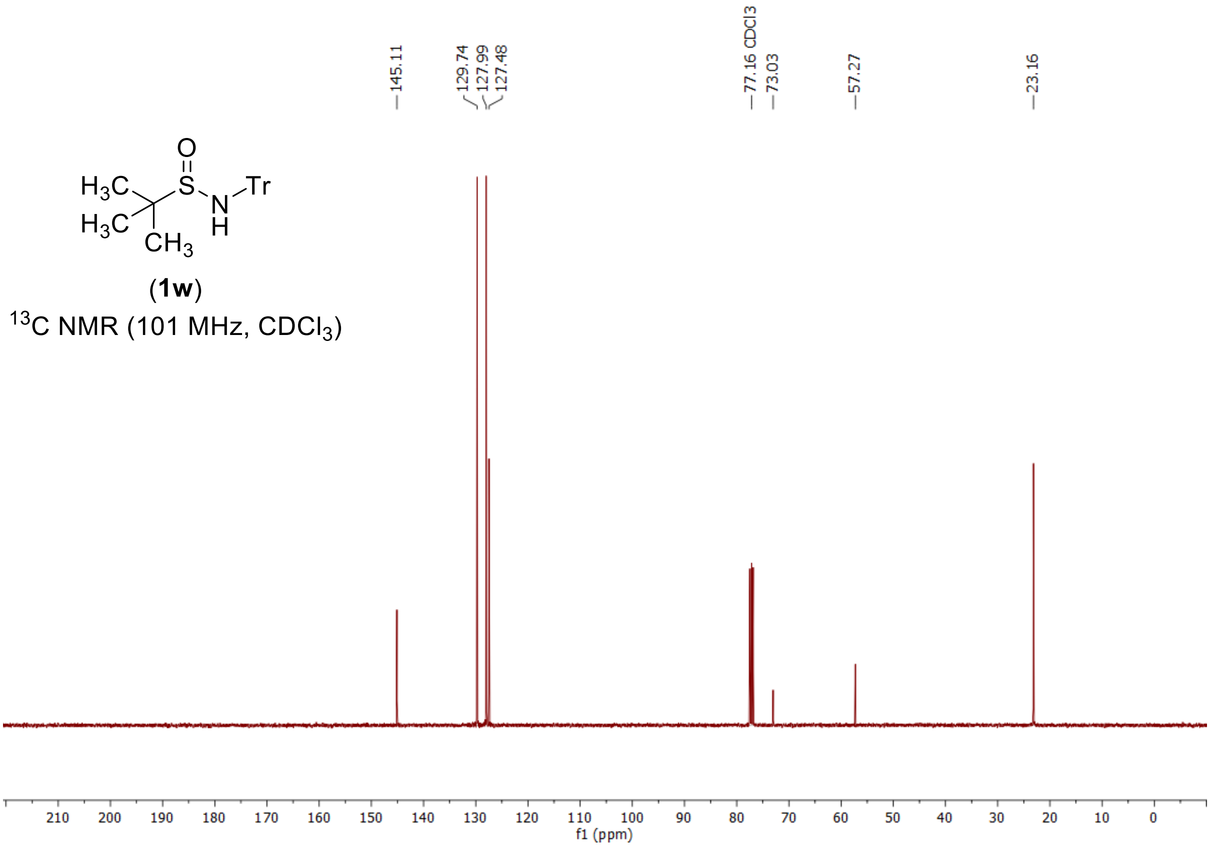

(**1w**)

^{13}C NMR (101 MHz, $CDCl_3$)

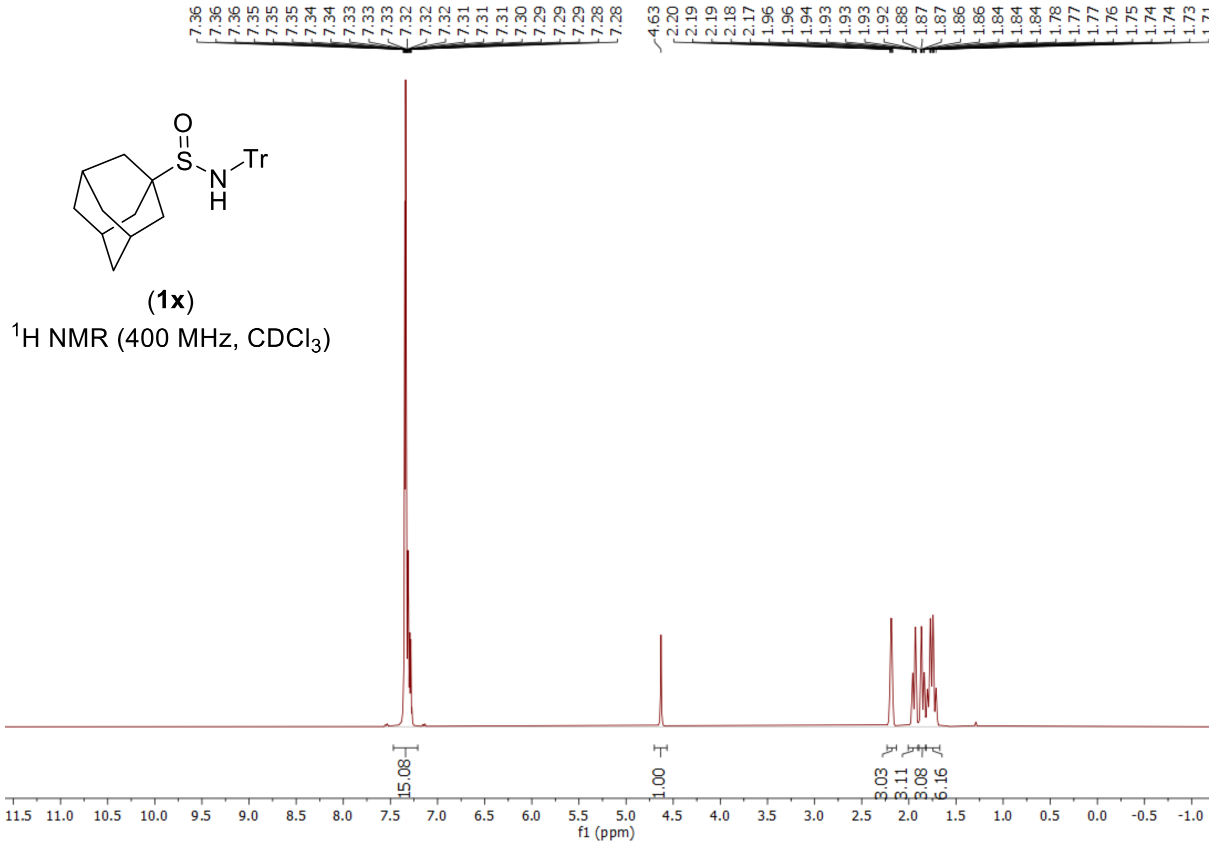
7.36
7.36
7.36
7.35
7.35
7.35
7.34
7.34
7.33
7.33
7.33
7.32
7.32
7.32
7.31
7.31
7.31
7.30
7.29
7.29
7.29
7.28
7.28
4.63
2.20
2.19
2.18
2.17
1.96
1.96
1.94
1.93
1.93
1.93
1.92
1.88
1.87
1.87
1.86
1.84
1.84
1.84
1.78
1.77
1.77
1.76
1.75
1.74
1.74
1.73
O
S
N
Tr
H
(1x)
1H NMR (400 MHz, CDCl3)
15.08
1.00
3.03
3.11
3.08
6.16
f1 (ppm)

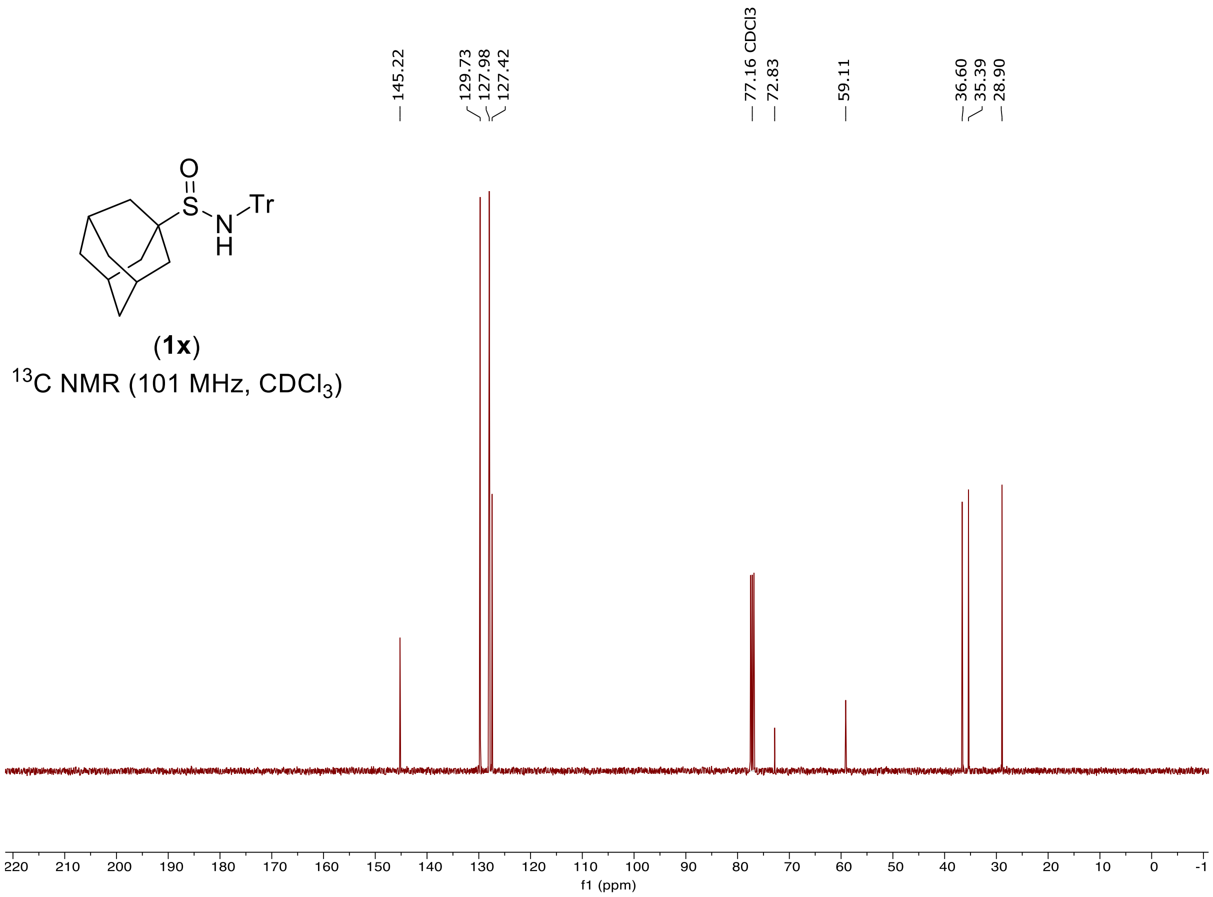
145.22
129.73
127.98
127.42
77.16 CDCl3
72.83
59.11
36.60
35.39
28.90
O
S
N
Tr
H
(1x)
13C NMR (101 MHz, CDCl3)
f1 (ppm)

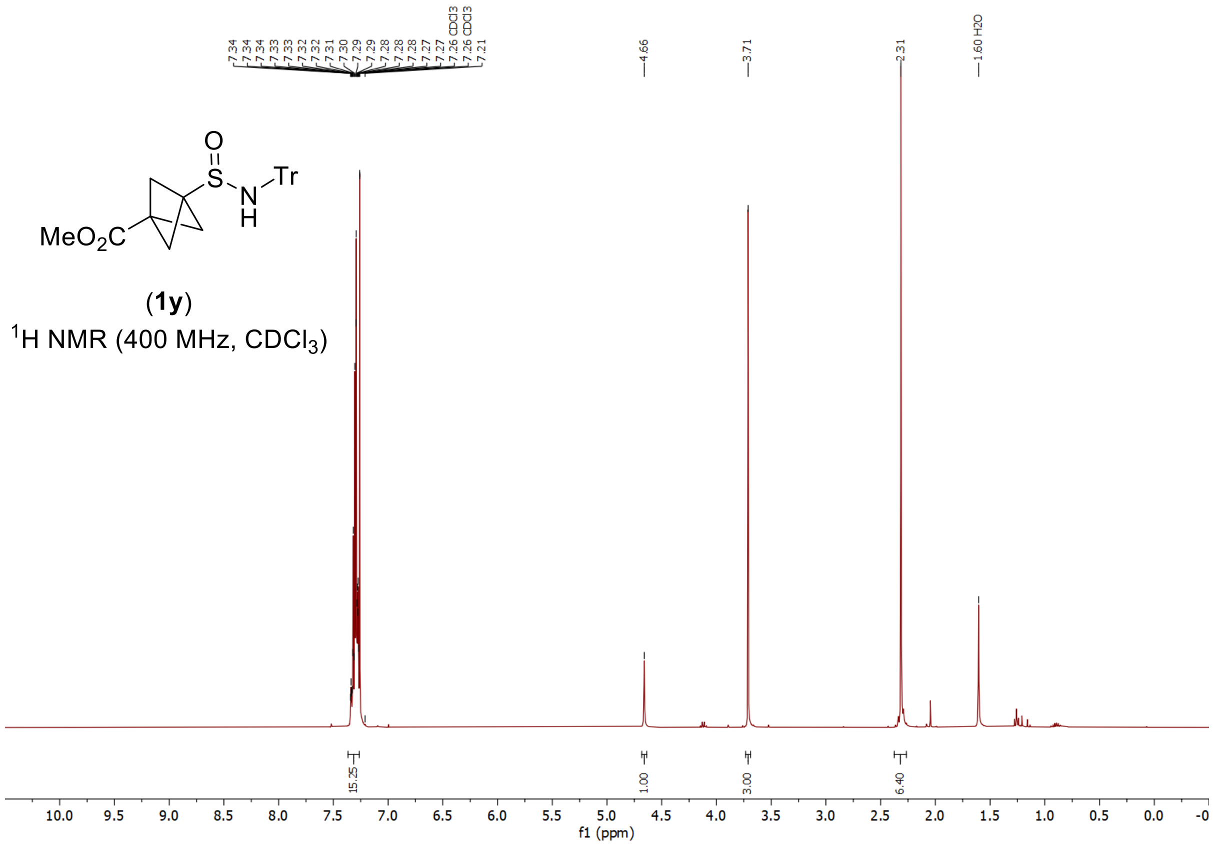

(**1y**)

^{1}H NMR (400 MHz, $CDCl_3$)

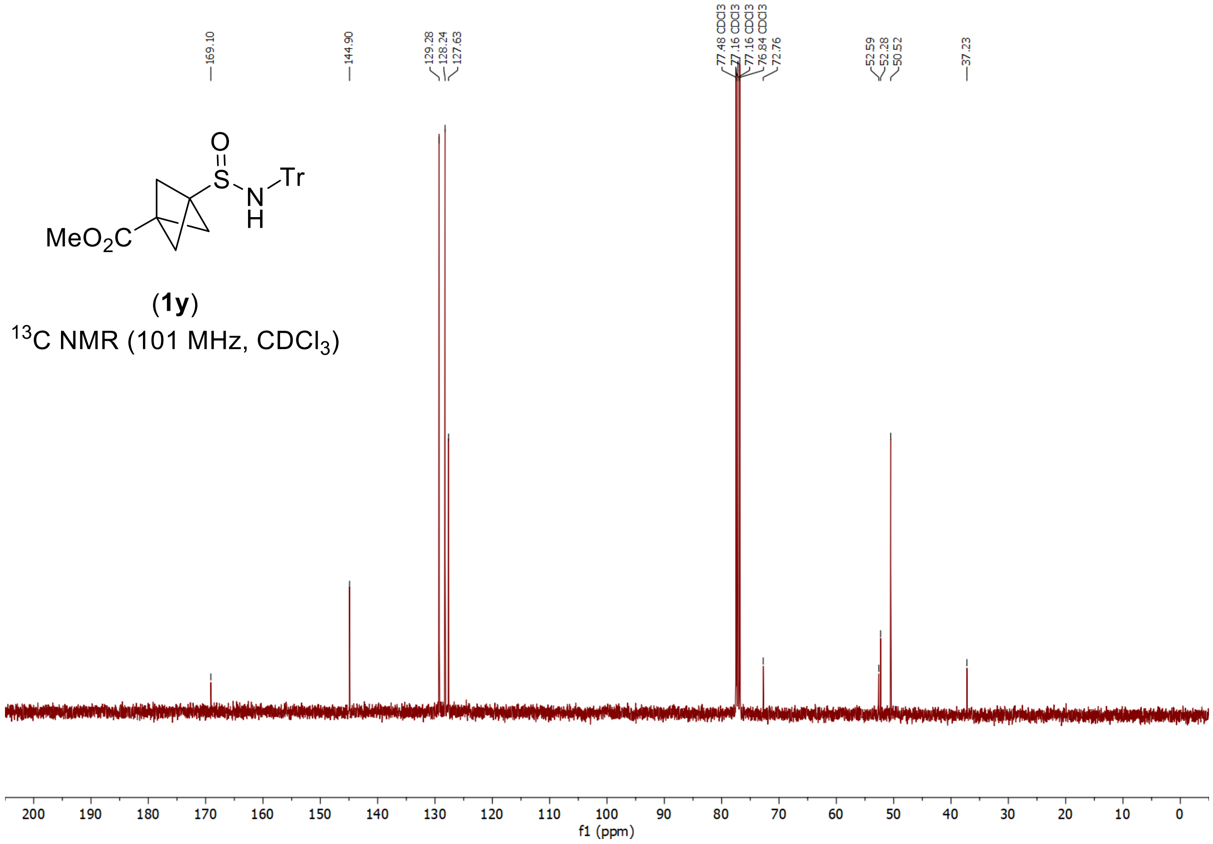

(**1y**)

^{13}C NMR (101 MHz, $CDCl_3$)

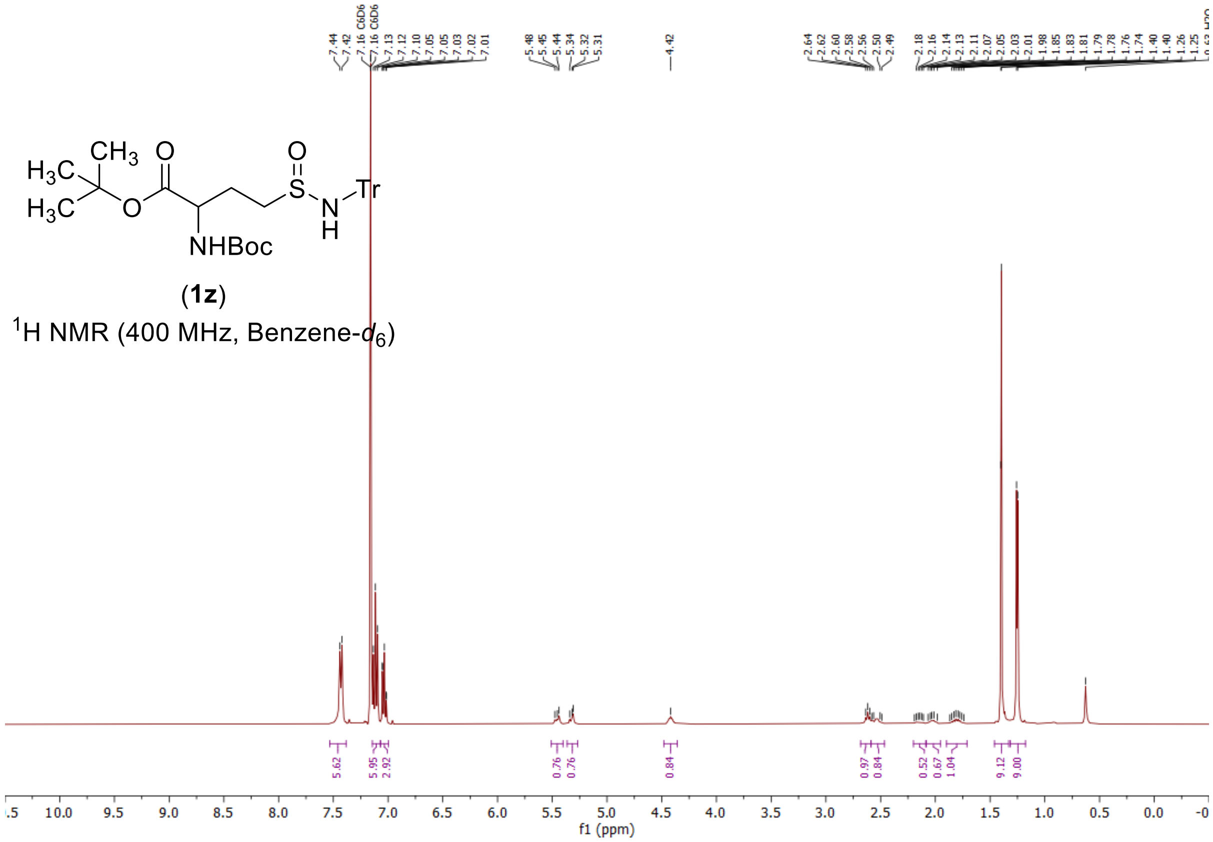
(1z)
^{1}H NMR (400 MHz, Benzene-d_6)
f1 (ppm)

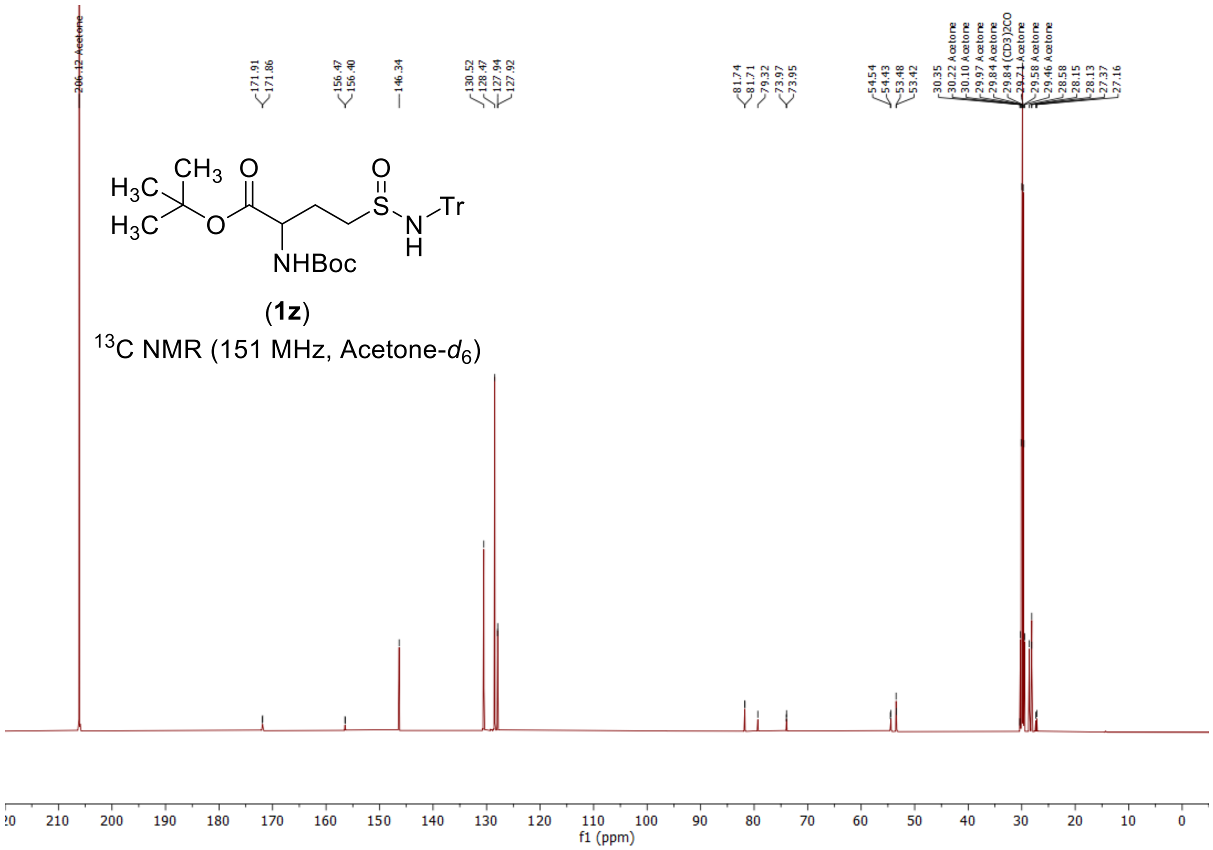
(1z)
^{13}C NMR (151 MHz, Acetone-d_6)
f1 (ppm)

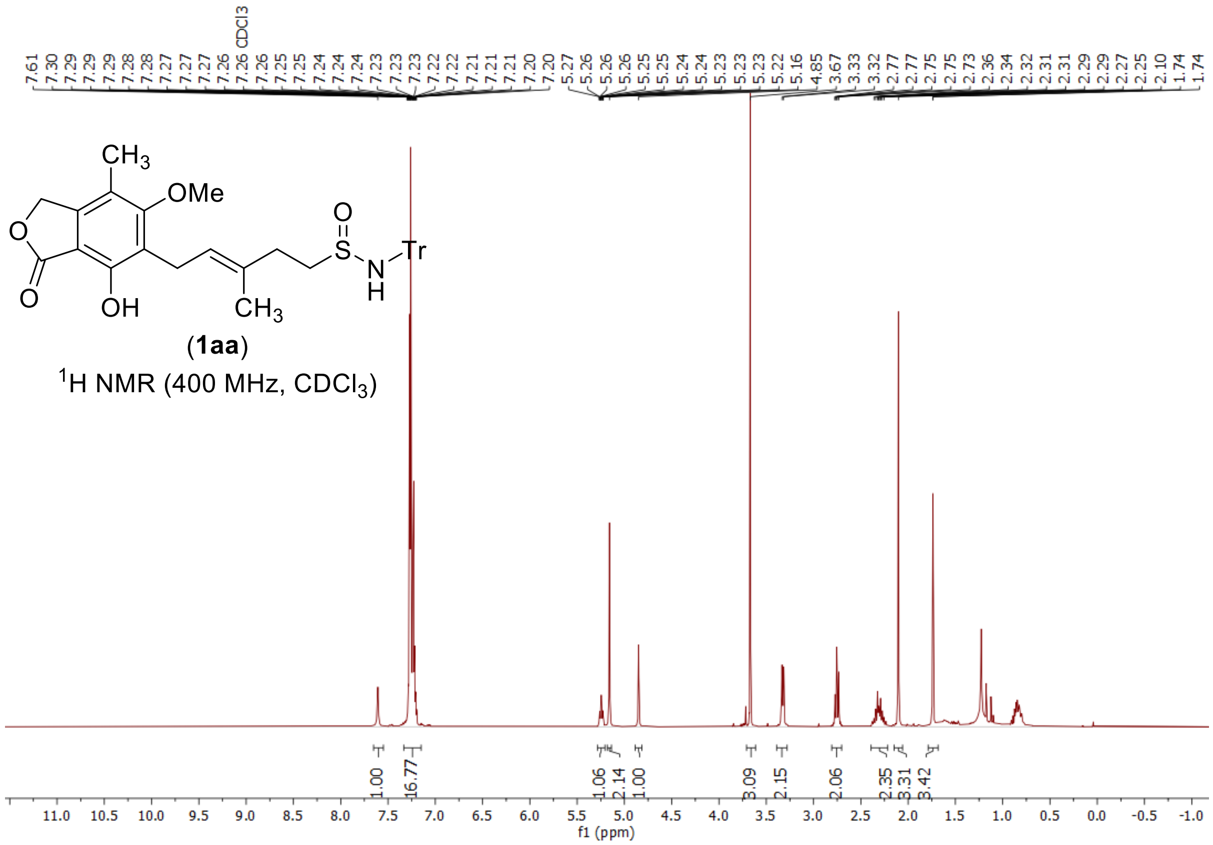
7.61 7.30 7.29 7.29 7.29 7.28 7.28 7.27 7.27 7.27 7.26 7.26 CDCl3 7.26 7.25 7.25 7.24 7.24 7.24 7.23 7.23 7.23 7.22 7.22 7.21 7.21 7.21 7.20 7.20 5.27 5.26 5.26 5.26 5.25 5.25 5.24 5.24 5.23 5.23 5.23 5.22 5.16 4.85 3.67 3.33 3.32 2.77 2.77 2.75 2.73 2.36 2.34 2.32 2.31 2.31 2.29 2.29 2.27 2.25 2.10 1.74 1.74
CH3
OMe
O
O
OH
CH3
O
S
N
H
Tr
(1aa)
1H NMR (400 MHz, CDCl3)
1.00 16.77 1.06 2.14 1.00 3.09 2.15 2.06 2.35 3.31 3.42
f1 (ppm)

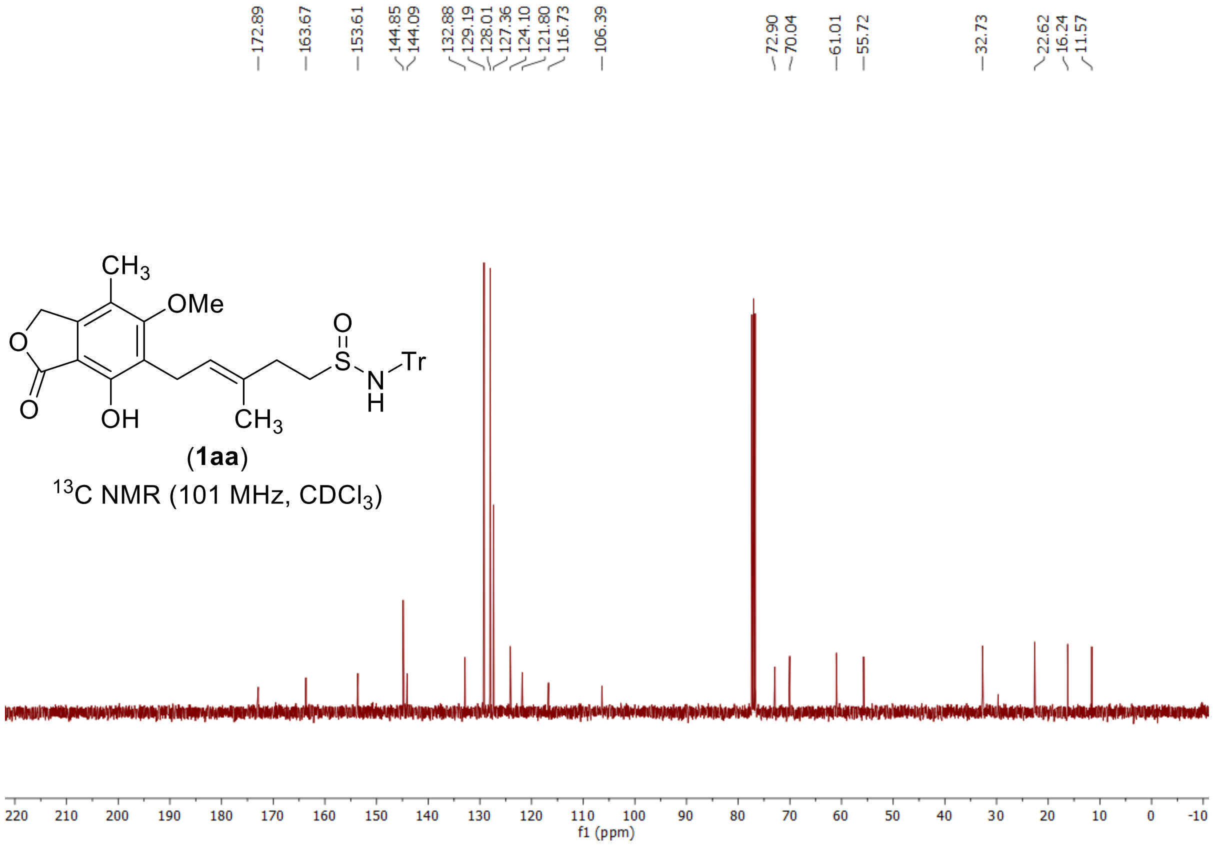
172.89 163.67 153.61 144.85 144.09 132.88 129.19 128.01 127.36 124.10 121.80 116.73 106.39 72.90 70.04 61.01 55.72 32.73 22.62 16.24 11.57
CH3
OMe
O
O
OH
CH3
O
S
N
H
Tr
(1aa)
13C NMR (101 MHz, CDCl3)
f1 (ppm)

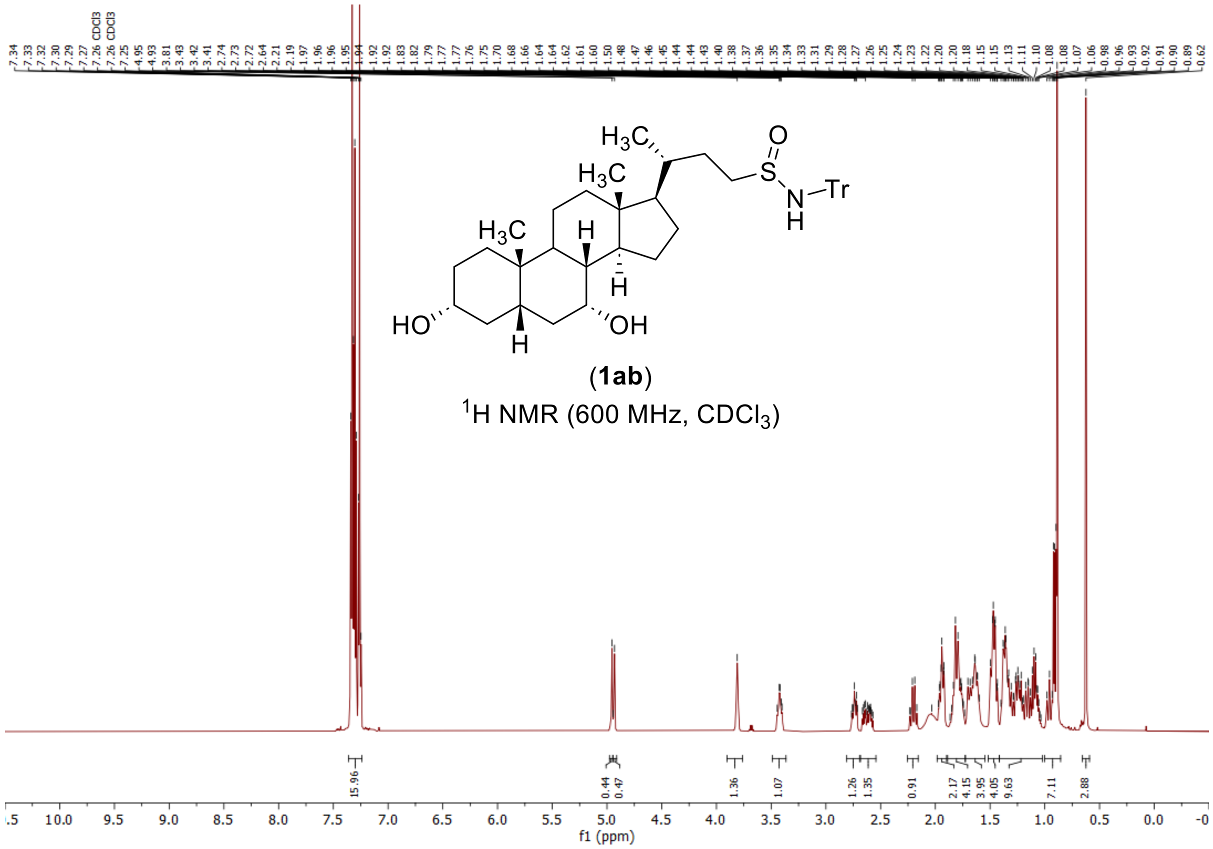
(1ab)
1H NMR (600 MHz, CDCl3)
f1 (ppm)

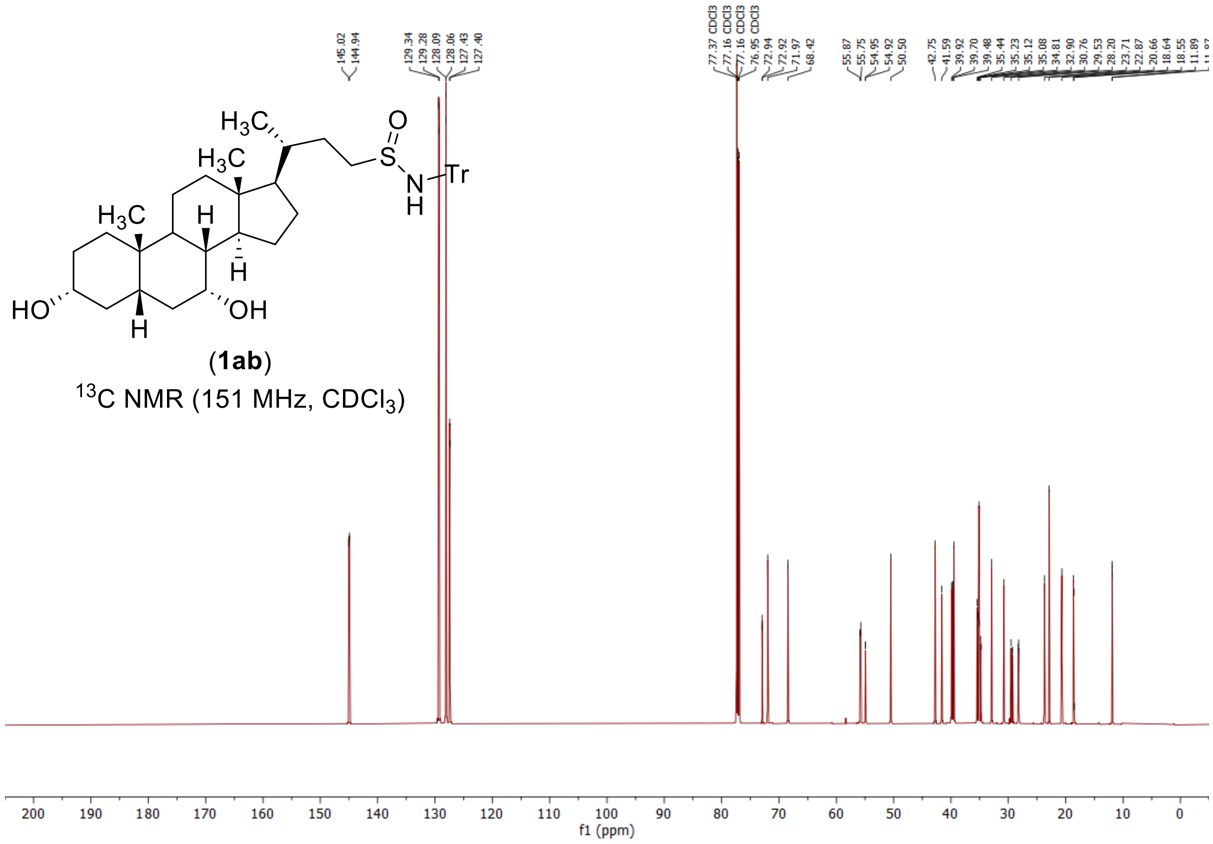
(1ab)
13C NMR (151 MHz, CDCl3)
f1 (ppm)

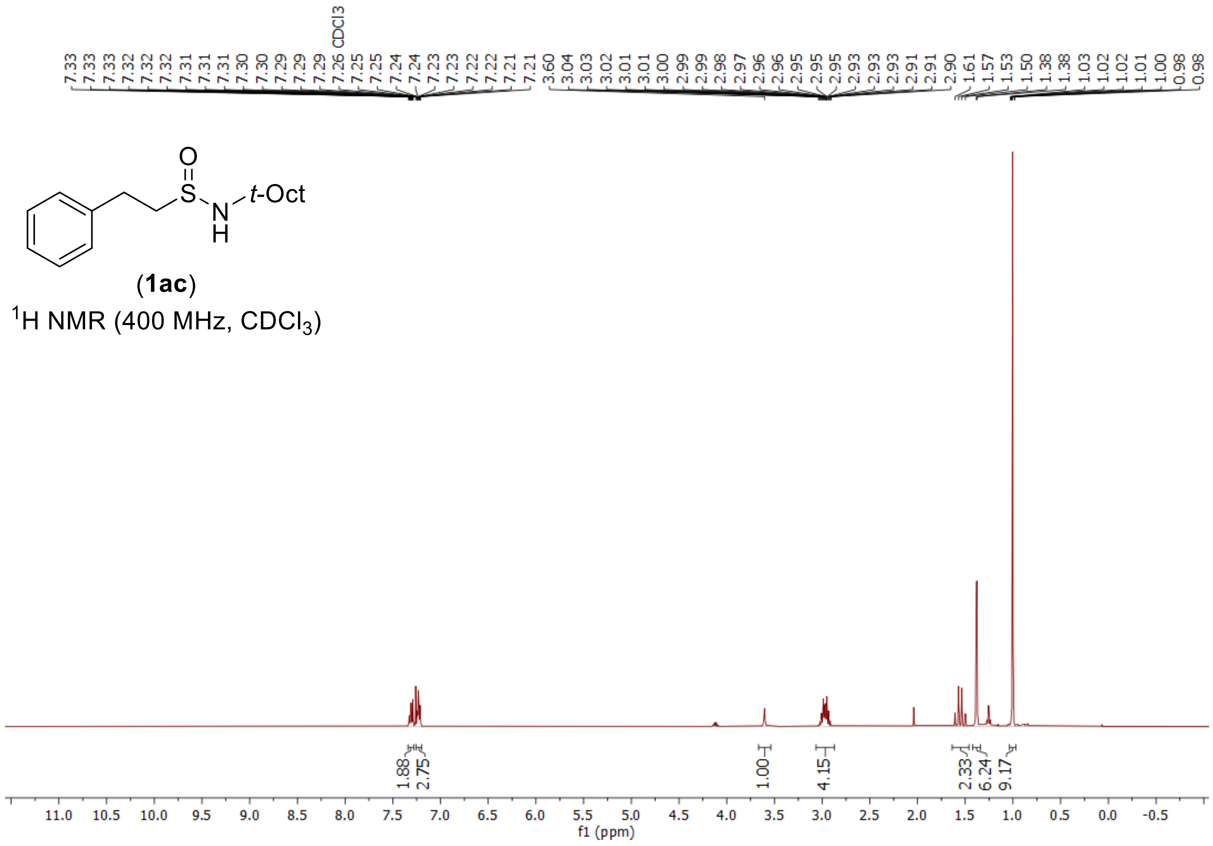

t-Oct
(1ac)
^{1}H NMR (400 MHz, CDCl$_3$)
f1 (ppm)

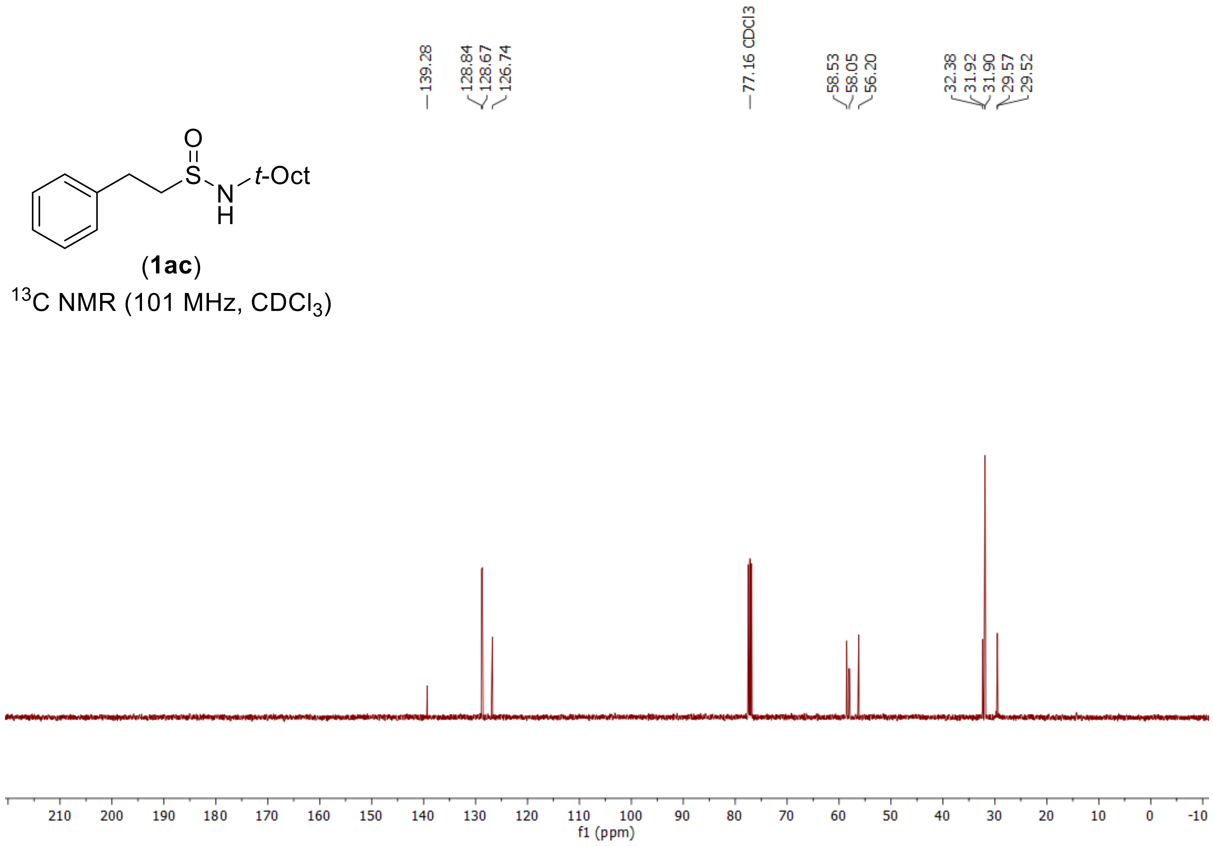

t-Oct
(1ac)
^{13}C NMR (101 MHz, CDCl$_3$)
f1 (ppm)

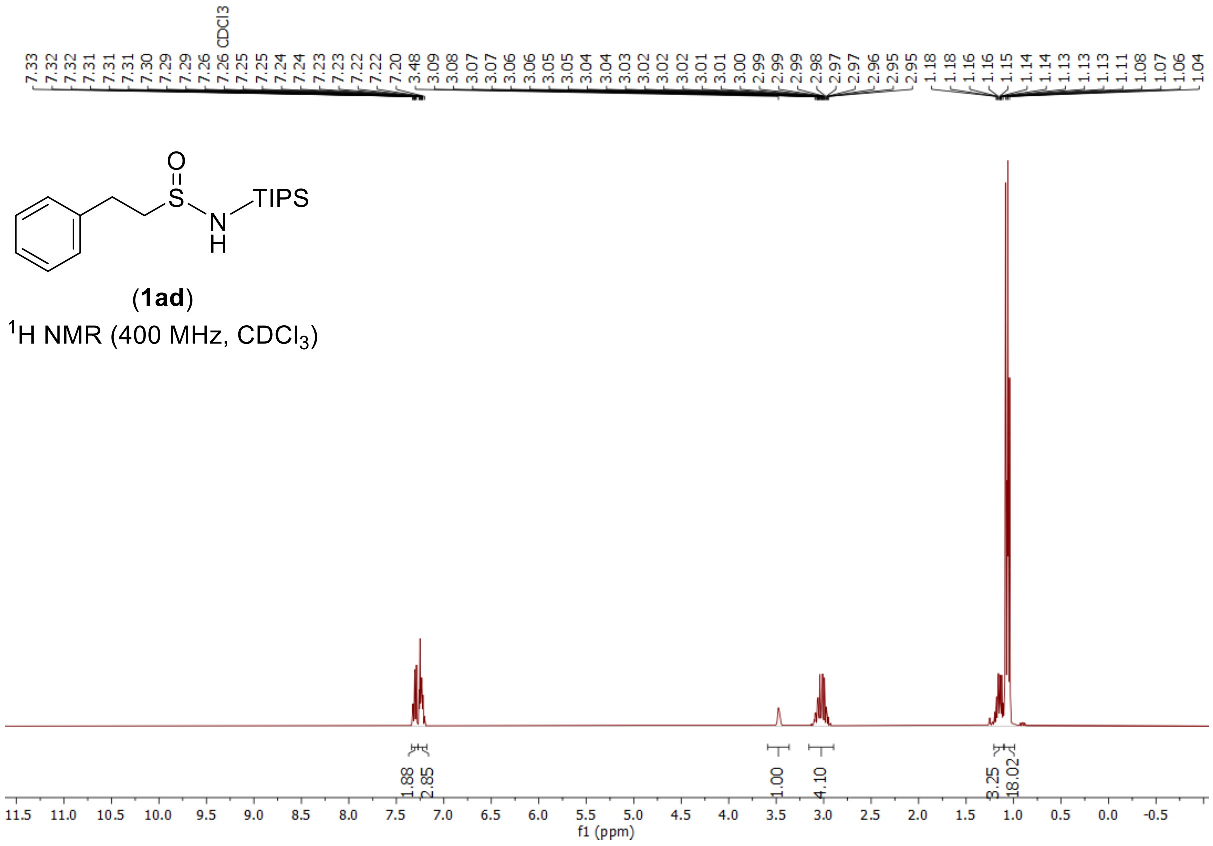

7.33 7.32 7.32 7.31 7.31 7.30 7.29 7.29 7.26 7.26 CDCl3 7.25 7.25 7.24 7.24 7.23 7.23 7.22 7.22 7.20 3.48 3.09 3.08 3.07 3.07 3.06 3.06 3.05 3.05 3.04 3.04 3.03 3.02 3.02 3.01 3.01 3.00 2.99 2.99 2.98 2.97 2.97 2.96 2.95 2.95 1.18 1.18 1.16 1.16 1.15 1.14 1.14 1.13 1.13 1.13 1.11 1.08 1.07 1.06 1.04
O
S
N
H
TIPS
(1ad)
1H NMR (400 MHz, CDCl3)
1.88
2.85
1.00
4.10
3.25
18.02
11.5 11.0 10.5 10.0 9.5 9.0 8.5 8.0 7.5 7.0 6.5 6.0 5.5 5.0 4.5 4.0 3.5 3.0 2.5 2.0 1.5 1.0 0.5 0.0 -0.5
f1 (ppm)

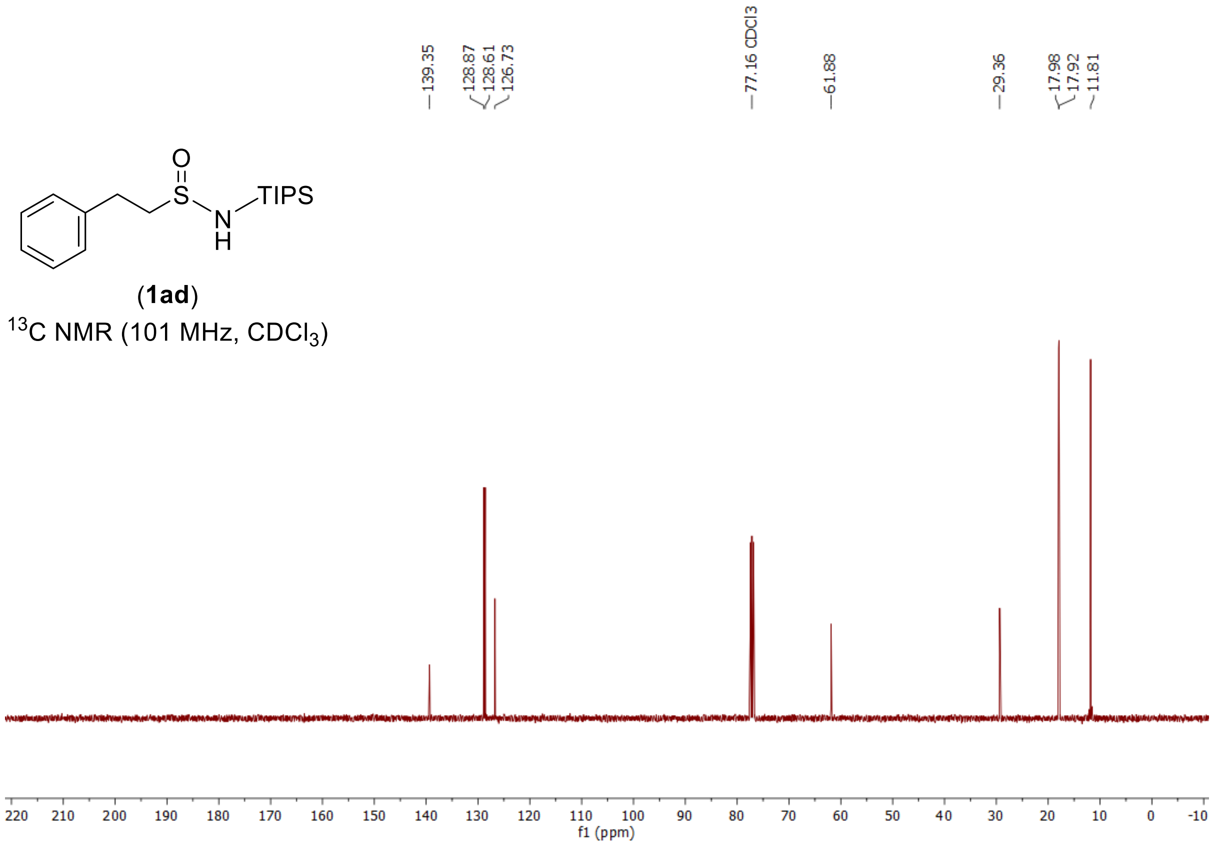

139.35
128.87
128.61
126.73
77.16 CDCl3
61.88
29.36
17.98
17.92
11.81
O
S
N
H
TIPS
(1ad)
13C NMR (101 MHz, CDCl3)
220 210 200 190 180 170 160 150 140 130 120 110 100 90 80 70 60 50 40 30 20 10 0 -10
f1 (ppm)

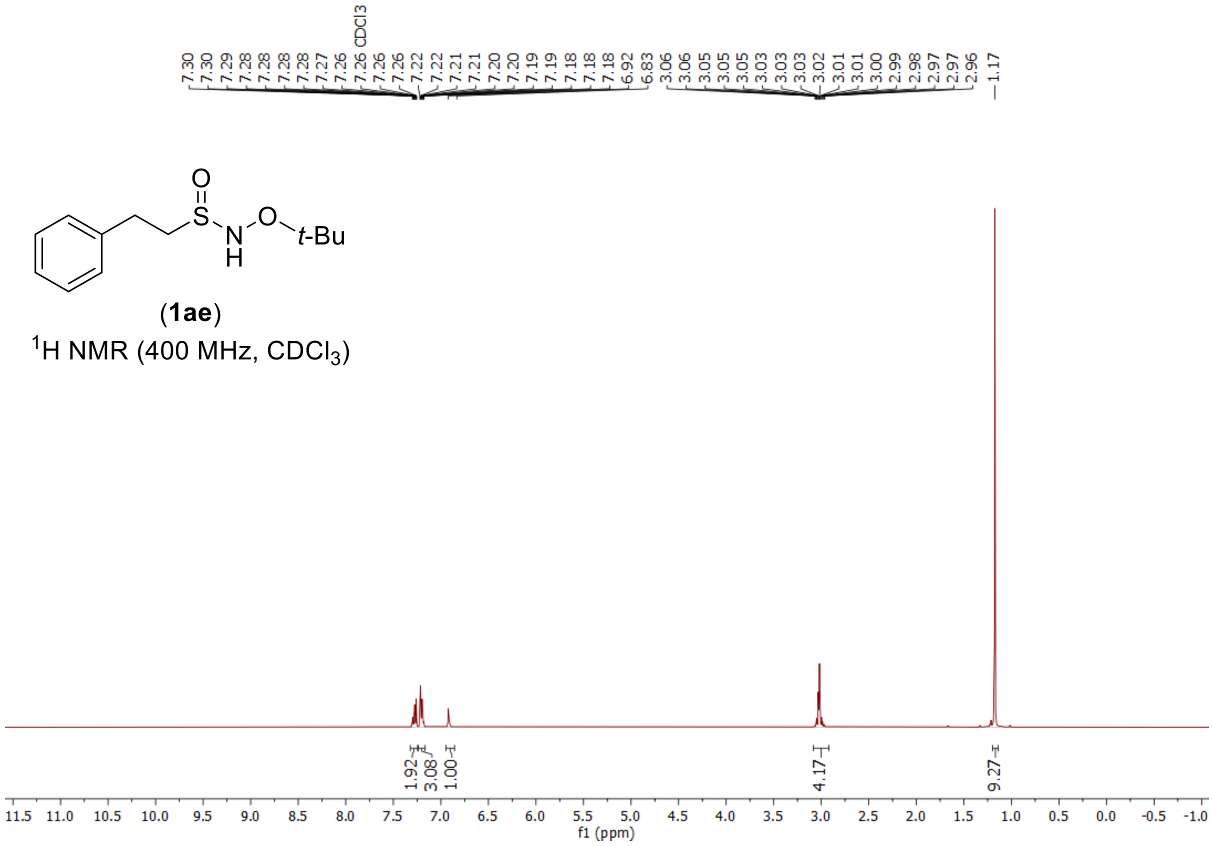

7.30 7.30 7.29 7.28 7.28 7.28 7.28 7.27 7.26 7.26 CDCl3 7.26 7.26 7.22 7.22 7.21 7.21 7.20 7.20 7.19 7.19 7.18 7.18 7.18 6.92 6.83 3.06 3.06 3.05 3.05 3.05 3.03 3.03 3.03 3.02 3.01 3.01 3.00 2.99 2.98 2.97 2.97 2.96 1.17
O
S
N
H
O
t-Bu
(1ae)
^{1}H NMR (400 MHz, CDCl$_3$)
1.92 3.08 1.00 4.17 9.27
f1 (ppm)

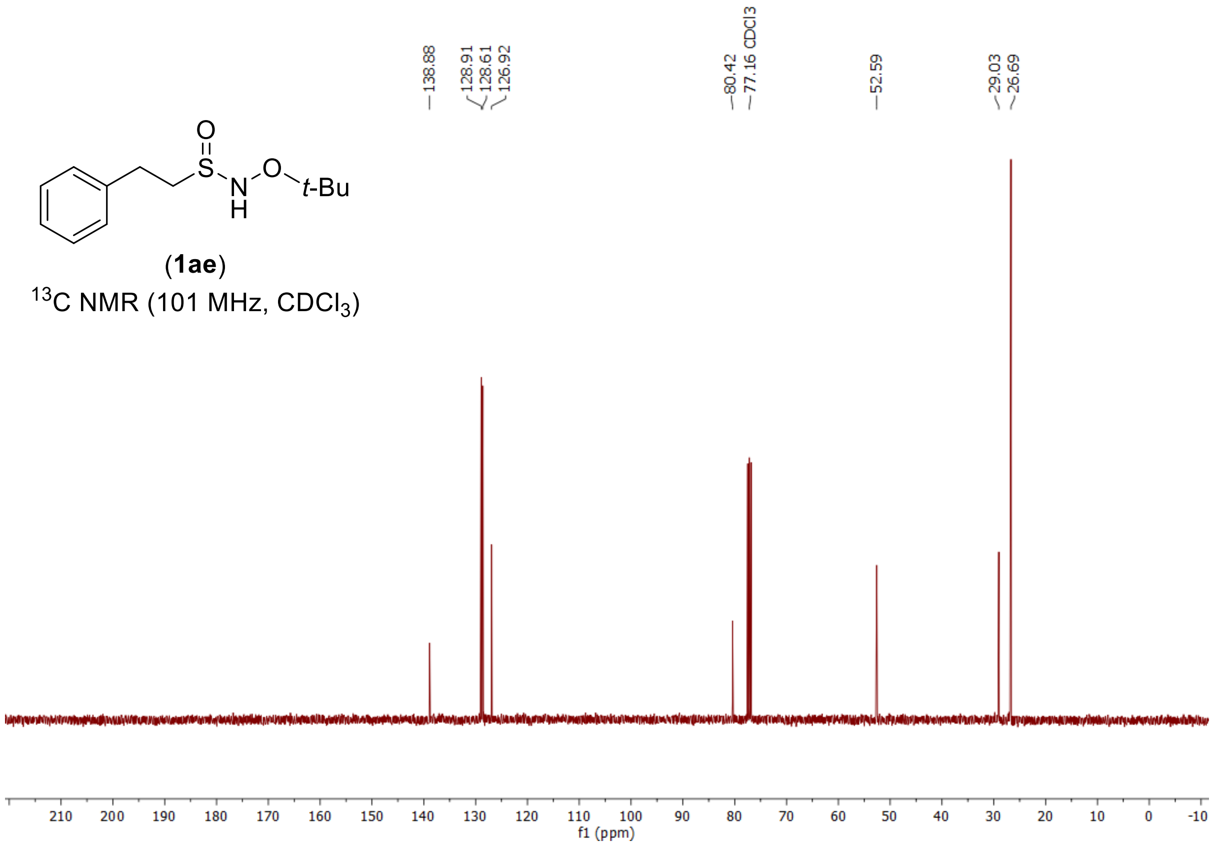

138.88 128.91 128.61 126.92 80.42 77.16 CDCl3 52.59 29.03 26.69
O
S
N
H
O
t-Bu
(1ae)
^{13}C NMR (101 MHz, CDCl$_3$)
f1 (ppm)

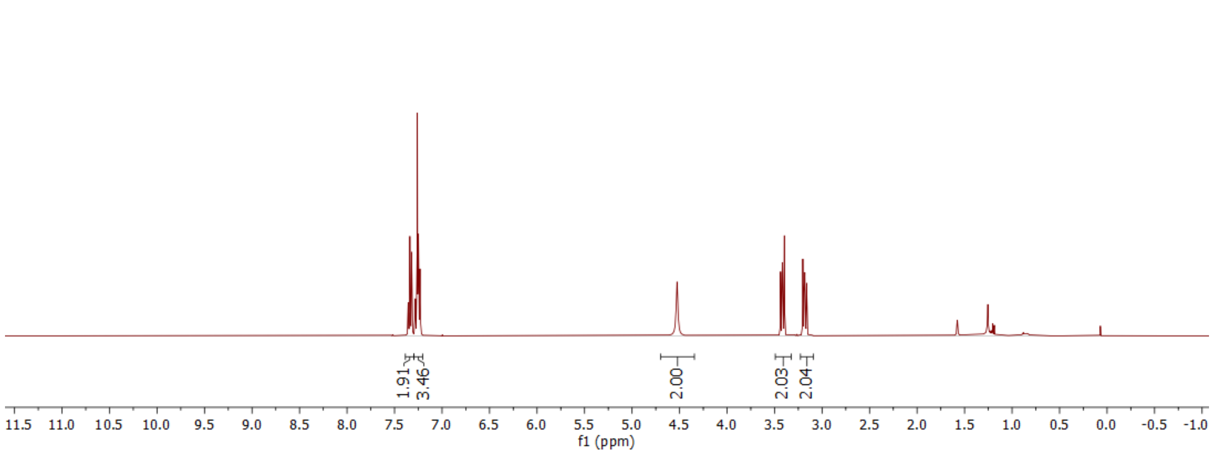

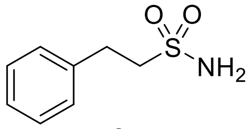

(**2a**)

^{1}H NMR (400 MHz, $CDCl_3$)

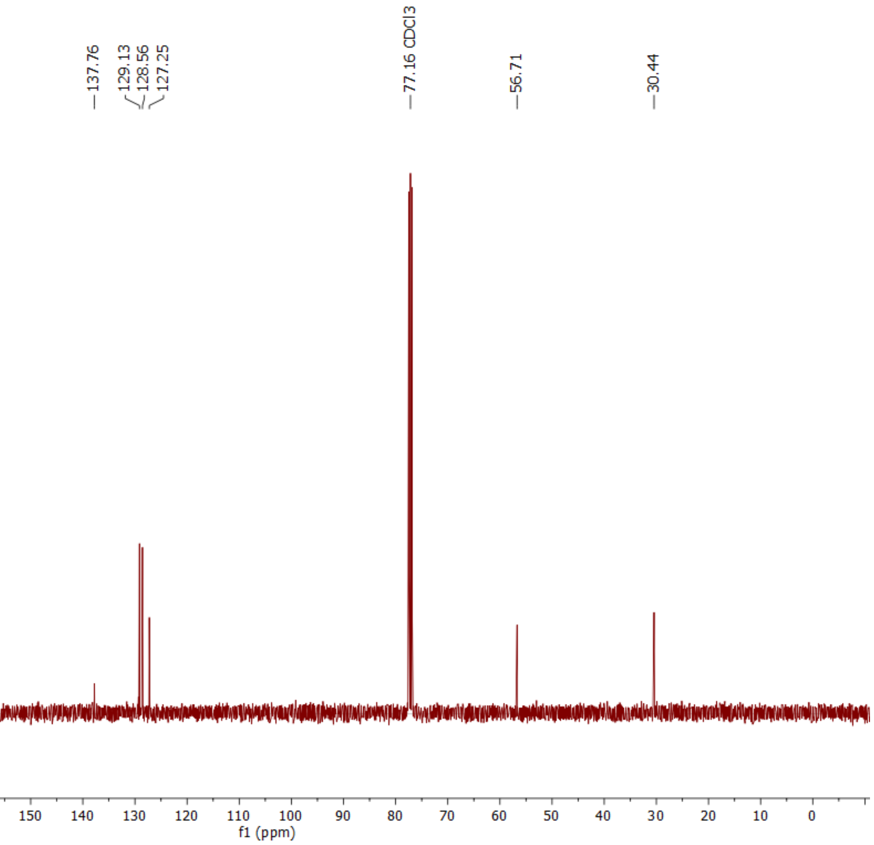

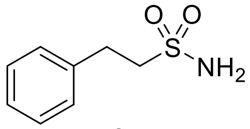

(**2a**)

^{13}C NMR (101 MHz, $CDCl_3$)

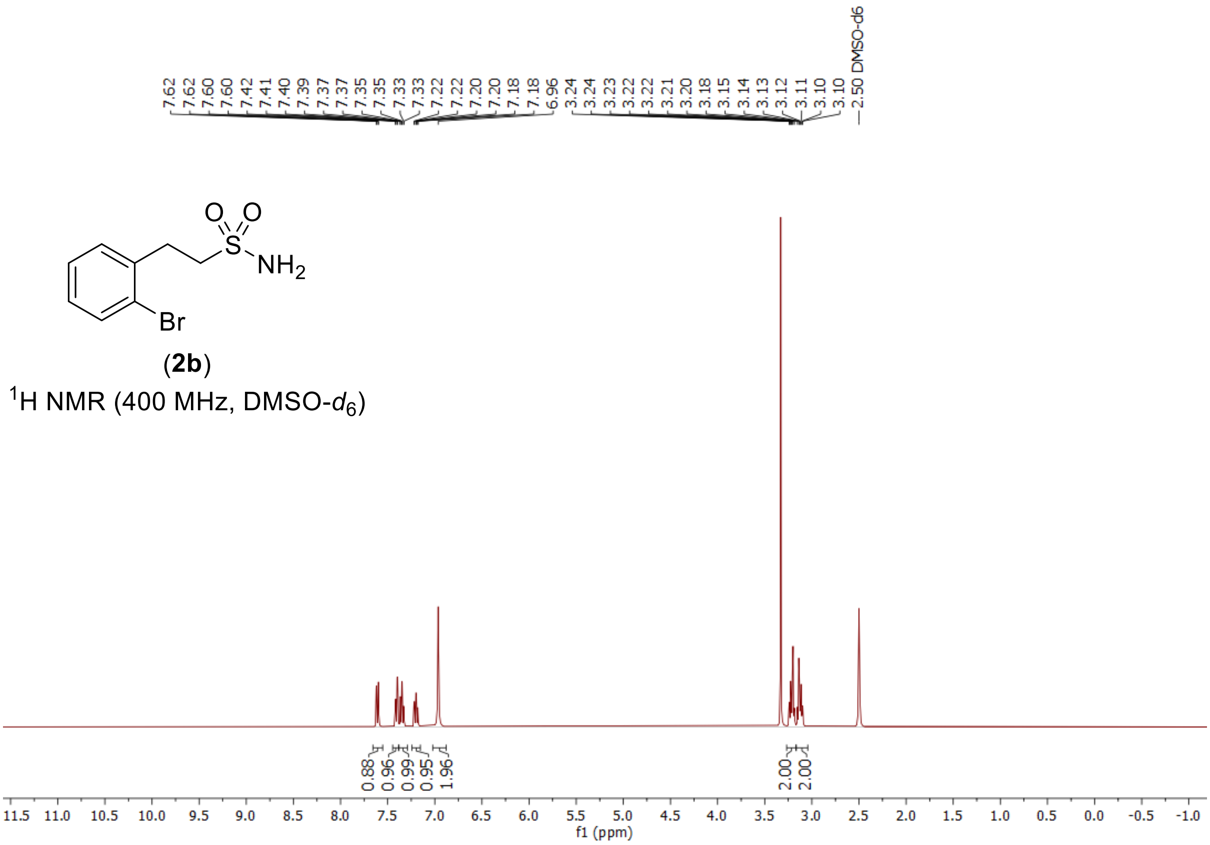

(**2b**)

^{1}H NMR (400 MHz, DMSO-d_6)

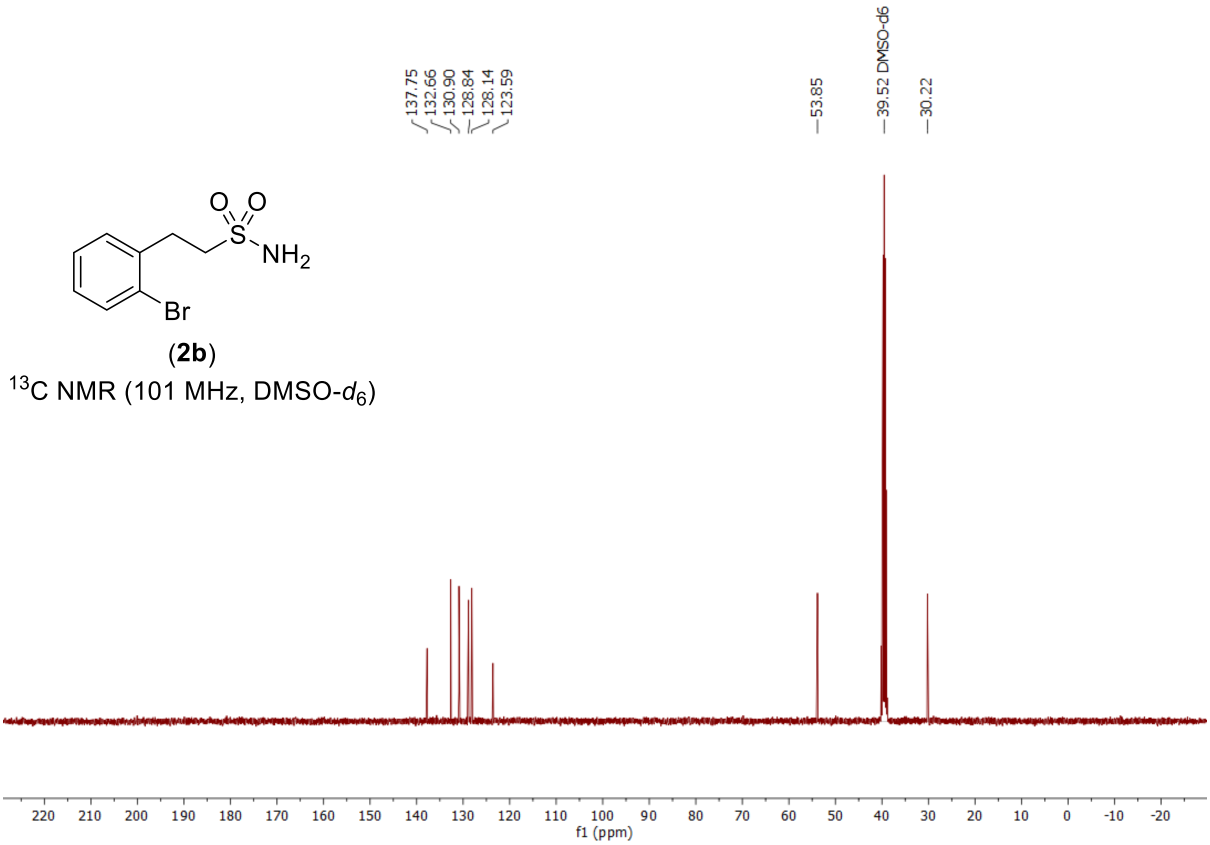

(**2b**)

^{13}C NMR (101 MHz, DMSO-d_6)

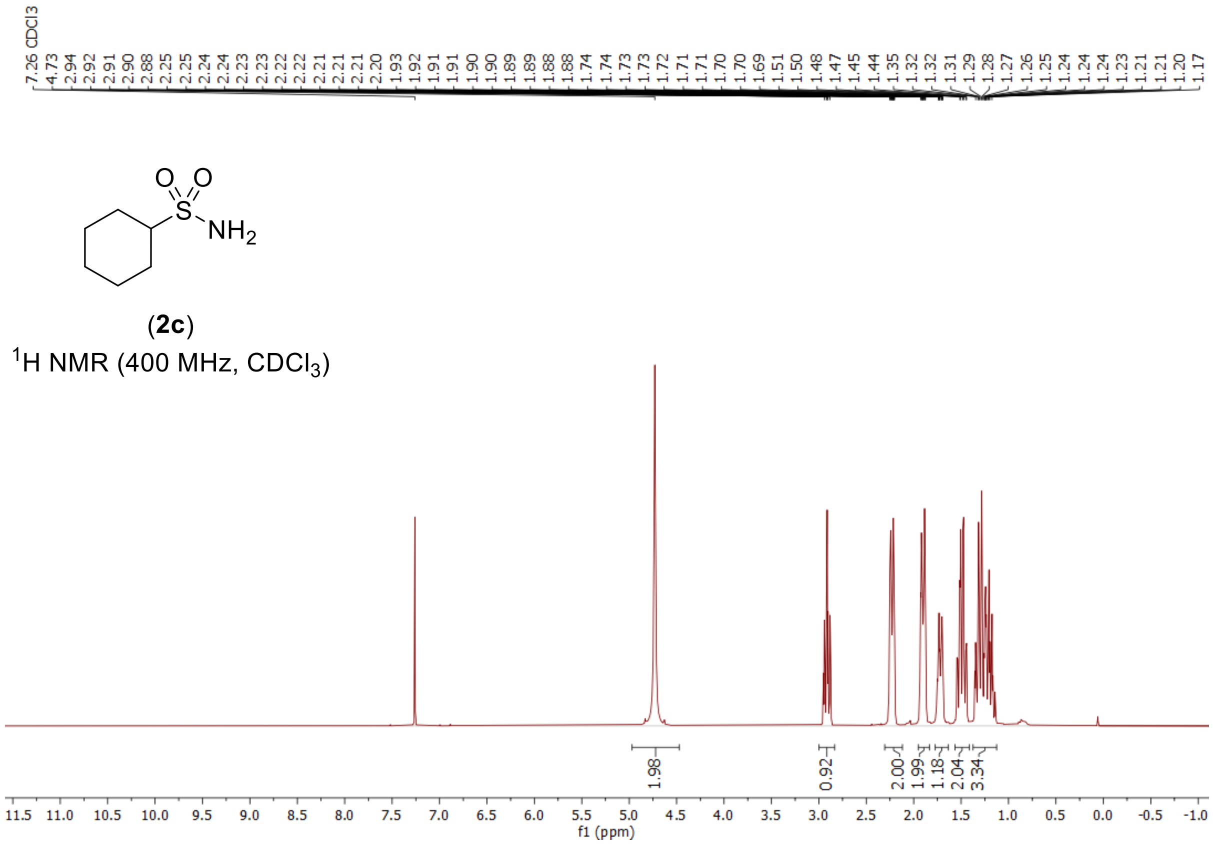

(**2c**)
^{1}H NMR (400 MHz, $CDCl_3$)

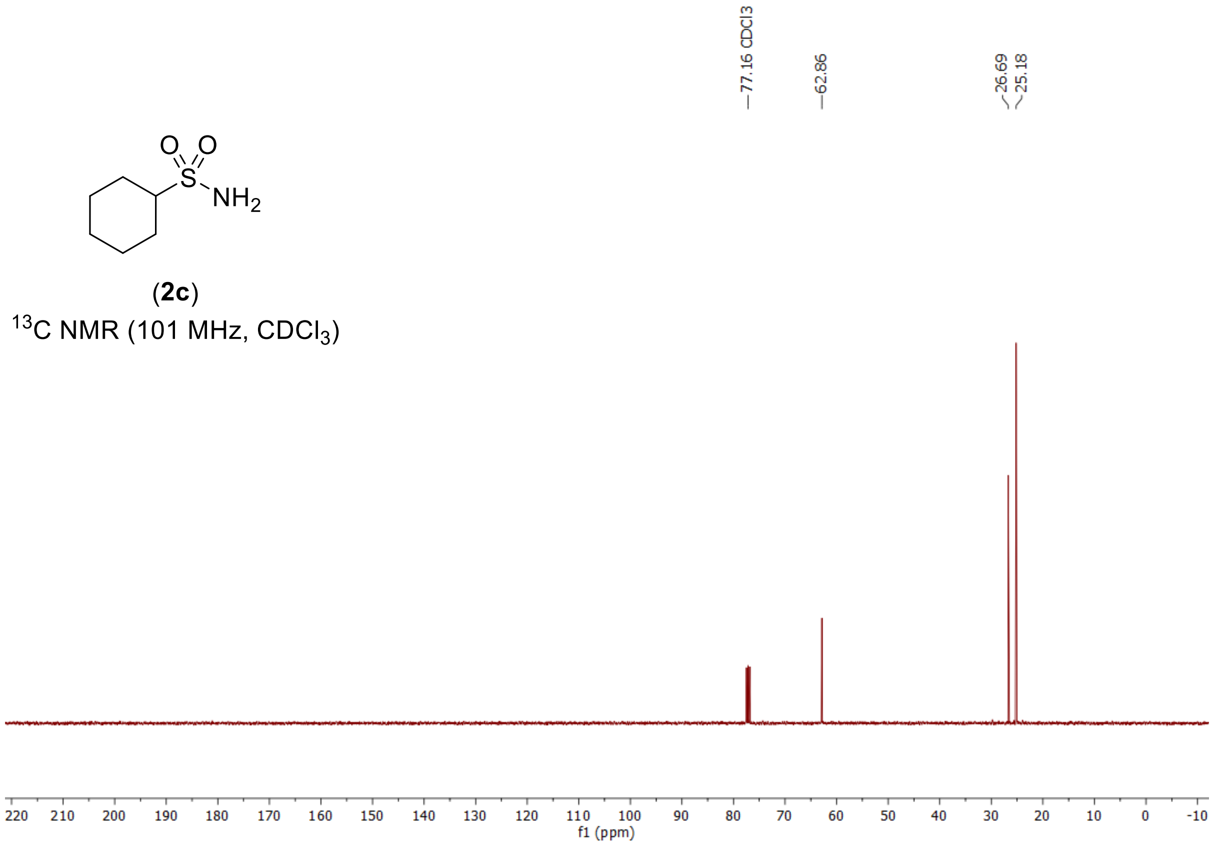

(**2c**)
^{13}C NMR (101 MHz, $CDCl_3$)

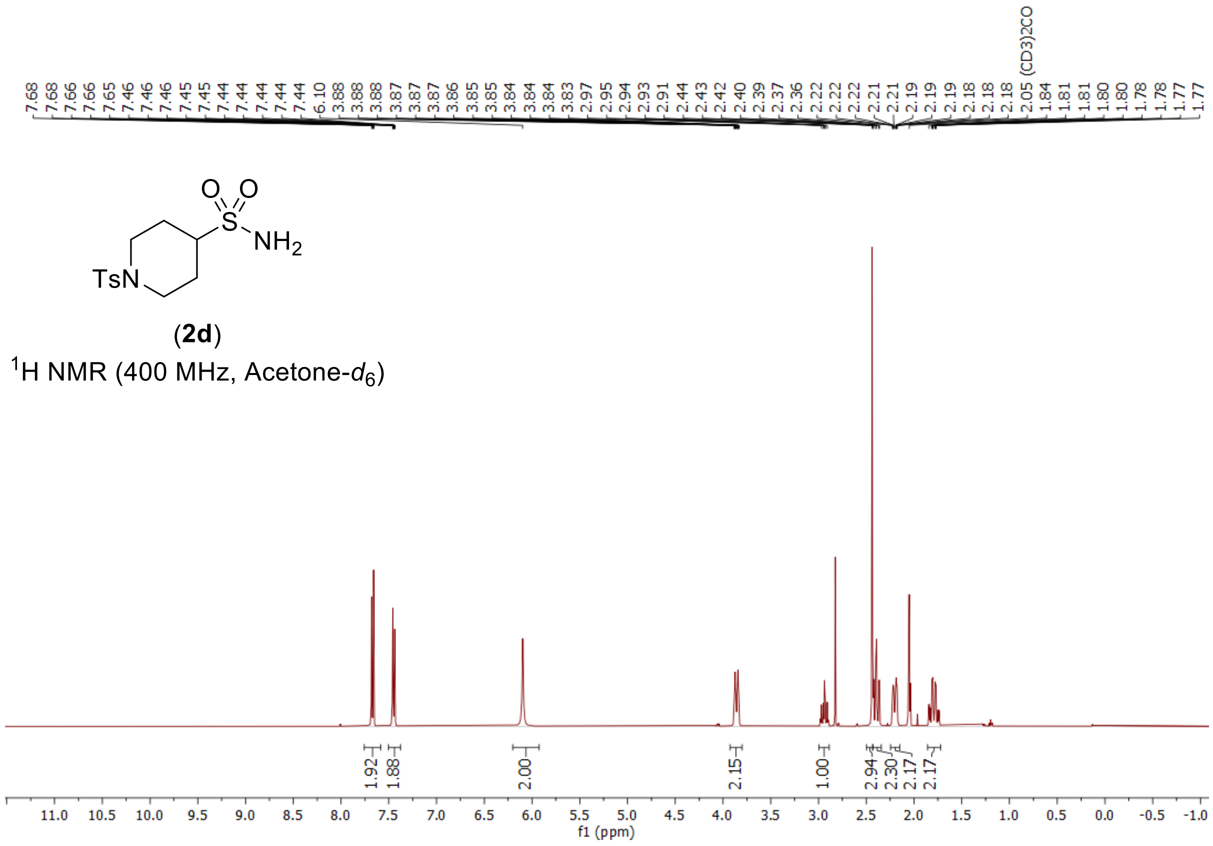

(**2d**)

^{1}H NMR (400 MHz, Acetone-d_6)

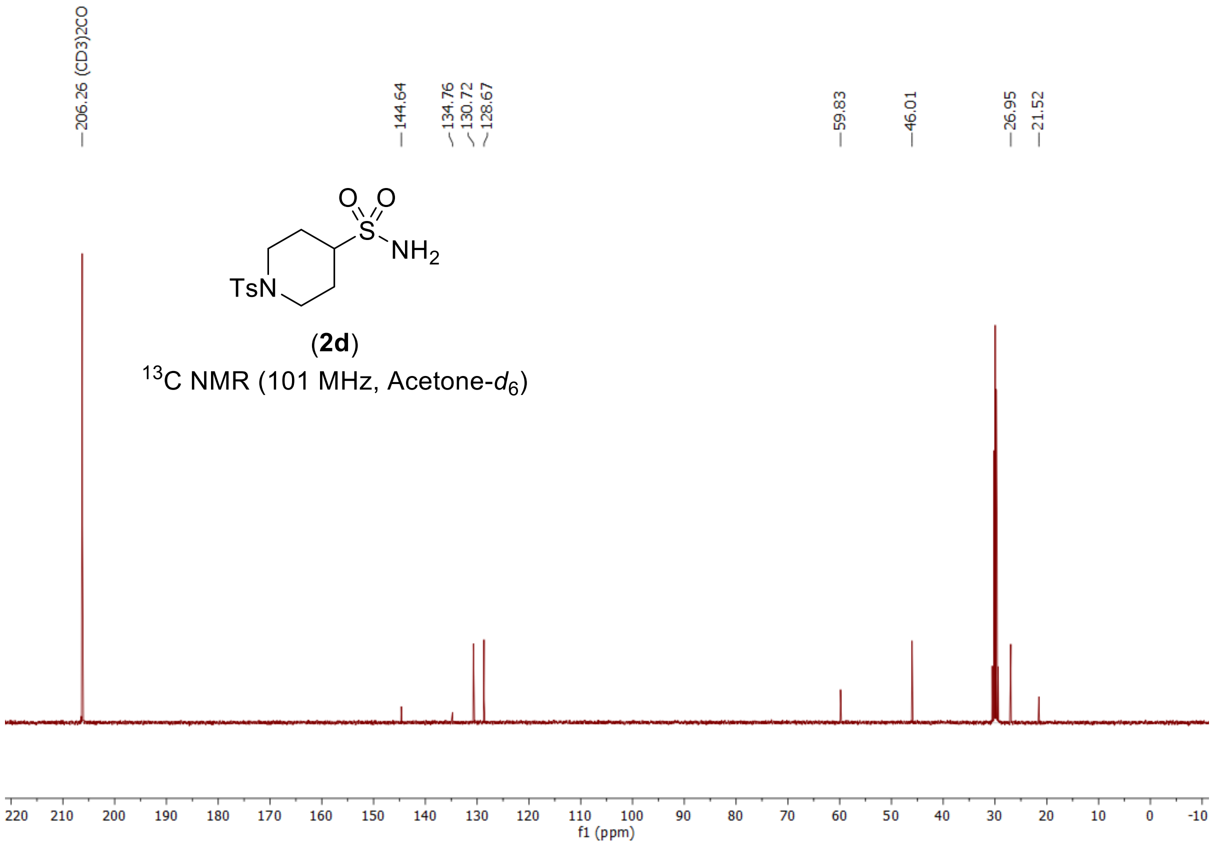

(**2d**)

^{13}C NMR (101 MHz, Acetone-d_6)

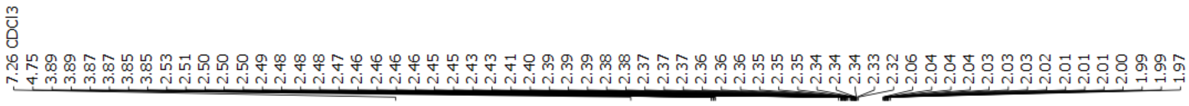

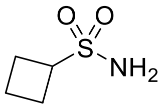

(**2e**)

^{1}H NMR (400 MHz, $CDCl_3$)

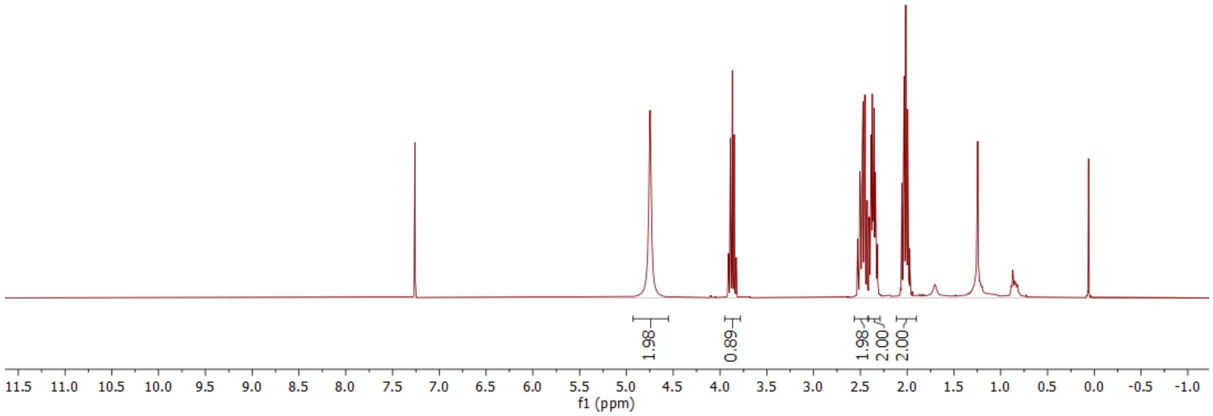

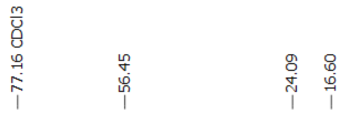

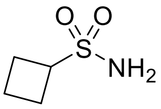

(**2e**)

^{13}C NMR (101 MHz, $CDCl_3$)

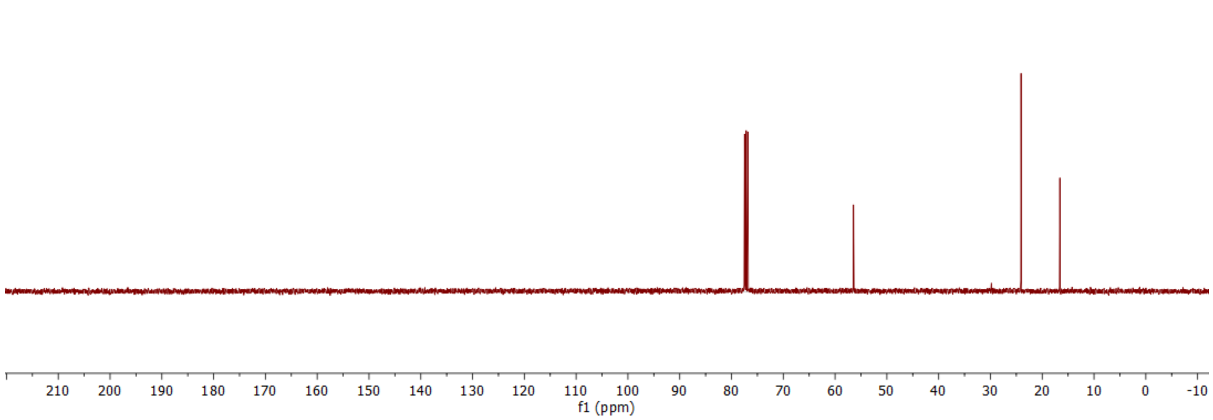

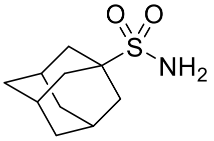

(**2f**)
^{1}H NMR (400 MHz, $CDCl_3$)

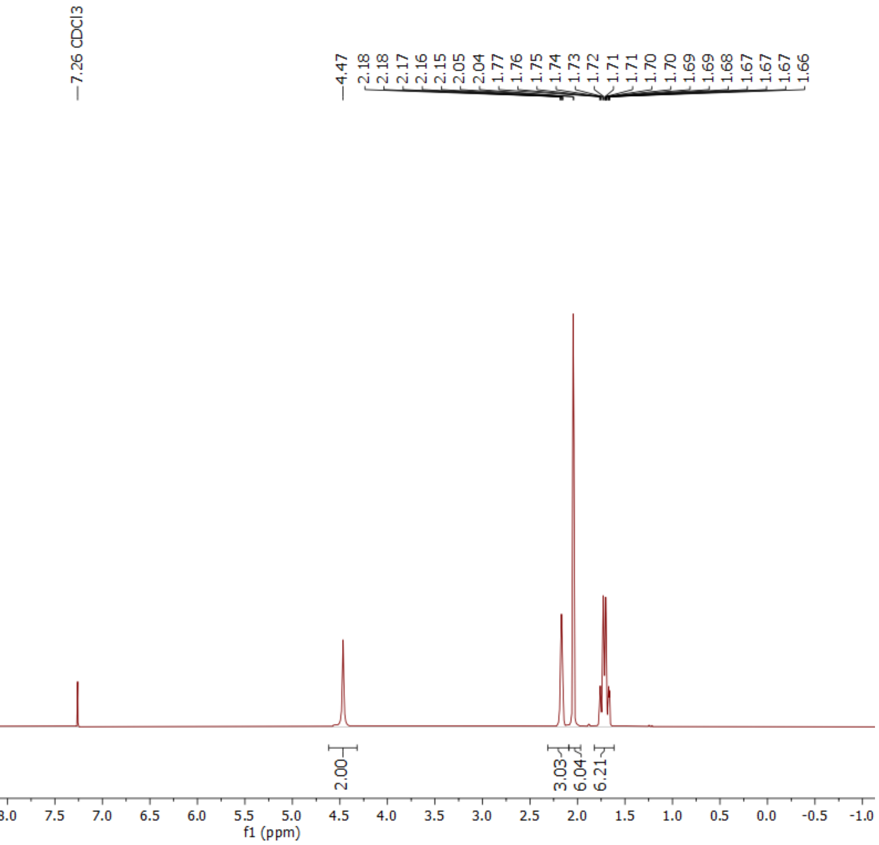

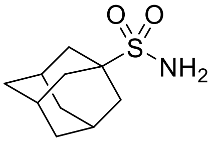

(**2f**)
^{13}C NMR (101 MHz, $CDCl_3$)

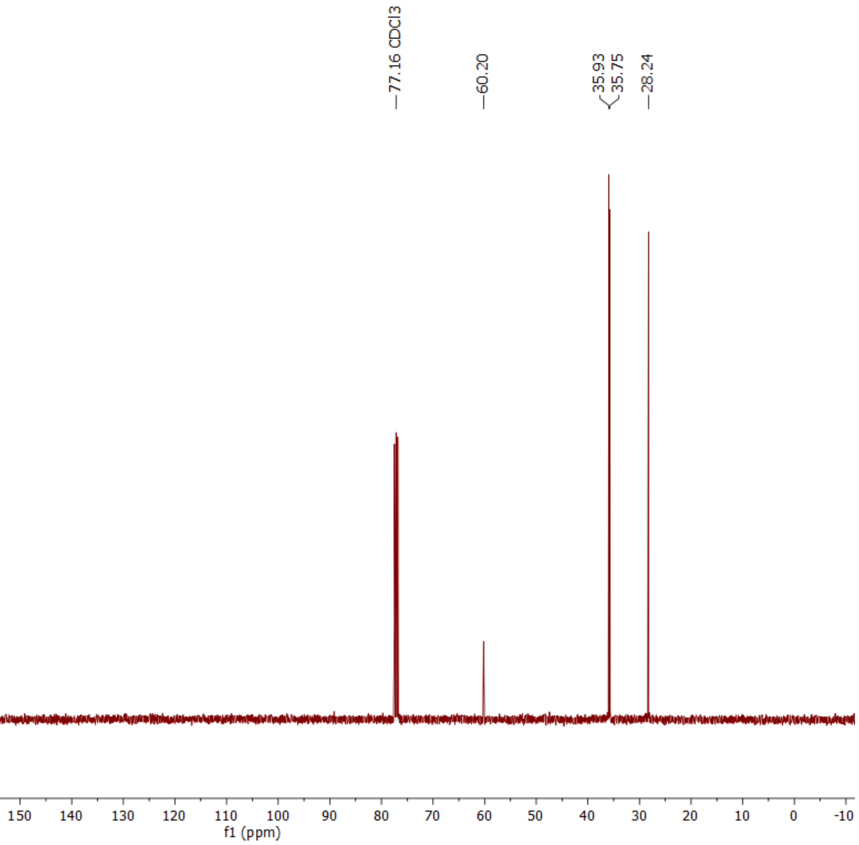

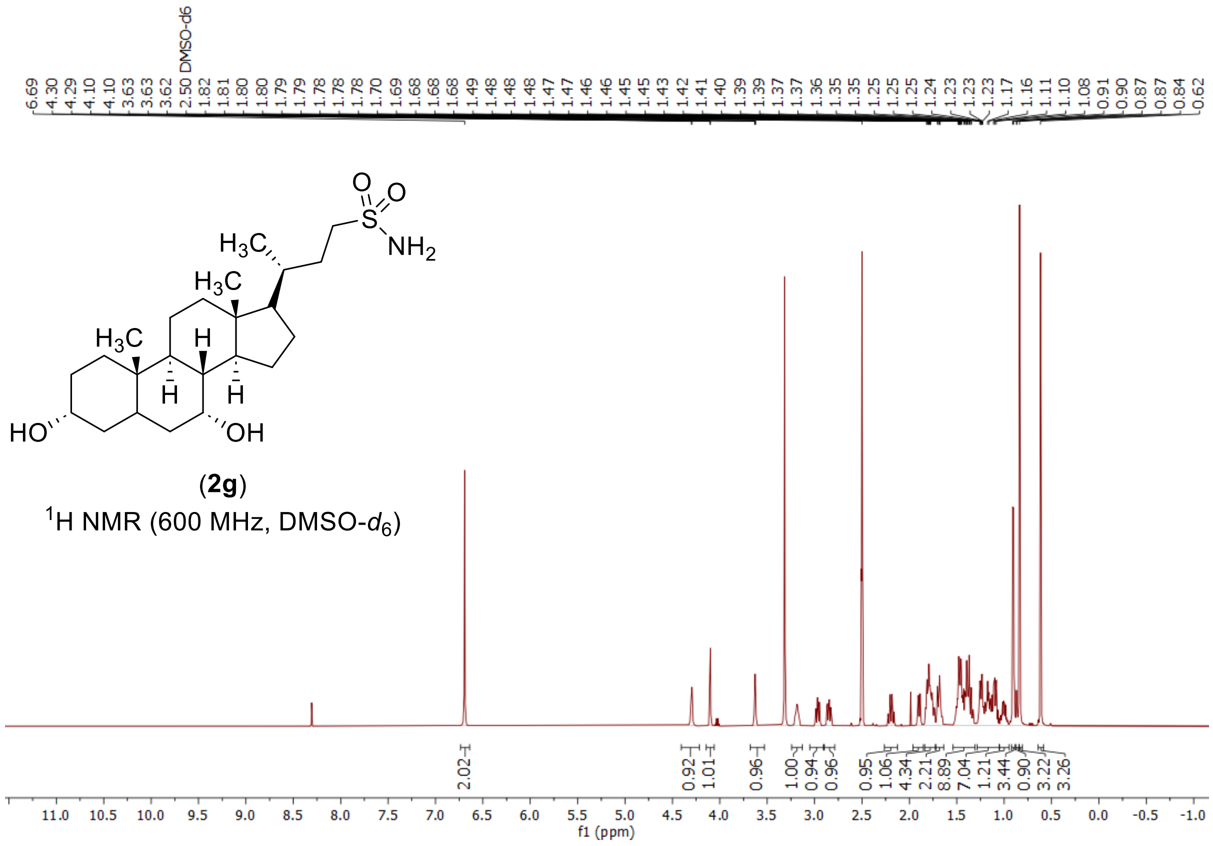

(**2g**)
^{1}H NMR (600 MHz, DMSO-d_6)

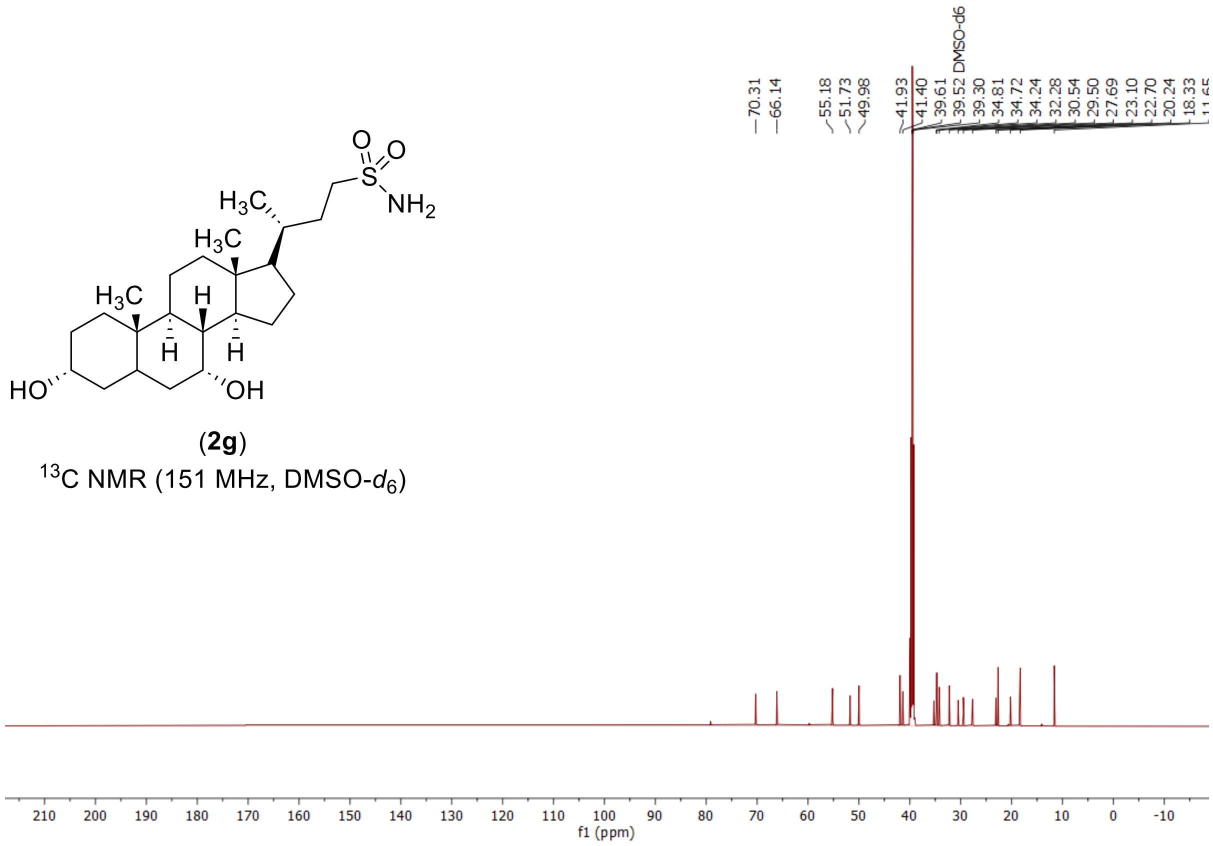

(**2g**)
^{13}C NMR (151 MHz, DMSO-d_6)

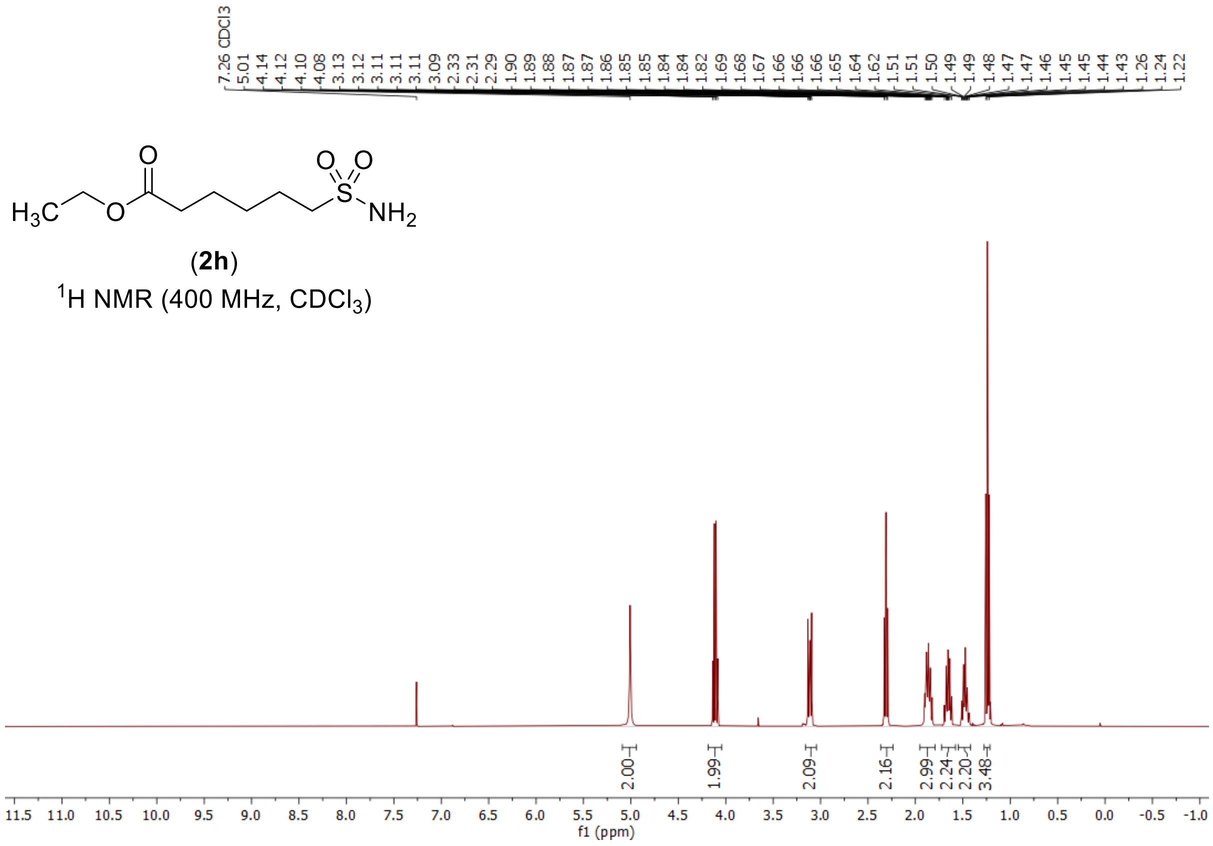

(**2h**)
^{1}H NMR (400 MHz, $CDCl_3$)

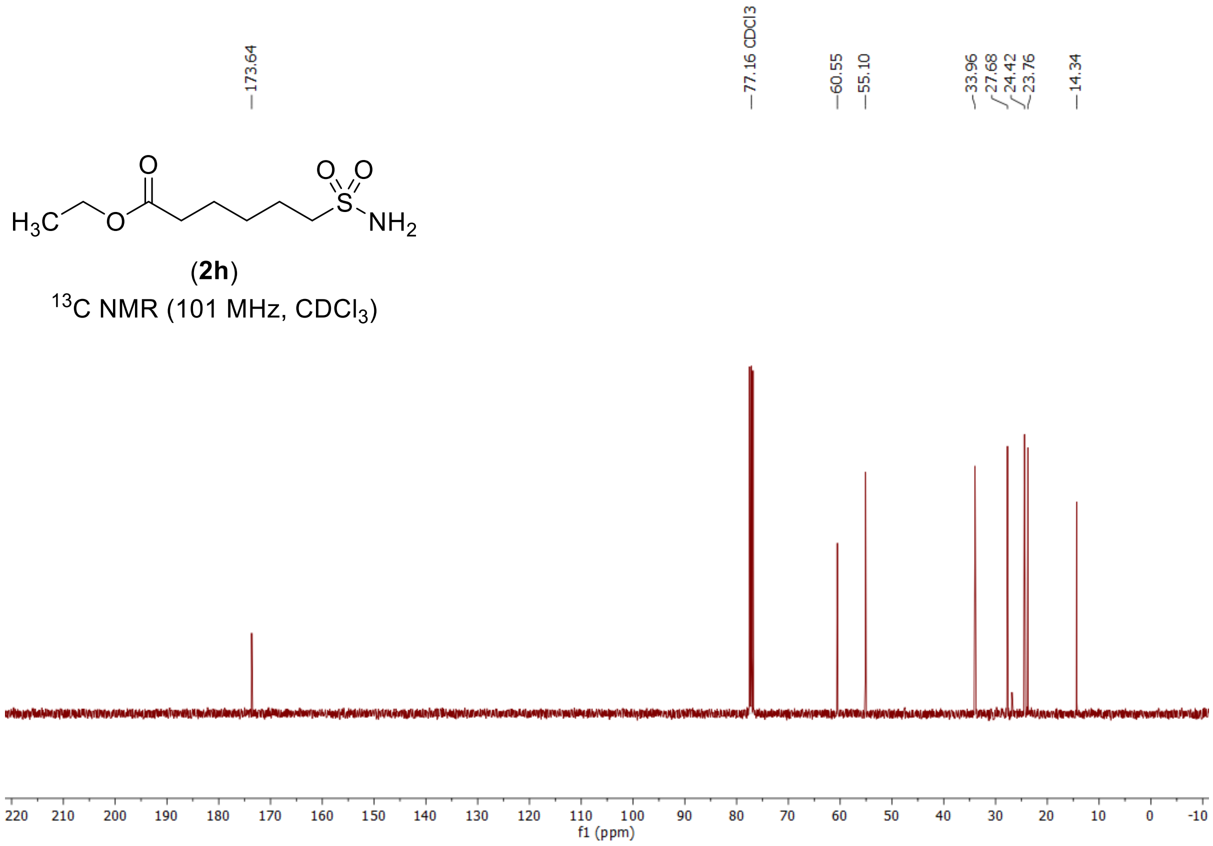

(**2h**)
^{13}C NMR (101 MHz, $CDCl_3$)

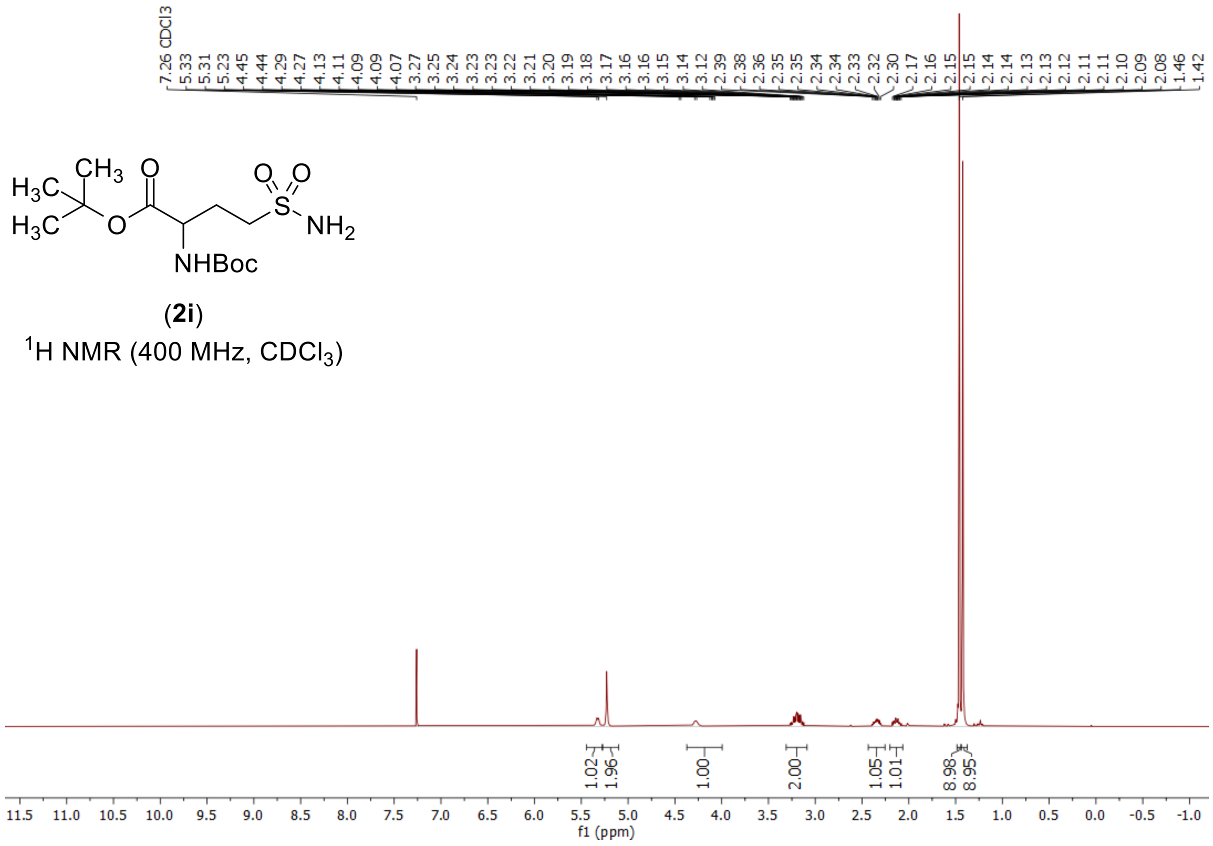

(**2i**)
^{1}H NMR (400 MHz, $CDCl_3$)

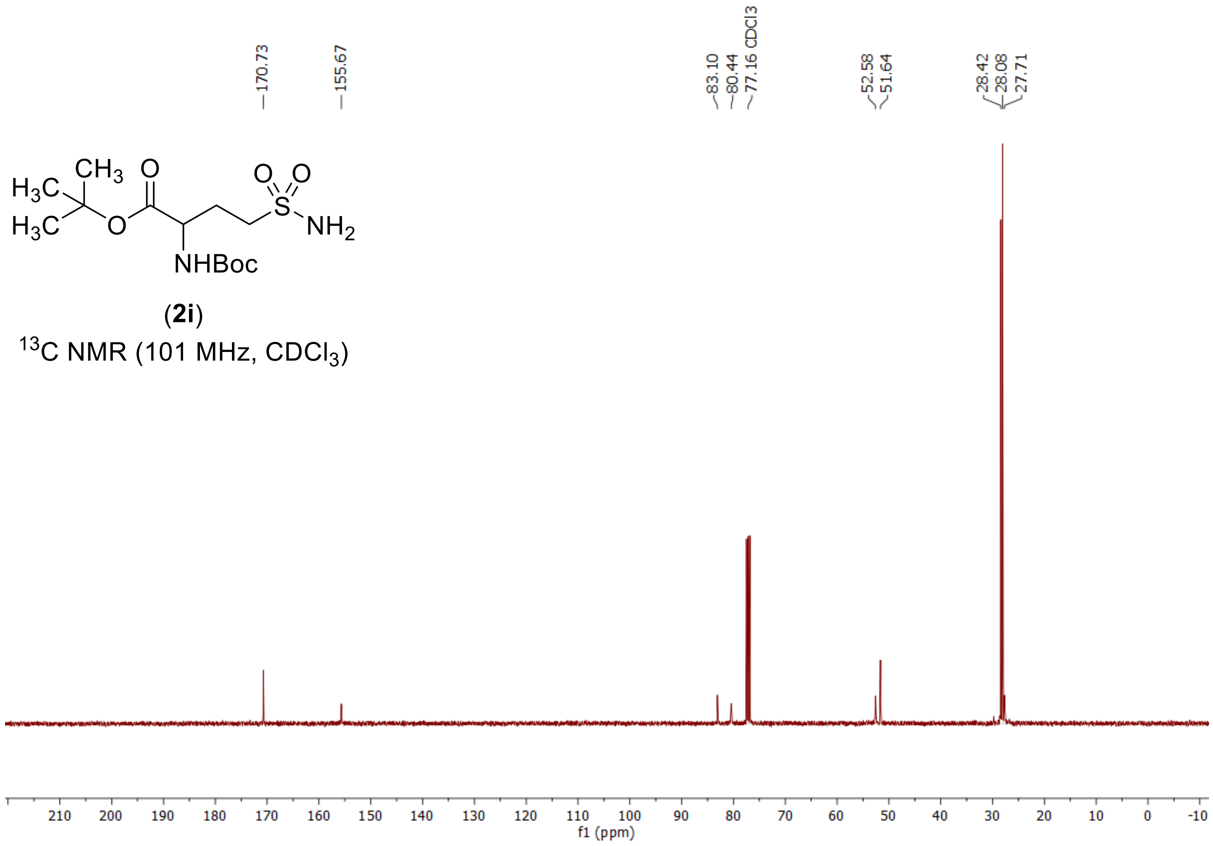

(**2i**)
^{13}C NMR (101 MHz, $CDCl_3$)

(**3a**)

^{1}H NMR (400 MHz, $CDCl_3$)

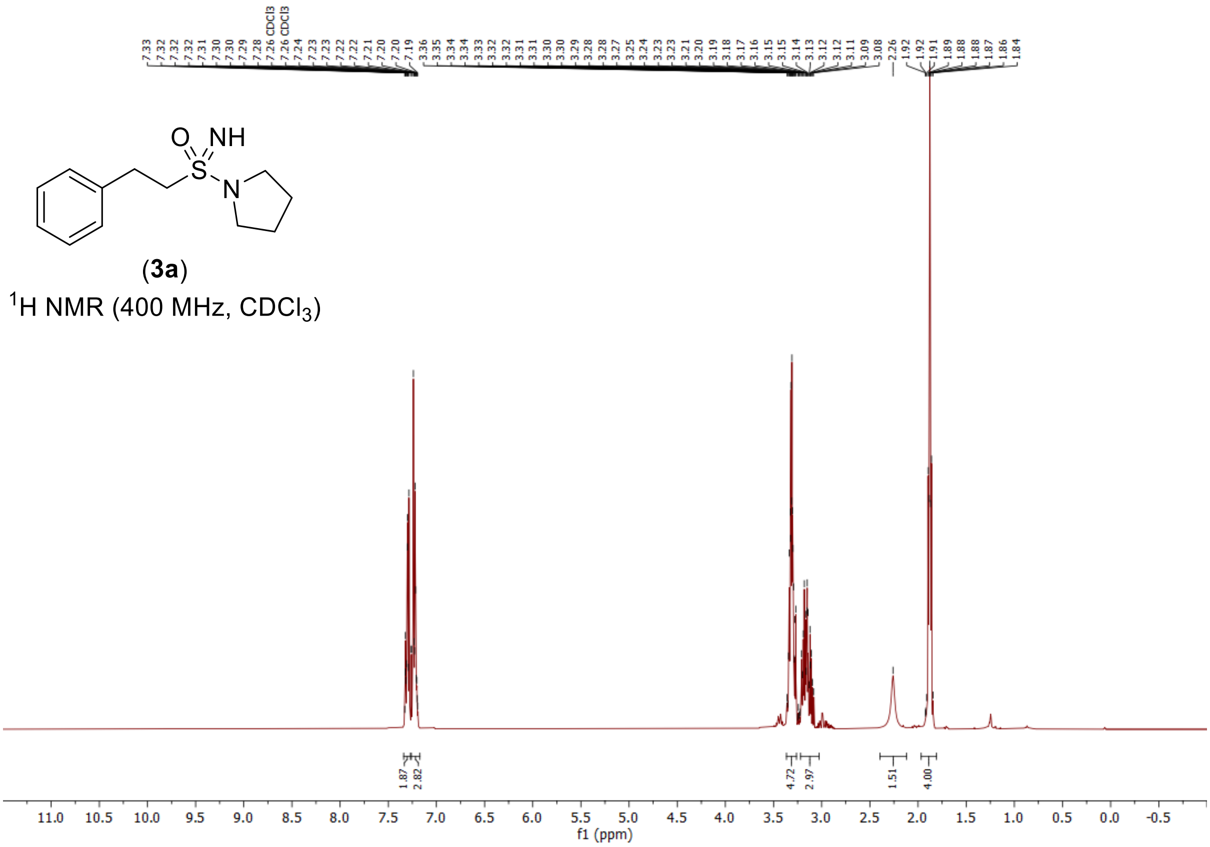

(**3a**)

^{13}C NMR (101 MHz, $CDCl_3$)

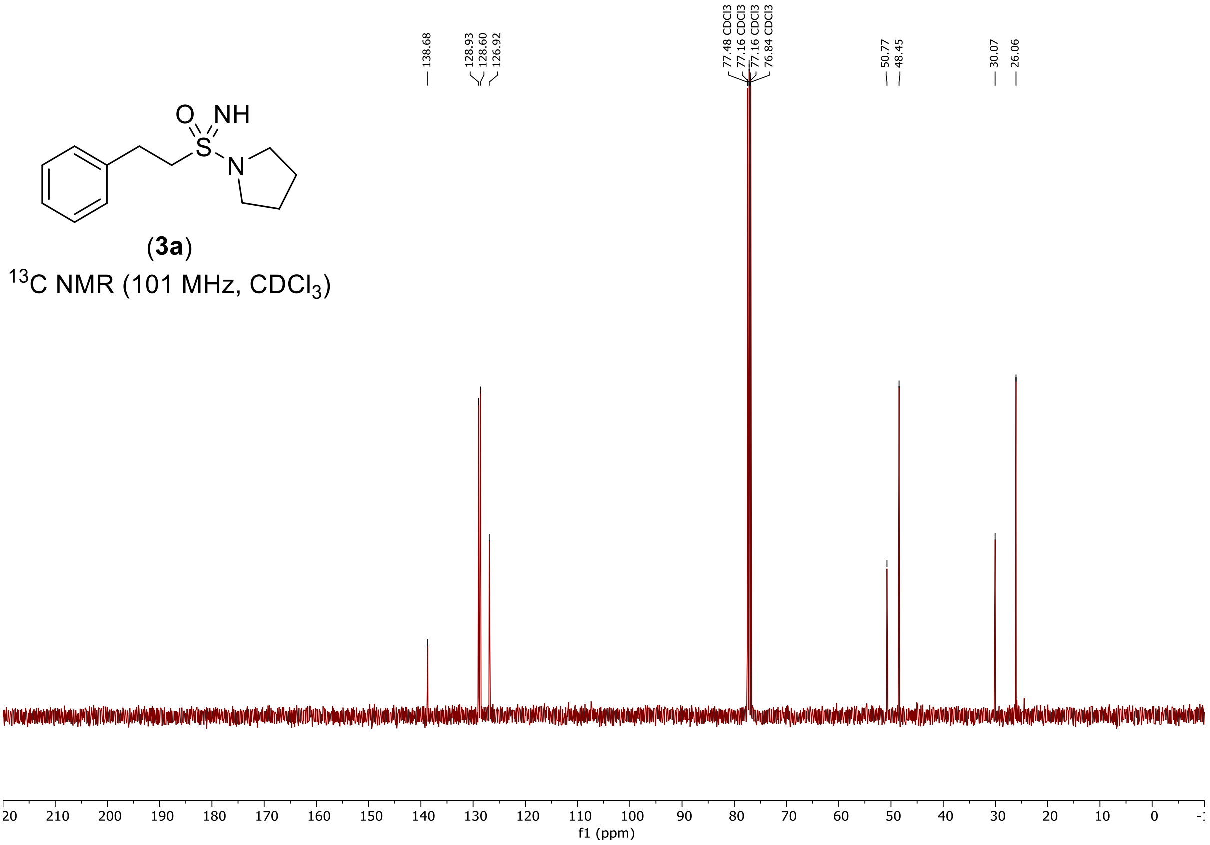

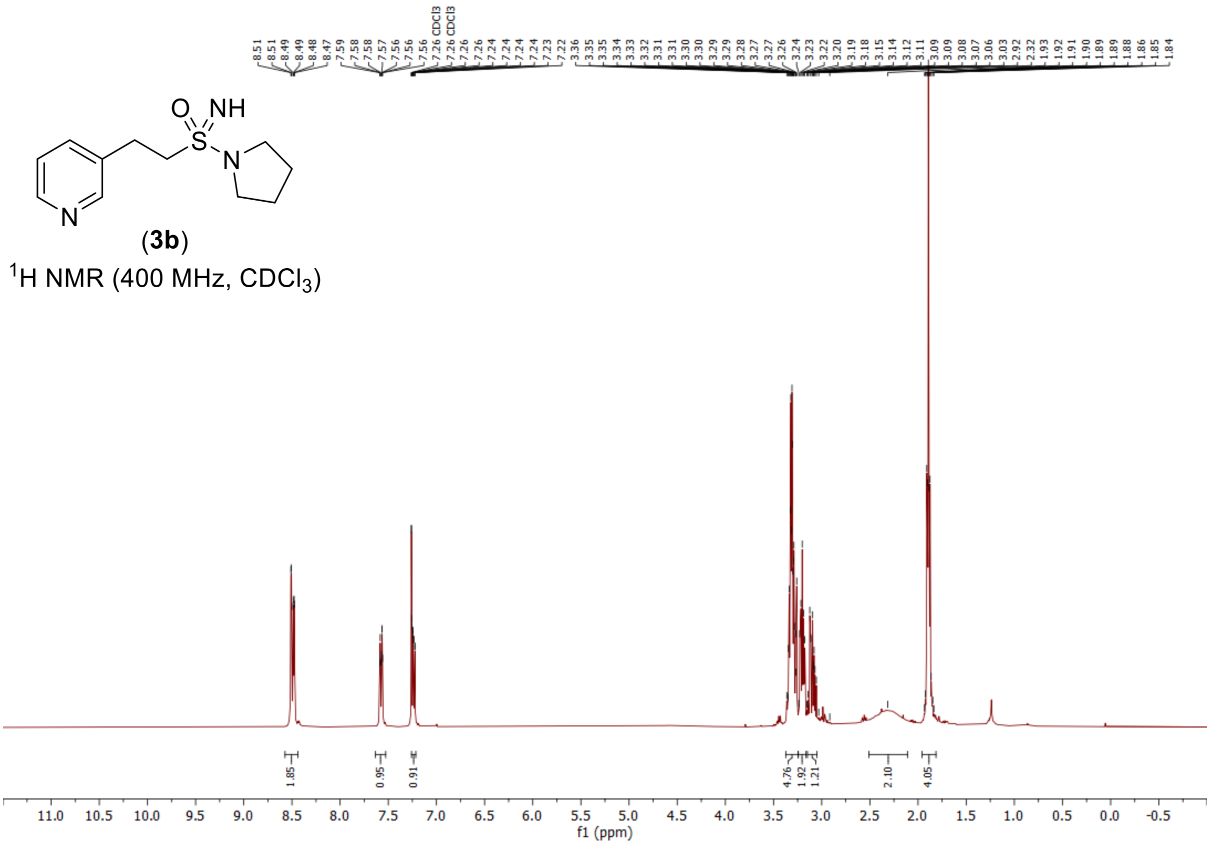

(3b)
1H NMR (400 MHz, CDCl3)
8.51 8.51 8.49 8.49 8.48 8.47 7.59 7.58 7.58 7.57 7.56 7.56 7.26 CDCl3 7.26 CDCl3 7.26 7.26 7.24 7.24 7.24 7.24 7.23 7.22 3.36 3.35 3.35 3.34 3.33 3.32 3.31 3.31 3.30 3.30 3.29 3.29 3.28 3.27 3.27 3.26 3.24 3.23 3.22 3.20 3.19 3.18 3.15 3.14 3.12 3.11 3.09 3.09 3.08 3.07 3.06 3.03 2.92 2.32 1.93 1.92 1.91 1.90 1.89 1.89 1.88 1.86 1.85 1.84
1.85
0.95
0.91
4.76
1.92
1.21
2.10
4.05
11.0 10.5 10.0 9.5 9.0 8.5 8.0 7.5 7.0 6.5 6.0 5.5 5.0 4.5 4.0 3.5 3.0 2.5 2.0 1.5 1.0 0.5 0.0 -0.5
f1 (ppm)

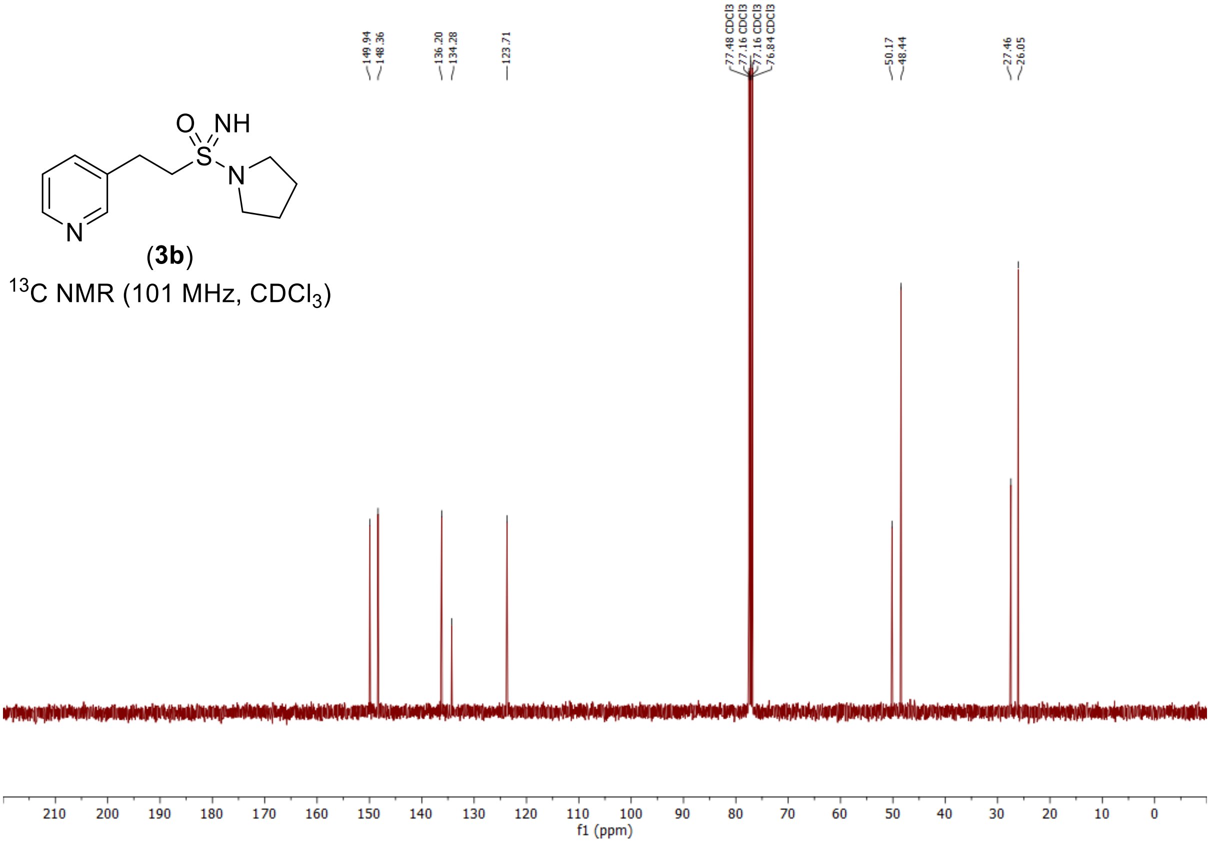

(3b)
13C NMR (101 MHz, CDCl3)
149.94
148.36
136.20
134.28
123.71
77.48 CDCl3
77.16 CDCl3
77.16 CDCl3
76.84 CDCl3
50.17
48.44
27.46
26.05
210 200 190 180 170 160 150 140 130 120 110 100 90 80 70 60 50 40 30 20 10 0
f1 (ppm)

(**3c**)

^{1}H NMR (400 MHz, $CDCl_3$)

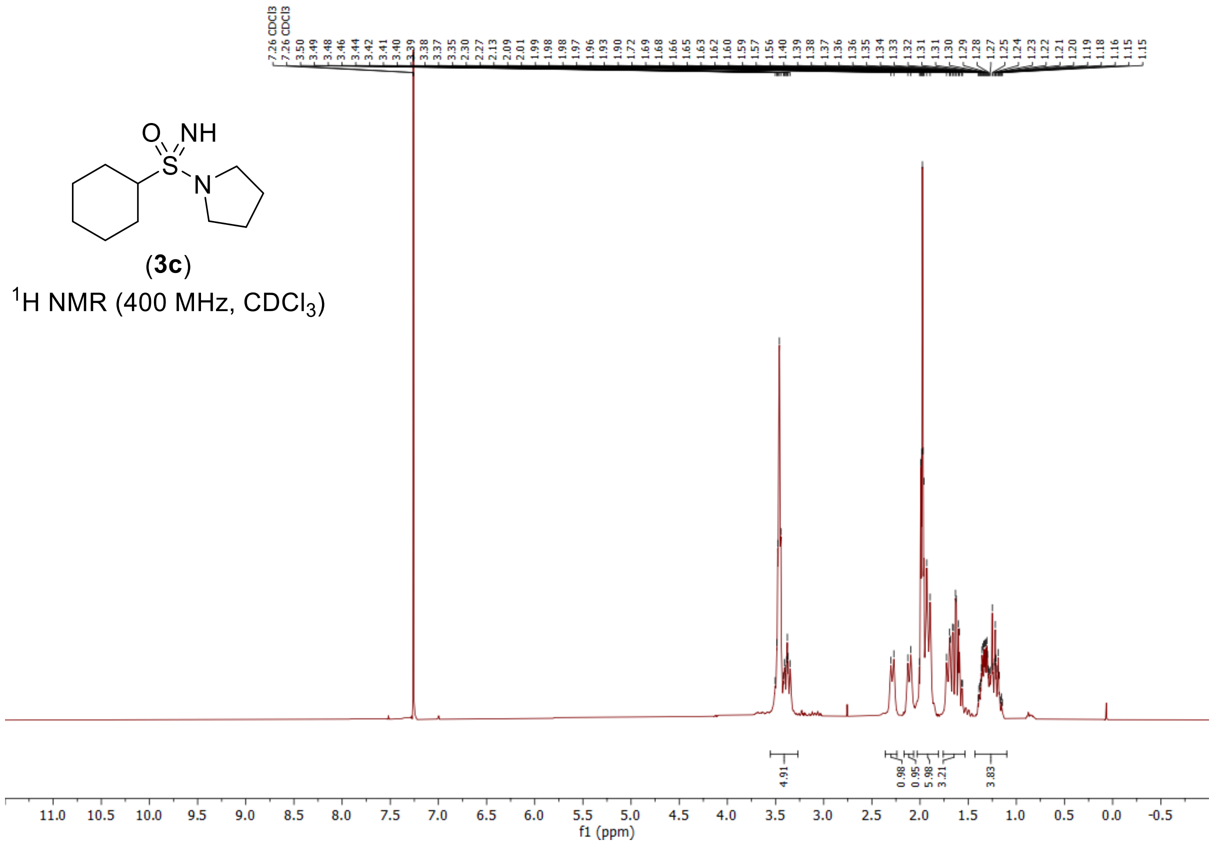

(**3c**)

^{13}C NMR (101 MHz, $CDCl_3$)

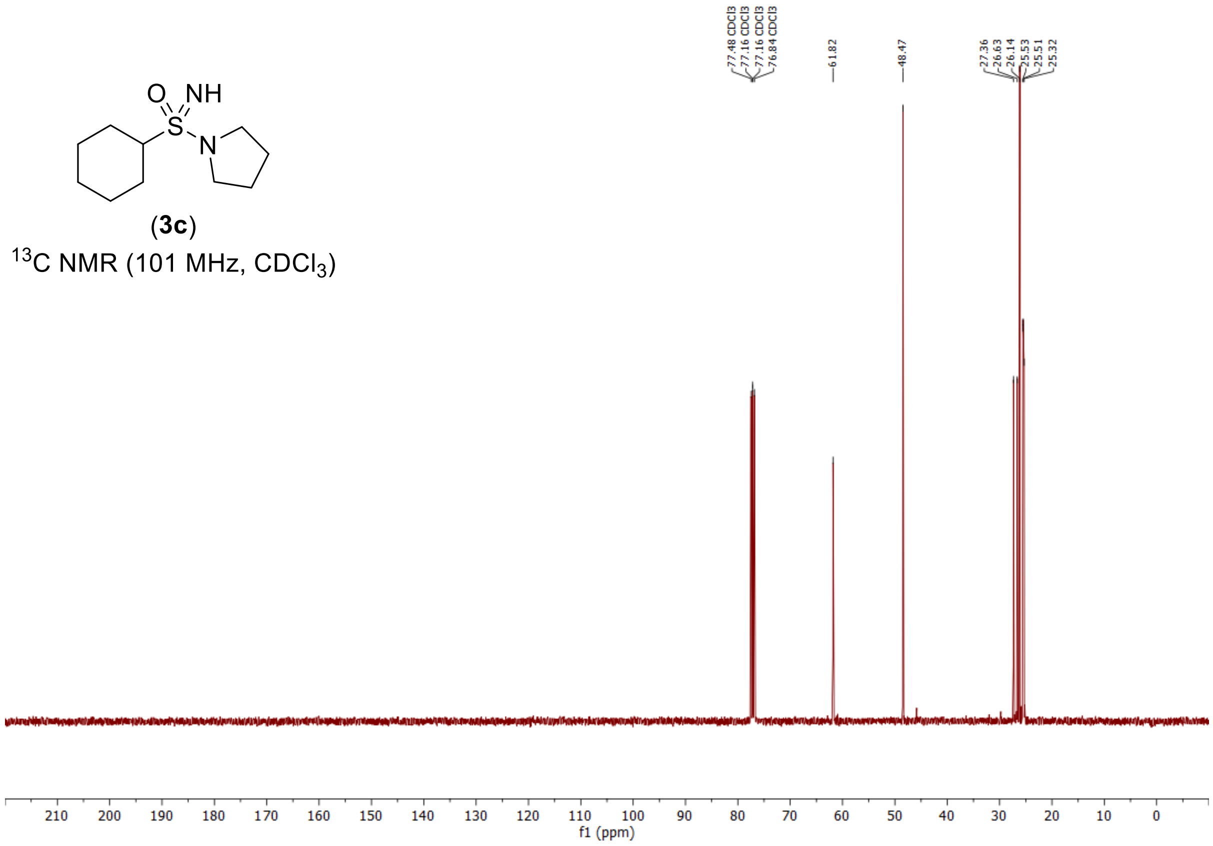

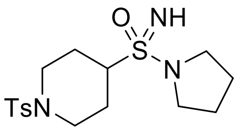

(**3d**)
^{1}H NMR (400 MHz, $CDCl_3$)

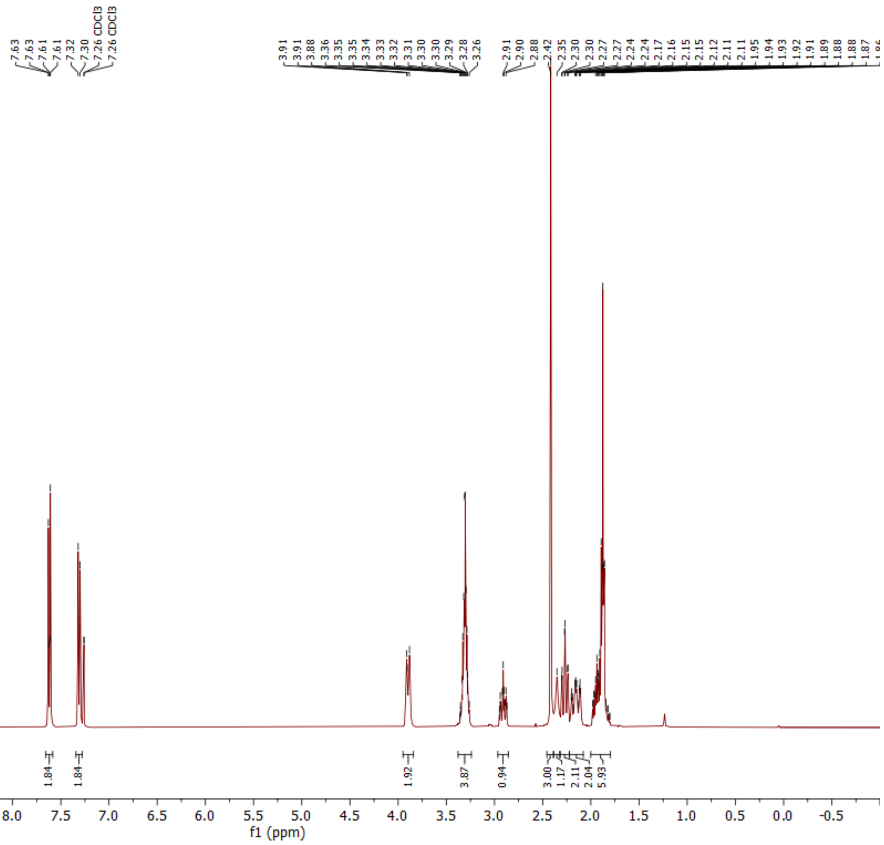

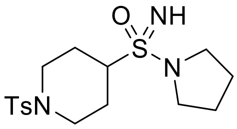

(**3d**)
^{13}C NMR (101 MHz, $CDCl_3$)

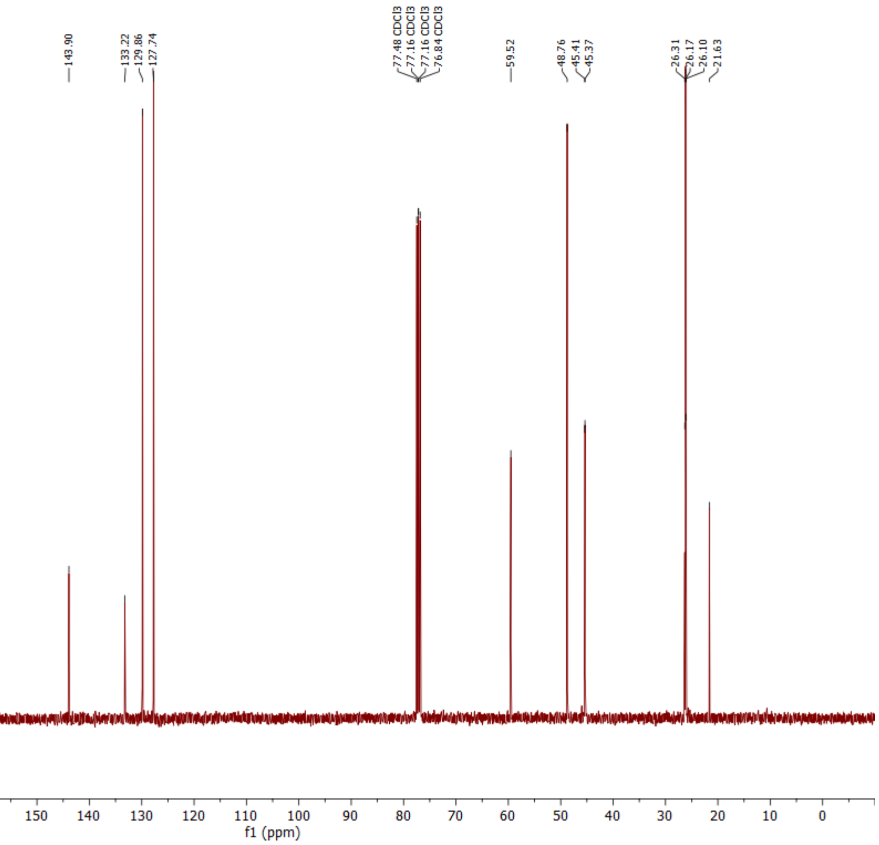

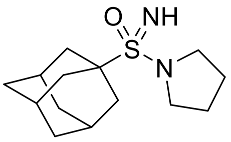

(**3e**)

^{1}H NMR (400 MHz, $CDCl_3$)

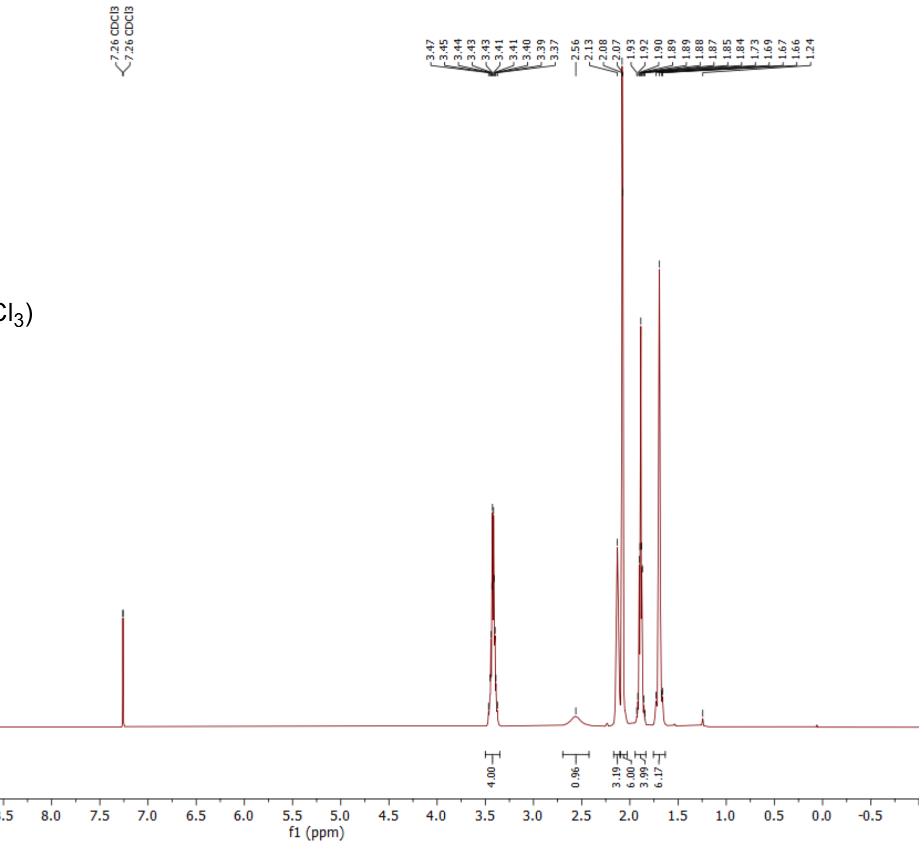

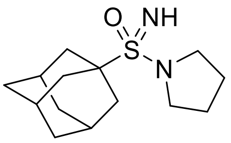

(**3e**)

^{13}C NMR (101 MHz, $CDCl_3$)

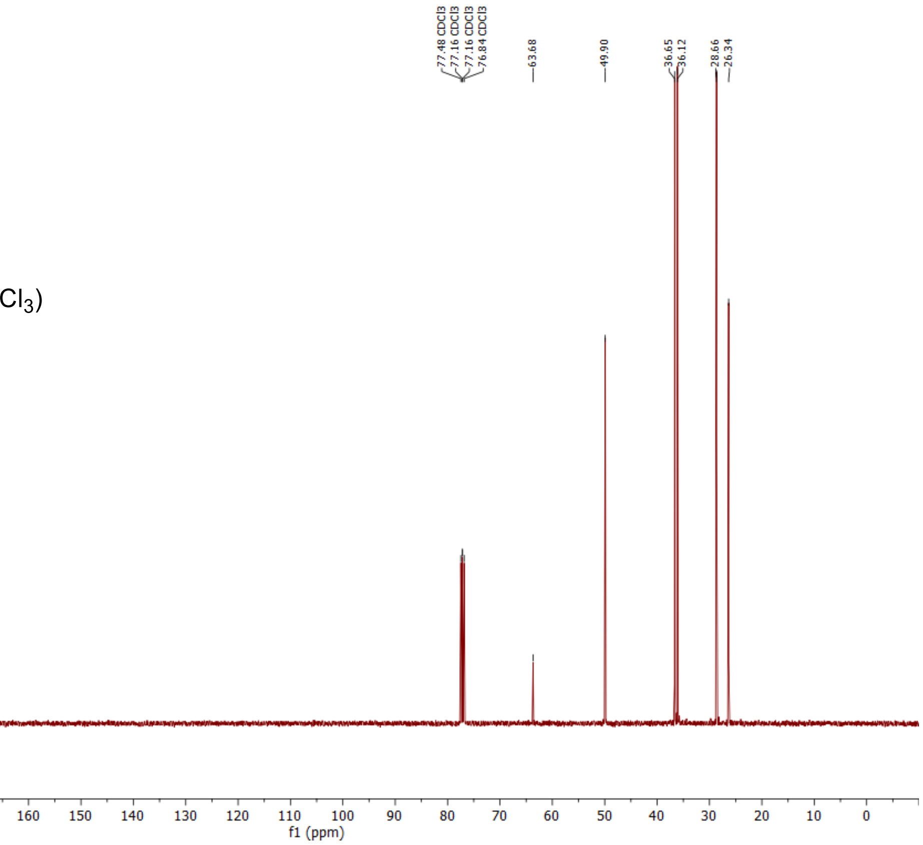

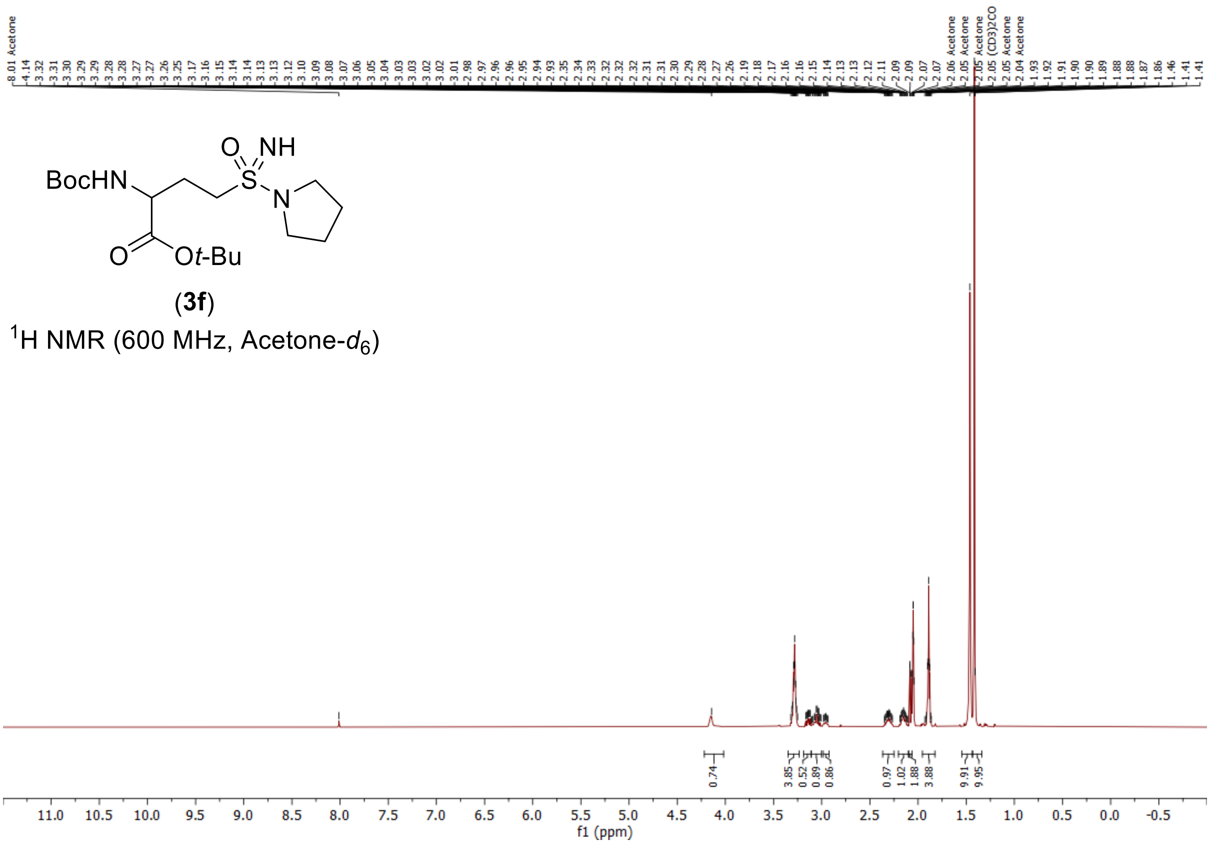

(**3f**)
^{1}H NMR (600 MHz, Acetone-d_6)

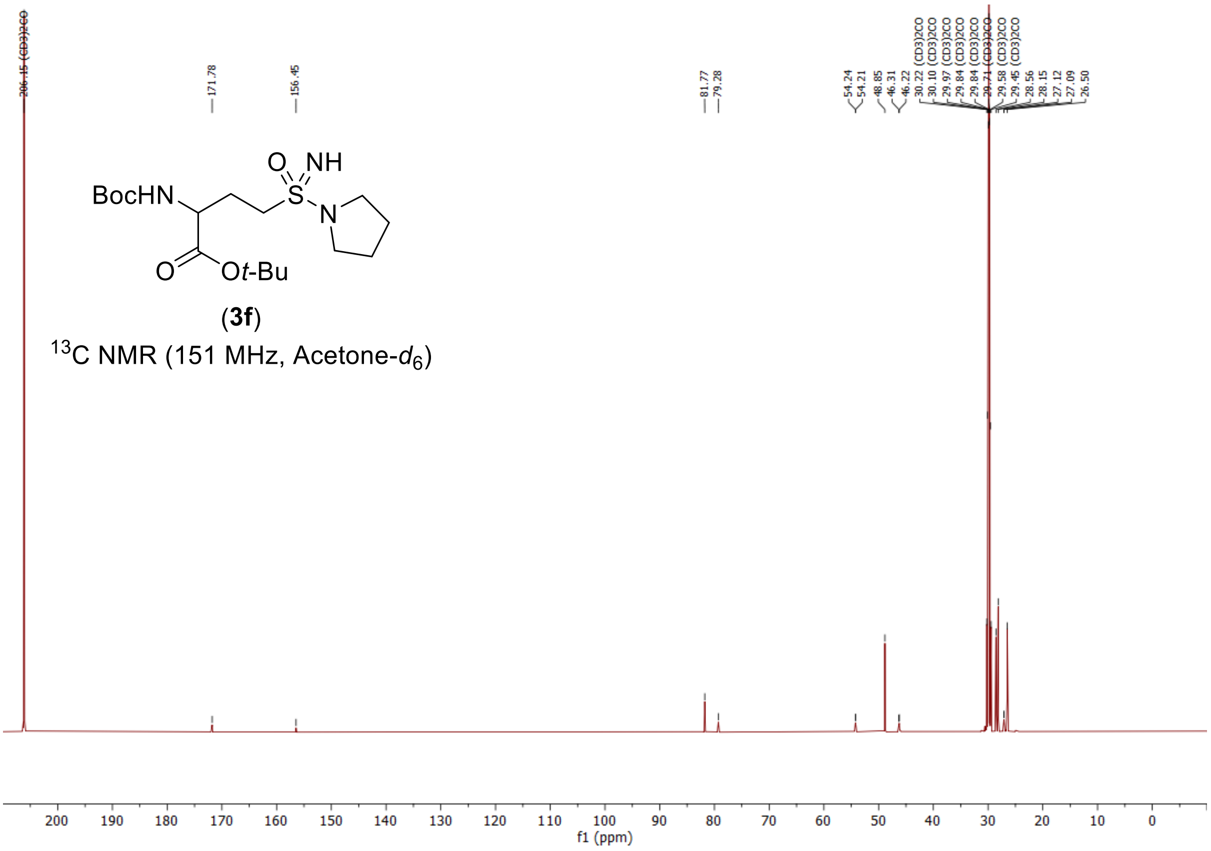

(**3f**)
^{13}C NMR (151 MHz, Acetone-d_6)

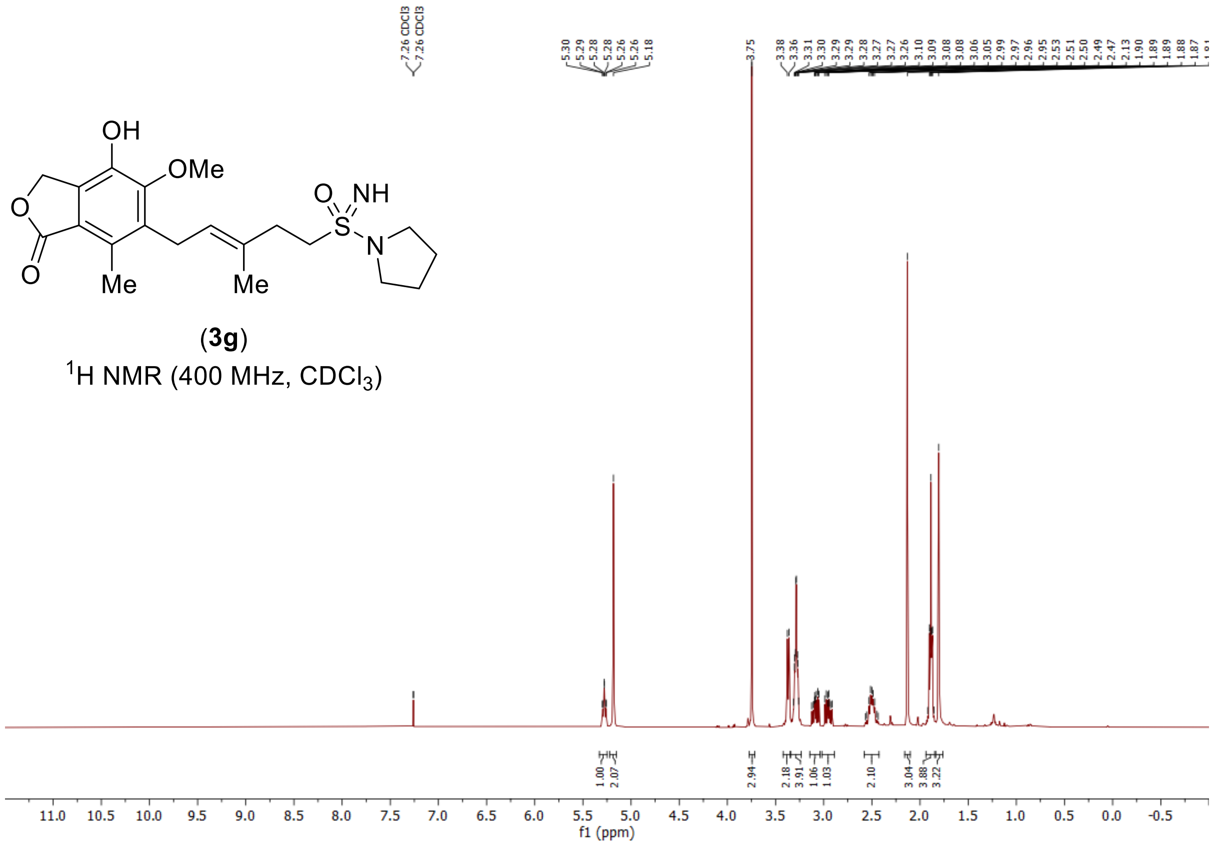

(**3g**)

^{1}H NMR (400 MHz, $CDCl_3$)

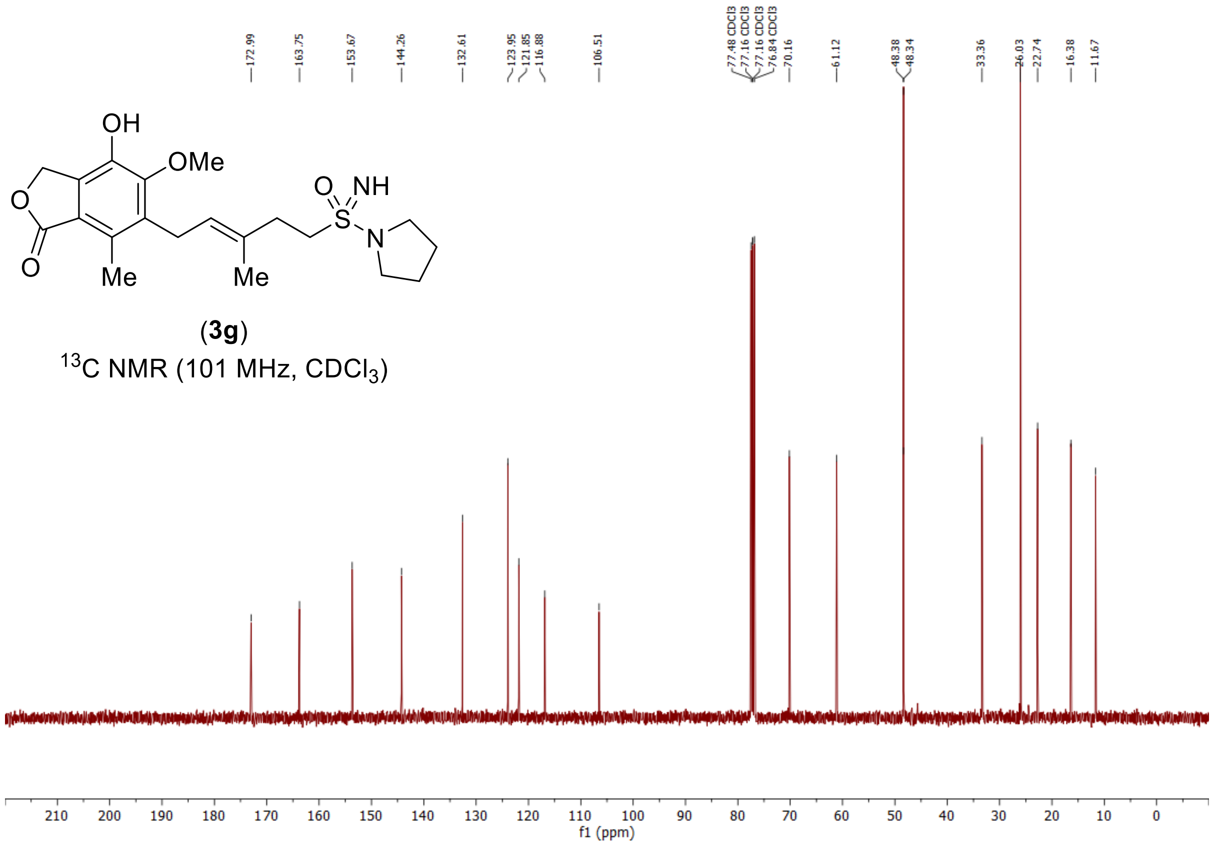

(**3g**)

^{13}C NMR (101 MHz, $CDCl_3$)

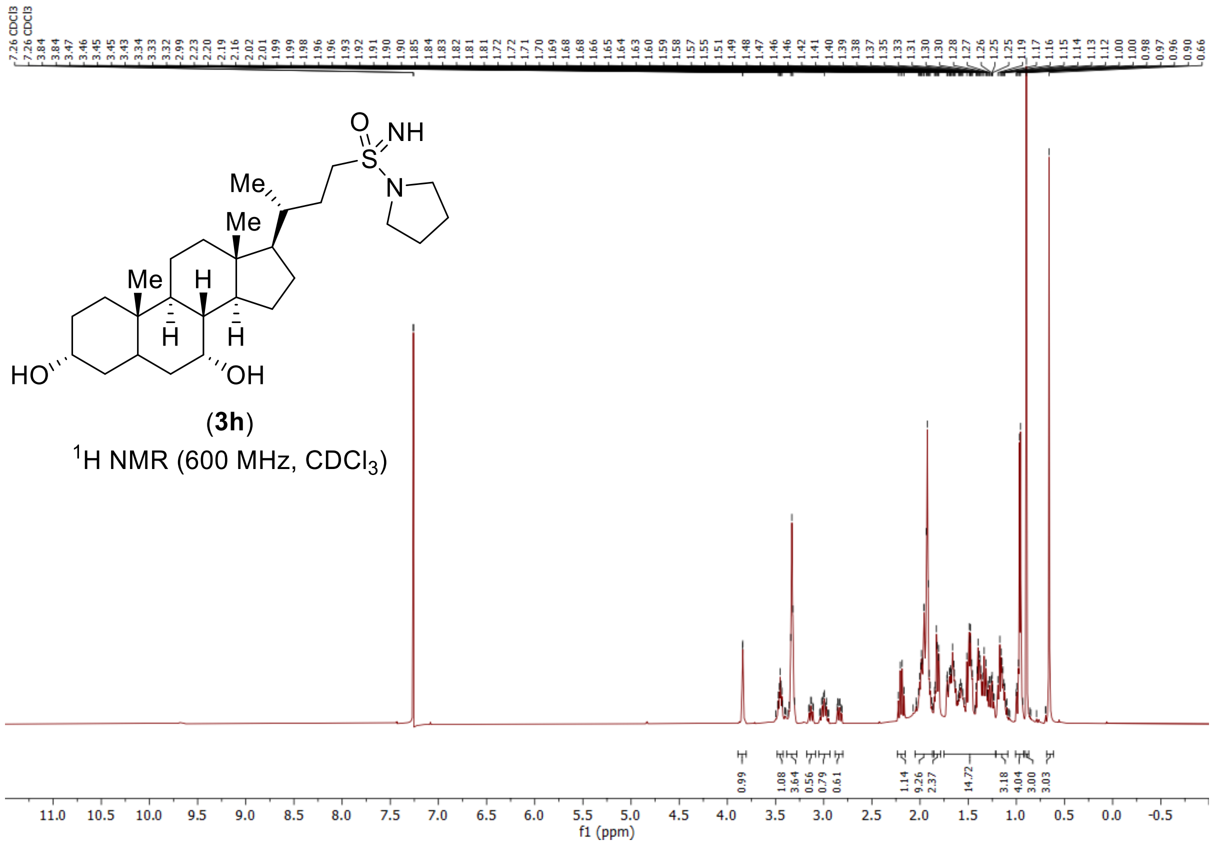

(**3h**)
^{1}H NMR (600 MHz, $CDCl_3$)

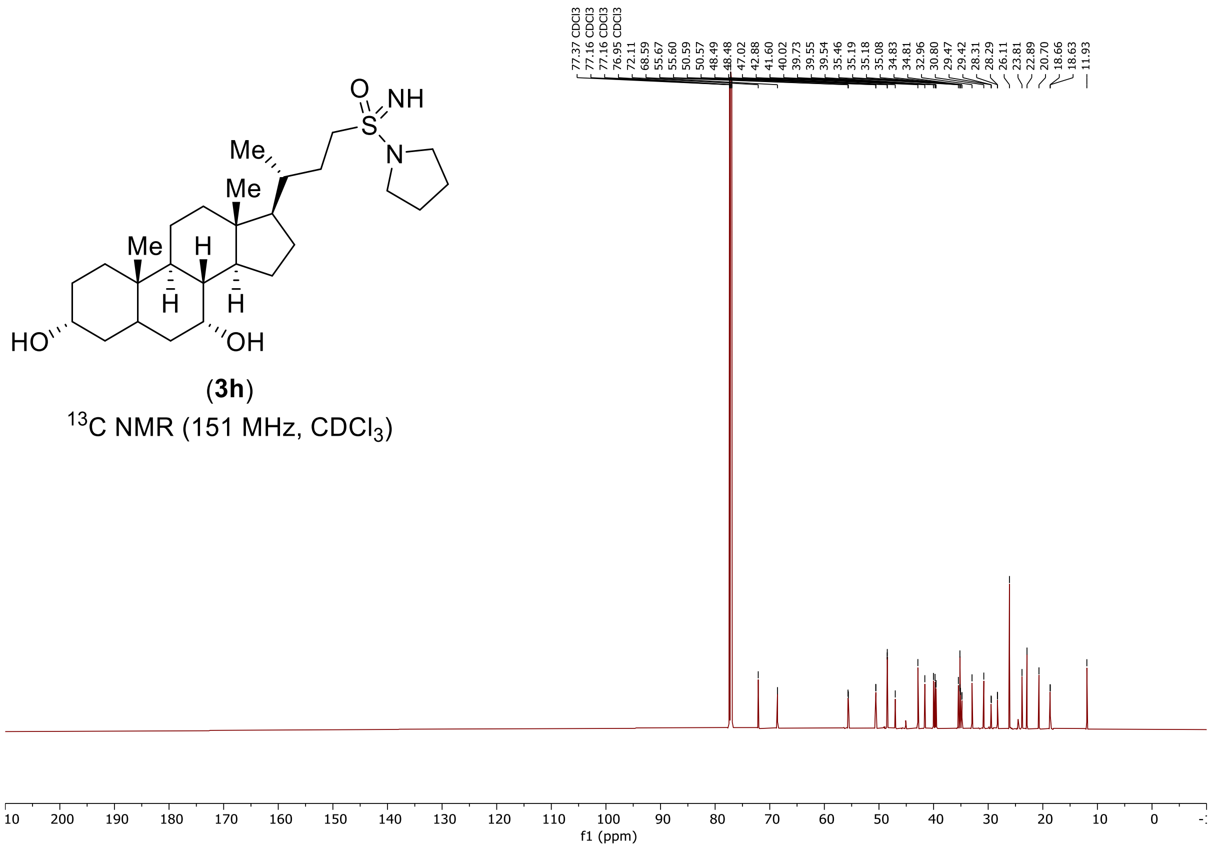

(**3h**)
^{13}C NMR (151 MHz, $CDCl_3$)

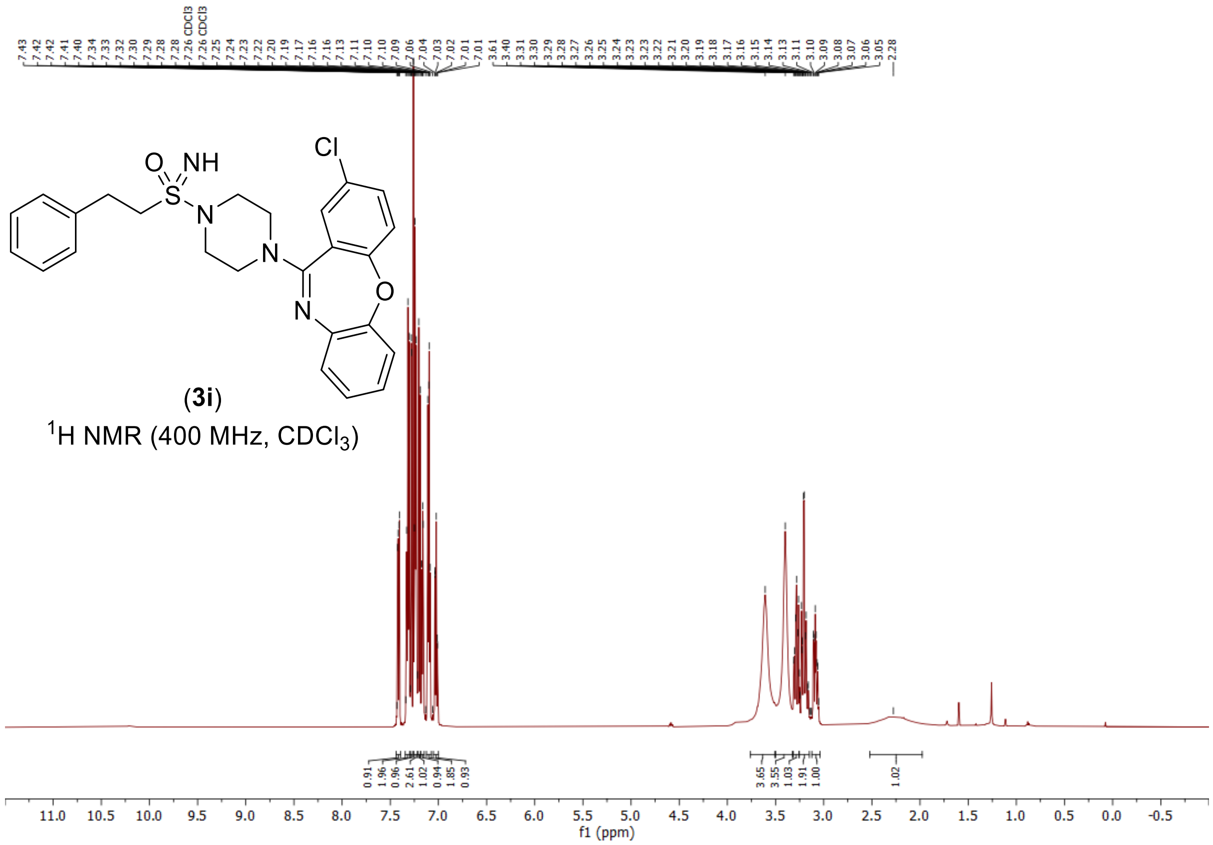
7.43 7.42 7.41 7.40 7.34 7.33 7.32 7.30 7.29 7.28 7.26 CDCl3 7.26 CDCl3 7.25 7.24 7.23 7.22 7.20 7.19 7.17 7.16 7.16 7.13 7.11 7.10 7.10 7.09 7.06 7.04 7.03 7.02 7.01 3.61 3.40 3.31 3.30 3.29 3.28 3.27 3.26 3.25 3.24 3.23 3.23 3.22 3.21 3.20 3.19 3.18 3.17 3.16 3.15 3.14 3.13 3.11 3.10 3.09 3.08 3.07 3.06 3.05 2.28
Cl
O
NH
S
N
N
N
O
(3i)
^{1}H NMR (400 MHz, $CDCl_3$)
0.91 1.96 0.96 2.61 1.02 0.94 1.85 0.93
3.65 3.55 1.03 1.91 1.00
1.02
f1 (ppm)

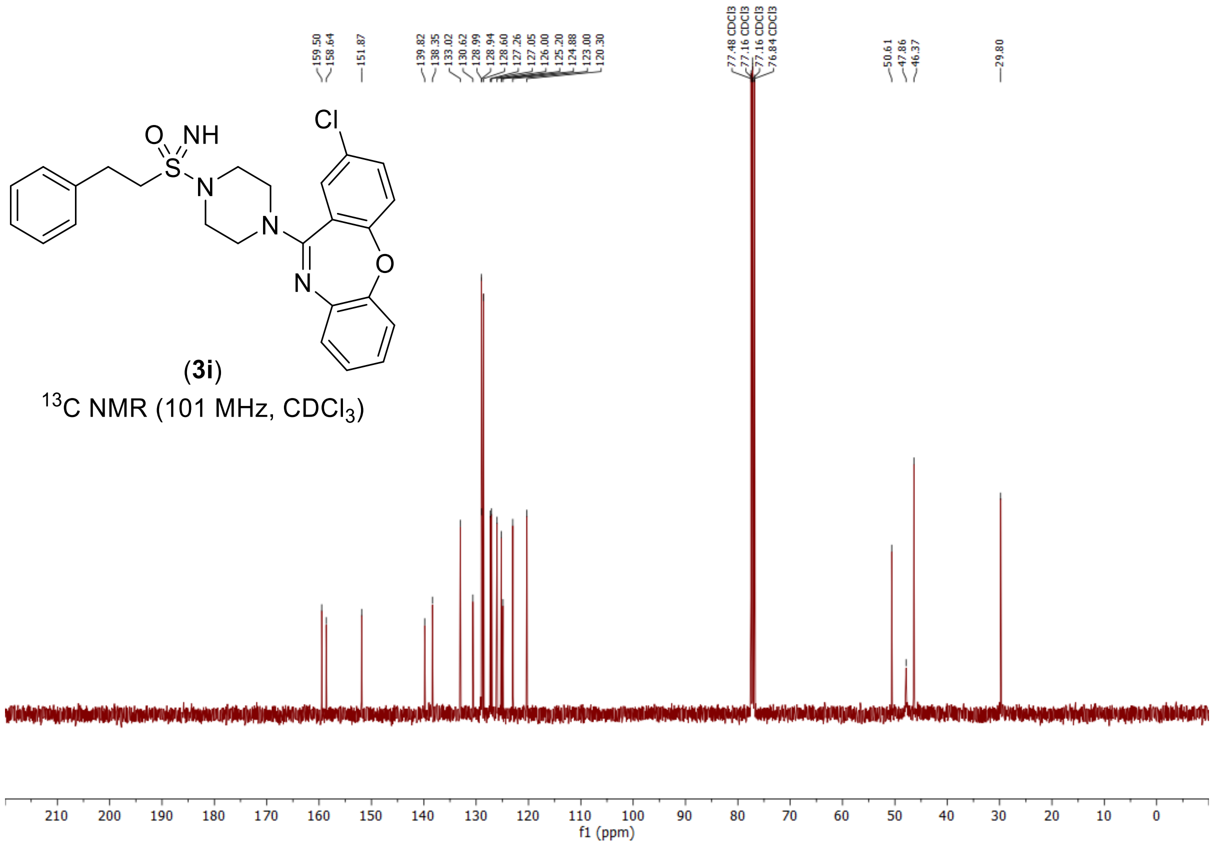
159.50 158.64 151.87 139.82 138.35 133.02 130.62 128.99 128.94 128.60 127.26 127.05 126.00 125.20 124.88 123.00 120.30
77.48 CDCl3 77.16 CDCl3 77.16 CDCl3 76.84 CDCl3
50.61 47.86 46.37 29.80
Cl
O
NH
S
N
N
N
O
(3i)
^{13}C NMR (101 MHz, $CDCl_3$)
f1 (ppm)

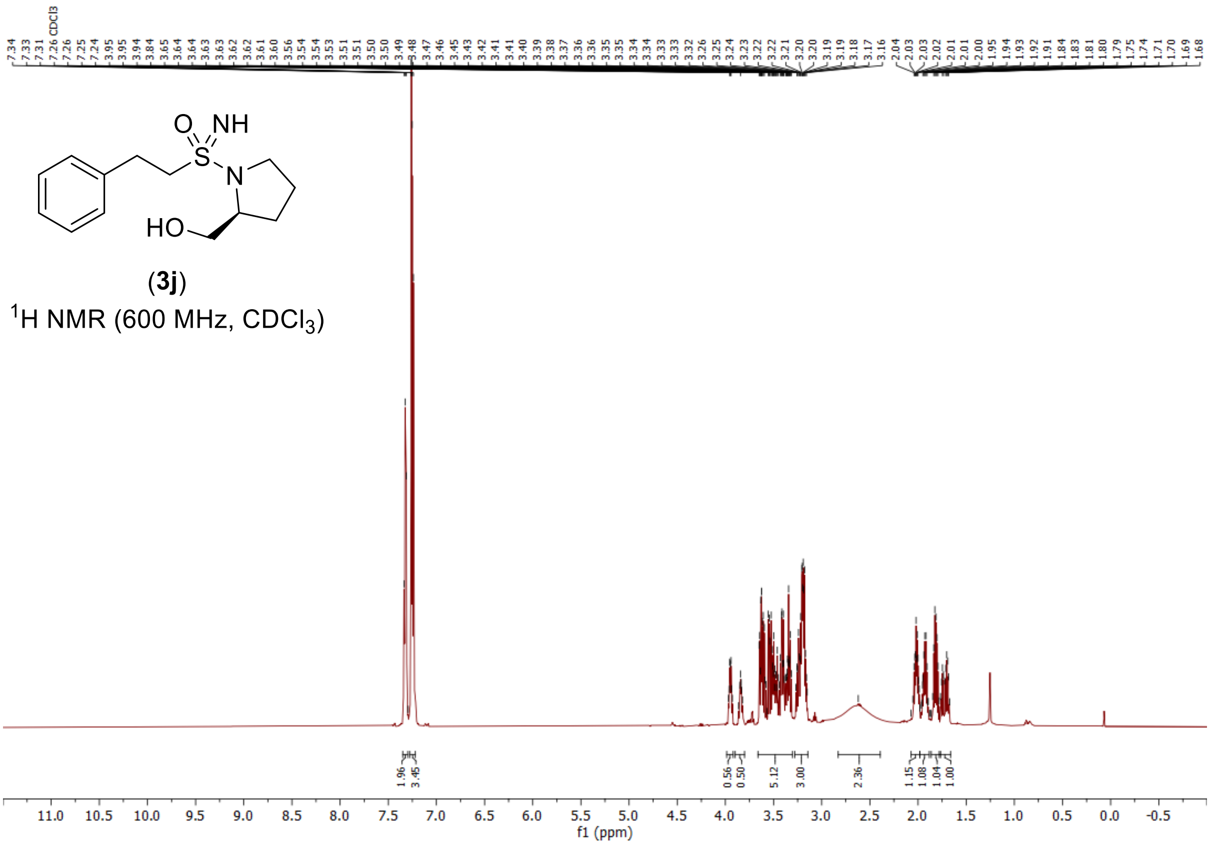
7.34 7.33 7.31 7.26 CDCl3 7.26 7.25 7.24 3.95 3.95 3.94 3.84 3.65 3.64 3.64 3.63 3.63 3.62 3.62 3.61 3.60 3.56 3.54 3.54 3.53 3.51 3.51 3.50 3.50 3.49 3.48 3.47 3.46 3.45 3.43 3.42 3.41 3.41 3.40 3.39 3.38 3.37 3.36 3.36 3.35 3.35 3.34 3.34 3.33 3.33 3.32 3.26 3.25 3.24 3.23 3.22 3.22 3.21 3.20 3.20 3.19 3.19 3.18 3.17 3.16 2.04 2.03 2.03 2.02 2.02 2.01 2.01 2.00 1.95 1.94 1.93 1.92 1.91 1.84 1.83 1.81 1.80 1.79 1.75 1.74 1.71 1.70 1.69 1.68
O NH
S
N
HO
(3j)
^{1}H NMR (600 MHz, $CDCl_3$)
1.96 3.45 0.56 0.50 5.12 3.00 2.36 1.15 1.08 1.04 1.00
11.0 10.5 10.0 9.5 9.0 8.5 8.0 7.5 7.0 6.5 6.0 5.5 5.0 4.5 4.0 3.5 3.0 2.5 2.0 1.5 1.0 0.5 0.0 -0.5
f1 (ppm)

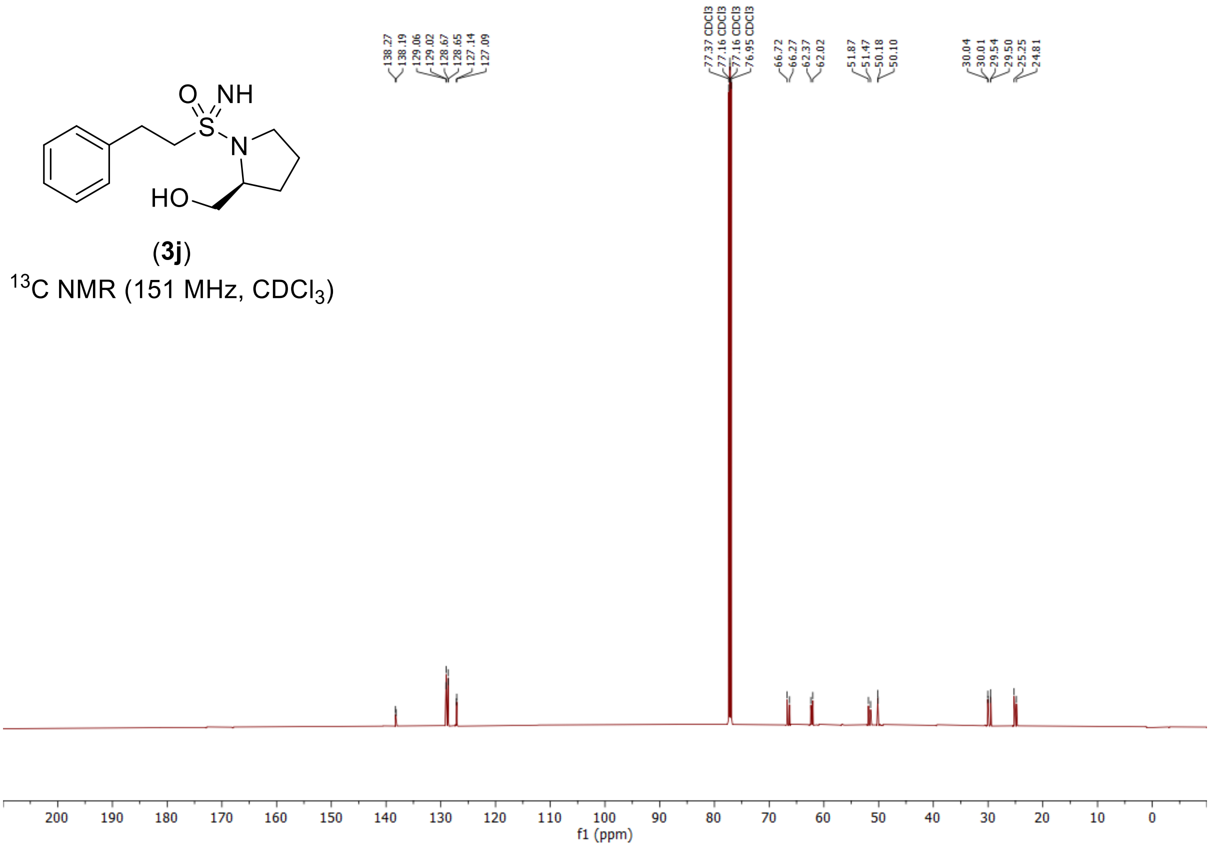
138.27 138.19 129.06 129.02 128.67 128.65 127.14 127.09
77.37 CDCl3 77.16 CDCl3 77.16 CDCl3 76.95 CDCl3
66.72 66.27 62.37 62.02 51.87 51.47 50.18 50.10 30.04 30.01 29.54 29.50 25.25 24.81
O NH
S
N
HO
(3j)
^{13}C NMR (151 MHz, $CDCl_3$)
200 190 180 170 160 150 140 130 120 110 100 90 80 70 60 50 40 30 20 10 0
f1 (ppm)

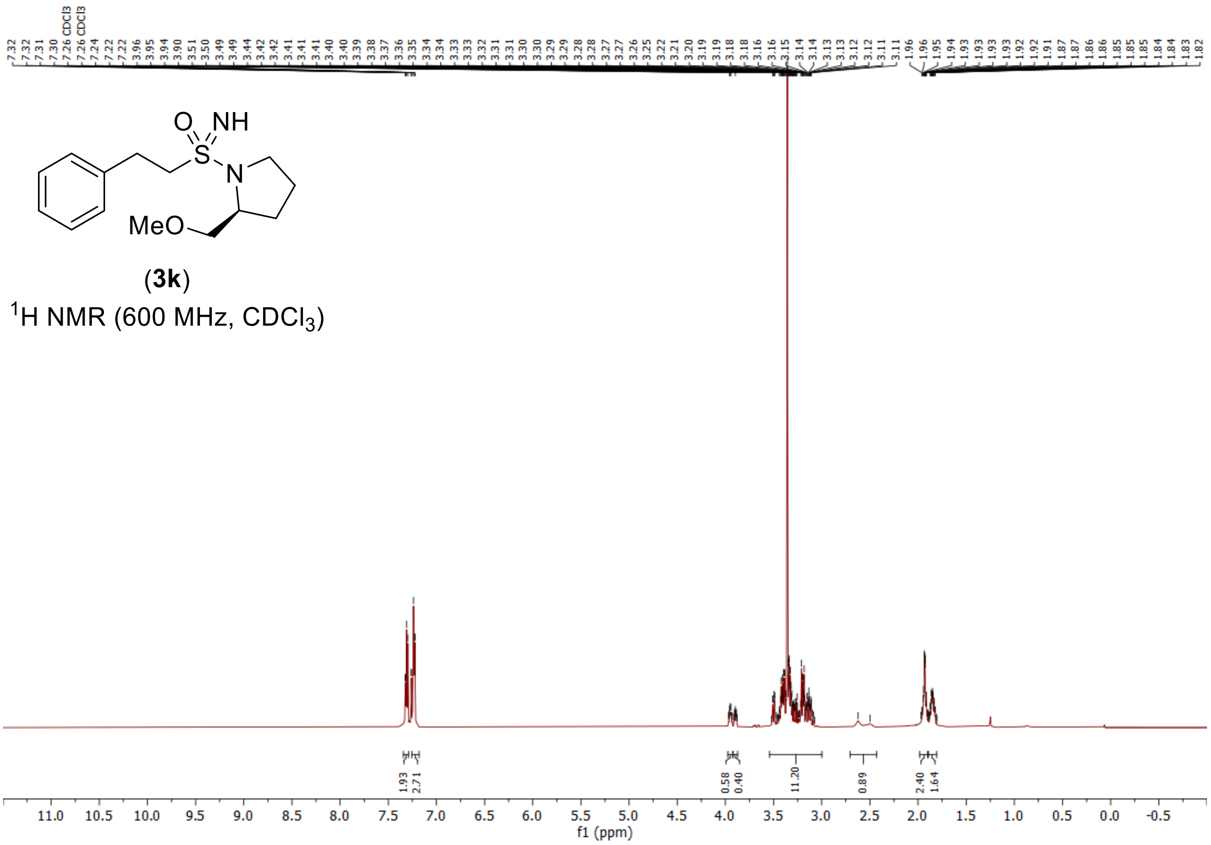

(**3k**)
^{1}H NMR (600 MHz, $CDCl_3$)

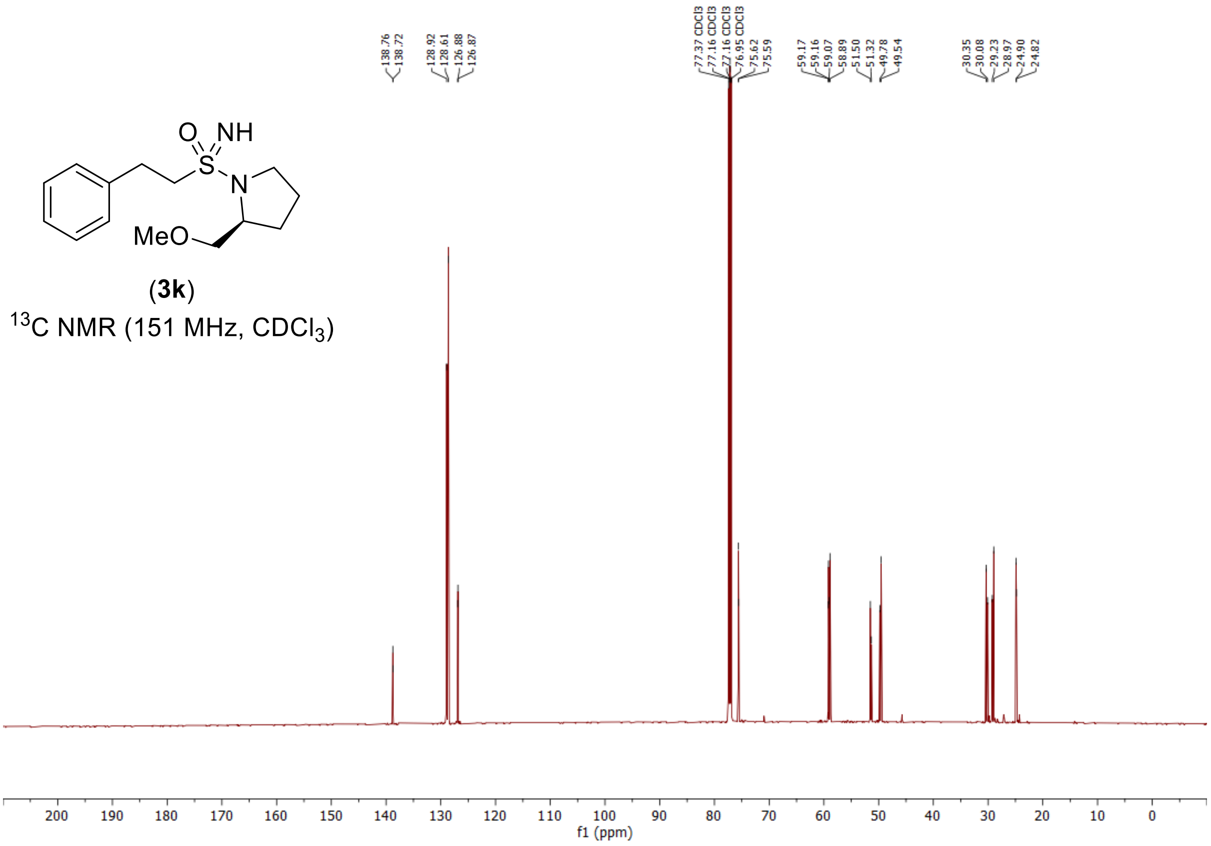

(**3k**)
^{13}C NMR (151 MHz, $CDCl_3$)

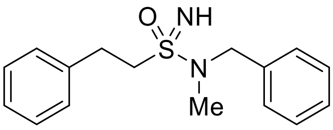

(**3m**)

^{1}H NMR (400 MHz, $CDCl_3$)

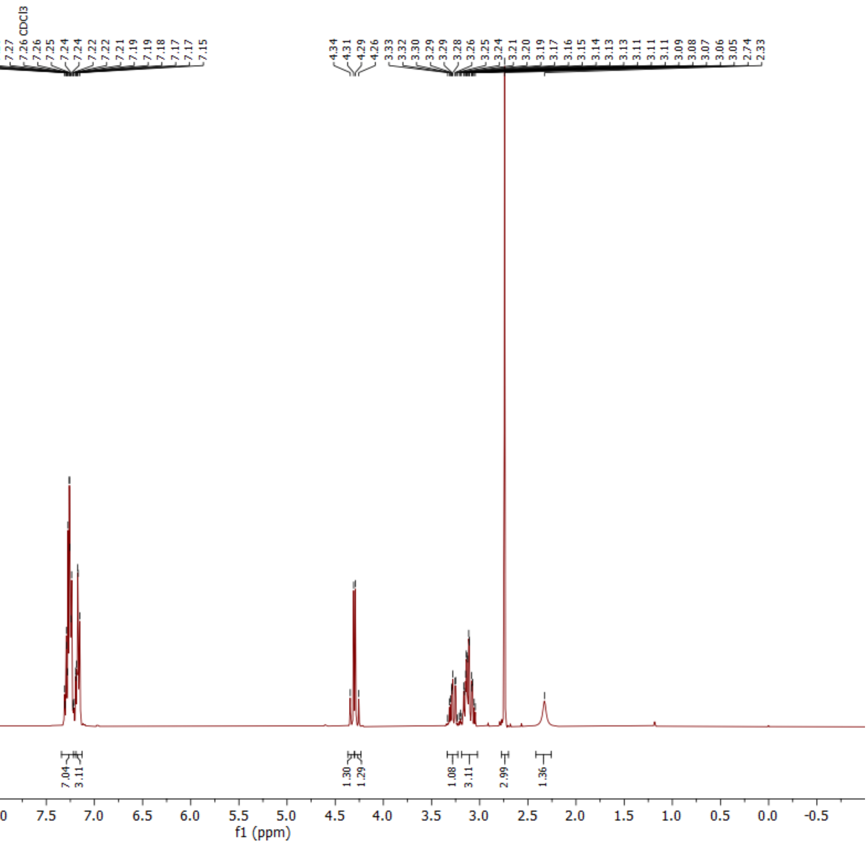

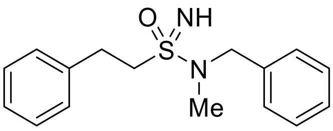

(**3m**)

^{13}C NMR (101 MHz, $CDCl_3$)

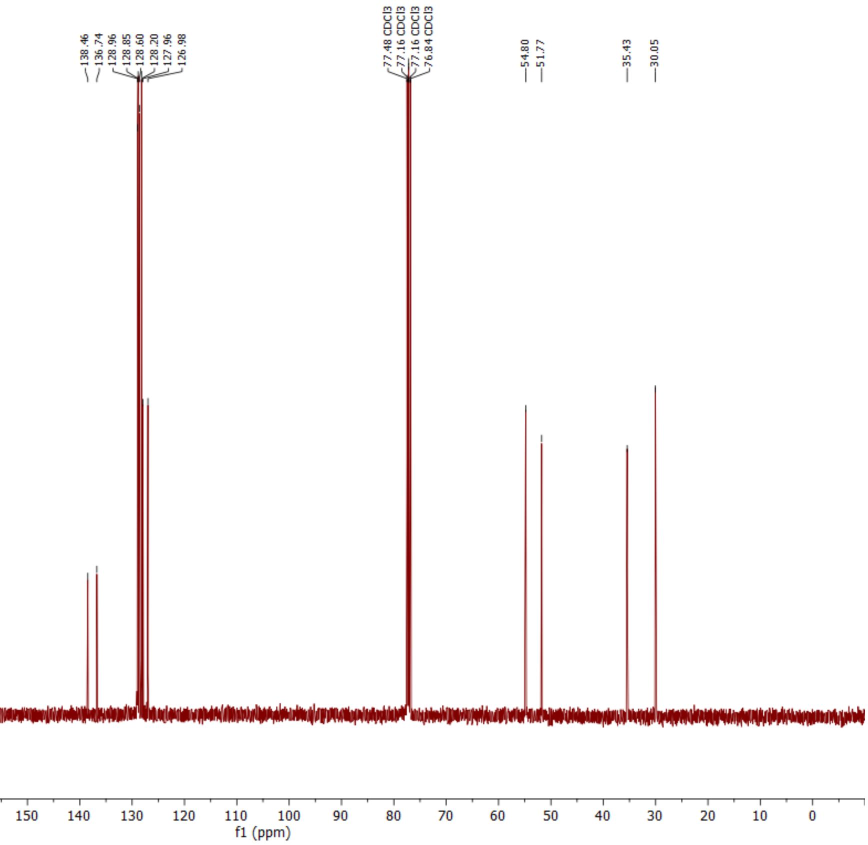

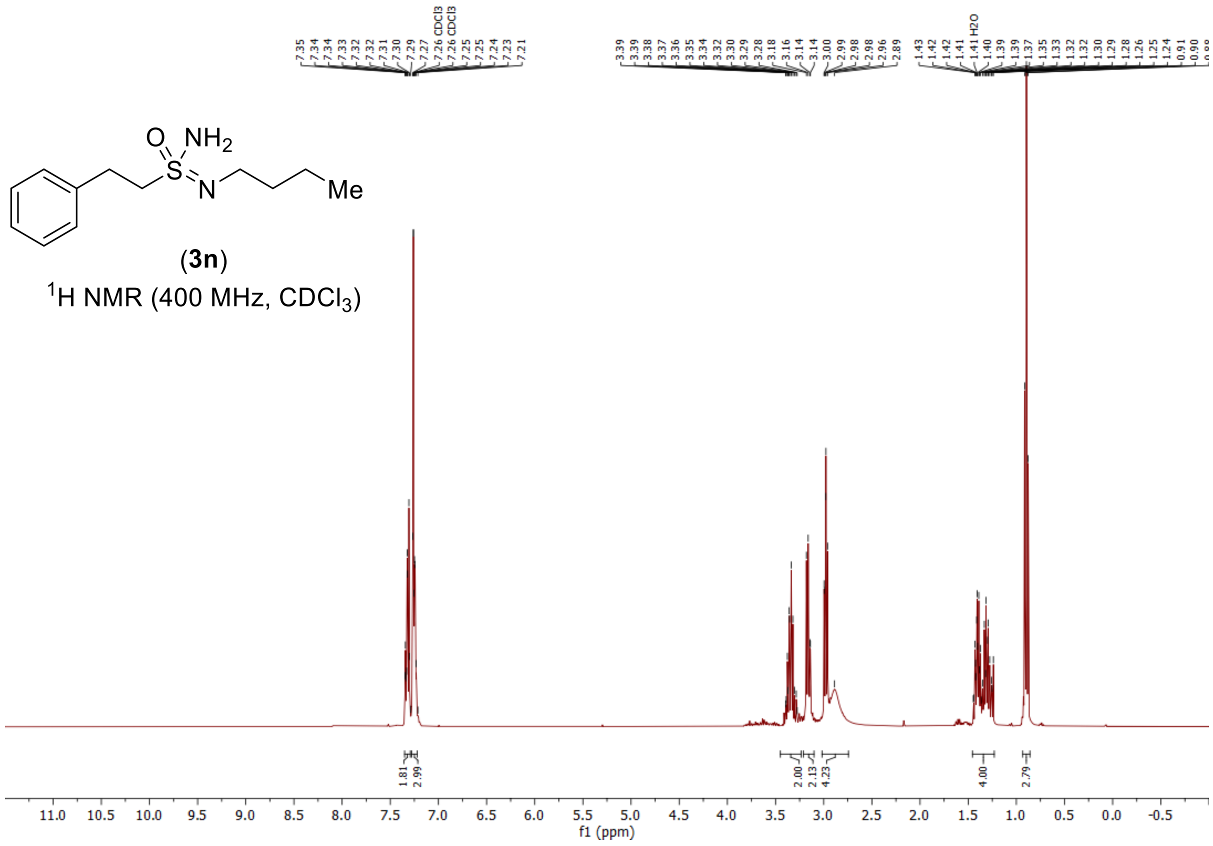
O NH2
S
N
Me
(3n)
1H NMR (400 MHz, CDCl3)
f1 (ppm)

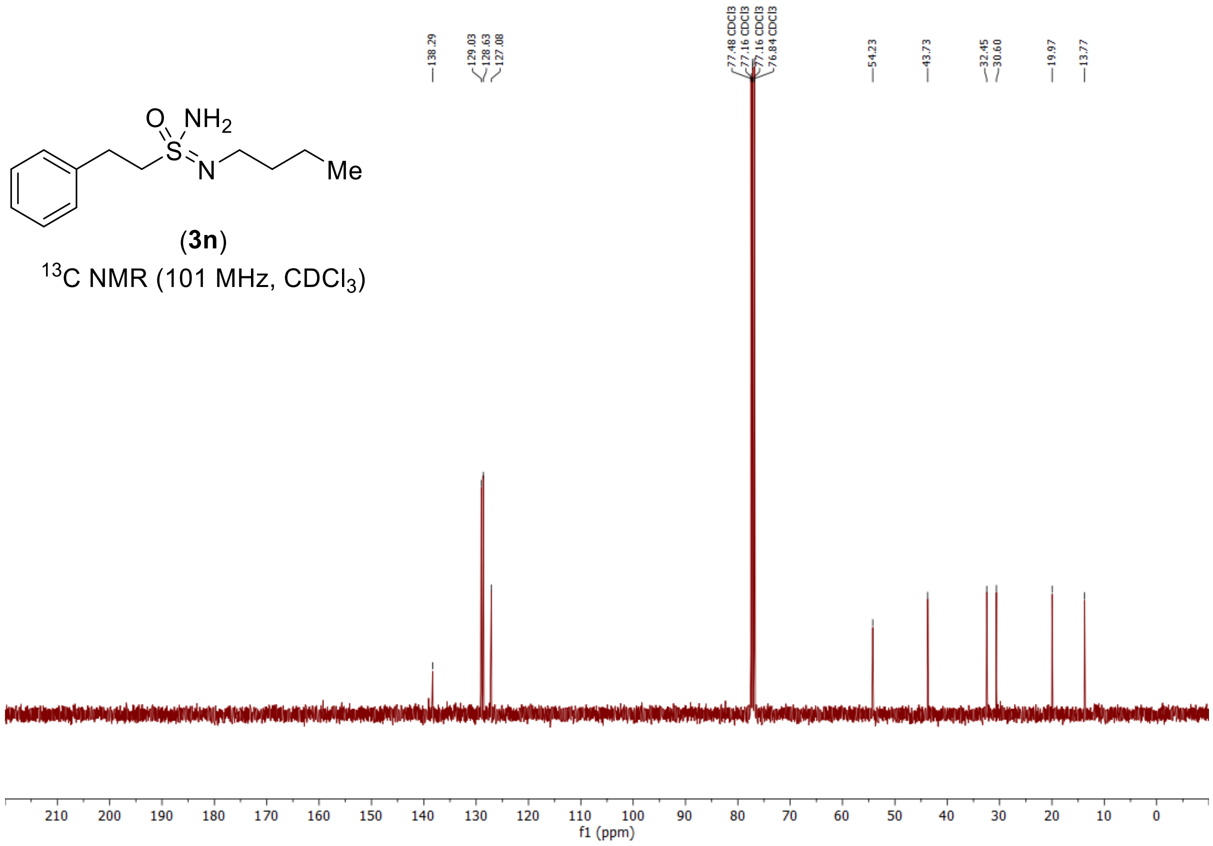
O NH2
S
N
Me
(3n)
13C NMR (101 MHz, CDCl3)
f1 (ppm)

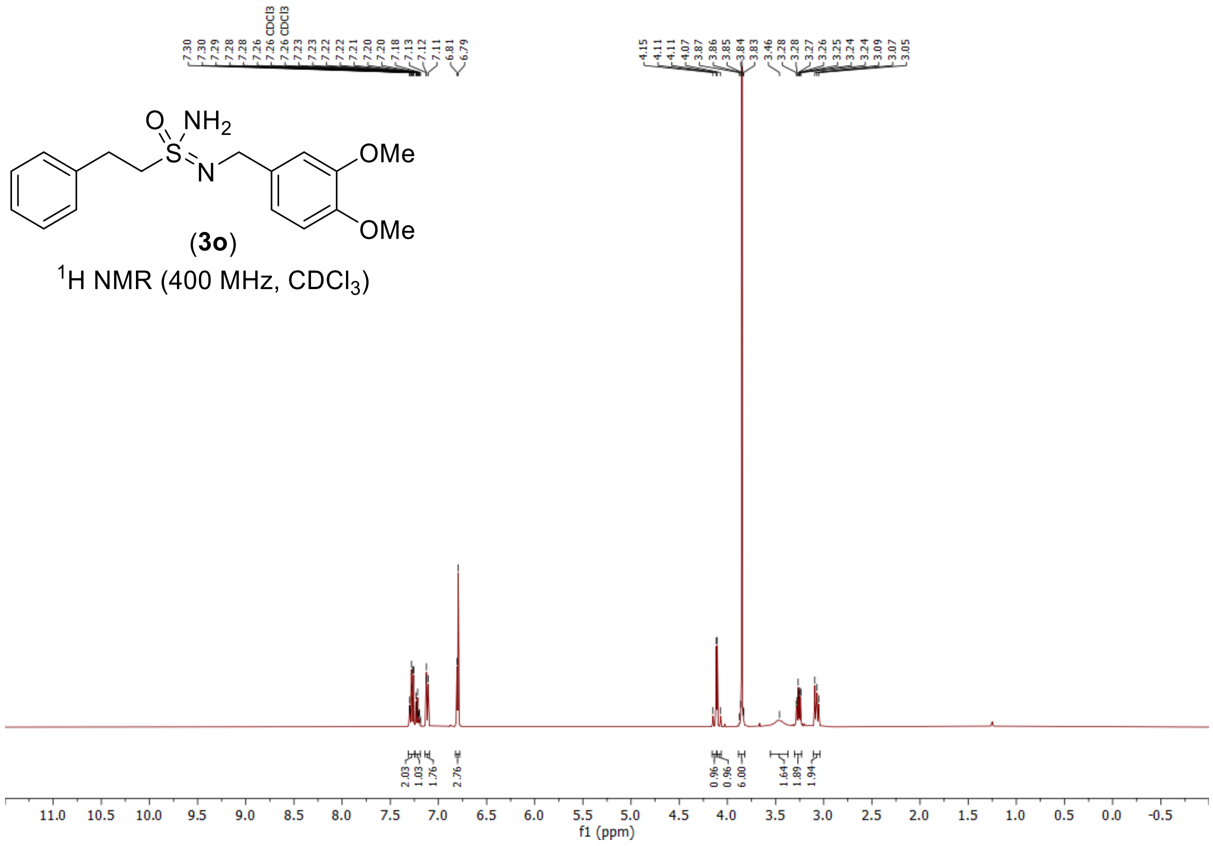

NH2
OMe
OMe
(3o)
1H NMR (400 MHz, CDCl3)
7.30 7.30 7.29 7.28 7.28 7.26 CDCl3 7.26 CDCl3 7.26 CDCl3 7.23 7.23 7.22 7.22 7.21 7.20 7.20 7.18 7.13 7.12 7.11 6.81 6.79
4.15 4.11 4.11 4.07 3.87 3.86 3.85 3.84 3.83 3.46 3.28 3.28 3.27 3.26 3.25 3.24 3.24 3.09 3.07 3.05
2.03 1.03 1.76 2.76 0.96 0.96 6.00 1.64 1.89 1.94
f1 (ppm)

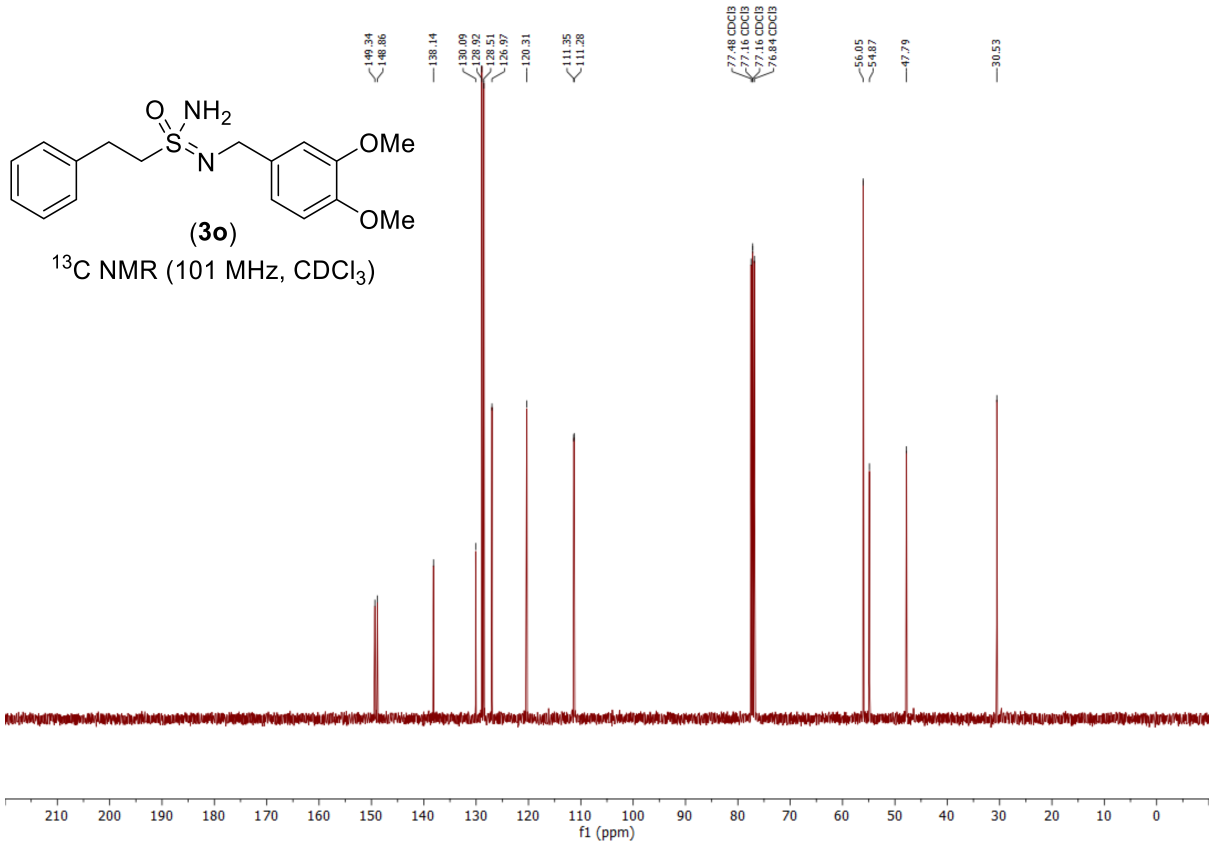

NH2
OMe
OMe
(3o)
13C NMR (101 MHz, CDCl3)
149.34 148.86 138.14 130.09 128.92 128.51 126.97 120.31 111.35 111.28
77.48 CDCl3 77.16 CDCl3 77.16 CDCl3 76.84 CDCl3
56.05 54.87 47.79 30.53
f1 (ppm)

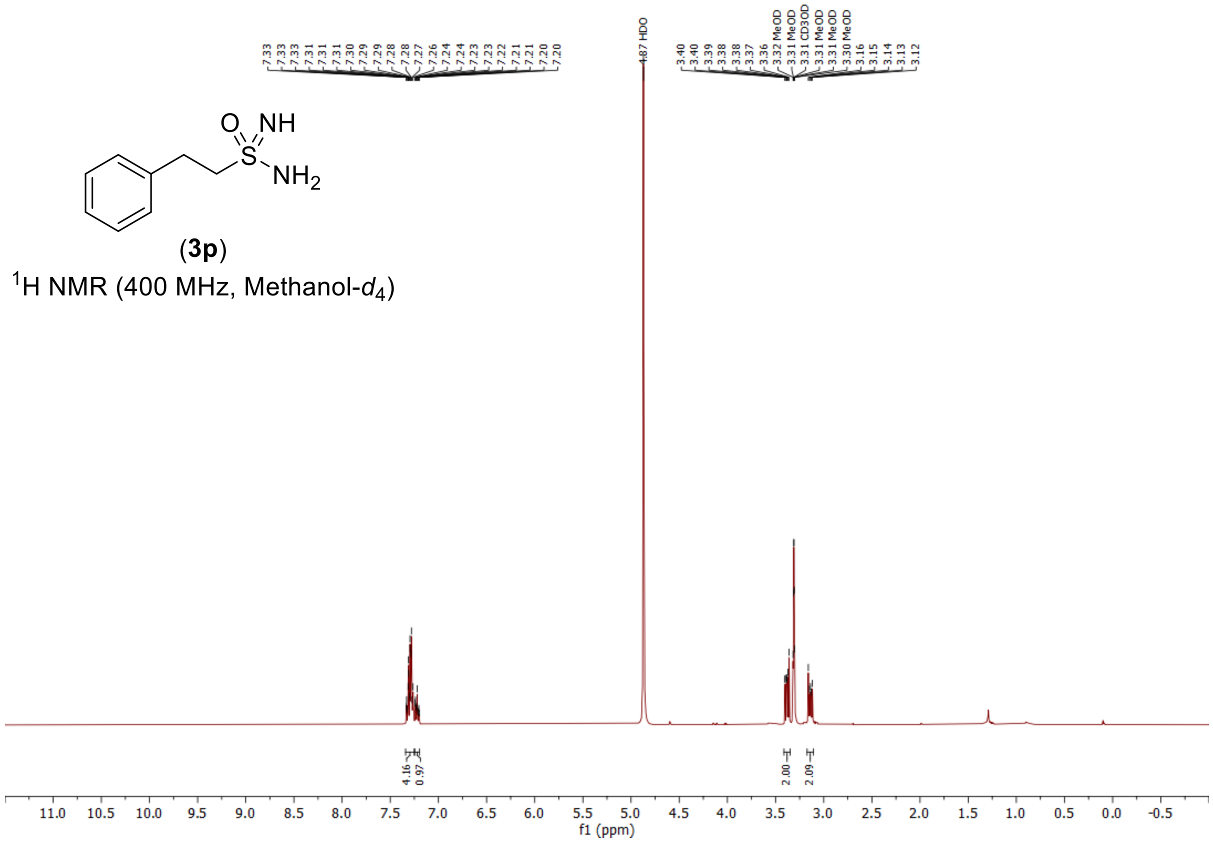
O NH
S
NH2
(3p)
1H NMR (400 MHz, Methanol-d4)
7.33
7.33
7.33
7.31
7.31
7.31
7.30
7.29
7.29
7.28
7.28
7.27
7.26
7.24
7.24
7.23
7.23
7.22
7.21
7.21
7.20
7.20
4.87 HDO
3.40
3.40
3.39
3.38
3.38
3.37
3.36
3.32 MeOD
3.31 MeOD
3.31 CD3OD
3.31 MeOD
3.31 MeOD
3.30 MeOD
3.16
3.15
3.14
3.13
3.12
4.16
0.97
2.00
2.09
f1 (ppm)

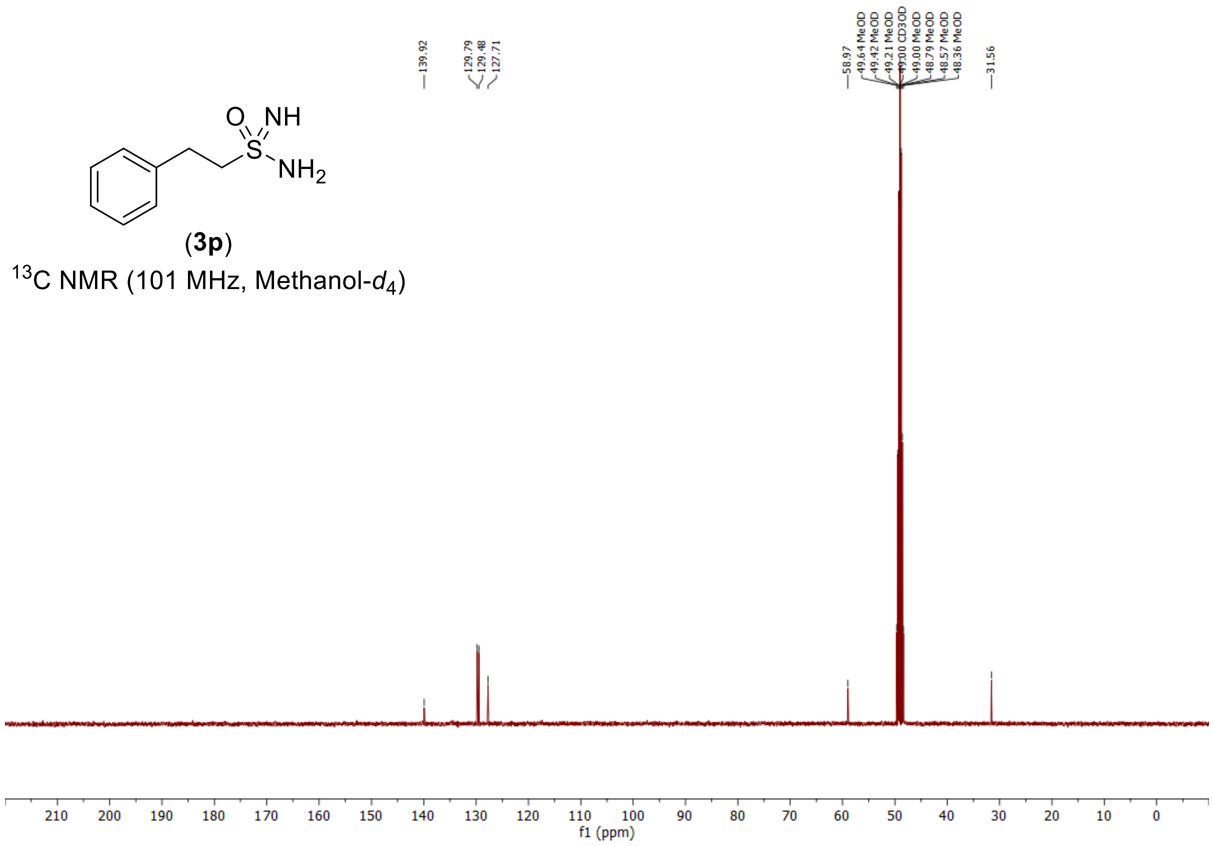
O NH
S
NH2
(3p)
13C NMR (101 MHz, Methanol-d4)
139.92
129.79
129.48
127.71
58.97
49.64 MeOD
49.42 MeOD
49.21 MeOD
49.00 CD3OD
49.00 MeOD
48.79 MeOD
48.57 MeOD
48.36 MeOD
31.56
f1 (ppm)

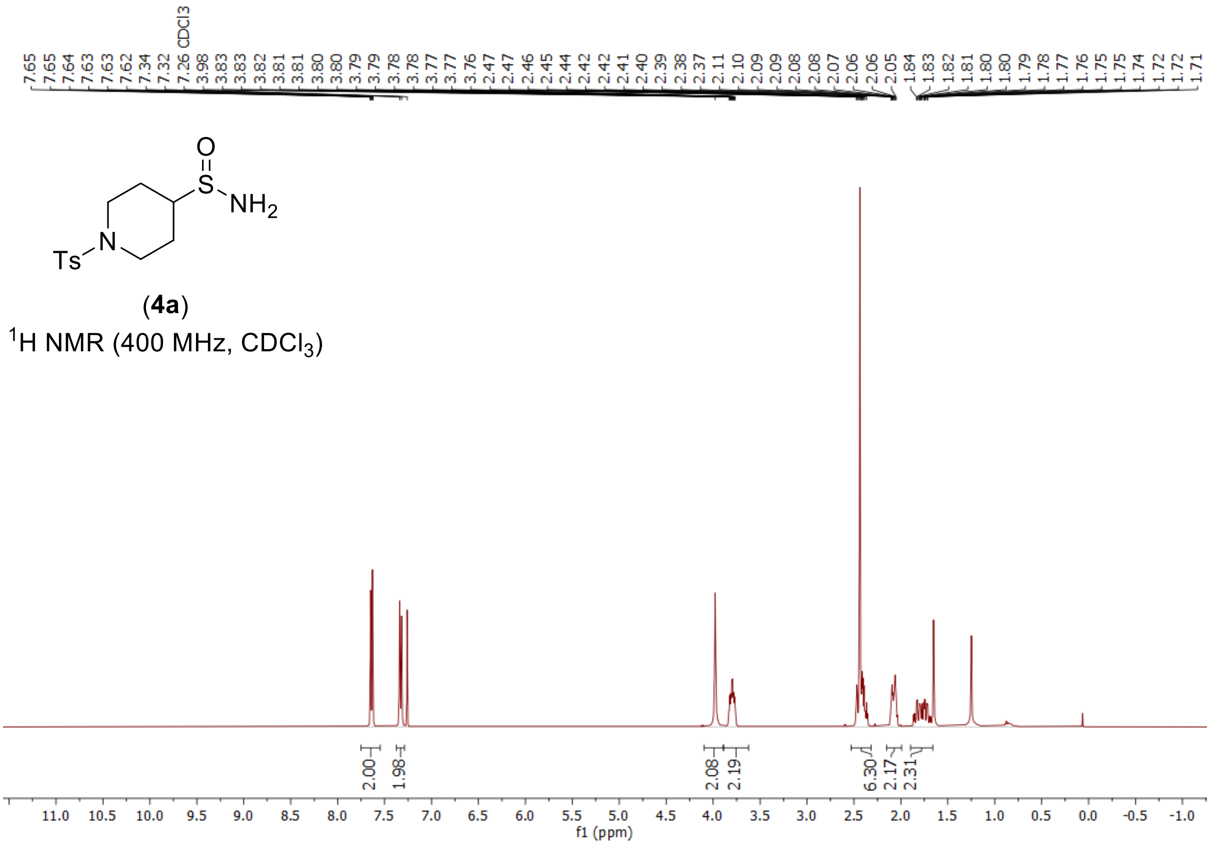

(**4a**)

^{1}H NMR (400 MHz, $CDCl_3$)

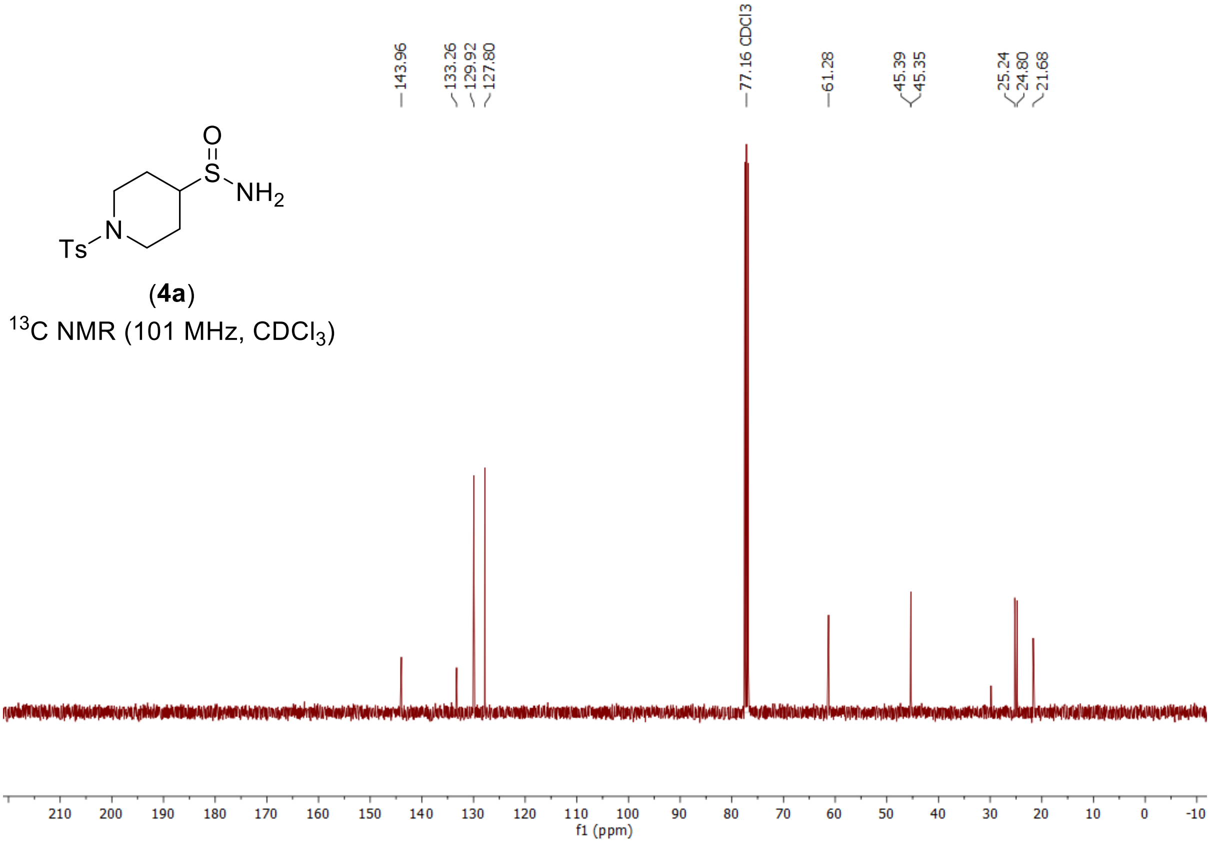

(**4a**)

^{13}C NMR (101 MHz, $CDCl_3$)

(**4c**)

^{1}H NMR (400 MHz, $CDCl_3$)

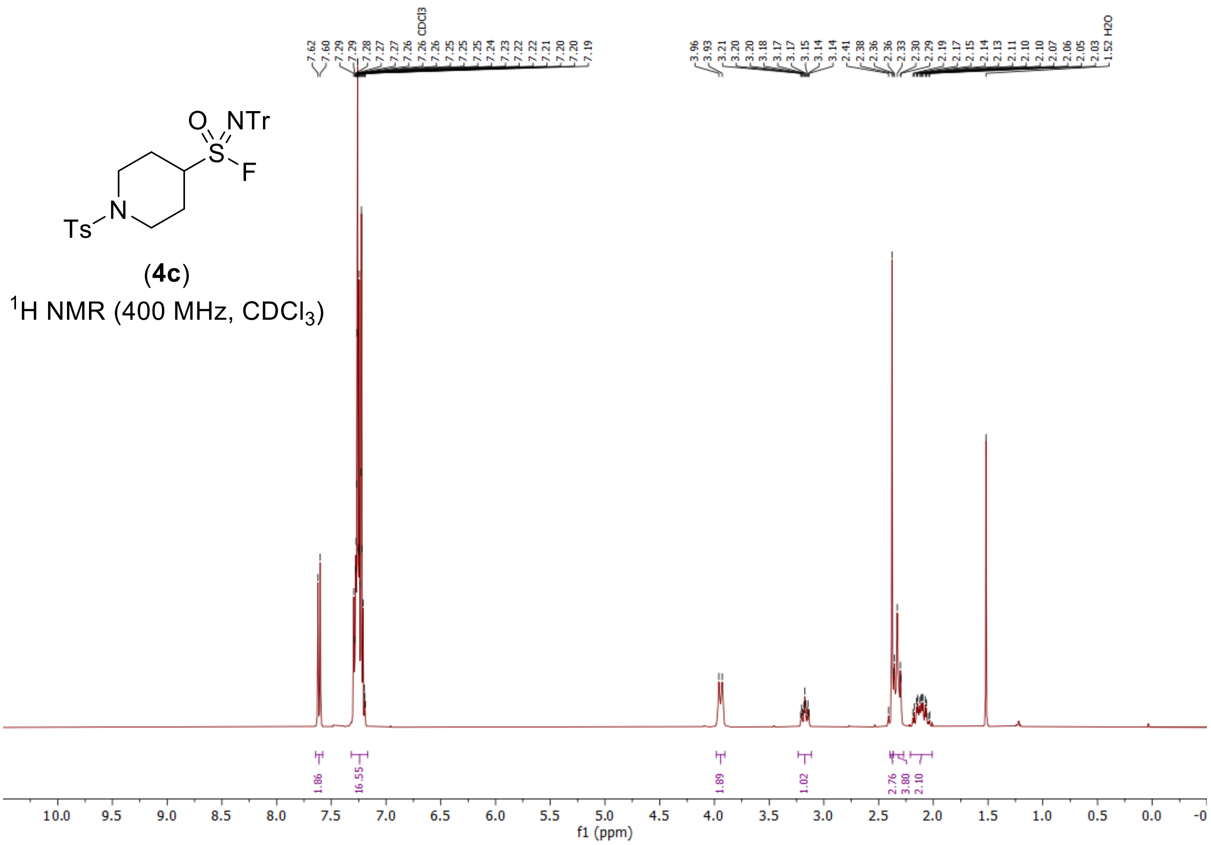

(**4c**)

^{13}C NMR (101 MHz, $CDCl_3$)

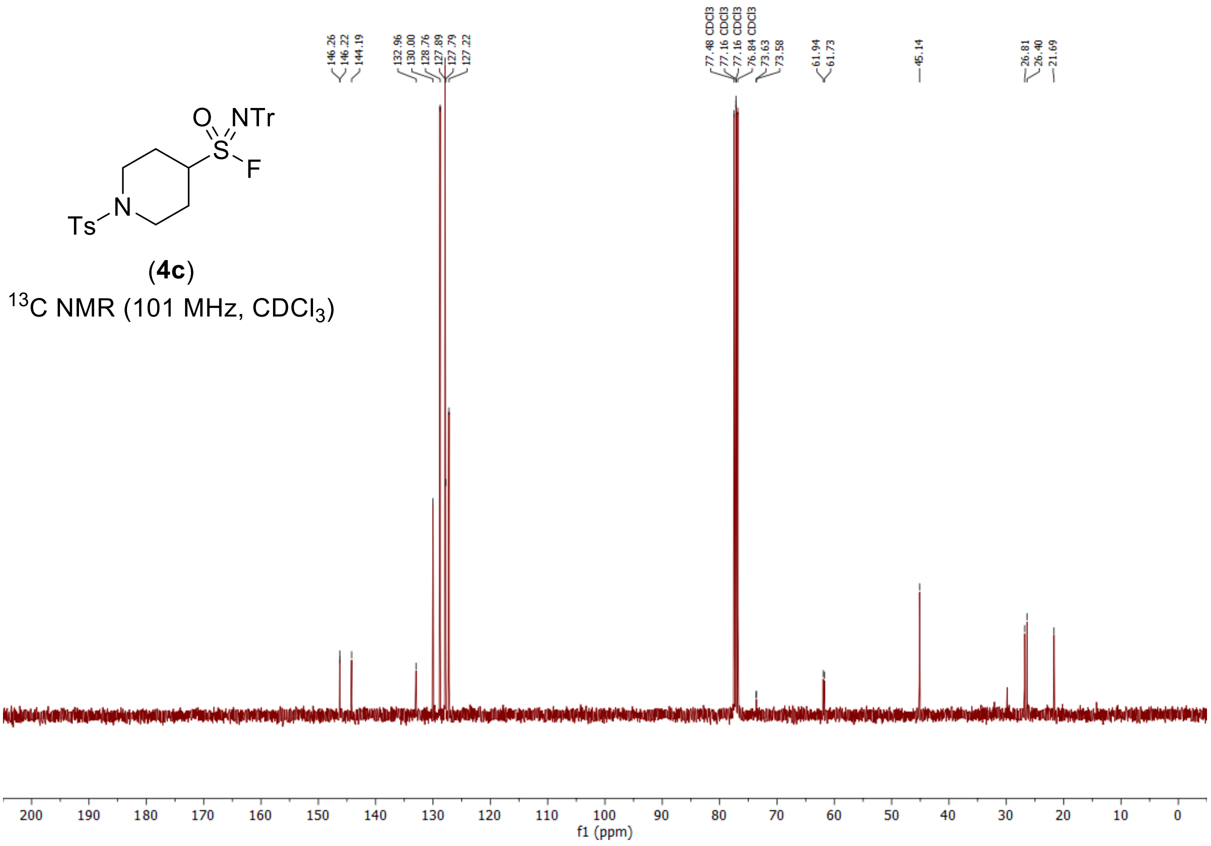

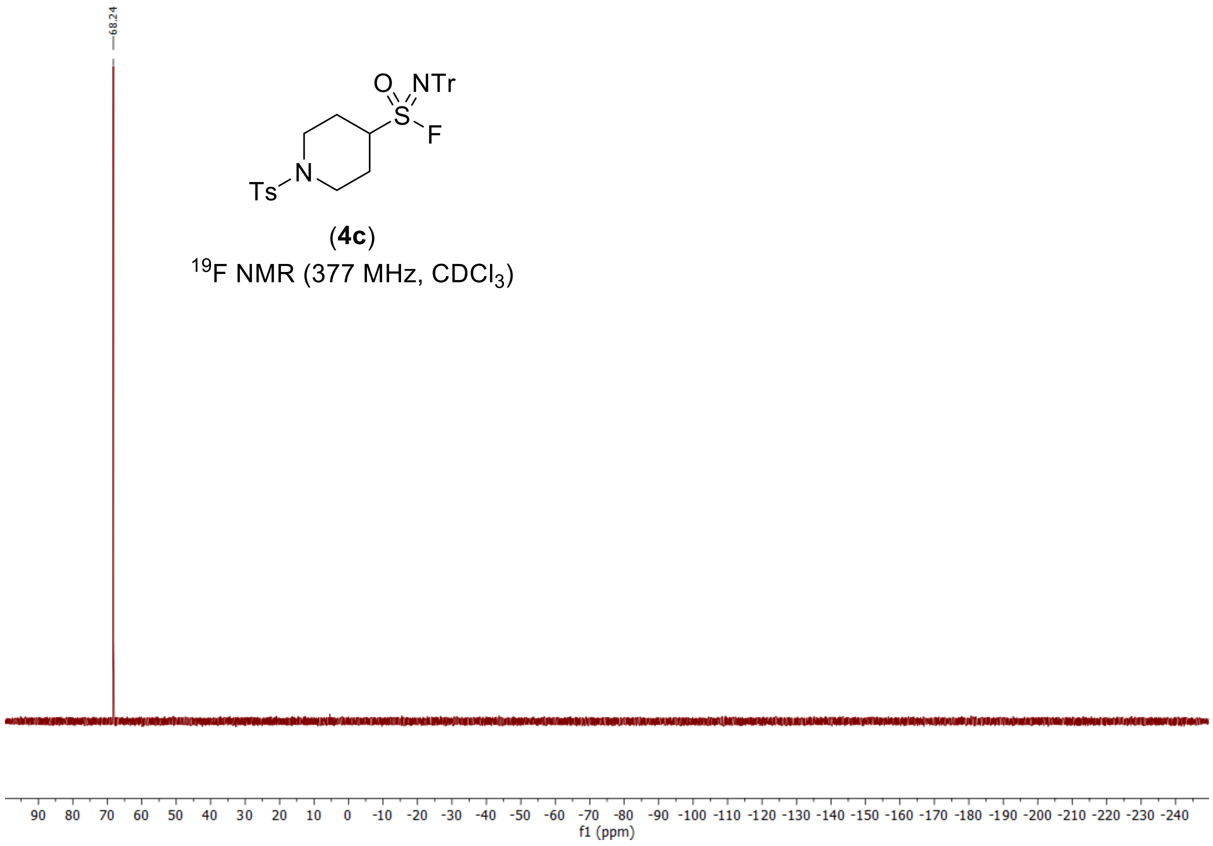

68.24
O
NTr
S
F
Ts
N
(4c)
19F NMR (377 MHz, CDCl3)
90 80 70 60 50 40 30 20 10 0 -10 -20 -30 -40 -50 -60 -70 -80 -90 -100 -110 -120 -130 -140 -150 -160 -170 -180 -190 -200 -210 -220 -230 -240
f1 (ppm)

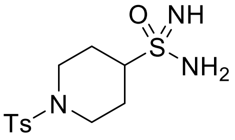

(**4d**)

^{1}H NMR (400 MHz, DMSO-d_6)

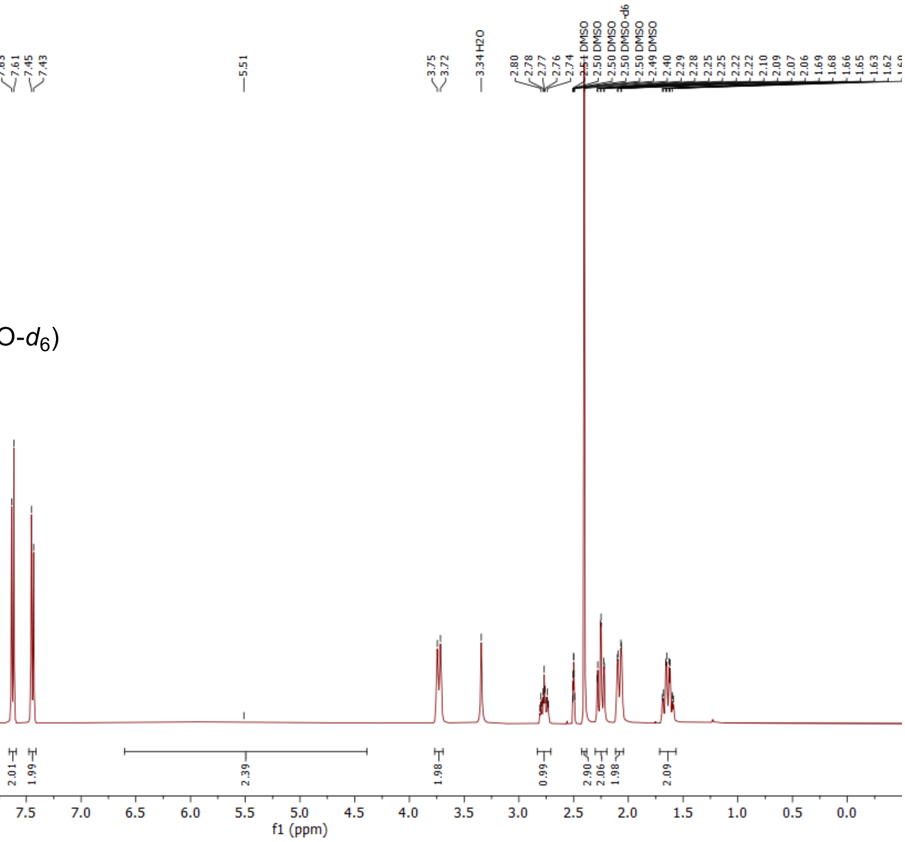

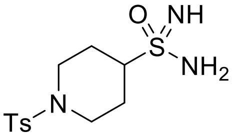

(**4d**)

^{13}C NMR (101 MHz, DMSO-d_6)

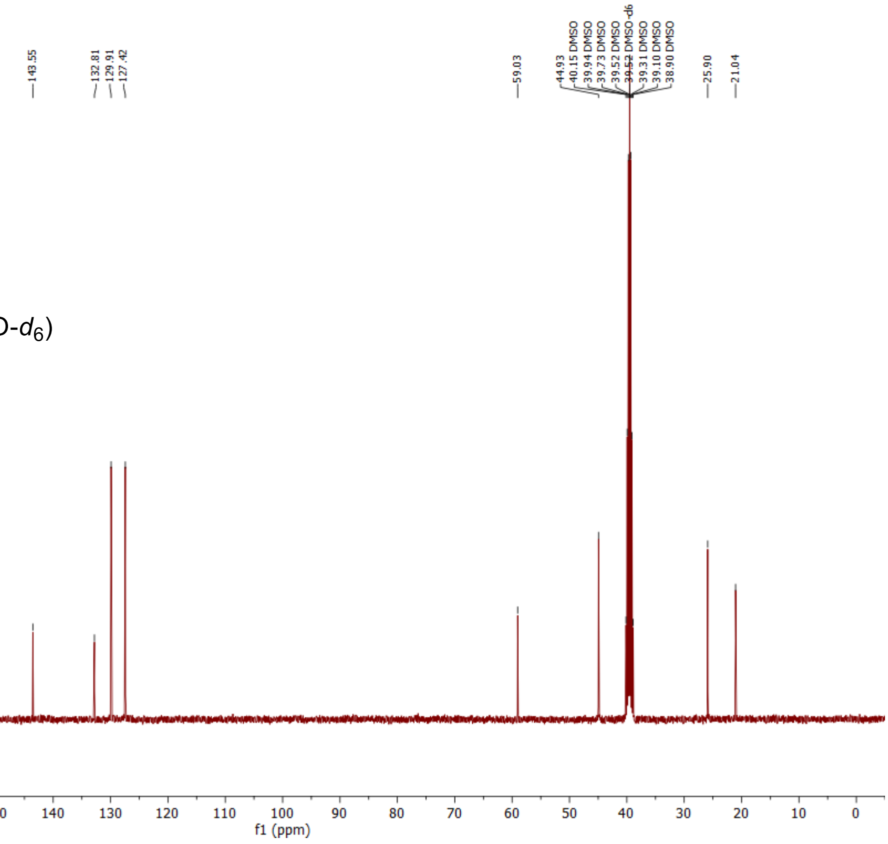

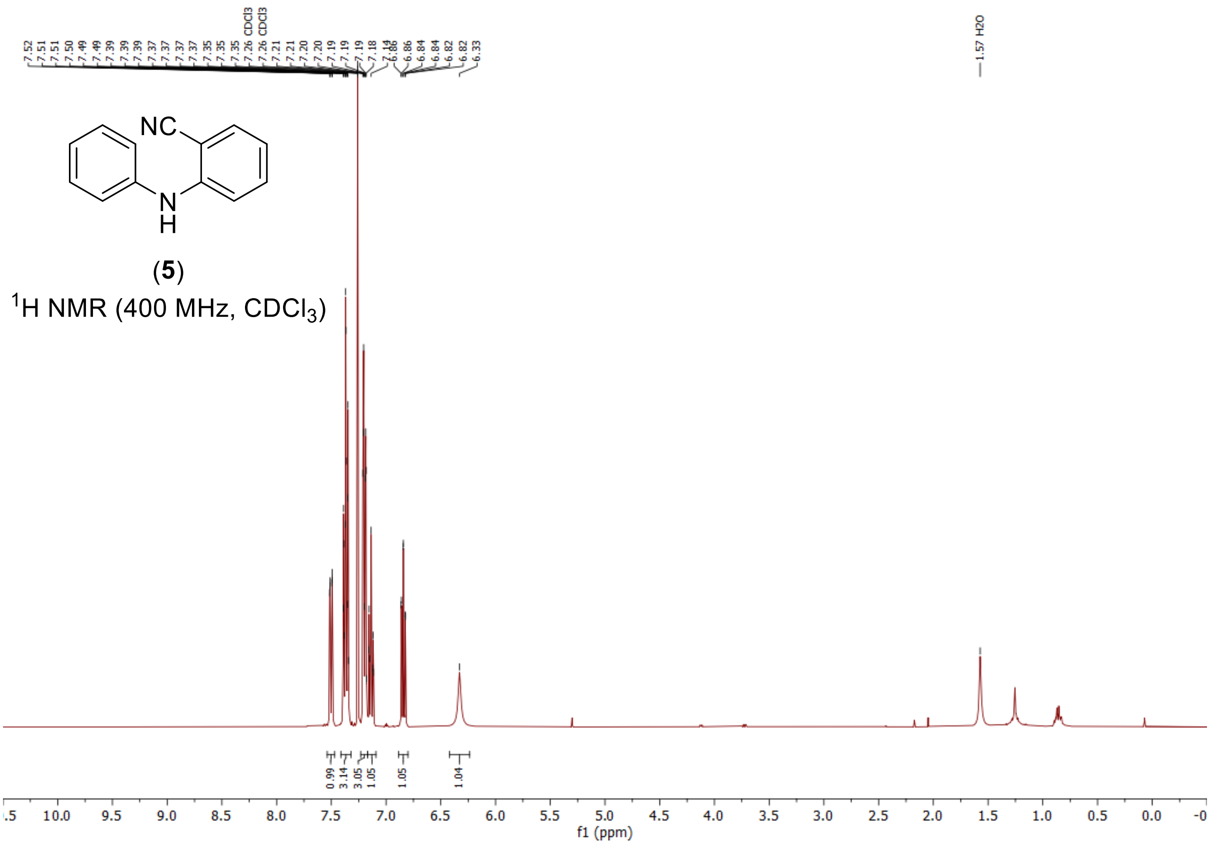
NC
N
H
(5)
1H NMR (400 MHz, CDCl3)
1.57 H2O
f1 (ppm)

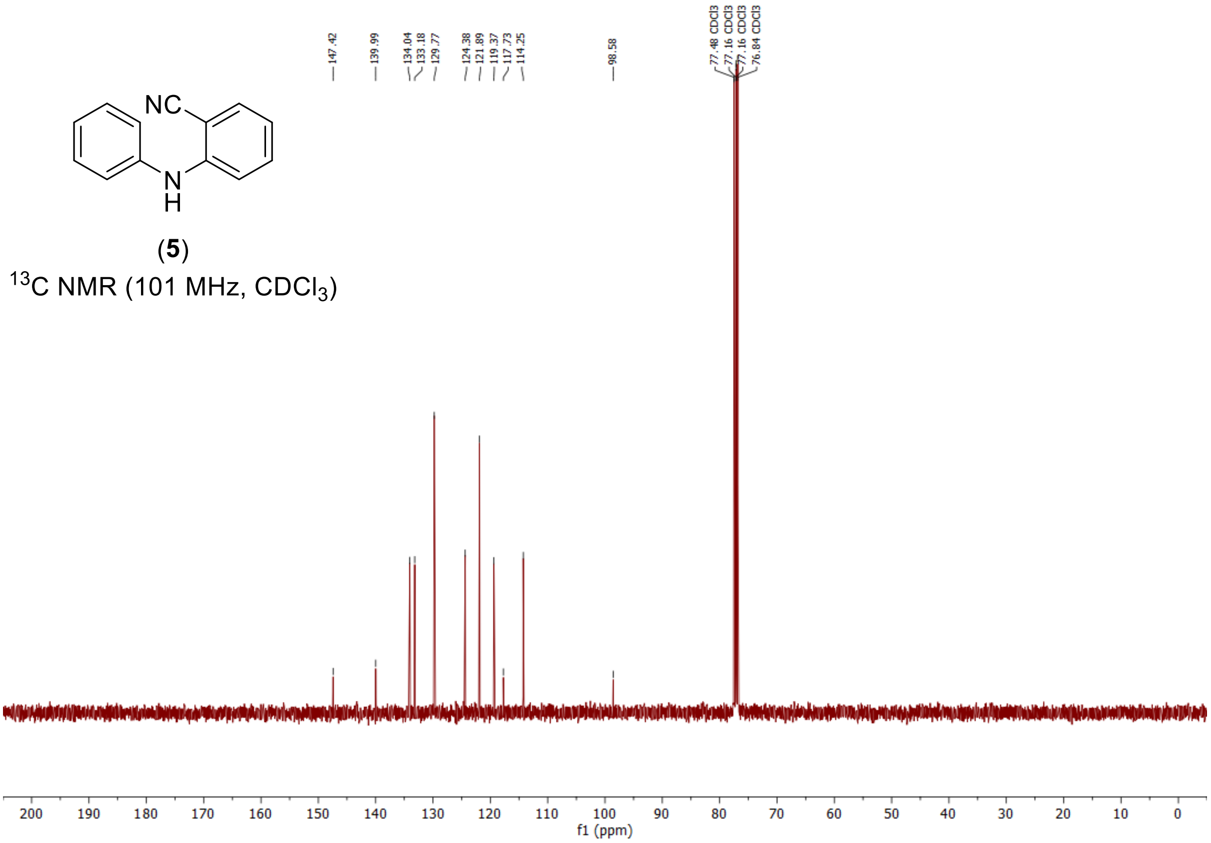
NC
N
H
(5)
13C NMR (101 MHz, CDCl3)
147.42
139.99
134.04
133.18
129.77
124.38
121.89
119.37
117.73
114.25
98.58
77.48 CDCl3
77.16 CDCl3
77.16 CDCl3
76.84 CDCl3
f1 (ppm)

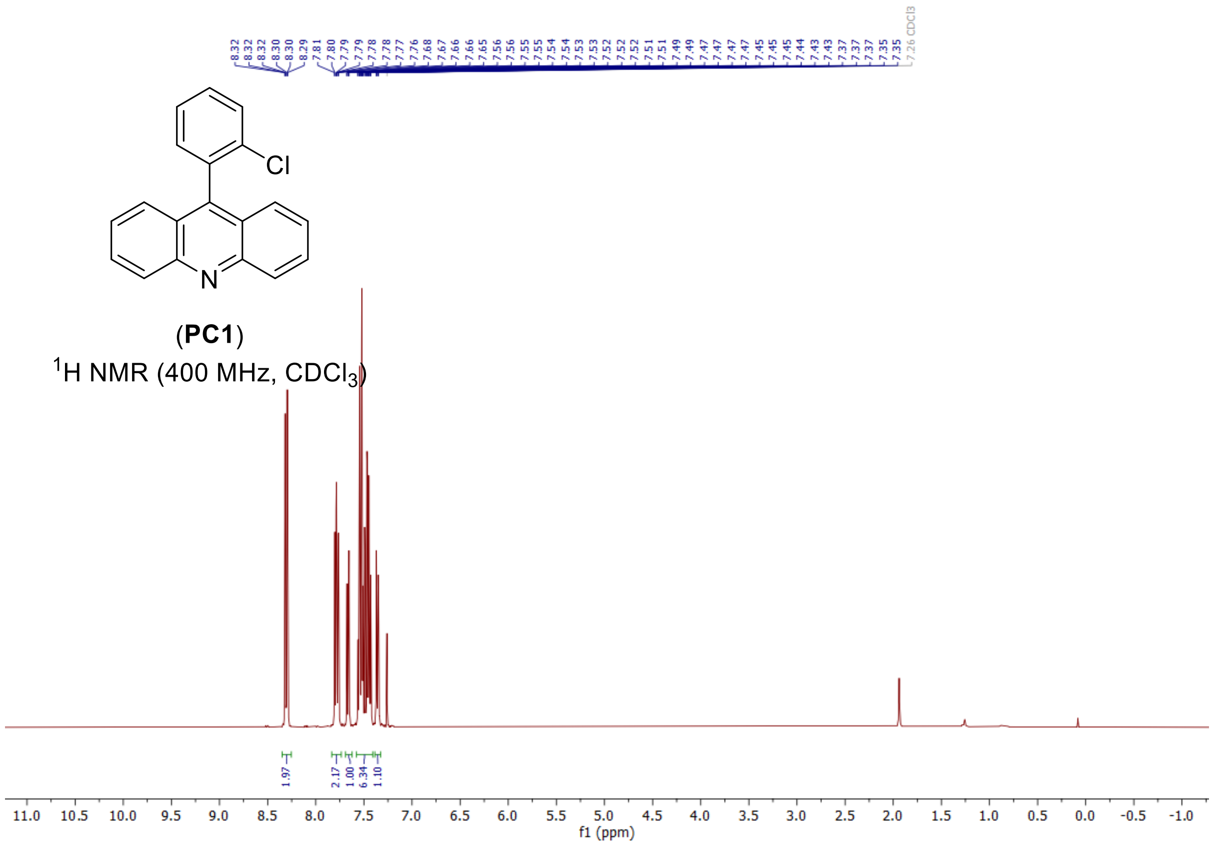

Cl
N
(PC1)
1H NMR (400 MHz, CDCl3)
7.26 CDCl3
f1 (ppm)

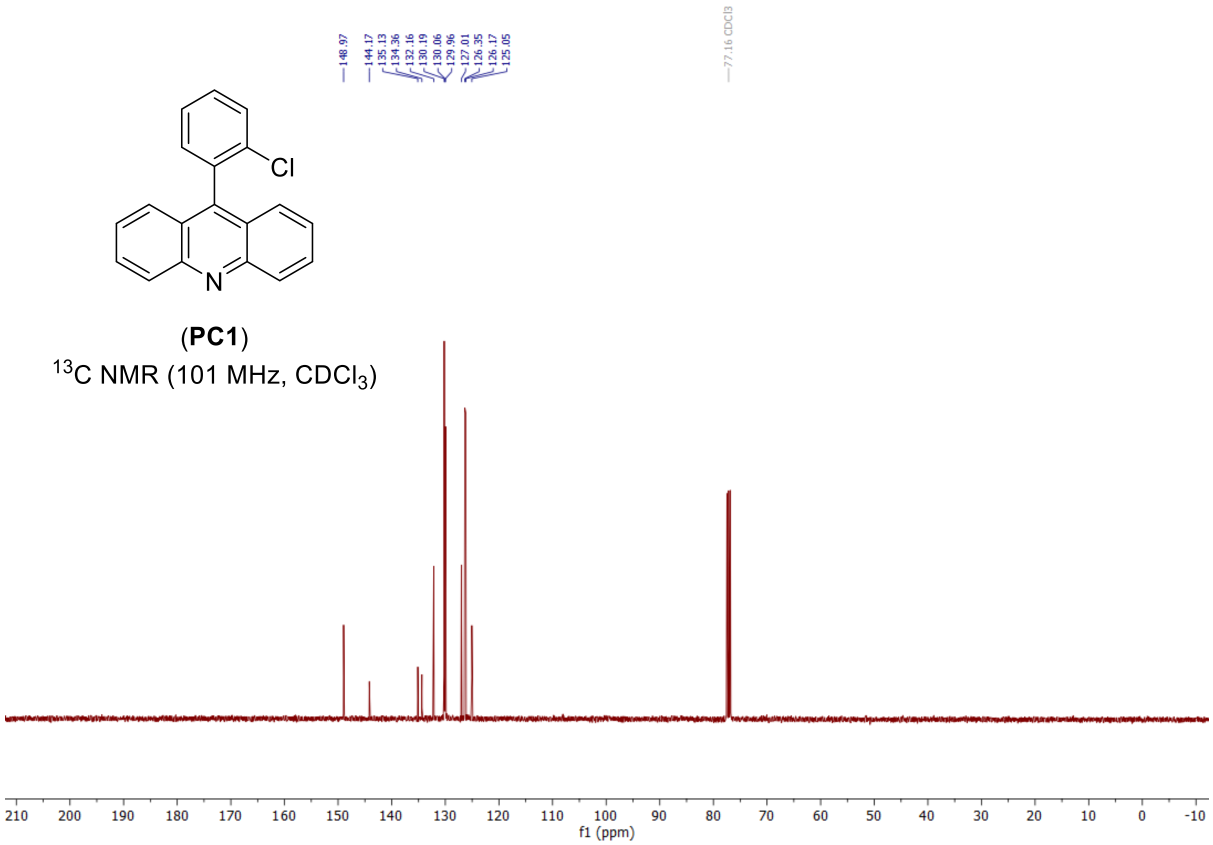

148.97
144.17
135.13
134.36
132.16
130.19
130.06
129.96
127.01
126.35
126.17
125.05
77.16 CDCl3
Cl
N
(PC1)
13C NMR (101 MHz, CDCl3)
f1 (ppm)

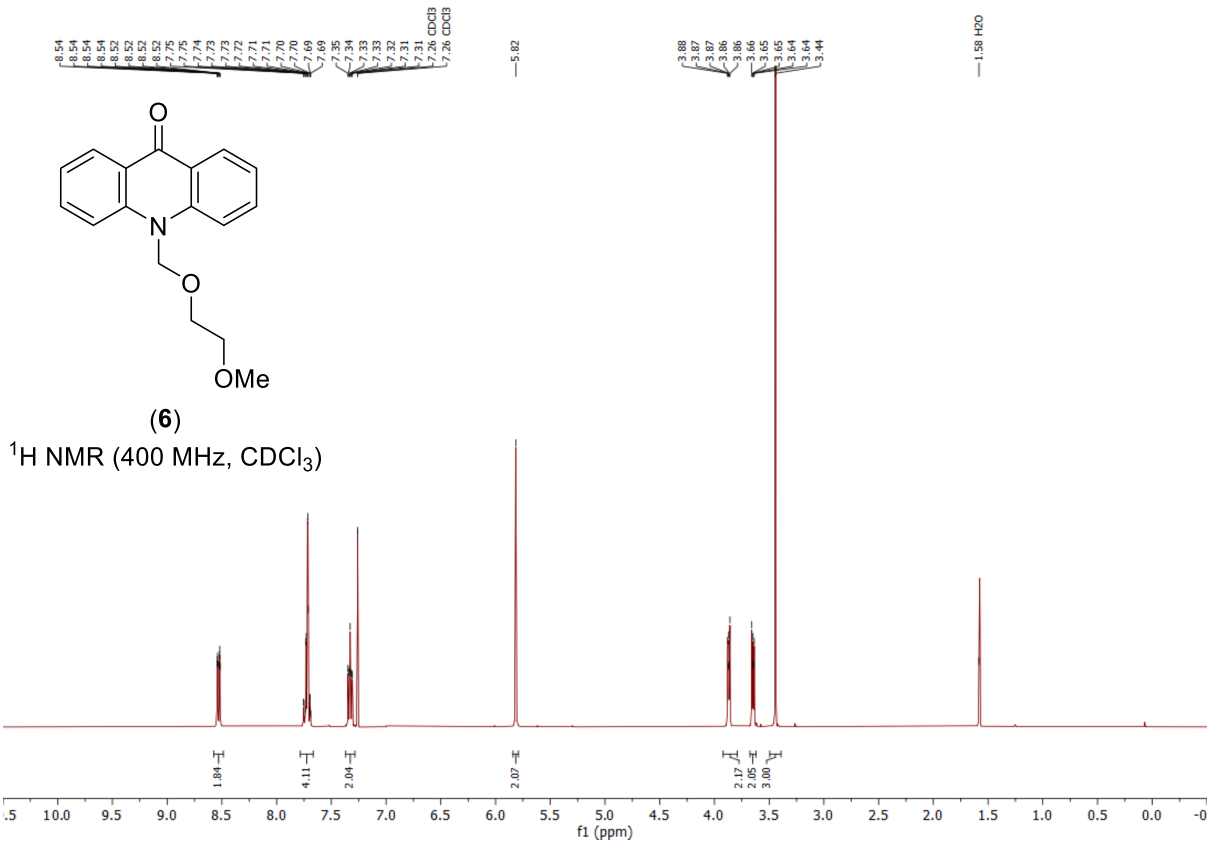

(**6**)

^1H NMR (400 MHz, $CDCl_3$)

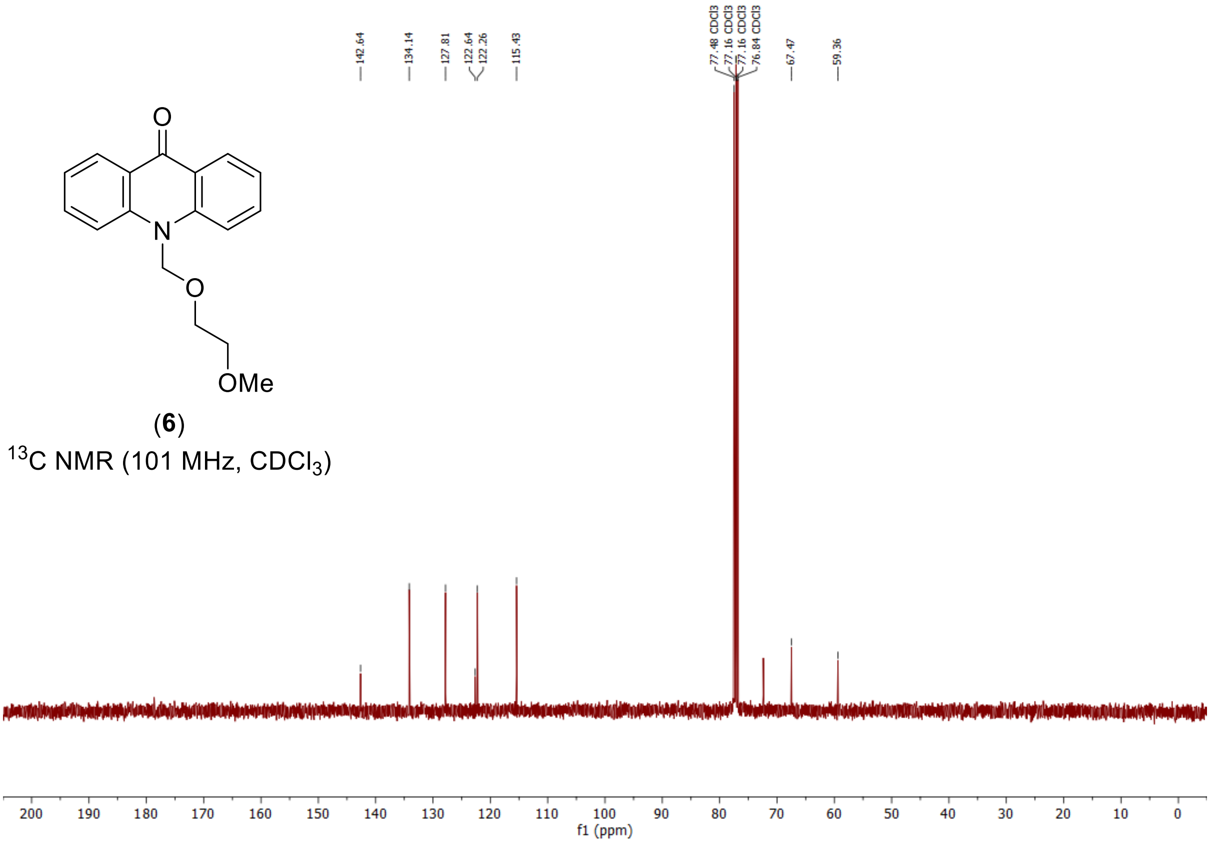

(**6**)

^{13}C NMR (101 MHz, $CDCl_3$)

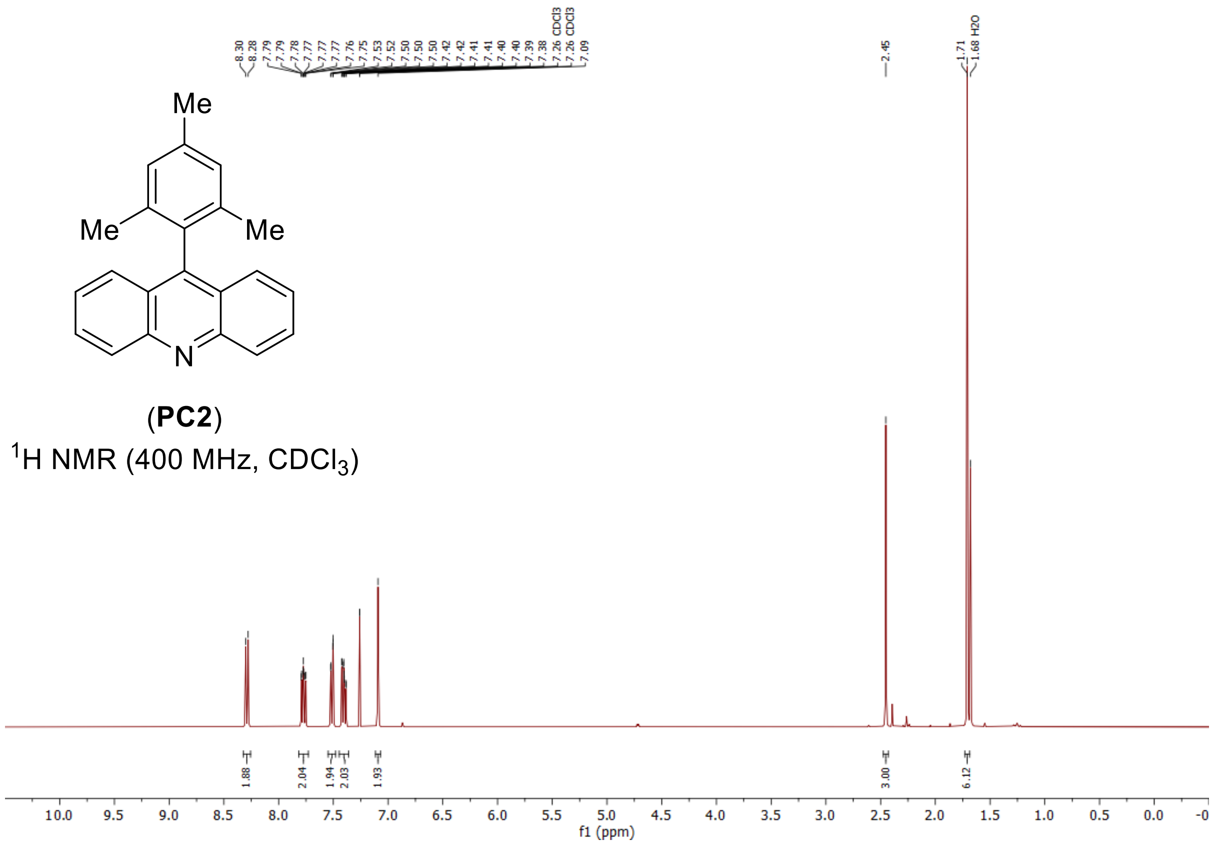
Me
Me
Me
N
(PC2)
^{1}H NMR (400 MHz, $CDCl_3$)
8.30
8.28
7.79
7.79
7.78
7.77
7.77
7.77
7.76
7.75
7.53
7.52
7.50
7.50
7.50
7.42
7.42
7.41
7.40
7.40
7.39
7.38
7.26 CDCl3
7.26 CDCl3
7.09
2.45
1.71
1.68 H2O
1.88
2.04
1.94
2.03
1.93
3.00
6.12
f1 (ppm)

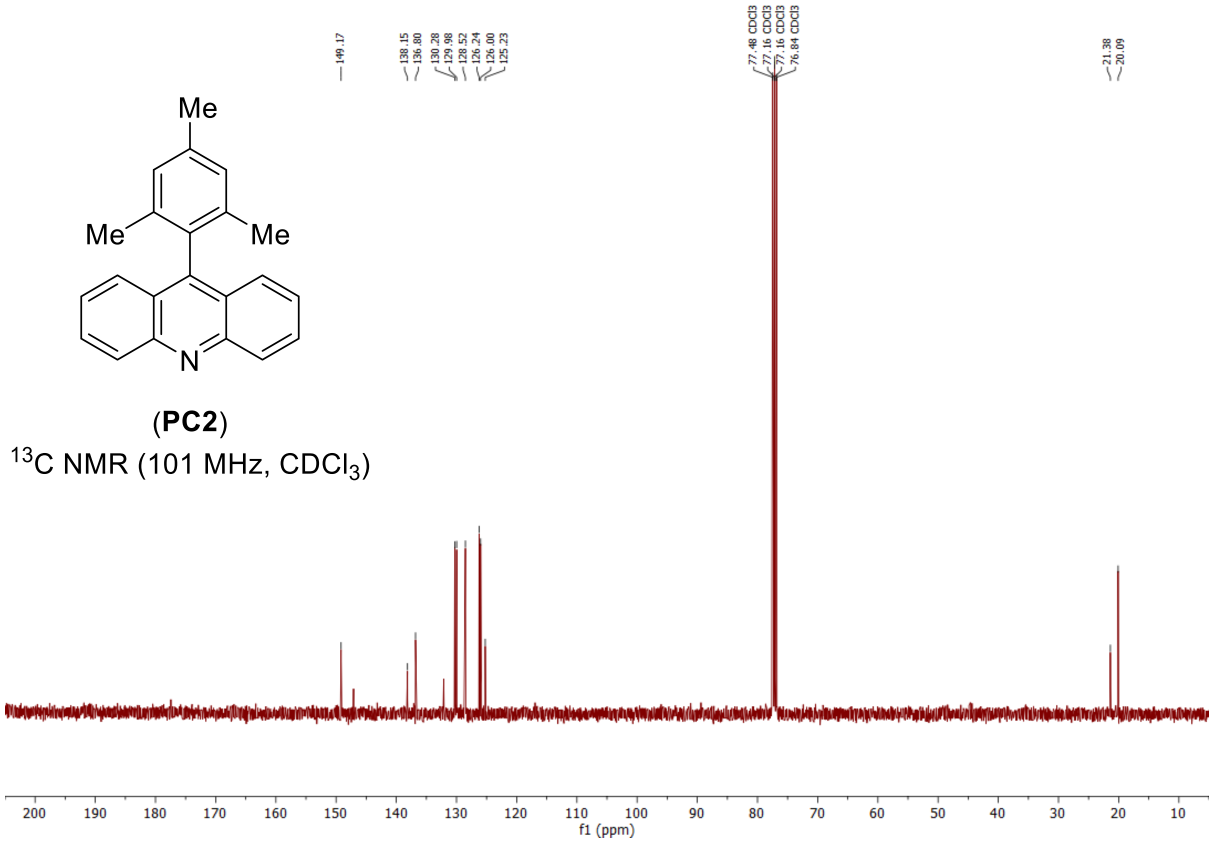
Me
Me
Me
N
(PC2)
^{13}C NMR (101 MHz, $CDCl_3$)
149.17
138.15
136.80
130.28
129.98
128.52
126.24
126.00
125.23
77.48 CDCl3
77.16 CDCl3
77.16 CDCl3
76.84 CDCl3
21.38
20.09
f1 (ppm)

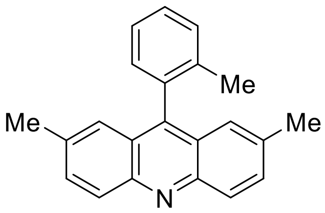

(**PC4**)
^{1}H NMR (400 MHz, $CDCl_3$)

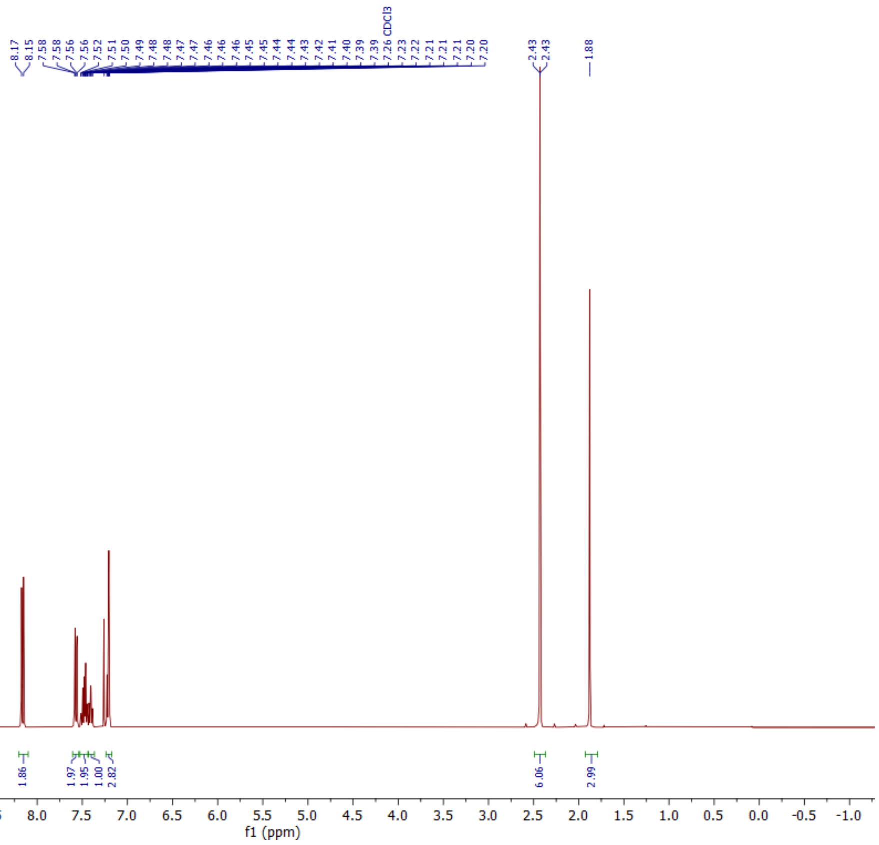

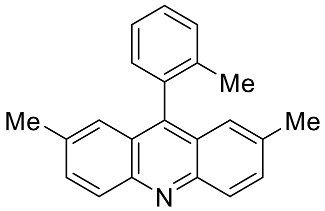

(**PC4**)
^{13}C NMR (101 MHz, $CDCl_3$)

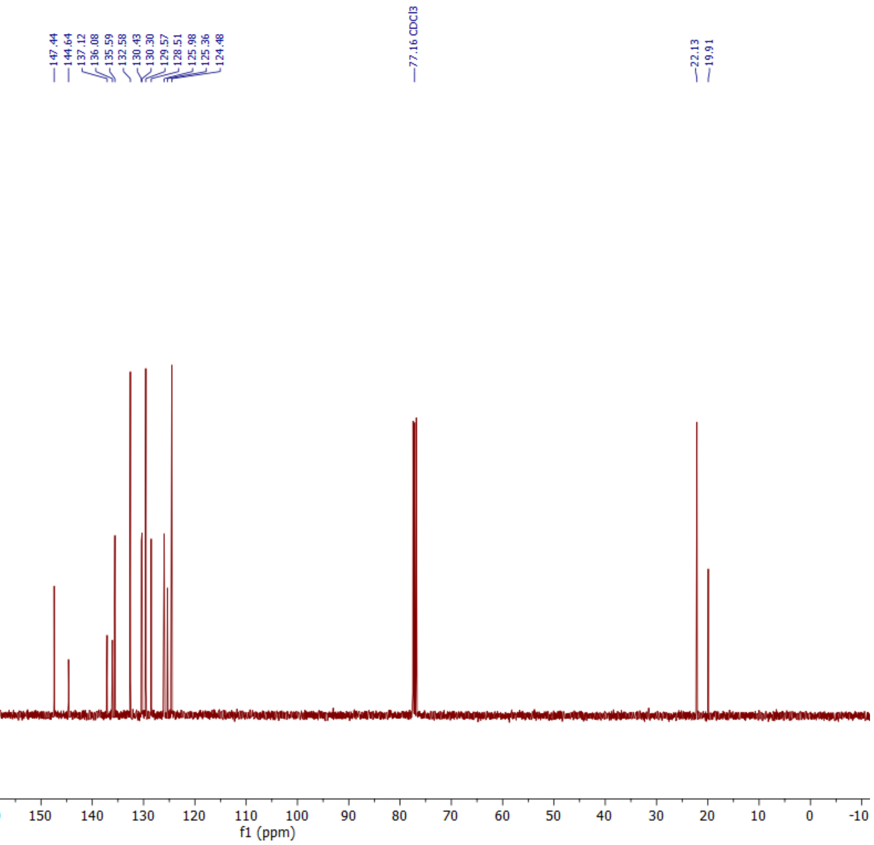

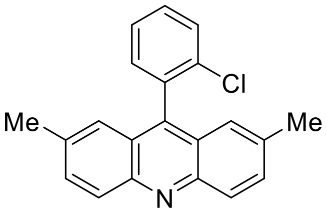

(**PC5**)
^{1}H NMR (400 MHz, $CDCl_3$)

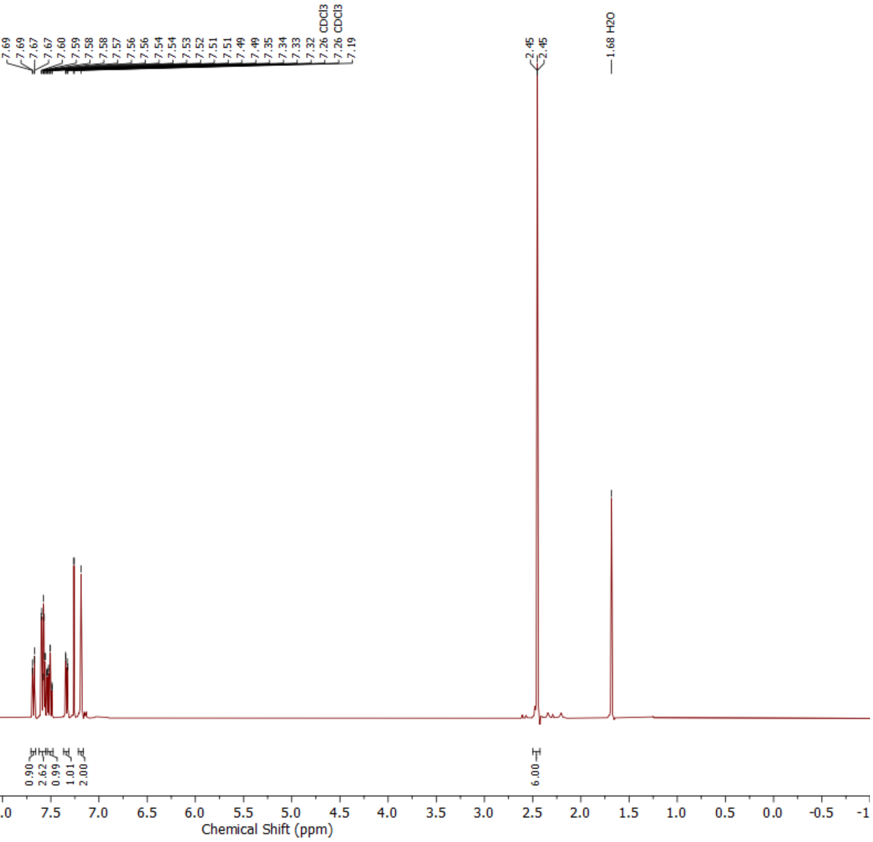

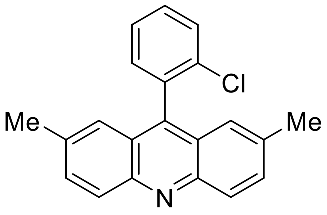

(**PC5**)
^{13}C NMR (101 MHz, $CDCl_3$)

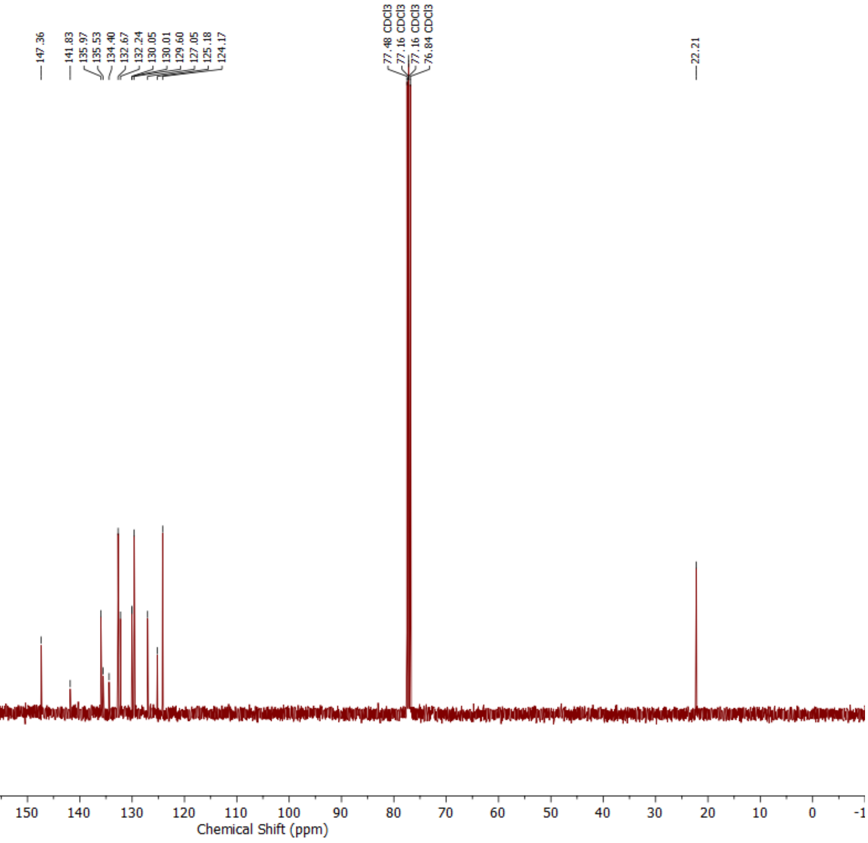

Supplement: Supplementary file 1 — ja3c07974_si_001.pdf [file ja3c07974_si_001.pdf]
